# LIM kinase inhibitors disrupt mitotic microtubule organization and impair tumor cell proliferation

**DOI:** 10.18632/oncotarget.6288

**Published:** 2015-11-03

**Authors:** Katerina Mardilovich, Mark Baugh, Diane Crighton, Dominika Kowalczyk, Mads Gabrielsen, June Munro, Daniel R. Croft, Filipe Lourenco, Daniel James, Gabriella Kalna, Lynn McGarry, Oliver Rath, Emma Shanks, Mathew J. Garnett, Ultan McDermott, Joanna Brookfield, Mark Charles, Tim Hammonds, Michael F. Olson

**Affiliations:** ^1^ Cancer Research UK Beatson Institute, Garscube Estate, Glasgow, UK; ^2^ Cancer Genome Project, Wellcome Trust Sanger Institute, Hinxton, UK; ^3^ Cancer Research Technology Discovery Laboratories, Jonas Webb Building, Babraham Research Campus, Cambridge, UK; ^4^ Cancer Research Technology Discovery Laboratories, London Bioscience Innovation Centre, London, UK

**Keywords:** cytoskeleton, microtubule, inhibitor, LIMK, kinase

## Abstract

The actin and microtubule cytoskeletons are critically important for cancer cell proliferation, and drugs that target microtubules are widely-used cancer therapies. However, their utility is compromised by toxicities due to dose and exposure. To overcome these issues, we characterized how inhibition of the actin and microtubule cytoskeleton regulatory LIM kinases could be used in drug combinations to increase efficacy. A previously-described LIMK inhibitor (LIMKi) induced dose-dependent microtubule alterations that resulted in significant mitotic defects, and increased the cytotoxic potency of microtubule polymerization inhibitors. By combining LIMKi with 366 compounds from the GSK Published Kinase Inhibitor Set, effective combinations were identified with kinase inhibitors including EGFR, p38 and Raf. These findings encouraged a drug discovery effort that led to development of CRT0105446 and CRT0105950, which potently block LIMK1 and LIMK2 activity *in vitro*, and inhibit cofilin phosphorylation and increase αTubulin acetylation in cells. CRT0105446 and CRT0105950 were screened against 656 cancer cell lines, and rhabdomyosarcoma, neuroblastoma and kidney cancer cells were identified as significantly sensitive to both LIMK inhibitors. These large-scale screens have identified effective LIMK inhibitor drug combinations and sensitive cancer types. In addition, the LIMK inhibitory compounds CRT0105446 and CRT0105950 will enable further development of LIMK-targeted cancer therapy.

## INTRODUCTION

The LIM kinases 1 and 2 (LIMK1 and LIMK2) are central regulators of cytoskeletal dynamics [1, 2]. Although their regulation of cofilin activity and filamentous actin (F-actin) stability have been extensively studied [3], they also contribute to microtubule dynamics via less well characterized mechanisms [4–6]. Given their cytoskeleton-associated functions and indications of elevated expression in various cancer types, research has largely focussed on their roles in tumor cell migration, invasion and metastasis [7–9]. More recent studies have shown that the LIM kinases modulate additional activities that contribute to cancer development, including cell proliferation and survival [4–6]. LIMK activity also contributes to cancer cell resistance to various chemotherapeutic agents and ionizing radiation, while blocking LIMK activity reverses this resistance or even increases sensitivity [10–14]. These observations have spurred efforts directed towards the discovery of LIMK inhibitors as potential cancer therapeutic agents that could be used as single agents or as part of combination therapies [15–17].

In recent years, the focus of cancer drug discovery has evolved from developing chemotherapeutics that could be widely-used across multiple cancers to treatments tailored for specific tumor types. In particular, this has led to the development of targeted therapies that utilise unique molecular properties identified in malignant cells to preferentially target tumor cells, while reducing potential detrimental effects on normal cells [18]. However, molecularly-targeted therapies have not been as successful as originally anticipated, with high rates of failure and limited numbers of patients that manifest positive clinical responses.

In order to improve chances for targeted therapy success, two strategies have been considered. Firstly, as an alternative or complement to molecularly-targeted therapies, other proteins or biological processes may be important targets because of accessory or auxiliary functions they provide. For example, stresses induced by oncogenic transformation often lead to reliance upon heat-shock protein chaperones, such that inhibition of heat shock proteins including Hsp90, Hsp70 and Hsp27 has become a large area of cancer drug development [19]. Similarly, associations between human cancers and alterations in the actin and microtubule cytoskeletons have been well documented [20], suggesting that drugs targeting cytoskeleton regulators might be efficacious for the treatment of certain tumor types [15–17]. Unbiased screening may reveal drug sensitivities that reflect dependence on these auxiliary functions that would not be identified by genetic profiling.

A number of small molecule LIMK inhibitors have been discovered [6, 21–25] and several have been reported to be efficacious as single agent therapeutics on cancer cells including: breast [6, 26], pancreatic [27], prostate [5], cervical adenocarcinoma [6], fibromatosis [28], leukemia [4, 6] and glioma [29]. To identify how LIMK inhibitors might best be utilized, we carried out two cell-based screens. In the first, a LIMK inhibitor was combined with microtubule-targeting drugs, or the GSK published kinase inhibitor set (PKIS) of 366 small molecule kinase inhibitors [30], to identify combinations with positive interactions. In the second, two novel LIMK inhibitors we developed were used to screen 656 tumor cell lines to identify cancer types with significant sensitivity to their anti-proliferative effects. These large-scale and unbiased cell-based screens revealed a range of drug combinations that could be considered for further development, and identified tumor types with significant sensitivity to LIMK inhibition.

## RESULTS

### LIMK inhibition affects microtubule organization and mitosis

**Figure 1:**
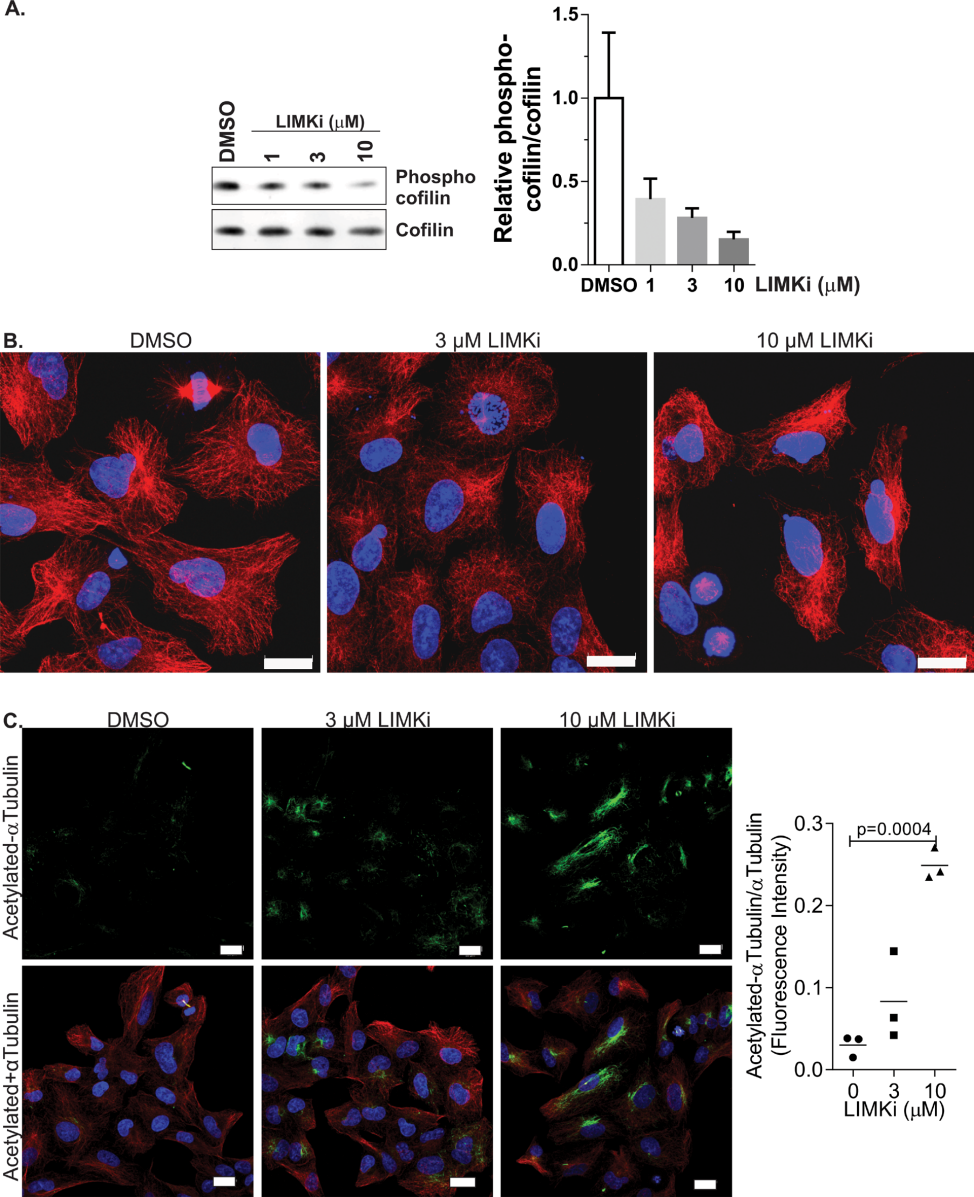
LIMK inhibition affects microtubule structures and acetylation **A.** A549 non-small cell lung adenocarcinoma cells were treated with LIMKi at the indicated concentrations for 24 hours, then cell lysates were Western blotted for phosphorylated and total cofilin. Graph indicates mean + SEM (*n* = 3). **B.** A549 non-small cell lung adenocarcinoma cells were treated as indicated for 24 hours, then fixed and stained with αTubulin antibody. Scale bar = 20 μm. **C.** A549 cells were treated as indicated for 24 hours, then fixed and stained with αTubulin (red) and acetylated αTubulin (green) antibodies. Nuclear DNA was stained with DAPI (blue). Scale bar = 20 μm. Immunofluorescence staining intensity of acetylated αTubulin was quantified with ImageJ software using a fixed intensity threshold, and normalized to total αTubulin immunofluorescence intensity levels. Statistical significance was analyzed by one-way ANOVA and Dunnett's *post-hoc* test (mean + SEM, *n* = 3).

To determine how LIMK inhibition affects cancer cell proliferation, we made use of the small molecular inhibitor N-{5-[2-(2,6-Dichloro-phenyl)-5-difluoromethyl-2H-pyrazol-3-yl]-thiazol-2-yl}-isobutyramide (compound 3 in [21]; hereafter referred to as LIMKi) that blocks both LIMK1 (7 nM IC_50_ *in vitro*) and LIMK2 (8 nM IC_50_) activity. The results of selectivity profiling of LIMKi at 10 μM against 287 kinases [21] using KINOME*scan* technology [31] was mapped onto a kinome phylogenetic tree [32] in Supplemental Figure 1A. To compare specificity quantitatively, the LIMKi S(35) selectivity score (a ratio of kinases inhibited by > 65% relative to the total number of kinases) was compared to S(35) values for 38 additional kinase inhibitors, including 7 FDA licenced drugs, at 10 μM (Supplemental Figure 1B; LIMKi indicated in blue). Furthermore, the inset graph in Supplemental Figure 1B of LIMKi S(1) (*i.e.* proportion of kinases inhibited by 99%), S(10) (proportion of kinases inhibited by 90%) and S(35) selectivity scores indicates the high selectivity of LIMKi. At 10 μM LIMKi, only 13 kinase targets (ADCK3, ALK4, AMPKα1, AMPKα2, BRSK1, BRSK2, DCAMKL1, DCAMKL2, DDR1, FGFR1, PAK3, PCTAIRE1) in addition to LIMK1 and LIMK2 were inhibited by > 65% [21]. We validated the dose-dependent effect of LIMKi on inhibiting LIMK activity by treating A549 human lung adenocarcinoma epithelial cells for 18 hours with DMSO vehicle or 1, 3 or 10 μM LIMKi [9, 21] and western blotting for phosphorylation of cofilin, a well-characterized LIMK substrate [9] (Figure 1A). We next examined how microtubule organization was affected by LIMKi in non-dividing cells by treating A549 cells for 24 hours with DMSO vehicle or 3 or 10 μM LIMKi. Representative images show progressive changes in microtubule morphology with increasing LIMKi dose (Figure 1B). To determine whether this effect was associated with changes in microtubule stability, we analysed the effect of LIMKi on αTubulin acetylation [33]. Confocal images of A549 cells co-stained with antibodies against acetylated-αTubulin (Figure 1C; green) and total αTubulin (Figure 1C; red) revealed a concentration-dependent increase in αTubulin acetylation after 24-hour LIMKi treatment. Quantification of fluorescence intensities revealed a moderate increase in αTubulin acetylation in response to 3 μM LIMKi, and a significant increase in response to 10 μM LIMKi treatment, relative to DMSO vehicle control. These results indicate that the LIMK inhibitor affected microtubule organization and post-translational modification.

**Figure 2:**
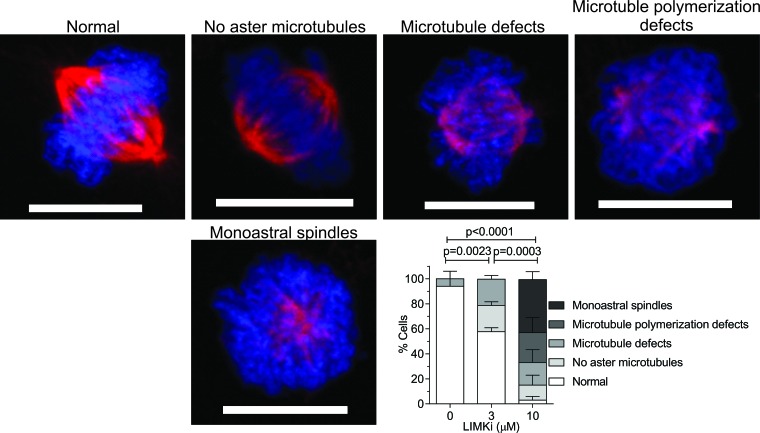
LIMK inhibition affects microtubule assembly in mitotic spindles A549 cells were treated as indicated for 24 hours, then fixed and stained for DNA with DAPI (blue) and αTubulin (red) antibody. Representative images of each type of mitotic defect observed are presented. For each condition, > 10 randomly selected metaphase cells were scored per indicated treatment. Scale bar = 10 μm. Statistical significance of changes in normal mitosis incidence were analyzed by one-way ANOVA and Tukey's multiple comparison *post-hoc* test (mean + SEM, *n* = 3).

To investigate the role of LIMK in mitosis, we analyzed the effect of LIMKi on mitotic spindle morphology. A549 cells were treated for 24 hours with DMSO vehicle, 3 or 10 μM LIMKi, then fixed and stained with αTubulin antibody and the DNA stain 4′,6-diamidino-2-phenylindole (DAPI). We observed significant alterations in spindle microtubule structure and organization with increasing LIMKi concentrations, including; decreased or complete loss of aster microtubules, defects in spindle microtubule integrity, defects in microtubule polymerization, or the appearance of monoastral spindles (Figure 2). To quantify these effects, > 10 representative mitotic cells per treatment were morphologically characterised for the above abnormalities and the percentage occurrence of each microtubule defect in three independent replicate experiments was determined (Figure 2). The occurrence of microtubule defects during mitosis progressively increased with increasing LIMKi concentration, with significant decreases in the percentage of normal cells with increasing LIMKi concentration (Figure 2). Therefore, we concluded that treatment with a LIMK inhibitory compound had a strong effect on cancer cell mitosis.

**Figure 3:**
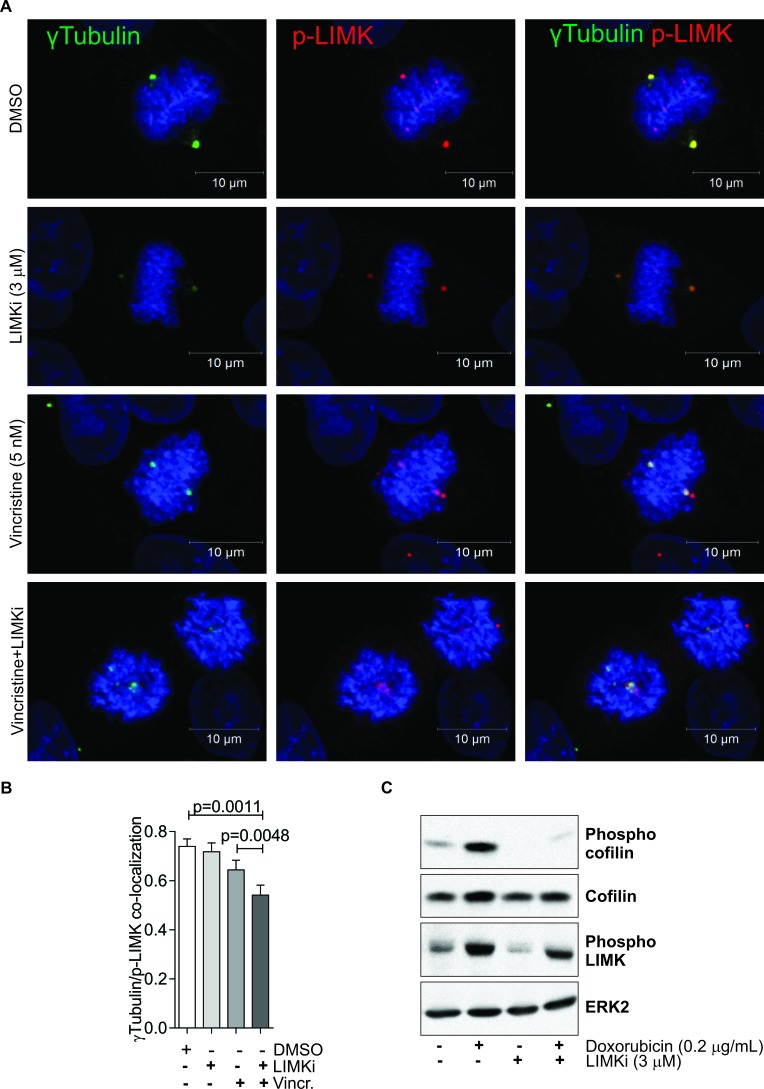
LIMK inhibition disrupts mitotic spindle integrity **A.** Co-localization of γTubulin (green) and phosphorylated LIMK (p-LIMK; red) was determined in A549 cells 24 hours after indicated treatments. Nuclear DNA was stained with DAPI (blue). **B.** Co-localization was analyzed on 10 randomly selected metaphase cells per indicated treatment. Pearson correlations of protein co-localization were quantified for each analyzed nucleus. Statistical significance of changes in protein co-localization were analyzed by one-way ANOVA and Tukey's multiple comparison *post-hoc* test (mean + SEM, *n* = 3). **C.** MCF-7 cells were treated with Adr (0.2 μg/ml) in the presence or absence of LIMKi (3 μM) for 24 h. Whole cell lysates were immunoblotted with antibodies against phospho-cofilin, cofilin, and phospho-LIMK. Equivalent protein loading was confirmed by ERK2 immunoblotting.

Consistent with the sensitivity of A549 cells to LIMKi, active phosphorylated LIM kinases have been previously found to co-localise with γTubulin at mitotic cell centrosomes [34]. To test if LIMK activity was important for active LIMK localization and mitotic spindle assembly, we tested the effect of LIMKi on active phosphorylated LIMK (p-LIMK) and γTubulin co-localization in mitotic A549 cells (Figure 3A). Treatment with 3 μM LIMKi had no effect on p-LIMK localization, indicating that p-LIMK localization to centrosomes is independent from LIMK activity (Figure 3B). Consistent with previous results [12], 3 μM LIMKi eliminated basal and induced cofilin phosphorylation, indicating inhibition of LIMK activity, while only slightly reducing LIMK phosphorylation (Figure 3C). Treatment with a low concentration of the vinca alkaloid Vincristine (5 nM), which did not detectably affect spindle morphology [35] had a small effect on p-LIMK and γTubulin co-localization (Figure 3B). The combination of 5 nM Vincristine and 3 μM LIMKi had a significant effect on p-LIMK and γTubulin co-localization greater than DMSO vehicle or Vincristine alone (Figure 3B). Moreover, immunofluorescence images show that combined LIMKi and vincristine treatment also had the strongest effect on spindle integrity, indicating possible co-operation between microtubules and LIMK function in mitosis (Figure 3A).

### Cancer cell proliferation is inhibited by LIMKi combined with microtubule polymerization inhibitors

**Figure 4:**
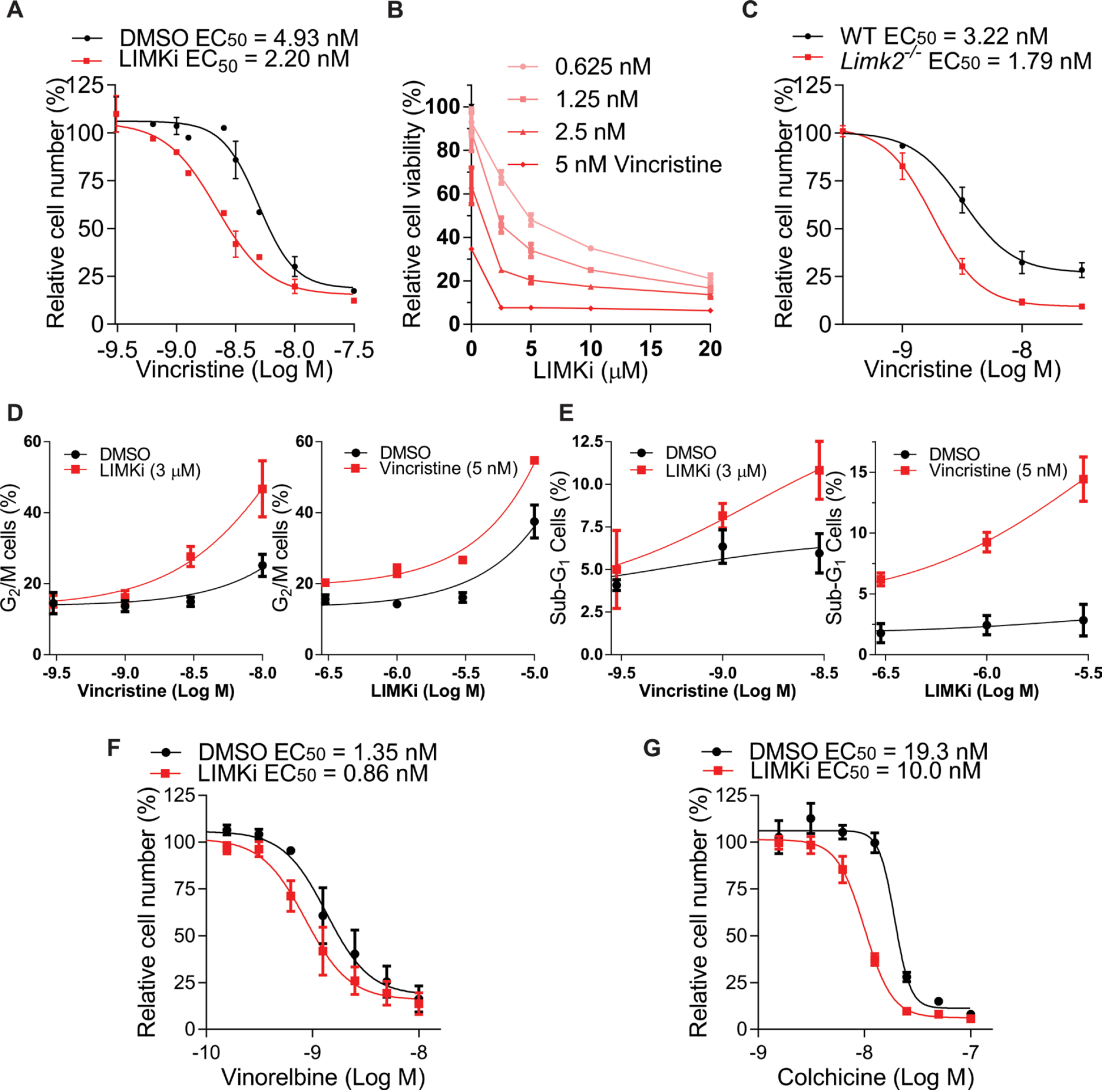
LIMK inhibition combines with microtubule polymerization inhibition **A.** A549 cells were treated with indicated concentrations of Vincristine in the presence of 3 μM LIMKi or DMSO for 72 hours, then cells were fixed with 4% paraformaldehyde and stained with DAPI. Nuclei were imaged and quantified relative to the number of cells treated with DMSO alone. Curve fitting was used to determine EC_50_ values (mean + SEM, *n* = 3). **B.** A549 cells were treated with combinations of two-fold serial dilutions of LIMKi and Vincristine, and relative cell viability determined by CellTiter-Glo^®^ (mean + SEM, *n* = 3). **C.** Spontaneously immortalized wild-type (WT) and *Limk2*^−/−^ mouse embryo fibroblasts (MEFs) were treated for 72 hours as indicated, and cell numbers relative to untreated controls used to determine EC_50_ values (mean + SEM, *n* = 3). **D.** The G_2_/M cell cycle profiles were determined by FACS of PI stained cells following 72 hours treatments as indicated (mean + SEM, *n* = 3). **E.** The sub-G_1_ DNA content profiles were determined by FACS of PI stained cells following 72 hour treatments as indicated (mean + SEM, *n* = 3). **F.** A549 cells were treated with indicated concentrations of Vinorelbine or **G.** Colchicine in the presence of 3 μM LIMKi or DMSO for 72 hours, then cells were fixed with 4% paraformaldehyde and stained with DAPI. Nuclei were imaged and quantified relative to the number of cells treated with DMSO alone. Curve fitting was used to determine EC_50_ values (mean + SEM, *n* = 3).

The effect of LIMKi combined with Vincristine on mitotic spindle integrity led us to test if LIMKi would have a combination effect on cancer cell proliferation. While 3 μM LIMKi alone did not affect A549 cell numbers, when treated with a concentration range of Vincristine in the presence of 3 μM LIMKi or DMSO in a 3-day assay based on counting remaining cell number, there was a ~2 fold decrease in Vincristine EC_50_ in the presence of LIMKi, indicating a synergistic cooperation between Vincristine and LIMKi (Figure 4A). By comparing the effects of fixed ratios of Vincristine (0.625-5 nM) and LIMKi (2.5-20 μM) concentrations on cell viability as determined by CellTiter-Glo^®^ Luminescent Cell Viability Assay, an average Combination Index of 0.77 + 0.02 indicating synergy was determined by the Chou-Talalay method [36] for LIMKi concentrations < 10 μM (Figure 4B). Furthermore, there was ~2-fold difference in Vincristine EC_50_ between spontaneously immortalized wild-type (WT) and *Limk2^−/−^* mouse embryo fibroblasts as determined by counting remaining cells (Figure 4C). To determine how the combined treatment of LIMKi and Vincristine decreased cell numbers, we profiled DNA content by fluorescence-activated cell sorting (FACS) of propidium iodide (PI) stained cells. The proportions of cells with 4N DNA content, indicative of G_2_/M cell cycle phases, were progressively increased with Vincristine concentration when combined with 3 μM LIMKi (Figure 4D, left panel) or with increased LIMKi concentration when combined with 5 nM Vincristine (Figure 4D, right panel) relative to DMSO vehicle control. In addition, the proportion of < 2N DNA content, typically a reflection of DNA fragmentation during apoptosis, progressively increased with increased Vincristine concentration when combined with 3 μM LIMKi (Figure 4E, left panel) or with increased LIMKi concentration when combined with 5 nM Vincristine (Figure 4E, right panel) relative to DMSO vehicle control. Taken together, these results indicate that the combined effect of LIMKi and a microtubule polymerization inhibitor was at least partially due to impaired mitosis, leading to impaired G_2_/M cell cycle progression and subsequent apoptosis.

To determine whether this effect was specific for Vincristine, we tested additional microtubule-targeting drugs with and without LIMKi. The microtubule polymerization inhibitors Vinorelbine (Figure 4F) and Colchicine (Figure 4G) had similar ~2-fold decreases in EC_50_ in the presence of LIMKi. These results indicate that LIMKi synergizes with agents that target microtubule polymerization.

### LIMKi potentiates kinase inhibitor reductions in proliferation

**Figure 5:**
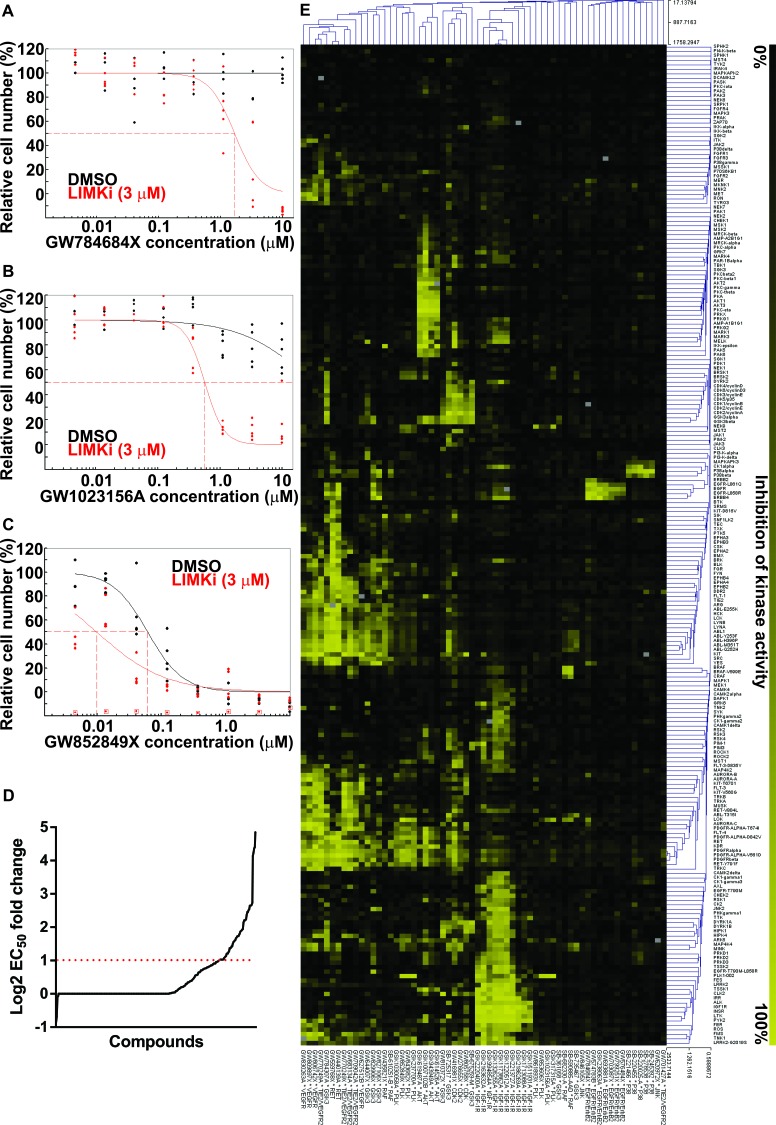
LIMK inhibition combination with Published Kinase Inhibitor Set compounds **A.** Cell numbers, relative to DMSO control, were used to determine EC_50_ values (indicated with dotted red lines) after 72 hours of treatment with DMSO or 3 μM LIMKi combined with GW784684X, **B.** GW1023156A or **C.** GW852849X as indicated. **D.** Log_2_ fold-change ratios of absolute EC_50_ values in the presence or absence of 3 μM LIMKi were plotted for each compound. Red dotted line indicates a 2-fold increase in compound sensitivity in the presence of 3 μM LIMKi. **E.** *In vitro* selectivity data of the selected 63 compounds against 224 kinase targets was used for unsupervised pairwise average-linkage cluster analysis with dot product distance option using Multiexperiment Viewer software. Percentage target inhibition is indicated in gradations from 0% (black) to 100% (yellow).

Given the consistent positive interactions between LIMKi and microtubule polymerization inhibitors, we wished to determine whether other cancer drug targets would also combine with LIMK inhibition to block cancer cell proliferation. A high-throughput screen was undertaken using the GSK published kinase inhibitor set (PKIS) of 366 small molecule kinase inhibitors [30]. A549 cells were treated with half-log serial dilutions of library compounds in the presence of 3 μM LIMKi or DMSO. Compounds having either no cytotoxic effect (Figure 5A), or an effect that did not reach 50% alone (Figure 5B), but achieved more than 50% reduction in relative cell number when combined with 3 μM LIMKi were selected as 20 hits (Supplemental Table 1). Compounds that produced a greater than 2-fold decrease in absolute EC_50_ when combined with 3 μM LIMKi (Figure 5C) were selected as 43 additional hits (Figure 5D; Supplemental Table 2). Remaining compounds either did not fit these criteria selection criteria (Supplemental Table 3) or were without combination effects (Supplemental Table 4).

To identify common drug targets from the selected hits, kinase inhibitor selectivity data for the 63 hits from GSK were clustered by pairwise average-linkage cluster analysis with the dot product distance option using TM4 [37] (Figure 5E), with color gradations ranging from 0% (black) to 100% inhibition (yellow). Amongst the most frequently occurring designated targets were VEGFR or VEGFR/TIE2, GSK3, AKT, CDK2 and IGF1R. However, the broad specificity of these inhibitors makes it difficult to draw conclusions about the contributions of individual kinases to the observed combination effects. In contrast, there were some clusters of kinase inhibitors with narrow selectivity profiles that highlight certain kinase targets as possibly indicating biological interactions with LIMK signaling, including PLK1, RAF, EGFR/ErbB2 and p38. These results indicate that there may be mechanism-based effective drug combinations with LIMK inhibitors, in addition to microtubule polymerization inhibitors, that could be characterized further for potential clinical use.

### Discovery of novel LIMK inhibitors

**Figure 6:**
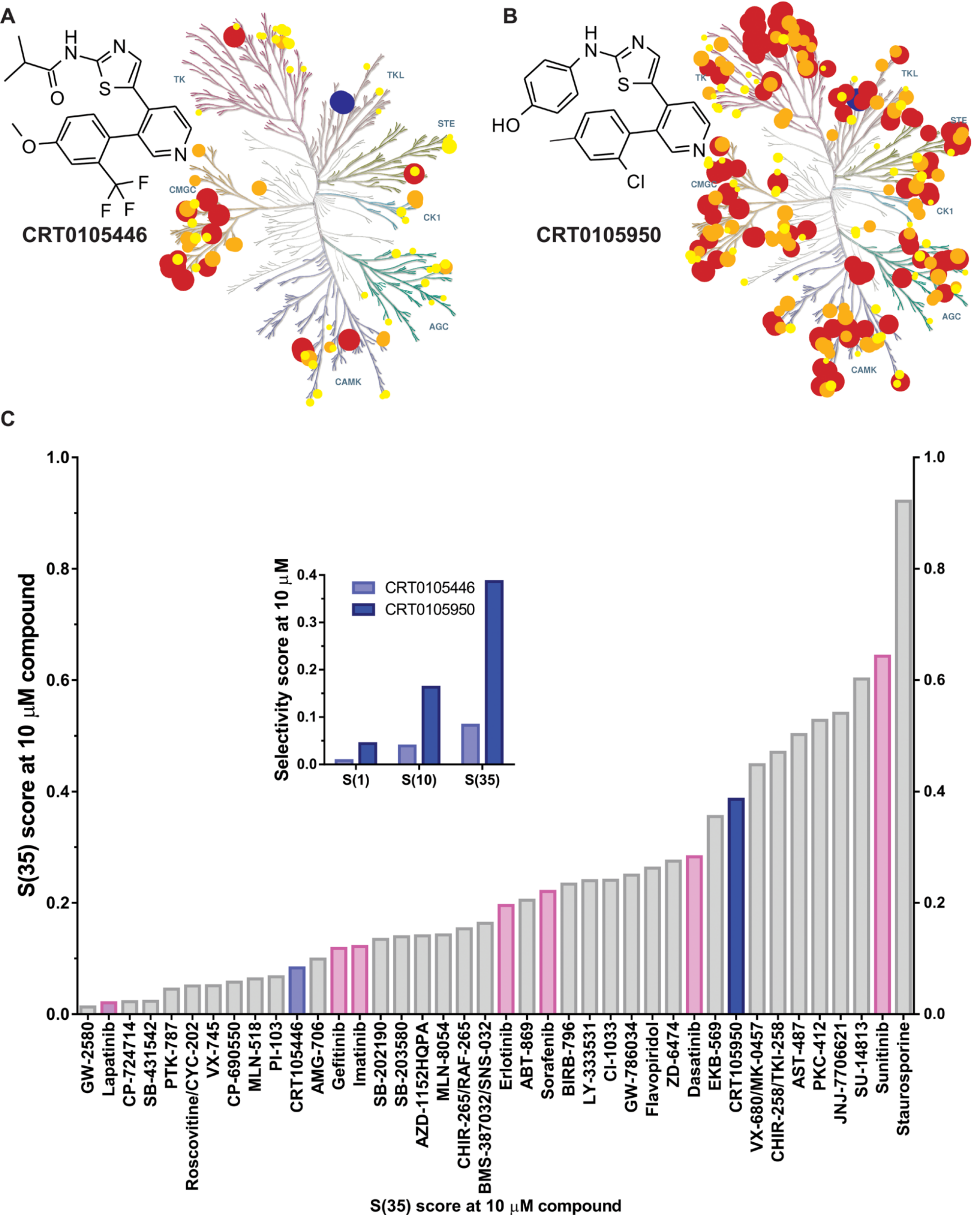
Discovery of novel LIMK inhibitors CRT0105446 and CRT0105950 **A.** CRT0105446 structure and percentage inhibition of 442 kinases at 10 μM mapped onto a kinome phylogenetic tree. Kinases inhibited >25% to 50% have yellow circles, 51% to 75% have orange circles, and >76% have red circles. In each case, the size of the circle is proportional to percentage inhibition. LIMK1 and LIMK2 positions are indicated in blue. Illustration reproduced courtesy of Cell Signaling Technology, Inc. (www.cellsignal.com). **B.** CRT0105950 structure and kinome selectivity mapping as described above. **C.** Selectivity S(35) scores were compared for CRT0105446 and CRT0105950 (blue) with 38 inhibitors, including 7 FDA-licenced compounds (pink). Inset graph indicates S(1), S(10) and S(35) scores for CRT0105446 and CRT0105950.

Given the potential for the use of LIMK inhibitors as cancer chemotherapeutics, a high-throughput drug discovery and medicinal chemistry program was undertaken. Details of the screening methodology and description of initial hit matter were described in [38]. Following iterative rounds of lead optimization medicinal chemistry and *in vitro* enzyme assays, two compounds were developed with potent LIMK inhibitory activity; *N*-{5-[3-(4-Methoxy-2-trifluoromethyl-phenyl)-pyridin-4-yl]-hiazol-2-yl}-isobutyramide (CRT0105446; Figure 6A) and 4-{5-[3-(2-Chloro-4-methyl-phenyl)-pyridin-4-yl]-thiazol-2-ylamino}-phenol (CRT0105950; Figure 6B). Details of their chemical synthesis are described in Charles *et al.* [39]. The *in vitro* IC_50_ values for LIMK1 and LIMK2 were 8 nM and 32 nM respectively for CRT0105446, and 0.3 nM and 1 nM respectively for CRT0105950. These and additional physiochemical parameters are in Supplemental Table 5. Kinase selectivity was profiled for 442 kinases using KINOME*scan* technology [31] at 10 μM CRT0105446 and CRT0105950 (Supplemental Table 6), and mapped onto a kinome phylogenetic tree [32] in Figure 6A and 6B. To compare specificity quantitatively, CRT0105446 and CRT0105950 S(35) selectivity scores (Figure 6C; indicated in blue), a ratio of kinases inhibited by >65% relative to the total number of kinases, were compared to S(35) values for 38 additional kinase inhibitors, including 7 FDA licenced drugs, at 10 μM (Figure 6C). Furthermore, the inset graph in Figure 6C depicting S(1) (*i.e.* proportion of kinases inhibited by 99%), S(10) (proportion of kinases inhibited by 90%) and S(35) selectivity scores depicts the greater selectivity of CRT0105446 relative to the more potent CRT0105950.

**Figure 7:**
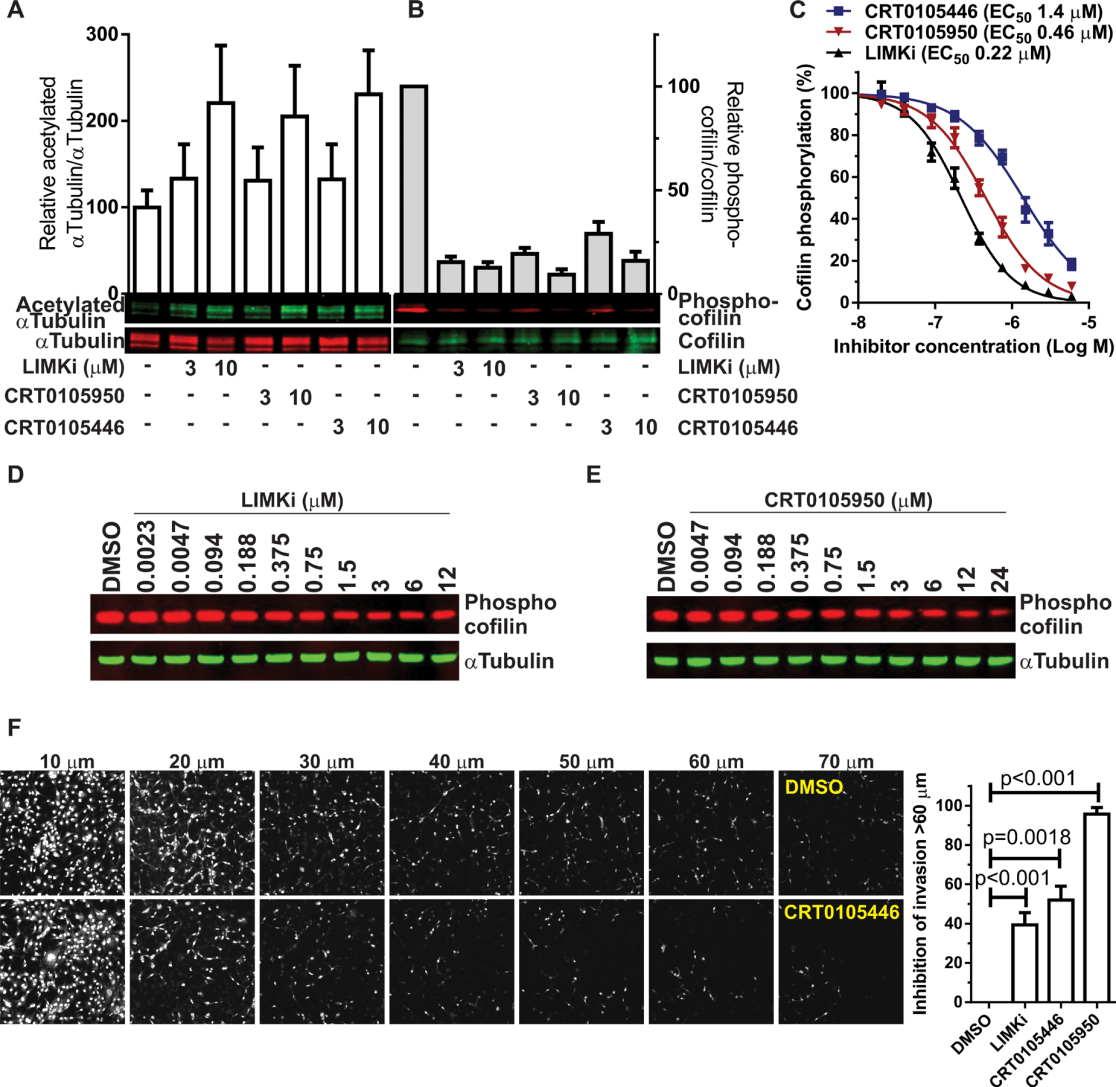
Comparison of CRT0105446 and CRT0105950 effects on LIMK functions in cells **A.** A549 cells were treated with indicated concentrations of LIMK inhibitors for 24 hours, then blotted for acetylated αTubulin (green) and αTubulin (red). Ratios of acetylated/total αTubulin were determined and plotted relative to vehicle treated cells (mean + SEM, *n* = 3). **B.** A549 cells were treated as above, then blotted for phosphorylated cofilin (red) and total cofilin (green). Ratios of phosphorylated/total cofilin were determined and plotted relative to vehicle treated cells (mean + SEM, *n* = 3). **C.** MCF7 breast cancer cells were treated with 2 μg/mL Doxorubicin plus DMSO or indicated concentrations of LIMK inhibitors for 18 hours, then cells were fixed and stained for phosphorylated cofilin. Single cell fluorescence intensities were determined and curve fitted to determine EC_50_ values (mean + SEM, *n* = 8). **D.** MDAMB231 breast cancer cells were treated with indicated concentrations of LIMKi or **E.** CRT0105950 for 24 hours, then cell lysates prepared and immunoblotted with antibodies against phospho-cofilin and αTubulin. **F.** Confocal images taken at 10 μm intervals through 3-D matrigel revealed significant inhibition of MDAMB231 breast cancer cell invasion above 60 μm by 3 μM LIMKi, CRT0105446 and CRT0105950 relative to DMSO control. Statistical significance was analyzed by one-way ANOVA and Dunnett's *post-hoc* test (mean + SEM, *N* = 3-8).

We compared the cell-based potencies of CRT0105446 and CRT0105950 with LIMKi using three read-outs: cofilin phosphorylation, αTubulin acetylation and three-dimensional (3-D) extracellular matrix invasion. All three compounds were equivalently potent at increasing αTubulin acetylation (Figure 7A) and decreasing cofilin phosphorylation (Figure 7B) in A549 lung cancer cells. To more precisely determine cell-based LIMK inhibitor activity, MCF7 breast cancer cells were treated with 2 μg/mL doxorubicin, which strongly increases cofilin phosphorylation through increased p53-mediated transcription of *LIMK2* and *RhoC* genes [12] to increase the dynamic range and signal-to-noise ratio of the assay, and varying concentrations of LIMK inhibitors for 18 hr, then cells were fixed and stained for immunofluorescence determination of phosphorylated cofilin intensity. Dose-response analysis revealed that all three compounds inhibited cofilin phosphorylation, with LIMKi being ~2-fold more potent than CRT0105950 and 7-fold more potent than CRT0105446 in this assay format (Figure 7C). We additionally established that LIMK inhibition resulted in decreased cofilin phosphorylation in MDAMB231 human breast cancer cells by comparing the effect of a range of LIMKi and CRT0105950 concentrations on phospho-cofilin levels. As shown in Figure 7D and 7E, both inhibitors induced dose-dependent decreases in cofilin phosphorylation. Finally, the inverse invasion of 3-D matrigel by MDAMB231 breast cancer cells above a 60 μm cut-off was significantly inhibited by 3 μM LIMKi, CRT0105446 and CRT0105950 (Figure 7F), consistent with our previous observations using LIMKi [9]. These results are consistent with all three inhibitors being potent inhibitors of LIMK activity in cells.

### Identification of cancer cells sensitive to LIMK inhibitors

**Figure 8:**
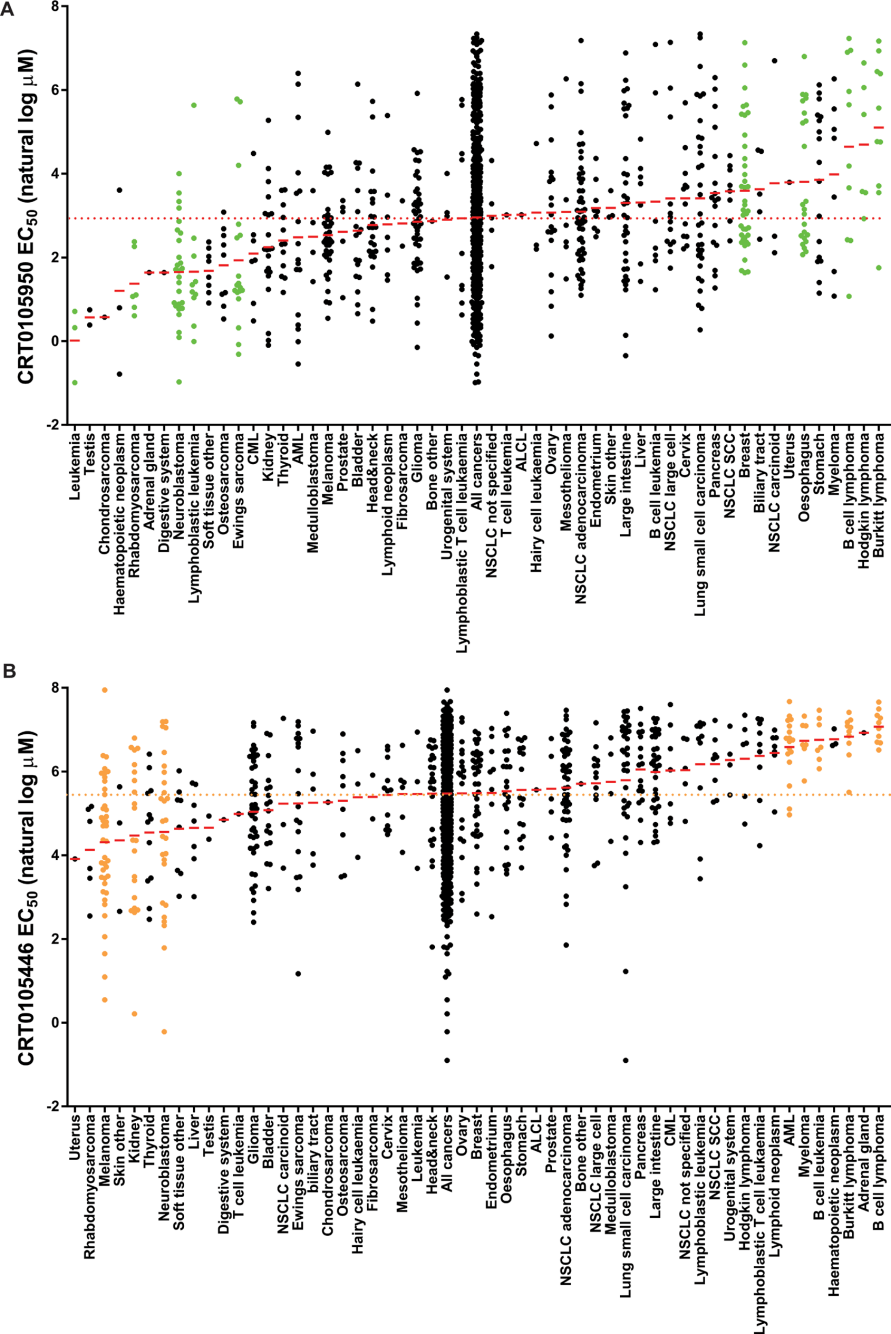
Evaluation of cancer cell line sensitivities to LIMK inhibitors **A.** A total of 656 cancer cell lines, divided into 54 cancer types, were treated with 9 concentrations of a 2-fold dilution series of CRT0105950 (top concentration = 10 μM). After 72 hours, cell density was measured, EC_50_ values calculated and MANOVA performed to identify cancer types significantly (*p* < 0.05; green dots) sensitive or resistant to CRT109590 relative to all cancers considered together. Red lines indicate mean EC_50_ for each cancer type, and red dotted line showing mean EC_50_ for all cancers. **B.** Sensitivity or resistance to CRT0105446 was determined as described above. Cancer types significantly (*p* < 0.05) different from all cancers indicated with orange dots.

To identify cancer cell types with significant sensitivity to chemical LIMK inhibitors, a screen was performed on 656 cancer cell lines [40] with CRT0105950 and CRT0105446. Each inhibitor was tested at concentrations from 0.3-10 μM, and cell numbers determined as described in [40]. By comparing calculated EC_50_ values for cell lines (Supplemental Tables 7-8) within a given cancer type against all cancer cell lines (Supplemental Table 9-10), significantly (p < 0.05) sensitive and resistant cancer types were identified. Figure 8A shows natural log EC_50_ values (in μM) of CRT0105950 for each cell line within each cancer type, with a red line indicating mean EC_50_ and arranged in order of their mean EC_50_ from low to high values. Relative to the mean EC_50_ of CRT0105950 for all cancer cell lines (19.2 μM), there were 5 cancer types that were significantly sensitive and 5 cancer types that were significantly resistant (shown with green dots). Similarly, 3 cancer types were significantly sensitive and 5 were significantly resistant (Figure 8B, orange dots), relative to the mean EC_50_ of all cancer cells to CRT0105446 (238 μM).

**Figure 9:**
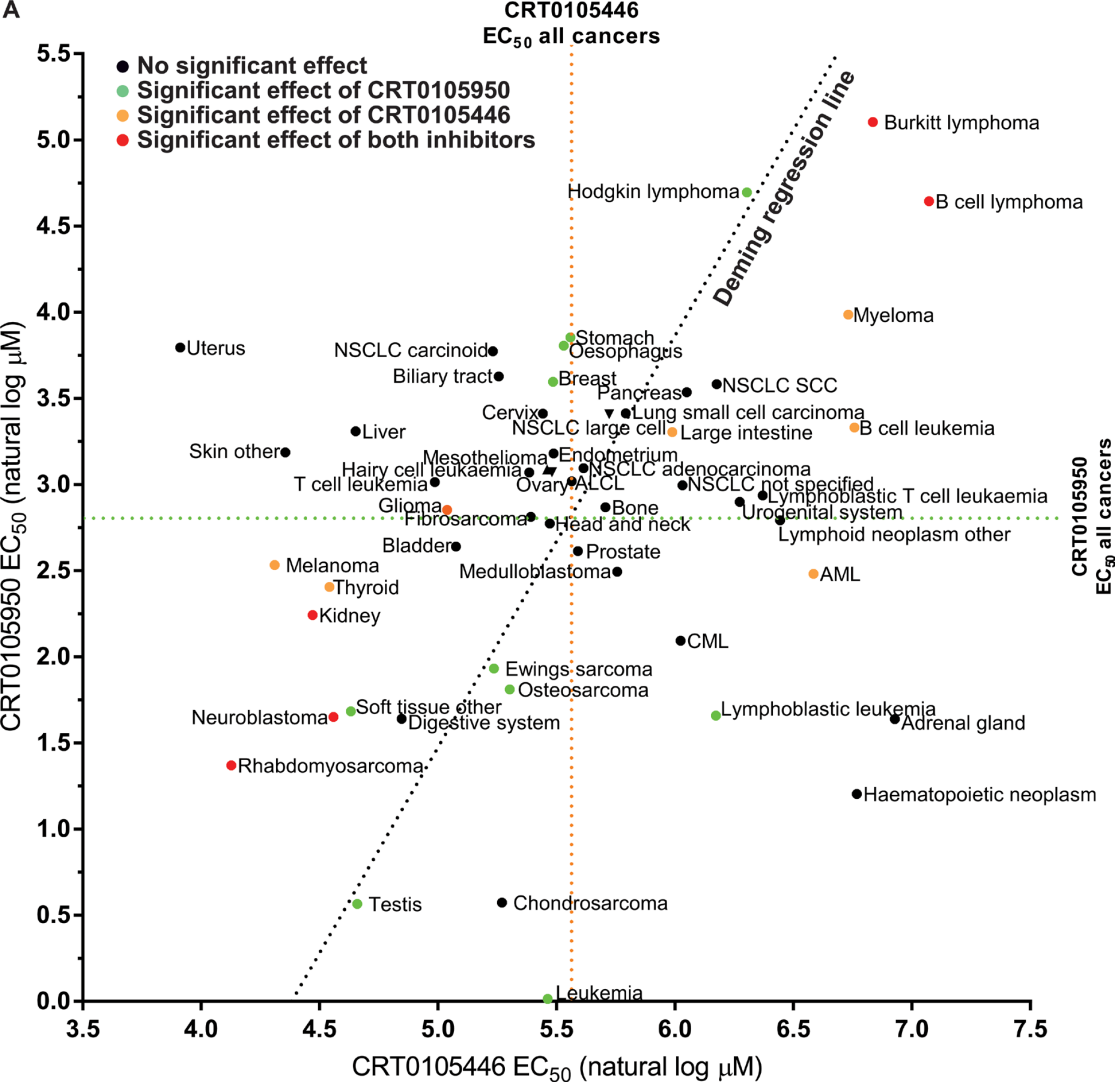
Comparison of cancer cell line sensitivities to LIMK inhibitors The mean EC_50_ values for each cancer type to CRT0105950 and CRT0105446 were plotted, with mean EC_50_ for all cancers indicated as a dotted green line for CRT0105950 and an orange dotted line for CRT0105446. Red dots indicate cancer types significantly (*p* < 0.05) resistant or sensitive to both inhibitors, while green dots indicate sensitivity or resistance to CRT0105950 alone and orange dots indicate sensitivity or resistance to only CRT0105446. Deming regression indicates that there is a significant (*p* = 0.0293) relationship between the sensitivities to both drugs.

To determine which cancer types were most likely affected by the on-target effects of CRT0105446 and CRT0105950, we plotted the mean EC_50_ values for both drugs of each cancer type (Figure 9). Cancer types that were significantly sensitive or resistant to both drugs are indicated with red dots, while those cancer types significantly affected by CRT0105446 are depicted with orange dots and those significantly affected by CRT0105950 are shown with green dots. Deming regression indicates that the slope of a fitted line was significantly different from 0, indicating that there was a direct relationship between cancer type responses to both drugs. This analysis revealed that neuroblastoma, rhabdomyosarcoma and kidney cancer cells were significantly sensitive to both LIMK inhibitors, with 6 additional cancer types being sensitive to either inhibitor and trending towards sensitivity to the other (*e.g.* Ewing's sarcoma, osteosarcoma and additional soft tissue cancer cell lines). Burkitt lymphoma and B cell lymphoma were the only significantly resistant cancer types, with 4 additional cancer types being sensitive to either inhibitor and trending towards sensitivity towards the other (*e.g.* Hodgkin lymphoma and myeloma).

**Figure 10:**
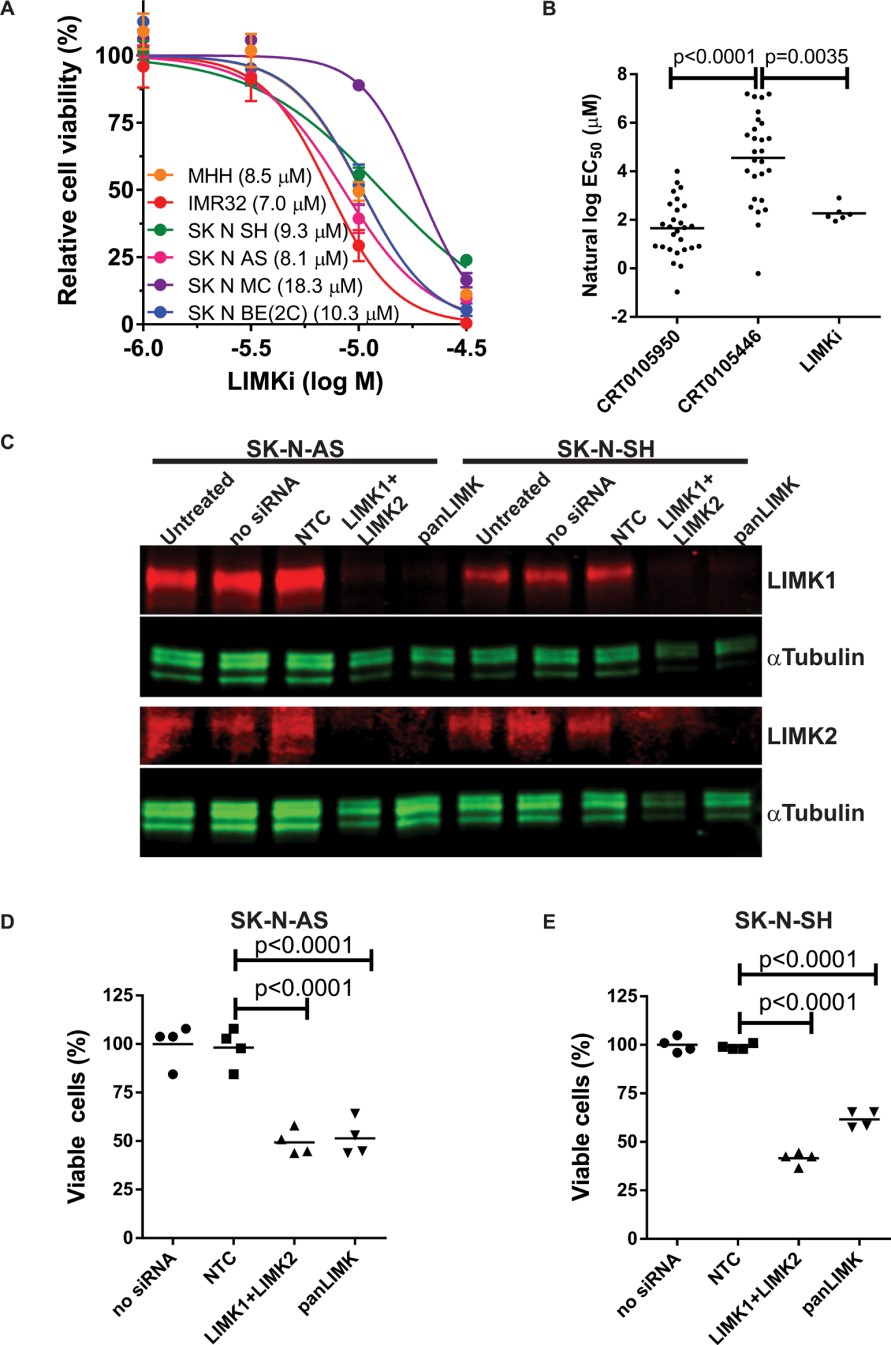
Sensitivity of neuroblastoma cell lines to LIMK inhibition **A.** Cell viability relative to DMSO vehicle control was determined by CellTiter-Glo^®^ after treatment of neuroblastoma cell lines with indicated concentrations of LIMKi. Figure legend shows the calculated EC_50_ for each cell line (mean + SEM, *n* = 3). **B.** Comparison of the sensitivities of 27 neuroblastoma cell lines to CRT0105950 and CRT0105446 with the sensitivity of 6 neuroblastoma cell lines to LIMKi. Line indicates mean EC_50_ for each LIMK inhibitor. Significance was analyzed by one-way ANOVA and *post-hoc* Tukey's test. **C.** SK-N-AS and SK-N-SH neuroblastoma cell lines were left untreated or transfected with non-targeting (NTC) siRNA, a combination of LIMK1 and LIMK2 siRNAs (LIMK1+LIMK2) or a dual LIMK1/LIMK2 targeting siRNA as indicated, then 72 hours later cell lysates were prepared and immunoblotted for LIMK1 and LIMK2. αTubulin blotting allowed for comparison of protein loading. **D.** Relative cell viability was determined for SK-N-AS and **E.** SK-N-SH cells 72 hours after siRNA transfection by CellTiter-Glo^®^ viability assay and normalized to untransfected cells in each experimental replicate. Significance was analyzed by one-way ANOVA and post-hoc Tukey's test.

To validate the sensitivity of neuroblastoma cells to LIMK inhibitors, we tested 6 neuroblastoma cell lines for their responses to LIMKi (Figure 10A). When the mean natural log EC_50_ values of LIMKi for these 6 cell lines were compared to CRT0105950 and CRT0105446 EC_50_ values for the 27 neuroblastoma cell lines from the screen, both LIMKi and CRT0105950 were significantly more effective than CRT0105446 but were not different from each other (Figure 10B), consistent with their rank order of potencies for inhibition of cofilin phosphorylation, tubulin acetylation and matrix invasion (Figure 7). To corroborate the requirement for LIMK activity in neuroblastoma cells, we knocked down LIMK1 and LIMK2, either by combining two siRNA oligonucleotides (LIMK1+LIMK2) or using an siRNA oligonucleotide (panLIMK) that reduces expression of both proteins, in SK-N-AS and SK-N-SH cells (Figure 10C). Relative to a non-targeting control (NTC) oligonucleotide, both forms of LIMK knockdown significantly reduced neuroblastoma cell viability in SK-N-AS cells (Figure 10D) and SK-N-SH cells (Figure 10E).

## DISCUSSION

The LIM kinases have well characterized roles in regulating the actin cytoskeleton through their regulation of cofilin activity, and consequently have been regarded as potential targets for anti-metastasis therapy [1, 15, 41]. It has recently emerged that LIMK1 and LIMK2 also influence microtubule dynamics and have important functions in mitosis [4, 5, 13], which is consistent with LIMK inhibitors being potential cancer therapeutics. In the present study, the effect of inhibiting LIMK activity on microtubule structures and mitosis has been validated, and combinations with microtubule destabilizing drugs shown to act synergistically. By screening the PKIS compound library, additional potential drug combinations with LIMK inhibition were identified, including EGFR and Raf kinases. Previous research on the chemosensitivity of 39 human cancer cell lines to 55 anti-cancer drugs revealed that elevated LIMK2 expression was significantly correlated to resistance to 18 drugs (including topoisomerase I inhibitors, bleomycin derivatives and anthracyclines such as doxorubicin) independent of the tissue of origin [10]. We also showed that DNA damaging agents induce *LIMK2* expression via p53, while inhibition or knockdown of LIMK2 activity increased sensitivity to these chemotherapeutics [12]. Taken together, these findings are consistent with LIMK activity being an important contributory factor to drug resistance.

To identify cancer types sensitive to LIMK inhibition, 656 cancer cell lines were screened with dose ranges of CRT0105446 and CRT0105950 that enabled statistical comparison of drug sensitivity. Three cancer types were significantly sensitive to both LIMK inhibitors; rhabdomyosarcoma, neuroblastoma and kidney cancer. Interestingly, previous research showed that neuroblastoma cells selected for resistance to Vincristine had increased LIMK2 expression, while LIMK2 knockdown led to the formation of abnormal mitotic spindles and sensitized neuroblastoma cells to Vincristine and Vinblastine [13]. Furthermore, LIMK2 knockdown increased cell cycle arrest apoptosis induced by microtubule targeting drugs [11]. These results are consistent with a prominent role for LIMK signalling in neuroblastoma.

The two novel LIMK inhibitors described in the present study, CRT0105446 and CRT0105950, are potent inhibitors of LIMK1 and LIMK2 that will enable further development of LIMK-targeted cancer therapy.

## MATERIALS AND METHODS

### Cell line culture and validation

A549 non-small cell lung adenocarcinoma, MDAMB231 and MCF7 breast cancer and neuroblastoma cell lines (MHH-NB-11, LAN-6, SH-SY5Y, SK-N-BE-2c, SK-N-AS, SK-N-SH, SK-N-MC, IMR-32) were cultured according to ATCC guidelines. Cell identities were validated using the GenePrint 10 system STR multiplex assay (Promega) that amplifies 9 tetranucleotide repeat loci and Amelogenin gender determining marker.

### Antibodies

αTubulin (Sigma, Cell Signaling Technology), acetylated-αTubulin (Novus Biologicals), pLIMK (Thr508 - LIMK1, Thr505 - LIMK2; Abcam or Cell Signaling Technology), γTubulin (Sigma), phospho-cofilin (Ser3; Cell Signaling Technology), cofilin (Abcam) and ERK2 (Ab122; CJ Marshall, Institute of Cancer Research, London).

### Western blotting

Cells were lysed with 500 μl of lysis buffer (1x Tris-buffered saline (TBS), 1% Triton-X, 1 nM EDTA, 0.2 mM Na3VO4, 20 mM NaF, 1 mM PMSF, and cOmplete Mini protease inhibitor cocktail (Roche)) per 10 cm plate. Lysates were cleared by 10-minute centrifugation and Western blotted [12] with corresponding antibodies. Quantification of Western blots was performed directly without signal amplification or X-ray film using infra-red emitting secondary antibodies and detection with an ODYSSEY® infrared imaging system (LI-COR).

### Immunofluorescence

Cells were plated on glass coverslips, 24 hours after treatment, cells were fixed and antibody stained as described [9]. Images were acquired on a Zeiss710 laser-scanning confocal microscope and processed in ZEN2010 (Zeiss).

### Immunofluorescence image quantification

Immunofluorescence intensity of acetylated-αTubulin was quantified in ImageJ using fixed intensity threshold, and normalized to total αTubulin. Co-localization analysis was performed on ten mitotic cell images per treatment per experiment using Volocity (PerkinElmer). Pearson correlation of protein co-localization was quantified for each cell analysed.

To quantify mitotic cell morphological changes, pictures of > 10 randomly-selected cells in metaphase were taken per treatment per experiment and scored based on their morphology as indicated. Percentages of cells with each defect were quantified for each treatment condition and reported as mean + SEM from triplicate determinations.

### Cytotoxicity assay

A549 cells were plated in 96-well plates at 2000 cells per well, 24 hours before treatment. Cells were treated with serial dilutions of indicated compounds alone or in the presence of 3 μM LIMKi or DMSO, for 72 hours. Surviving cells were fixed with 4% paraformaldehyde and stained with 250 ng/ml DAPI. Experiments were repeated with three independent replicate experiments. Nuclei were imaged on a High Content Imaging Operetta system (PerkinElmer) and quantified using Harmony^®^ High Content Imaging and Analysis Software (PerkinElmer). Cell numbers were plotted as percent change from DMSO-treated control and EC_50_ values were calculated from dose-response curves using Prism 5 (GraphPad). Drug combination synergy was determined by treating cells with serial dilutions of LIMKi (2.5-20 μM) and Vincristine (0.625-5 nM) alone or in 4×4 combinations, and quantifying cells 72 hours using CellTiter-Glo^®^ Luminescent Cell Viability Assay (Promega), following manufacturer's protocol. Combination index and effect parameters were determined with CalcuSyn [36]. Neuroblastoma cell viability was quantified using CellTiter-Glo^®^ Luminescent Cell Viability Assay (Promega), following manufacturer's protocol.

### Sub-G_1_ and G_2_/M quantification

Cells were plated in 6 well plates at 5×10^5^ cells/well and treated the next day with indicated drugs for 72 hours. Percentage cells with sub-G_1_ DNA content or in G_2_/M phase were measured and analyzed as described [12].

### PKIS compound screen

Cells were plated at 350 cells per well in 384-well plates, in 5 technical replicates. 24 hours after plating, cells were treated with serial dilutions of indicated compounds (ranging from 0.005 to 10 μM in half-log dilutions) alone or in the presence of 3 μM LIMKi or DMSO, for 72 hours. Vincristine was used as a positive control and DMSO (final 0.4%) was used as a negative control. Cells were fixed, stained with DAPI, and nuclei of surviving cells quantified as in the cytotoxicity assay. Results were plotted as percent change in cell number relative to the median DMSO alone-treated cell number and absolute EC_50_ values were calculated for each compound in the presence of LIMKi or DMSO control using the Vortex software analysis program.

### Synthesis of CRT0105446 and CRT0105950

Discovery and synthesis of CRT0105446 and CRT0105950 as described in Charles *et al.* [39].

### Kinase selectivity profiling

Selectivity profiling was undertaken by Ambit (now DiscoveRx) as described in [42].

### Cofilin phosphorylation quantification

MCF7 human breast cancer cells were plated at 5×10^3^ cells/well in black polystyrene glass-bottomed 96 well plates and treated the next day with 0.2 μg/mL doxorubicin plus indicated LIMK inhibitor concentrations for 18 hours. Cells were washed, fixed with 4% paraformaldehyde, permeabilized with 0.5% Triton X-100 (v/v) and blocked for 1 hour with 1% BSA (w/v). Fixed cells were stained with rabbit antibody against phospho-Ser3 cofilin, then Alexa 488 anti-rabbit antibody (Invitrogen), Texas red Phalloidin (Molecular Probes) and DAPI (Sigma). After washing, cells were imaged on a High Content Imaging Operetta system (PerkinElmer) and phospho-Ser3 cofilin fluorescence intensity for each cell quantified using Harmony^®^ High Content Imaging and Analysis Software (PerkinElmer), and plotted as percent change from DMSO alone-treated control for eight independent replicate determinations. EC_50_ values were calculated from dose-response curves using Prism 5 (GraphPad).

### Inverse invasion assay

MDAMB231 human breast cancer cell inverse invasion was assayed as described in [9].

### Cancer cell line screen

Cancer cell line screening as described [40, 43].

### siRNA knock-down

SK-N-AS or SK-N-SH cells were transiently transfected in suspension with indicated siRNA oligonucleotides at 25 nmoles per well, using DreamFect^TM^ Gold transfection reagent (OZ Biosciences) according to manufacturer's instructions, and plated at 500 cells per well in 96-well plates and incubated for 72 hours for determination of cell viability by CellTiter-Glo^®^ Luminescent Cell Viability Assay (Promega) according to manufacturer's instructions. LIMK1 and LIMK2 ON-TARGETplus siRNA SMARTpools (Thermo Scientific); pan-LIMK siRNA, sense strand sequence: 5′-AGGCTATCAAGGTGACACA-3′; NTC (non-targeting control) siRNA - (Thermo Scientific). For knock-down validation, 4,000 transfected cells from each assay were plated in 24-well plates and lysed after 72 hours. LIMK1 and LIMK2 protein levels were determined by Western blotting, with αTubulin blotting allowing for comparison of protein loading.

### Statistical analysis

All other statistical analyses were performed with Prism5 (GraphPad).

## SUPPLEMENTARY MATERIAL FIGURE AND TABLES






















