# Supplementary material for: LIM kinase inhibitors disrupt mitotic microtubule organization and impair tumor cell proliferation
Source: Oncotarget. 2015 Nov 3;6(36):38469–86. doi: 10.18632/oncotarget.6288 (PMC4770715; doi:10.18632/oncotarget.6288)
Supplement: Supplementary file 1 [file oncotarget-06-38469-s001.pdf]

LIM kinase inhibitors disrupt mitotic microtubule organization and impair tumor cell proliferation

Supplementary Material

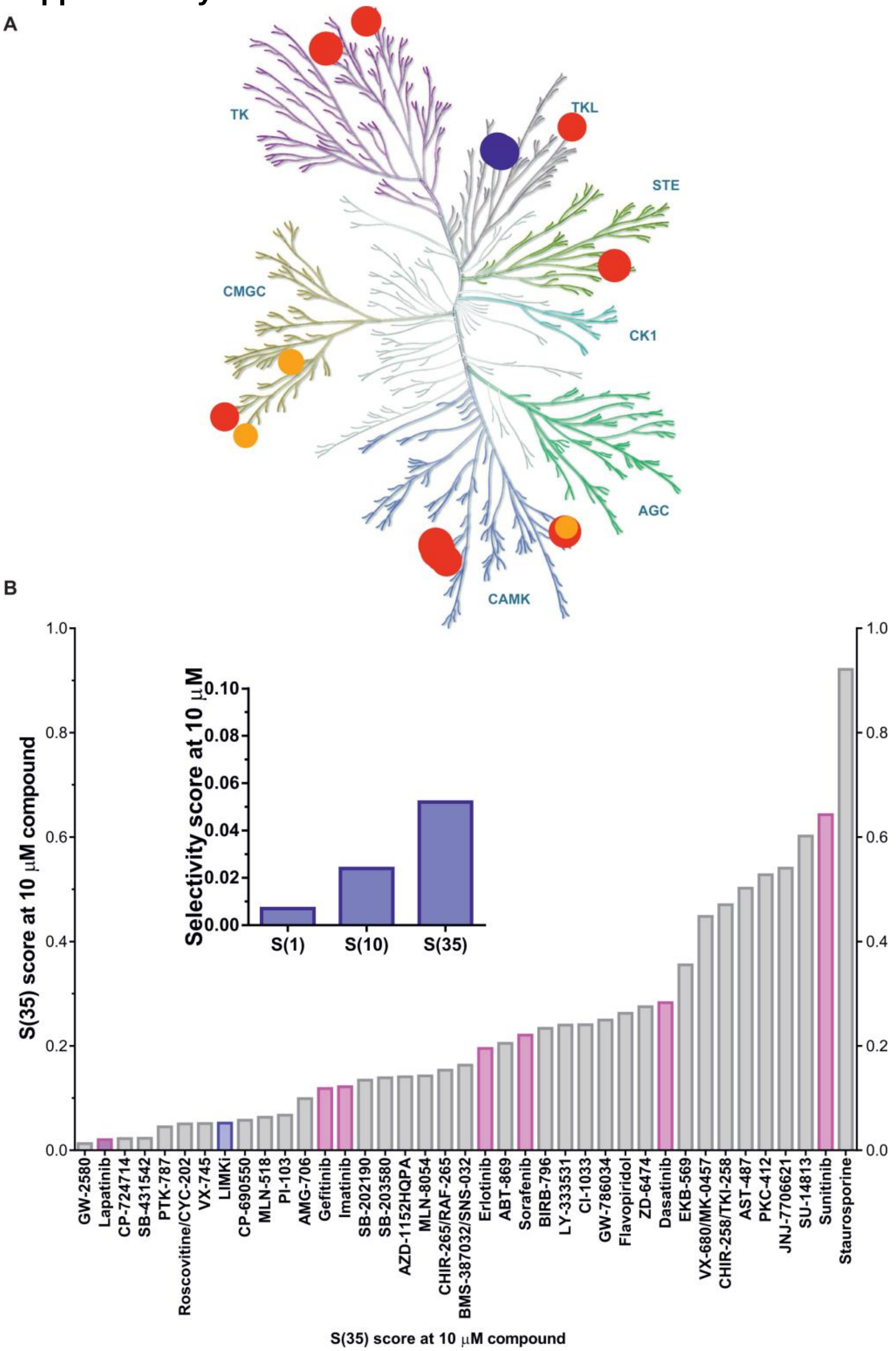

Supplemental Figure 1. Selectivity profiling of LIMKi.

A. LIMKi percentage inhibition of 287 kinases at 10 μM mapped onto a kinome

phylogenetic tree. Kinases inhibited by 51% to 75% have orange circles and >76% have red circles. In each case, the size of the circle is proportional to percentage inhibition. LIMK1 and LIMK2 positions are indicated in blue. Illustration reproduced courtesy of Cell Signaling Technology, Inc. (www.cellsignal.com). **B.** Selectivity S(35) score for LIMKi (blue) with 38 inhibitors, including 7 FDA-licenced compounds (pink). Inset graph indicates S(1), S(10) and S(35) scores LIMKi.

Supplemental Table 1: Combination screen with PKIS inhibitors

Supplemental Table 2: Combination screen with PKIS inhibitors

Supplemental Table 3: Combination screen with PKIS inhibitors

Supplemental Table 4: Combination screen with PKIS inhibitors

Supplemental Table 5: Physicochemical properties of CRT0105446 and CRT0105950

Supplemental Table 6: Selectivity data for CRT0105446 and CRT0105950

Supplemental Table 7: Cell line sensitivity for CRT0105446

Supplemental Table 8: Cell line sensitivity for CRT0105950

Supplemental Table 9: Cancer type data for CRT0105446

Supplemental Table 10: Cancer type data for CRT0105950
