# Supplementary material for: LIM kinase inhibitors disrupt mitotic microtubule organization and impair tumor cell proliferation
Source: Oncotarget. 2015 Nov 3;6(36):38469–86. doi: 10.18632/oncotarget.6288 (PMC4770715; doi:10.18632/oncotarget.6288)
Supplement: Supplementary file 2 [file oncotarget-06-38469-s002.pdf]

Supplier Ref	Formatted ID	pXC50 Mo	REL pXC50	ABS XC50	Min	Max	Hillslope	R2	Condition	Graph	XC50 M	REL pXC50	ABS XC50	Min	Max	Hillslope	R2	Condition	Graph
GW644007X	BDP-00006356	=	5.4629	3.444348	0	100	-19.211	0.7391	+DMSO	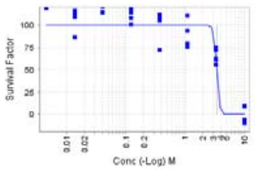	=	5.7372	1.831313	0	100	-1.7504	0.841	+LIMK	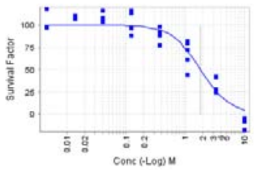
SB-361058	BDP-00006189	<	5	<5	0	100	-11.359	-0.1176	+DMSO	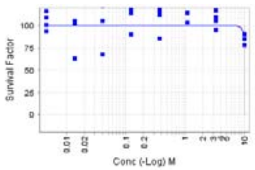	=	5.0565	8.780325	0	100	-21.883	0.7905	+LIMK	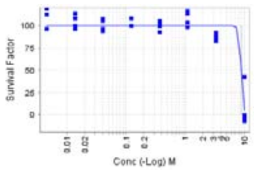
SB-735467	BDP-00006207	<	5	<5	0	100	-506.3	-0.0717	+DMSO	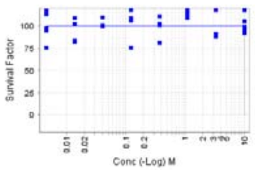	=	5.0589	8.730894	0	100	-1.9054	0.622	+LIMK	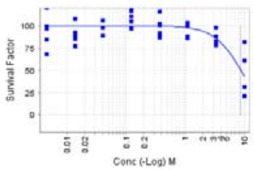
GSK317315A	BDP-00006258	=	7.0217	0.095129	0	100	-1.9964	0.772	+DMSO	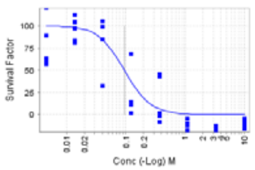	>	8.3372	>8.3372	0	100	-0.5818	0.4853	+LIMK	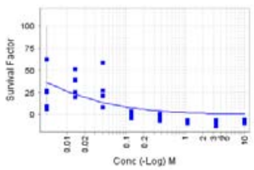
GSK1220512A	BDP-00006280	=	7.9618	0.01092	0	100	-1.5915	0.7838	+DMSO	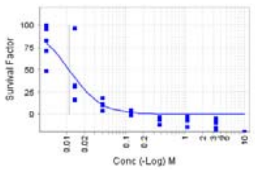	>	8.3372	>8.3372	0	100	-1.0279	0.6488	+LIMK	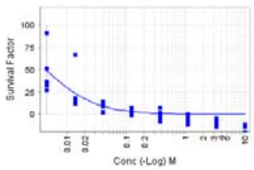
GW830263A	BDP-00006298	<	5	<5	0	100	-0.7309	0.0298	+DMSO	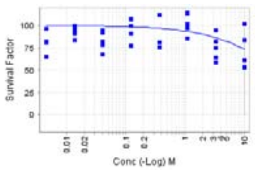	=	5.25	5.623925	0	100	-1.9496	0.7642	+LIMK	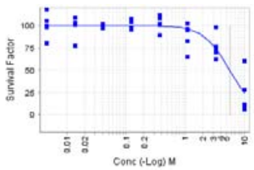
GW701427A	BDP-00006313	<	5	<5	0	100	-1.3694	0.0953	+DMSO	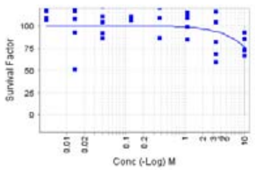	=	5.4174	3.825115	0	100	-1.9217	0.7433	+LIMK	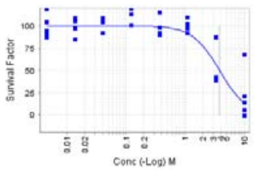

GSK1023156A	BDP-00006315	<	5	<5	0	100	-0.7882	0.2991	+DMSO	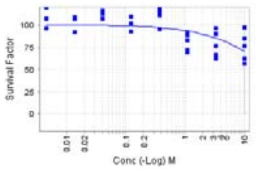	=	6.2458	0.567816	0	100	-2.8356	0.898	+LIMK	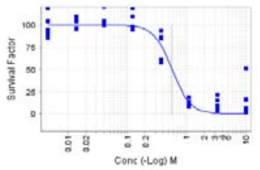
GSK1030058A	BDP-00006316	<	5	<5	0	100	-11.562	0.0106	+DMSO	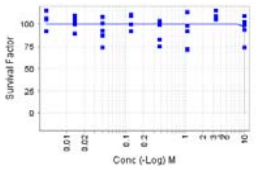	=	5.0912	8.10592	0	100	-1.7247	0.5011	+LIMK	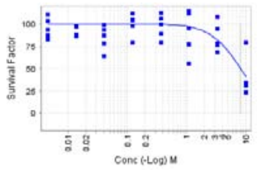
GW853606X	BDP-00006323	<	5	<5	0	100	-0.6023	0.134	+DMSO	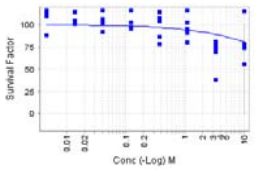	=	5.7373	1.83123	0	100	-1.332	0.7739	+LIMK	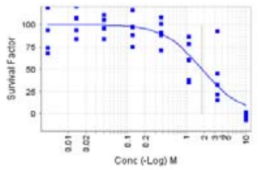
GSK619487A	BDP-00006332	<	5	<5	0	100	-0.2794	0.1411	+DMSO	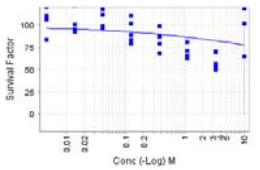	=	5.8051	1.566427	0	100	-0.9437	0.8773	+LIMK	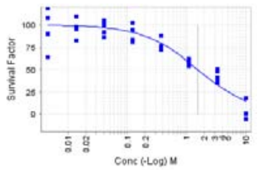
GW827106X	BDP-00006376	>	8.3372	>8.3372	0	100	2.0386	-0.6498	+DMSO	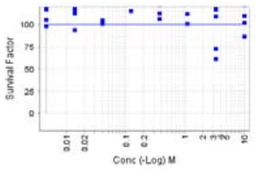	=	5.3654	4.311181	0	100	-1.9301	0.6222	+LIMK	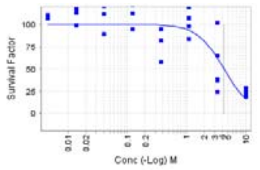
GW829906X	BDP-00006382	<	5	<5	0	100	-363	-0.234	+DMSO	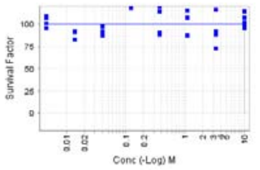	=	5.29	5.128767	0	100	-1.7586	0.56	+LIMK	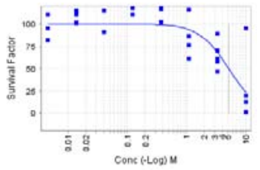
GW574783B	BDP-00006391	<	5	<5	0	100	-212.58	-0.4842	+DMSO	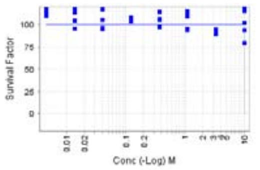	=	5.1013	7.919524	0	100	-2.0802	0.7543	+LIMK	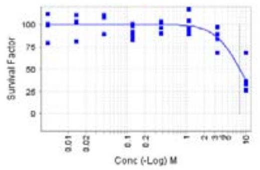

GW576484X	BDP-00006392	<	5	<5	0	100	-2.9854	-1.187	+DMSO	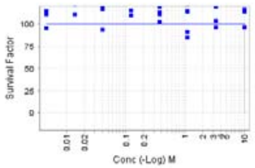	=	5.1961	6.366166	0	100	-3.1346	0.588	+LIMK	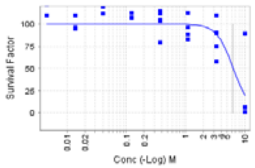
SB-610251-B	BDP-00006494	<	5	<5	0	100	-20.202	-0.8064	+DMSO	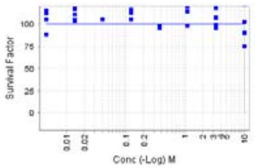	<	5	<5	0	100	-11.303	-0.5137	+LIMK	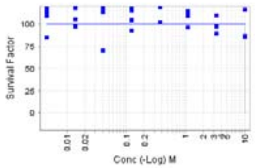
SB-590885-AAD	BDP-00006495	<	5	<5	0	100	-11.613	0.0083	+DMSO	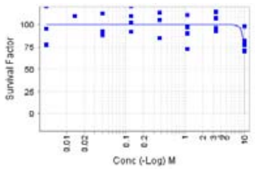	=	5.1028	7.892057	0	100	-3.2435	0.614	+LIMK	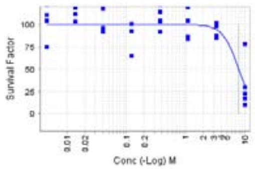
GSK238063A	BDP-00006501	<	5	<5	0	100	-1.4033	0.148	+DMSO	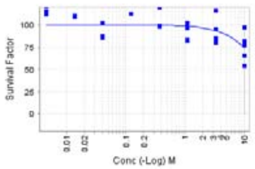	=	5.1677	6.797116	0	100	-2.1171	0.7066	+LIMK	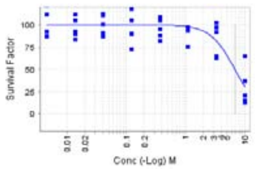
GW693881A	BDP-00006505	<	5	<5	0	100	-3.3962	0.216	+DMSO	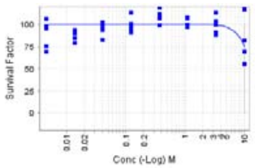	<	5	<5	0	100	-0.5056	0.076	+LIMK	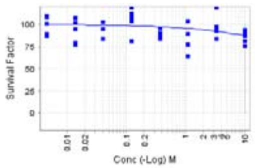
GW784684X	BDP-00006506	>	8.3372	>8.3372	0	100	2.0862	-0.1052	+DMSO	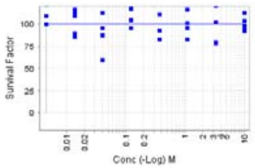	=	5.774	1.682542	0	100	-2.1521	0.8211	+LIMK	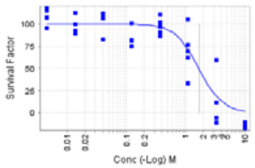
