# Supplementary material for: LIM kinase inhibitors disrupt mitotic microtubule organization and impair tumor cell proliferation
Source: Oncotarget. 2015 Nov 3;6(36):38469–86. doi: 10.18632/oncotarget.6288 (PMC4770715; doi:10.18632/oncotarget.6288)
Supplement: Supplementary file 3 [file oncotarget-06-38469-s003.pdf]

Supplier Ref	Formatted ID	pXC50 Mo	REL pXC50	ABS XC50	Min	Max	Hillslope	R2	onditi	Graph	XC50 Mo	REL pXC50	ABS XC50	Min	Max	Hillslope	R2	Conditior	Graph	Ratio ABS XC50
GW843682X	BDP-00006263	=	6.2472	0.565988	0	100	-0.3752	0.3648	DMSO	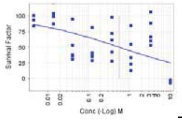	=	7.7073	0.019619	0	100	-0.9646	0.7251	+LIMK	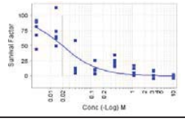	28.85
GW416981X	BDP-00006434	=	5.7552	1.757117	0	100	-0.3073	0.4725	DMSO	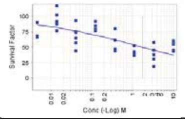	=	7.0794	0.083282	0	100	-0.651	0.7413	+LIMK	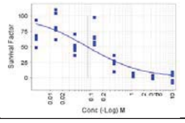	21.10
GW806742X	BDP-00006296	=	6.1352	0.732396	0	100	-0.5779	0.301	DMSO	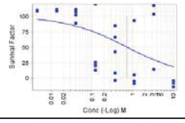	=	7.4123	0.038698	0	100	-15.027	0.9277	+LIMK	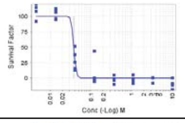	18.93
GW770249X	BDP-00006415	=	5.1367	7.299197	0	100	-1.8769	0.5163	DMSO	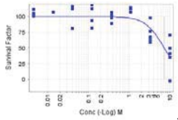	=	5.9594	1.097905	0	100	-39.474	0.9048	+LIMK	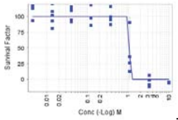	6.65
GSK2186269A	BDP-00006409	=	5.9369	1.156276	0	100	-2.6988	0.7262	DMSO	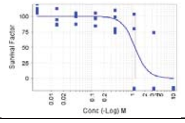	=	6.7332	0.18486	0	100	-3.8016	0.7923	+LIMK	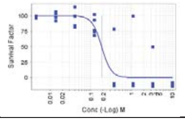	6.25
GSK2220400A	BDP-00006410	=	5.4755	3.346142	0	100	-13.032	0.7436	DMSO	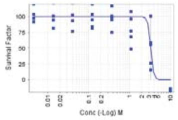	=	6.2703	0.536618	0	100	-1.6606	0.9121	+LIMK	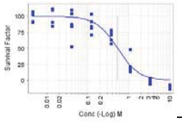	6.24
GW852849X	BDP-00006264	=	7.2138	0.061118	0	100	-1.5899	0.9031	DMSO	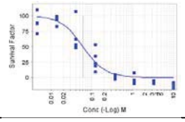	=	8.0084	0.009809	0	100	-0.815	0.6856	+LIMK	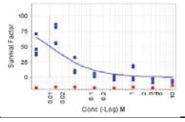	6.23

GW709042A	BDP-00006314	=	5.1935	6.404701	0	100	-1.5896	0.5466	DMSO	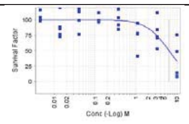	=	5.9593	1.098163	0	100	-1.3513	0.7283	+LIMK	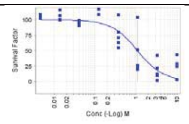	5.83
GW784307A	BDP-00006357	=	6.9057	0.124258	0	100	-1.2399	0.9161	DMSO	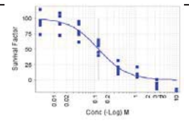	=	7.6438	0.022711	0	100	-0.8478	0.8728	+LIMK	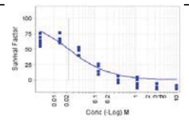	5.47
GSK1713088A	BDP-00006401	=	5.692	2.032518	0	100	-1.2022	0.6444	DMSO	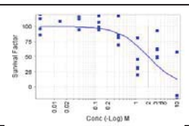	=	6.4061	0.392565	0	100	-19.732	0.6472	+LIMK	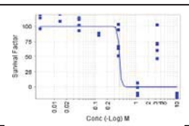	5.18
GSK1173862A	BDP-00006279	=	7.1808	0.065943	0	100	-1.9192	0.8785	DMSO	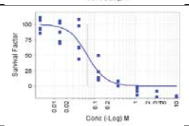	=	7.8509	0.014097	0	100	-1.3268	0.8748	+LIMK	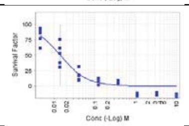	4.68
SB-682330-A	BDP-00006497	=	5.0279	9.377868	0	100	-17.14	0.7059	DMSO	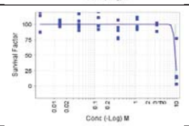	=	5.6968	2.009921	0	100	-5.0216	0.8409	+LIMK	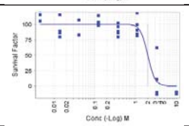	4.67
GW627512B	BDP-00006156	=	5.4033	3.95063	0	100	-0.9551	0.713	DMSO	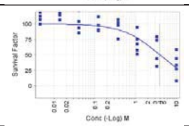	=	6.066	0.85909	0	100	-1.9432	0.7844	+LIMK	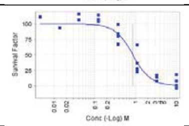	4.60
GSK2163632A	BDP-00006408	=	6.4805	0.330754	0	100	-1.8211	0.9069	DMSO	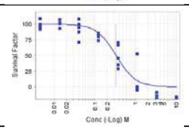	=	7.1427	0.071988	0	100	-1.7178	0.8943	+LIMK	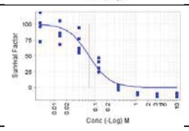	4.59

GW861893X	BDP-00006340	=	5.067	8.57137	0	100	-1.1712	0.5577	DMSO	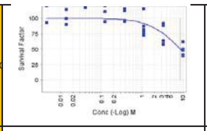	=	5.7106	1.947179	0	100	-0.9246	0.7349	+LIMK	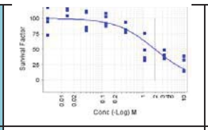	4.40
GSK1326255A	BDP-00006281	=	6.8726	0.134075	0	100	-2.1469	0.7922	DMSO	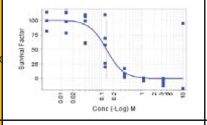	=	7.4562	0.034982	0	100	-2.0163	0.9219	+LIMK	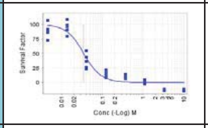	3.83
GW683134A	BDP-00006308	=	5.3143	4.849051	0	100	-2.344	0.6164	DMSO	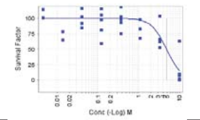	=	5.894	1.276305	0	100	-1.1308	0.7734	+LIMK	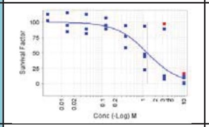	3.80
GSK237700A	BDP-00006255	=	5.2099	6.16683	0	100	-3.3912	0.5501	DMSO	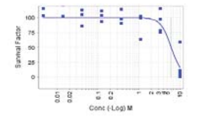	=	5.7806	1.657172	0	100	-2.9175	0.8332	+LIMK	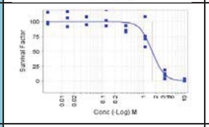	3.72
GW846105X	BDP-00006165	=	5.7456	1.796239	0	100	-2.2394	0.8773	DMSO	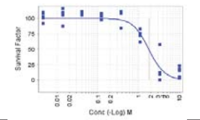	=	6.2904	0.512374	0	100	-1.7028	0.9153	+LIMK	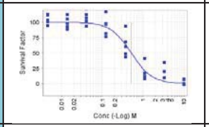	3.51
GW805758X	BDP-00006483	=	6.563	0.273535	0	100	-1.1584	0.7646	DMSO	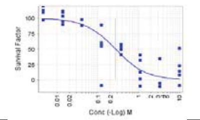	=	7.0997	0.079493	0	100	-4.3672	0.8697	+LIMK	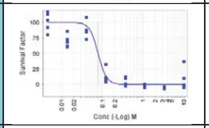	3.44
GW810372X	BDP-00006369	=	5.2691	5.382007	0	100	-5.9125	0.7885	DMSO	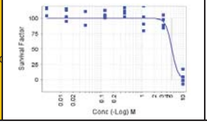	=	5.791	1.617915	0	100	-1.6052	0.797	+LIMK	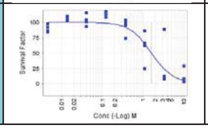	3.33

GSK994854A	BDP-00006283	=	7.388	0.040925	0	100	-1.2944	0.7637	DMSO	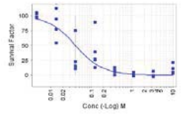	=	7.9094	0.012319	0	100	-1.5835	0.8745	+LIMK	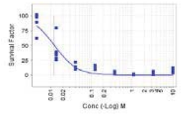	3.32
GW809897X	BDP-00006297	=	6.9163	0.121247	0	100	-2.713	0.7976	DMSO	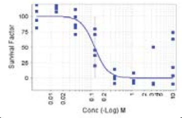	=	7.434	0.03681	0	100	-2.7629	0.8529	+LIMK	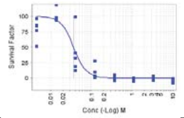	3.29
SB-221466	BDP-00006464	=	5.9009	1.256268	0	100	-2.8858	0.8665	DMSO	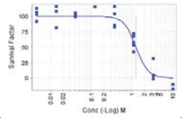	=	6.4026	0.395727	0	100	-3.1646	0.9105	+LIMK	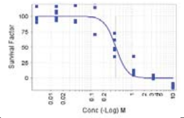	3.17
GW770249A	BDP-00006415	=	5.4394	3.63559	0	100	-20.305	0.7892	DMSO	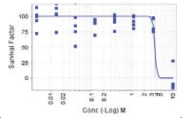	=	5.9265	1.184451	0	100	-1.7776	0.8345	+LIMK	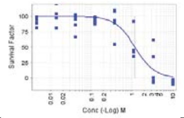	3.07
GW703087X	BDP-00006217	=	5.9301	1.174649	0	100	-2.8965	0.8198	DMSO	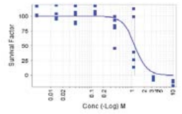	=	6.4127	0.38664	0	100	-2.4766	0.8657	+LIMK	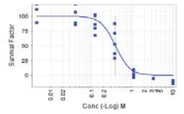	3.04
GW440139A	BDP-00006491	=	5.3915	4.059666	0	100	-0.9503	0.5036	DMSO	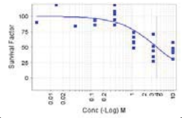	=	5.8614	1.376047	0	100	-1.6398	0.8686	+LIMK	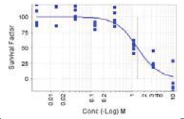	2.95
GSK2213727A	BDP-00006403	=	7.0844	0.08234	0	100	-2.161	0.5143	DMSO	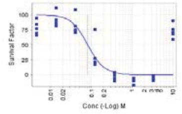	=	7.5152	0.030532	0	100	-1.3555	0.479	+LIMK	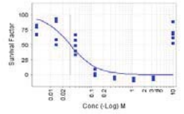	2.70

GSK1511931A	BDP-00006405	=	5.9137	1.219775	0	100	-1.154	0.793	DMSO	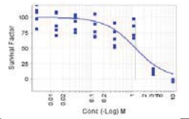	=	6.3265	0.471477	0	100	-0.9611	0.9013	+LIMK	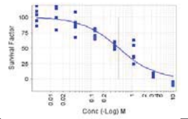	2.59
GW799251X	BDP-00006220	=	5.1311	7.393861	0	100	-1.7892	0.7666	DMSO	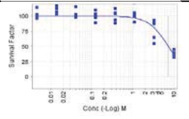	=	5.5434	2.861554	0	100	-2.2112	0.8591	+LIMK	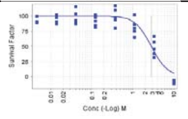	2.58
GW559768X	BDP-00006492	=	6.5411	0.287675	0	100	-0.5883	0.8204	DMSO	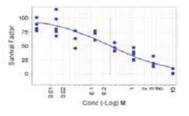	=	6.9267	0.11838	0	100	-0.3488	0.5874	+LIMK	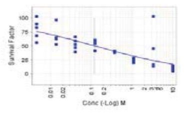	2.43
GW276655X	BDP-00006419	=	5.097	7.999329	0	100	-1.8578	0.4871	DMSO	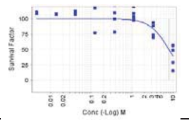	=	5.4621	3.450983	0	100	-2.3148	0.8264	+LIMK	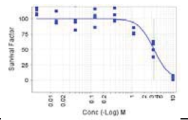	2.32
GW620972X	BDP-00006164	=	7.2923	0.051018	0	100	-2.6539	0.877	DMSO	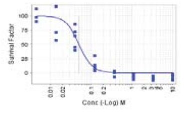	=	7.6572	0.022017	0	100	-3.1391	0.9301	+LIMK	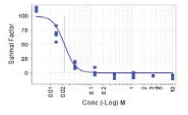	2.32
GSK614526A	BDP-00006331	=	5.5449	2.851595	0	100	-1.7034	0.8222	DMSO	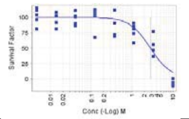	=	5.9033	1.24937	0	100	-0.9702	0.8541	+LIMK	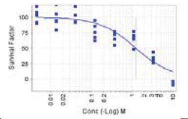	2.28
SB-220455	BDP-00006463	=	5.4589	3.476232	0	100	-17.032	0.6524	DMSO	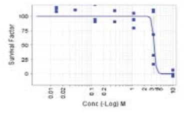	=	5.7982	1.591601	0	100	-4.0857	0.8806	+LIMK	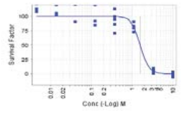	2.18

SB-725317	BDP-00006204	=	6.3472	0.44958	0	100	-1.163	0.9029	DMSO	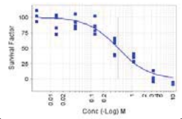	=	6.6855	0.206317	0	100	-1.4959	0.9222	+LIMK	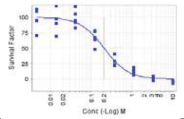	2.18
SB-278538	BDP-00006449	=	5.6311	2.338389	0	100	-1.6694	0.8175	DMSO	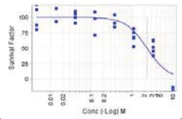	=	5.9586	1.10009	0	100	-39.297	0.9279	+LIMK	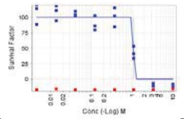	2.13
SB-220025-A	BDP-00006462	=	6.1383	0.727299	0	100	-1.1908	0.8876	DMSO	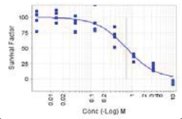	=	6.4503	0.354549	0	100	-1.0408	0.9086	+LIMK	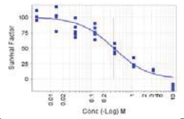	2.05
SB-675259-M	BDP-00006198	=	6.2186	0.60451	0	100	-1.8962	0.8411	DMSO	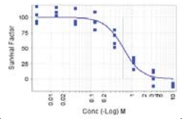	=	6.5291	0.295756	0	100	-1.1646	0.8991	+LIMK	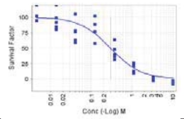	2.04
GSK943949A	BDP-00006225	=	5.5453	2.849103	0	100	-2.4602	0.8478	DMSO	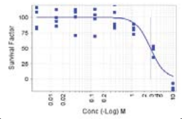	=	5.8556	1.394527	0	100	-1.2104	0.9045	+LIMK	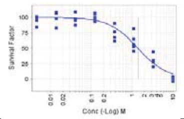	2.04
GSK1007102B	BDP-00006223	=	6.8736	0.133778	0	100	-1.4524	0.8856	DMSO	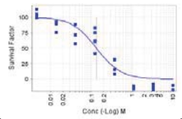	=	7.1837	0.065511	0	100	-1.6543	0.9522	+LIMK	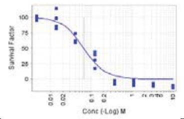	2.04
GW435821X	BDP-00006233	=	5.8192	1.516454	0	100	-2.1684	0.8975	DMSO	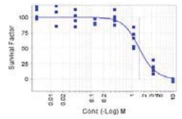	=	6.1285	0.743828	0	100	-1.7169	0.8829	+LIMK	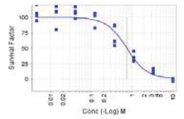	2.04

SB-245392	BDP-00006440	=	5.9306	1.173129	0	100	-19.726	0.8706	+DMSO	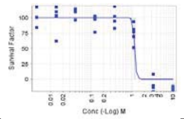	=	6.2364	0.58022	0	100	-5.0337	0.8974	+LIMK	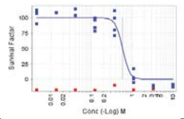	2.02
