# Supplementary material for: LIM kinase inhibitors disrupt mitotic microtubule organization and impair tumor cell proliferation
Source: Oncotarget. 2015 Nov 3;6(36):38469–86. doi: 10.18632/oncotarget.6288 (PMC4770715; doi:10.18632/oncotarget.6288)
Supplement: Supplementary file 4 [file oncotarget-06-38469-s004.pdf]

Supplier Ref	Formatted ID	pXC50 Mo	REL pXC50	ABS XC50	Min	Max	Hillslope	R2	onditi	Graph	XC50 M	REL pXC50	ABS XC50	Min	Max	Hillslope	R2	Conditior	Graph	Ratio ABS XC50
SB-251505	BDP-00006442	=	5.211	6.152424	0	100	-3.1165	0.7402	+DMSO	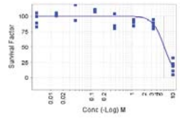	=	5.5098	3.091936	0	100	-1.5424	0.804	+LIMK	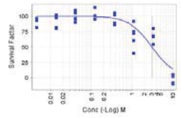	1.99
GSK312948A	BDP-00006321	=	5.7994	1.587156	0	100	-2.4335	0.7638	+DMSO	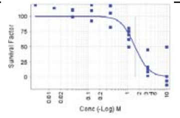	=	6.0924	0.808398	0	100	-1.8658	0.906	+LIMK	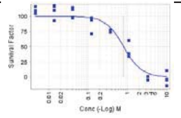	1.96
SB-732881	BDP-00006203	=	6.8763	0.132967	0	100	-0.5533	0.7247	+DMSO	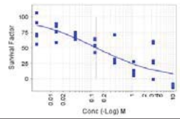	=	7.1634	0.068649	0	100	-0.7469	0.9115	+LIMK	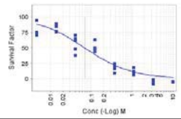	1.94
GW683003X	BDP-00006475	=	5.0031	9.92982	0	100	-15.658	0.428	+DMSO	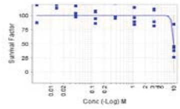	=	5.2777	5.275417	0	100	-3.4141	0.7762	+LIMK	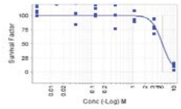	1.88
SB-236687	BDP-00006467	=	5.7303	1.860959	0	100	-1.8308	0.8663	+DMSO	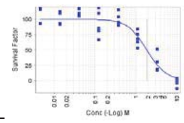	=	5.9978	1.005186	0	100	-1.8127	0.8898	+LIMK	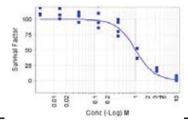	1.85
GSK1751853A	BDP-00006402	=	5.8313	1.474786	0	100	-1.046	0.7857	+DMSO	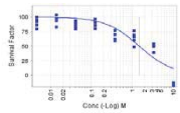	=	6.0981	0.797837	0	100	-1.3377	0.8803	+LIMK	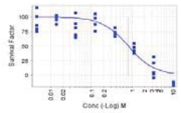	1.85
GW618013A	BDP-00006458	=	5.169	6.776777	0	100	-2.7229	0.6901	+DMSO	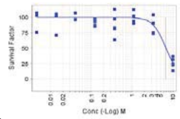	=	5.4274	3.737984	0	100	-19.426	0.9037	+LIMK	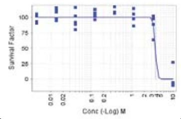	1.81

GW406731X	BDP-00006230	=	5.2449	5.689497	0	100	-1.965	0.7392	DMSO	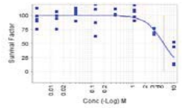	=	5.5014	3.151905	0	100	-3.2703	0.7821	+LIMK	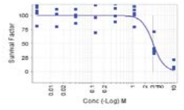	1.81
GW589933X	BDP-00006424	=	5.5017	3.149801	0	100	-1.2292	0.7986	DMSO	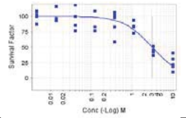	=	5.757	1.749826	0	100	-2.5125	0.8265	+LIMK	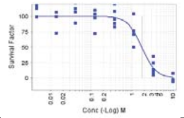	1.80
GSK317314A	BDP-00006257	=	7.1606	0.069086	0	100	-2.5883	0.9013	DMSO	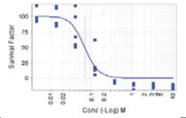	=	7.4134	0.038599	0	100	-14.35	0.8847	+LIMK	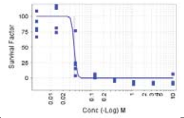	1.79
GW759710A	BDP-00006349	=	5.1772	6.649779	0	100	-4.0066	0.6921	DMSO	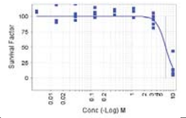	=	5.4232	3.77381	0	100	-10.188	0.8547	+LIMK	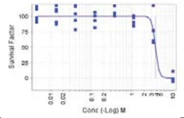	1.76
SB-742864	BDP-00006212	=	6.068	0.855112	0	100	-2.4279	0.8557	DMSO	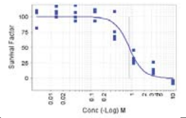	=	6.3101	0.489651	0	100	-1.9215	0.9594	+LIMK	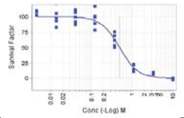	1.75
SB-698596-AC	BDP-00006201	=	5.0107	9.757068	0	100	-1.9437	0.1297	DMSO	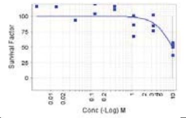	=	5.2505	5.617179	0	100	-1.5044	0.6929	+LIMK	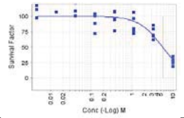	1.74
GW694234A	BDP-00006310	=	5.252	5.598222	0	100	-2.5479	0.6799	DMSO	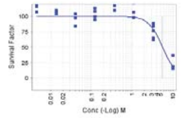	=	5.4876	3.254143	0	100	-11.958	0.7256	+LIMK	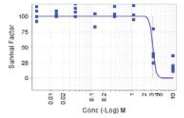	1.72

GW869810X	BDP-00006471	=	5.0596	8.717529	0	100	-4.9364	0.6484	DMSO	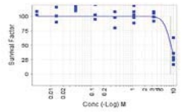	=	5.2913	5.113463	0	100	-5.265	0.8821	+LIMK	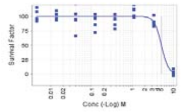	1.70
SB-284847-BT	BDP-00006451	=	5.4632	3.442016	0	100	-20.262	0.8576	DMSO	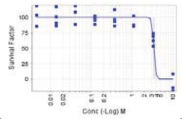	=	5.694	2.022867	0	100	-1.5461	0.8562	+LIMK	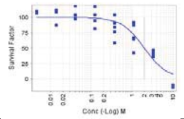	1.70
GSK466314A	BDP-00006268	=	5.2076	6.200311	0	100	-2.3165	0.7183	DMSO	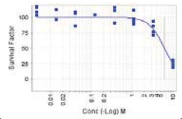	=	5.437	3.655691	0	100	-1.7797	0.8314	+LIMK	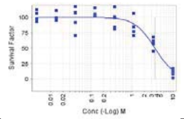	1.70
GW806290X	BDP-00006367	=	7.4804	0.033082	0	100	-1.6222	0.9409	DMSO	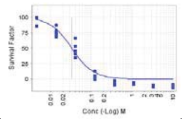	=	7.7053	0.019709	0	100	-1.3188	0.9191	+LIMK	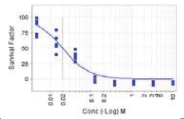	1.68
SB-226879	BDP-00006466	=	5.1721	6.72866	0	100	-2.9618	0.7313	DMSO	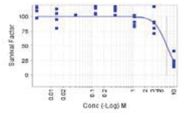	=	5.3926	4.04982	0	100	-1.8353	0.7488	+LIMK	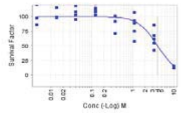	1.66
SB-250715	BDP-00006441	=	5.2485	5.642631	0	100	-2.9983	0.7564	DMSO	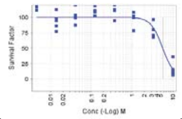	=	5.461	3.459376	0	100	-20.115	0.874	+LIMK	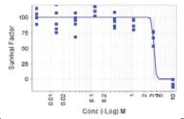	1.63
GW607049C	BDP-00006304	=	5.2685	5.389456	0	100	-4.2777	0.8358	DMSO	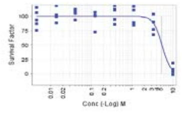	=	5.4802	3.309506	0	100	-18.848	0.7969	+LIMK	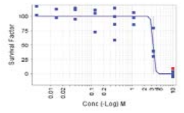	1.63

SB-409514	BDP-00006194	=	5.0246	9.448226	0	100	-17.184	0.8243	-DMSO	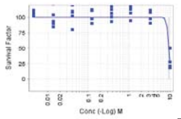	=	5.2359	5.809363	0	100	-3.475	0.7659	+LIMK	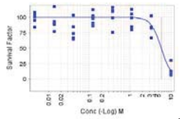	1.63
SB-253226	BDP-00006444	=	5.4648	3.429125	0	100	-27.568	0.8814	-DMSO	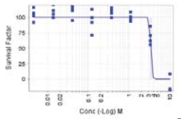	=	5.6648	2.163445	0	100	-3.3025	0.92	+LIMK	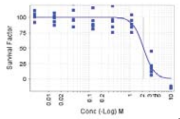	1.59
GW874091X	BDP-00006341	=	5.339	4.581064	0	100	-1.8008	0.8388	-DMSO	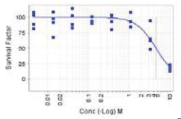	=	5.5308	2.945734	0	100	-1.2712	0.8419	+LIMK	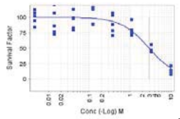	1.56
SB-220025-R	BDP-00006462	=	6.4983	0.317476	0	100	-20.282	0.9077	-DMSO	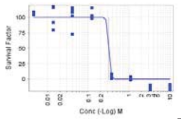	=	6.6734	0.212141	0	100	-5.3676	0.9051	+LIMK	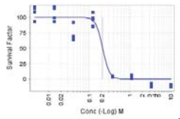	1.50
SB-242719	BDP-00006438	=	5.4485	3.56042	0	100	-19.576	0.7551	-DMSO	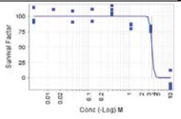	=	5.6142	2.430983	0	100	-1.8331	0.883	+LIMK	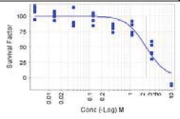	1.46
GW406108X	BDP-00006488	=	5.0698	8.515001	0	100	-3.5333	0.5243	-DMSO	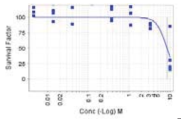	=	5.2044	6.245911	0	100	-3.8251	0.7677	+LIMK	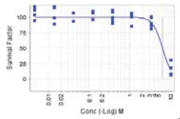	1.36
GW876790X	BDP-00006333	=	5.3025	4.982952	0	100	-2.5216	0.7229	-DMSO	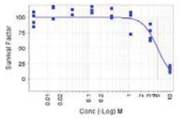	=	5.4308	3.708654	0	100	-2.3416	0.7234	+LIMK	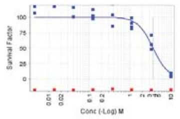	1.34

GSK1392956A	BDP-00006282	=	6.5798	0.263175	0	100	-2.0762	0.9494	-DMSO	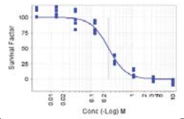	=	6.6972	0.200831	0	100	-1.5791	0.9211	+LIMK	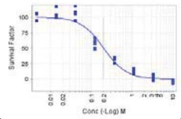	1.31
GSK2110236A	BDP-00006407	=	7.0611	0.08688	0	100	-2.377	0.9342	-DMSO	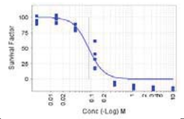	=	7.1773	0.066475	0	100	-2.4928	0.9411	+LIMK	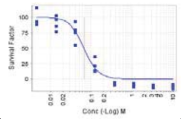	1.31
SB-390527	BDP-00006192	=	5.5006	3.157876	0	100	-2.0373	0.8285	-DMSO	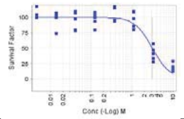	=	5.6113	2.447551	0	100	-3.4799	0.8825	+LIMK	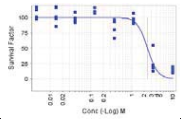	1.29
SB-750140	BDP-00006183	=	5.1166	7.644967	0	100	-1.5952	0.609	-DMSO	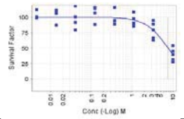	=	5.2216	6.003355	0	100	-1.7384	0.541	+LIMK	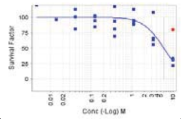	1.27
SB-678557-A	BDP-00006199	=	5.0045	9.897678	0	100	-2.2518	0.4337	-DMSO	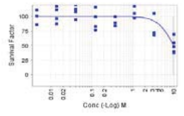	=	5.0987	7.966595	0	100	-3.5019	0.5736	+LIMK	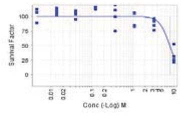	1.24
SB-686709-A	BDP-00006200	=	5.4843	3.278511	0	100	-2.6374	0.8571	-DMSO	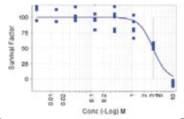	=	5.5741	2.666502	0	100	-2.382	0.7387	+LIMK	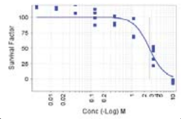	1.23
SB-400868-A	BDP-00006335	=	5.4299	3.716119	0	100	-20.377	0.8151	-DMSO	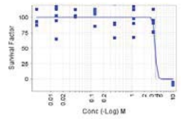	=	5.5021	3.146759	0	100	-19.2	0.8877	+LIMK	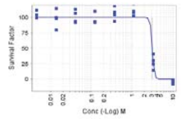	1.18

GW575808A	BDP-00006348	=	5.3356	4.617017	0	100	-3.0293	0.8471	-DMSO	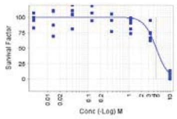	=	5.4057	3.929345	0	100	-3.6876	0.8093	+LIMK	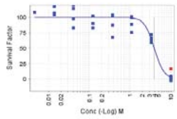	1.18
SB-732881-H	BDP-00006203	=	6.7717	0.169159	0	100	-3.5112	0.9222	-DMSO	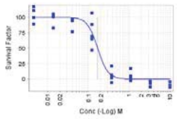	=	6.8315	0.147398	0	100	-2.958	0.9152	+LIMK	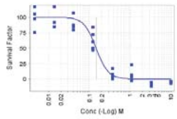	1.15
SB-741905	BDP-00006211	=	5.1582	6.947787	0	100	-3.2295	0.7396	-DMSO	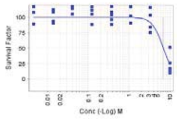	=	5.2087	6.184895	0	100	-4.8024	0.8241	+LIMK	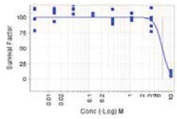	1.12
SB-739245-AC	BDP-00006209	=	5.4138	3.856274	0	100	-17.9	0.7808	-DMSO	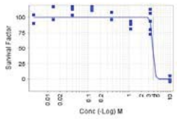	=	5.4602	3.465548	0	100	-14.394	0.8246	+LIMK	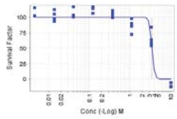	1.11
GSK949675A	BDP-00006226	=	5.3932	4.044171	0	100	-4.0243	0.8661	-DMSO	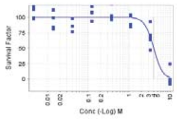	=	5.4254	3.754573	0	100	-5.4048	0.8848	+LIMK	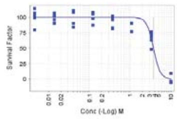	1.08
SB-732941	BDP-00006205	=	5.4603	3.465172	0	100	-17.797	0.6872	-DMSO	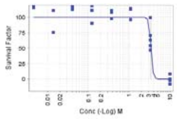	=	5.4875	3.254284	0	100	-15.916	0.7148	+LIMK	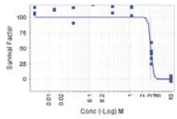	1.06
GW612286X	BDP-00006290	=	6.1648	0.684162	0	100	-1.5249	0.9066	-DMSO	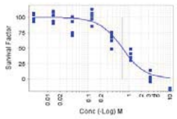	=	6.1811	0.658975	0	100	-1.74	0.935	+LIMK	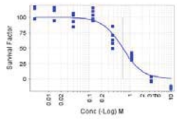	1.04

GSK978744A	BDP-00006262	=	6.9395	0.114957	0	100	-3.8906	0.9224	DMSO	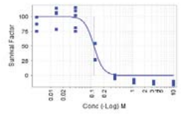	=	6.9526	0.111529	0	100	-20.92	0.9553	+LIMK	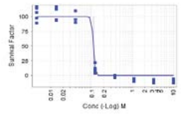	1.03
SB-239272	BDP-00006435	=	5.0039	9.909662	0	100	-4.2872	0.6619	DMSO	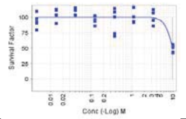	=	5.0094	9.786615	0	100	-2.6824	0.5349	+LIMK	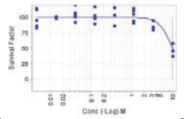	1.01
GW673715X	BDP-00006306	=	5.0266	9.405027	0	100	-17.064	0.7001	DMSO	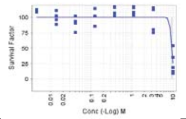	=	5.0054	9.875421	0	100	-16.669	0.4836	+LIMK	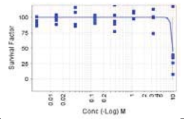	0.95
GSK312948A	BDP-00006321	=	5.1198	7.589701	0	100	-2.8029	0.5557	DMSO	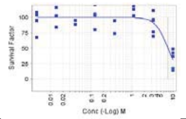	=	5.0903	8.122335	0	100	-2.2739	0.5187	+LIMK	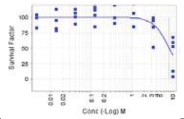	0.93
GW843682X	BDP-00006263	=	5.6479	2.249788	0	100	-1.6698	0.7858	DMSO	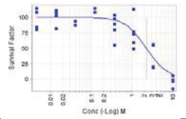	=	5.5226	3.002176	0	100	-23.203	0.8836	+LIMK	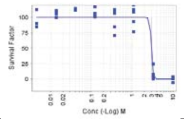	0.75
SB-742864	BDP-00006212	=	5.1788	6.625823	0	100	-5.7713	0.7153	DMSO	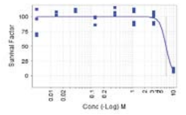	=	5.0422	9.074967	0	100	-15.239	0.7387	+LIMK	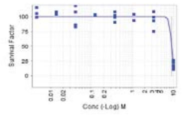	0.73
GSK237701A	BDP-00006256	=	5.2281	5.913738	0	100	-23.439	0.8063	DMSO	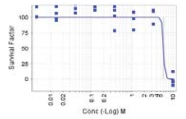	=	5.0856	8.210355	0	100	-20.468	0.7842	+LIMK	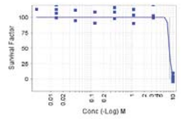	0.72

SB-814597	BDP-00006168	=	5.1802	6.603438	0	100	-2.3411	0.659	DMSO	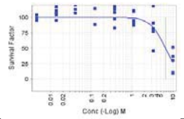	=	5.0002	9.996295	0	100	-0.8102	0.5441	+LIMK	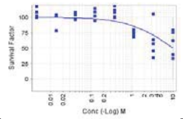	0.66
GSK614526A	BDP-00006331	=	5.2916	5.109279	0	100	-1.7832	0.7162	DMSO	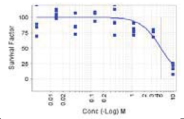	=	5.0866	8.192008	0	100	-1.3329	0.7131	+LIMK	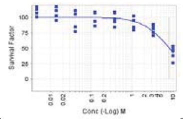	0.62
GW837331X	BDP-00006339	=	5.9756	1.057708	0	100	-0.7645	0.8495	DMSO	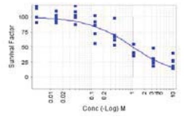	=	5.7664	1.712466	0	100	-0.5746	0.7468	+LIMK	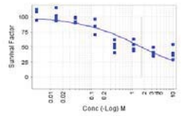	0.62
SB-675259-M	BDP-00006198	=	5.2681	5.393501	0	100	-0.9643	0.469	DMSO	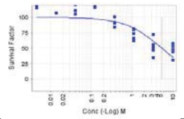	=	5.0488	8.937745	0	100	-1.0907	0.2609	+LIMK	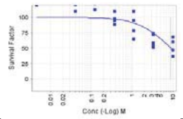	0.60
GW680191X	BDP-00006485	=	5.4394	3.635952	0	100	-1.8584	0.7966	DMSO	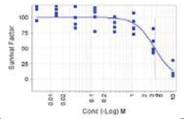	=	5.1861	6.515358	0	100	-0.5469	0.4694	+LIMK	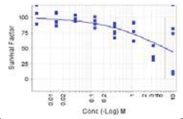	0.56
