# Supplementary material for: LIM kinase inhibitors disrupt mitotic microtubule organization and impair tumor cell proliferation
Source: Oncotarget. 2015 Nov 3;6(36):38469–86. doi: 10.18632/oncotarget.6288 (PMC4770715; doi:10.18632/oncotarget.6288)
Supplement: Supplementary file 5 [file oncotarget-06-38469-s005.pdf]

Supplier Ref	Formatted ID	REL pXC50 Modifier	REL pXC50	ABS XC50	Min	Max	Hillslope	R2	Condition	Graph	REL pXC50 Modifier	REL pXC50	ABS XC50	Min	Max	Hillslope	R2	Condition	Graph
GW769076X	BDP-00006145	<	5	<5	0	100	-0.8898	-0.1234	+DMSO	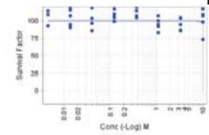	<	5	<5	0	100	-11.026	-0.2524	+LIMK	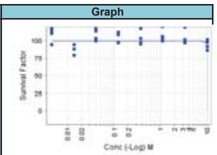
GW775608X	BDP-00006146	<	5	<5	0	100	-88.174	-0.3606	+DMSO	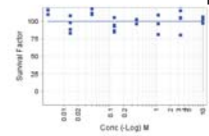	<	5	<5	0	100	-12.14	0.0858	+LIMK	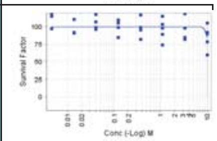
GW549390X	BDP-00006147	<	5	<5	0	100	-8.9223	-0.4163	+DMSO	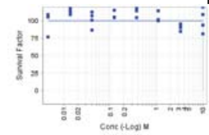	<	5	<5	0	100	-0.7895	0.2256	+LIMK	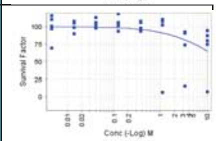
GW572399X	BDP-00006148	<	5	<5	0	100	-0.7388	0.5256	+DMSO	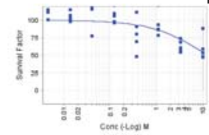	=	5.302	4.989159	0	100	-0.8315	0.7525	+LIMK	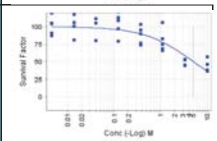
GW572401X	BDP-00006149	<	5	<5	0	100	-0.632	0.012	+DMSO	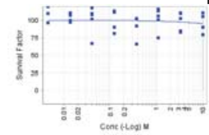	<	5	<5	0	100	-0.6019	0.1076	+LIMK	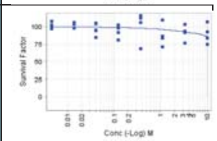
GW575533A	BDP-00006150	<	5	<5	0	100	-659.58	-0.3354	+DMSO	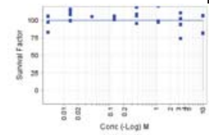	>	8.3372	>8.3372	0	100	3.4163	-0.1116	+LIMK	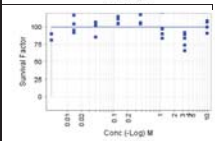

GW577921A	BDP-00006151	>	8.3372	>8.3372	0	100	9.2067	-0.2467	+DMSO	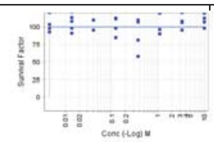	<	5	<5	0	100	-15.1	-1.9645	+LIMK	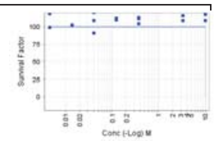
GW580509X	BDP-00006152	<	5	<5	0	100	-0.9055	0.0411	+DMSO	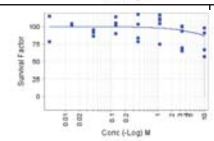	<	5	<5	0	100	-0.847	0.1844	+LIMK	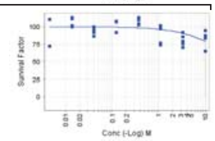
GW621431X	BDP-00006153	<	5	<5	0	100	-62.671	-0.8055	+DMSO	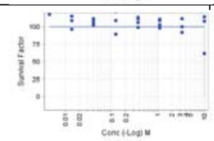	<	5	<5	0	100	-0.6663	0.0592	+LIMK	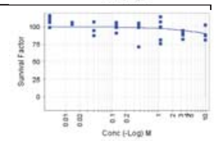
GW621970X	BDP-00006154	<	5	<5	0	100	-0.7053	0.2777	+DMSO	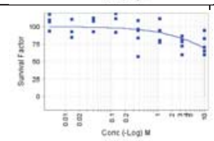	<	5	<5	0	100	-0.4555	0.2833	+LIMK	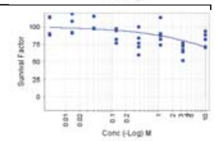
GW622055X	BDP-00006155	<	5	<5	0	100	-349.55	-0.3622	+DMSO	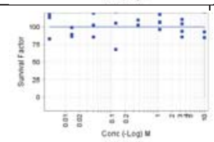	<	5	<5	0	100	-17.849	-0.0907	+LIMK	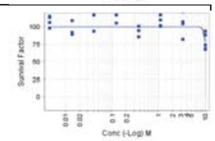
GW627834A	BDP-00006157	<	5	<5	0	100	-1.4697	-0.2761	+DMSO	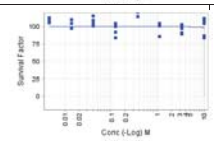	<	5	<5	0	100	-1.8995	-0.0824	+LIMK	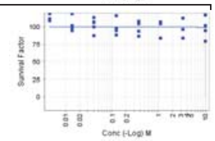

GW631581B	BDP-00006158	<	5	<5	0	100	-9.033	-1.2988	+DMSO	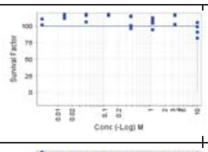	>	8.3372	>8.3372	0	100	-10.083	-0.3448	+LIMK	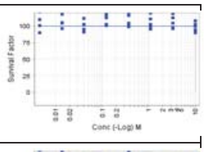
GW632046X	BDP-00006159	<	5	<5	0	100	-51.292	-0.6812	+DMSO	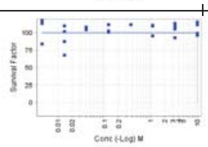	<	5	<5	0	100	-49.769	-0.3299	+LIMK	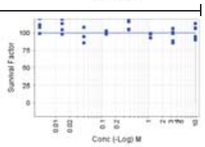
GW641155A	BDP-00006160	>	8.3372	>8.3372	0	100	-8.7732	-0.72	+DMSO	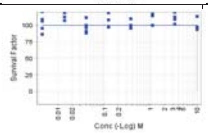	<	5	<5	0	100	-0.1176	0.0024	+LIMK	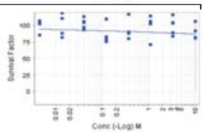
GW678313X	BDP-00006161	<	5	<5	0	100	-41.973	-0.2124	+DMSO	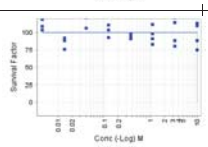	<	5	<5	0	100	-1.8773	-0.0845	+LIMK	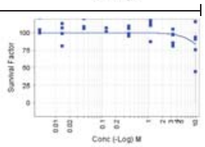
GSK718429A	BDP-00006162	<	5	<5	0	100	-11.646	-0.6831	+DMSO	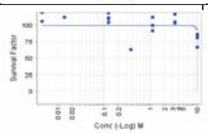	<	5	<5	0	100	-1.5185	0.2991	+LIMK	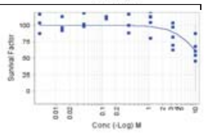
GW572738X	BDP-00006163	>	8.3372	>8.3372	0	100	-0.8464	0.0196	+DMSO	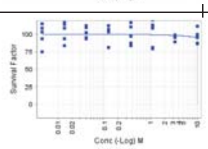	<	5	<5	0	100	-0.495	0.2911	+LIMK	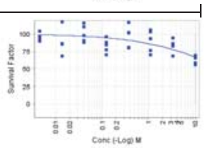

SB-347804	BDP-00006166	<	5	<5	0	100	-10.453	-0.2453	+DMSO	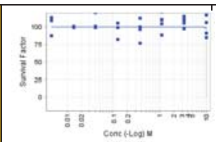	<	5	<5	0	100	-0.0833	0.011	+LIMK	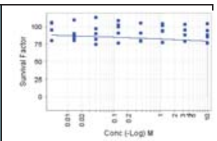
SB-657836-AAA	BDP-00006167	>	8.3372	>8.3372	0	100	4.8013	-0.5692	+DMSO	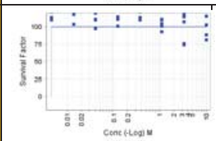	<	5	<5	0	100	-10.351	-0.1056	+LIMK	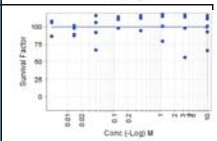
SB-814597	BDP-00006168	<	5	<5	0	100	-2.2604	0.1177	+DMSO	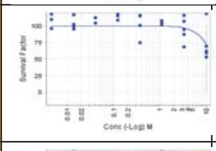	=	5.5694	2.695399	0	100	-0.7184	0.6242	+LIMK	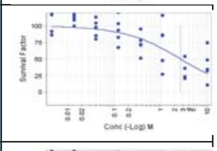
GSK586581A	BDP-00006169	<	5	<5	0	100	-11.429	0.0037	+DMSO	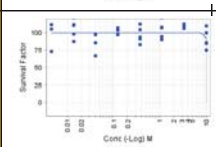	<	5	<5	0	100	-1.3891	-0.3614	+LIMK	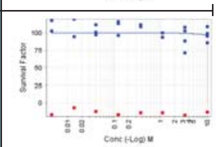
GSK605714A	BDP-00006170	<	5	<5	0	100	-187.2	-0.5254	+DMSO	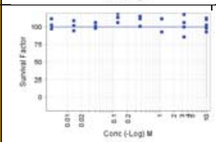	<	5	<5	0	100	-309.56	-0.2517	+LIMK	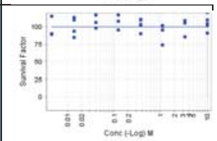
GSK620503A	BDP-00006171	<	5	<5	0	100	-1.9492	-0.3173	+DMSO	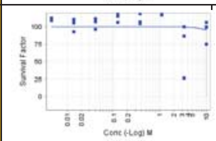	<	5	<5	0	100	-0.7035	0.1273	+LIMK	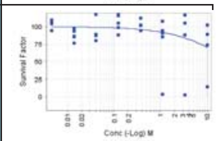

GSK625137A	BDP-00006172	<	5	<5	0	100	-11.024	-1.0064	+DMSO	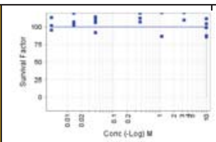	<	5	<5	0	100	-11.878	-0.4269	+LIMK	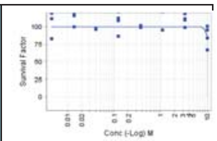
GSK635416A	BDP-00006173	<	5	<5	0	100	-27.082	-0.9441	+DMSO	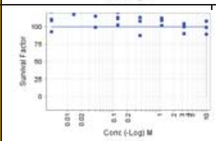	=	5.2434	5.709663	0	100	-1.7378	0.7379	+LIMK	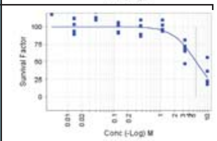
GSK711701A	BDP-00006174	<	5	<5	0	100	-0.9485	0.0788	+DMSO	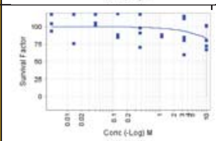	<	5	<5	0	100	-1.9835	-0.1554	+LIMK	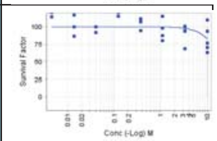
GW549034X	BDP-00006175	<	5	<5	0	100	-2.7254	-0.2732	+DMSO	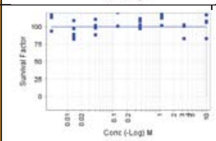	<	5	<5	0	100	-212.74	-0.1188	+LIMK	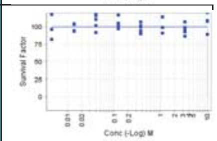
GW785404X	BDP-00006176	>	8.3372	>8.3372	0	100	3.0603	-0.1065	+DMSO	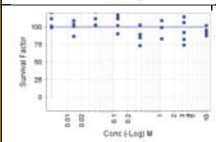	>	8.3372	>8.3372	0	100	0.1814	0.0147	+LIMK	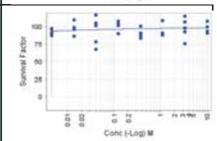
SB-734117	BDP-00006177	<	5	<5	0	100	-163.89	-1.218	+DMSO	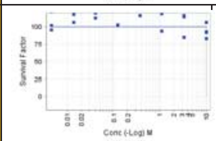	<	5	<5	0	100	-1.2119	-0.0747	+LIMK	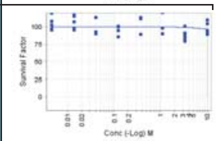

SB-736290	BDP-00006178	<	5	<5	0	100	-66.557	-0.2821	+DMSO	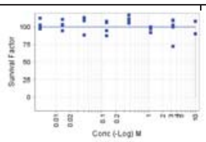	<	5	<5	0	100	-13.244	-1.5402	+LIMK	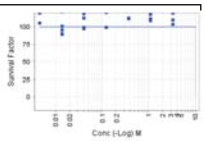
SB-736302	BDP-00006179	<	5	<5	0	100	-12.366	0.1145	+DMSO	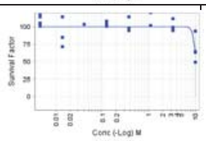	=	5.0084	9.808709	0	100	-16.262	0.2257	+LIMK	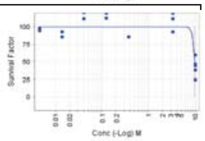
SB-738561	BDP-00006180	<	5	<5	0	100	-421.05	-0.4962	+DMSO	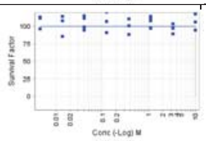	<	5	<5	0	100	-320.19	-0.1111	+LIMK	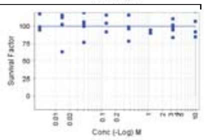
SB-737198	BDP-00006181	>	8.3372	>8.3372	0	100	0.1414	0.0181	+DMSO	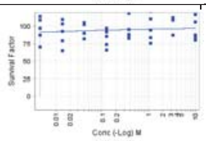	<	5	<5	0	100	-0.0662	0.0075	+LIMK	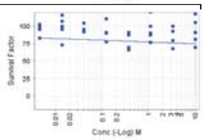
SB-744941	BDP-00006182	<	5	<5	0	100	-11.729	-0.1106	+DMSO	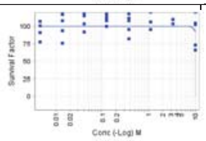	<	5	<5	0	100	-22.937	-0.7956	+LIMK	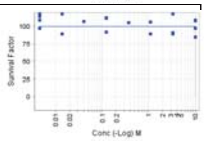
SB-751148	BDP-00006184	<	5	<5	0	100	-10.302	-0.0459	+DMSO	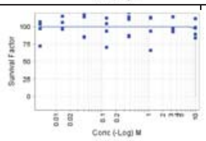	<	5	<5	0	100	-41.712	-0.3729	+LIMK	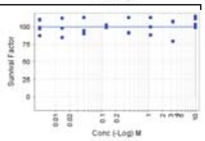

SB-751399	BDP-00006185	<	5	<5	0	100	-1.2405	-0.0489	+DMSO	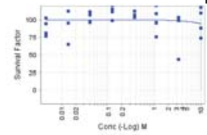	<	5	<5	0	100	-19.838	-0.2124	+LIMK	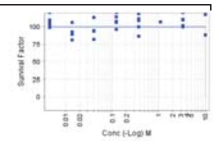
SB-333612	BDP-00006186	<	5	<5	0	100	-0.8213	0.1806	+DMSO	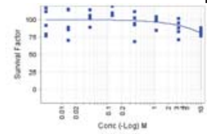	<	5	<5	0	100	-0.4273	0.0596	+LIMK	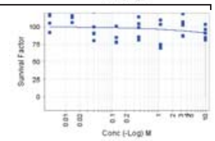
SB-358518	BDP-00006187	<	5	<5	0	100	-14.318	-0.1538	+DMSO	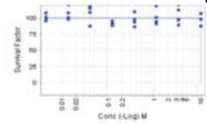	<	5	<5	0	100	-2.3144	-0.2975	+LIMK	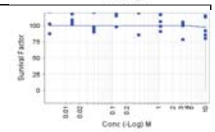
SB-360741	BDP-00006188	>	8.3372	>8.3372	0	100	1.8581	0.0763	+DMSO	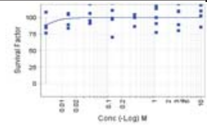	>	8.3372	>8.3372	0	100	0.2306	0.008	+LIMK	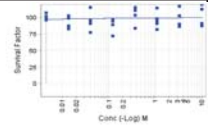
SB-376719	BDP-00006190	<	5	<5	0	100	-1.2498	0.5651	+DMSO	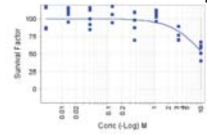	<	5	<5	0	100	-0.8953	-0.0891	+LIMK	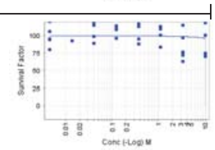
SB-390523	BDP-00006191	<	5	<5	0	100	-522.85	-0.1281	+DMSO	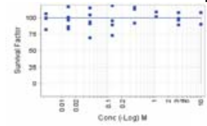	<	5	<5	0	100	-10.672	-0.284	+LIMK	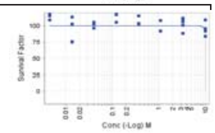

SB-409513	BDP-00006193	<	5	<5	0	100	-0.4846	0.0232	+DMSO	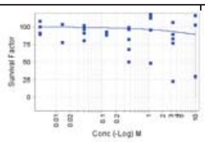	>	8.3372	>8.3372	0	100	1.5341	-0.0028	+LIMK	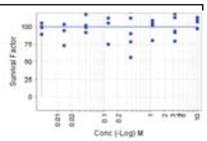
SKF-62604	BDP-00006195	<	5	<5	0	100	-1.3538	-0.0209	+DMSO	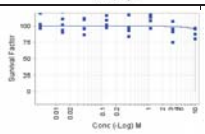	>	8.3372	>8.3372	0	100	0.0124	0	+LIMK	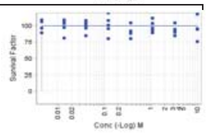
GW819230X	BDP-00006196	<	5	<5	0	100	-12.427	-0.3295	+DMSO	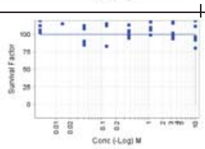	<	5	<5	0	100	-11.264	0.0088	+LIMK	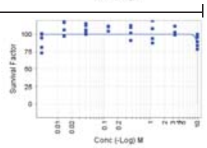
GSK319347A	BDP-00006197	<	5	<5	0	100	-3.0707	-1.5512	+DMSO	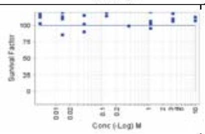	<	5	<5	0	100	-11.83	-0.4518	+LIMK	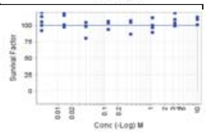
SB-711237	BDP-00006202	<	5	<5	0	100	-203.72	-0.7312	+DMSO	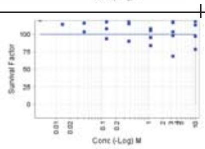	<	5	<5	0	100	-2.0627	-0.0663	+LIMK	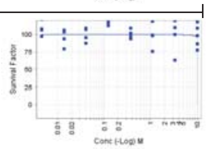
SB-735465	BDP-00006206	<	5	<5	0	100	-9.2346	-0.2865	+DMSO	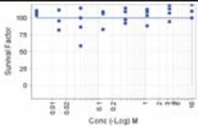	>	8.3372	>8.3372	0	100	-0.8593	0.2166	+LIMK	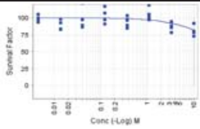

SB-738482	BDP-00006208	<	5	<5	0	100	-7.3069	-0.0396	+DMSO	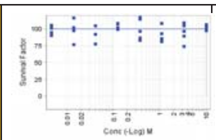	<	5	<5	0	100	-133.47	-0.2756	+LIMK	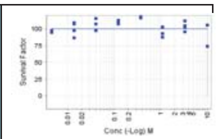
SB-739452	BDP-00006210	<	5	<5	0	100	-0.7717	0.0365	+DMSO	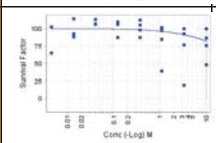	<	5	<5	0	100	-0.968	-0.0209	+LIMK	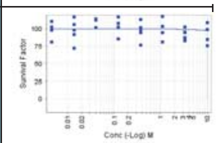
SB-742865	BDP-00006213	>	8.3372	>8.3372	0	100	2.1254	-0.1137	+DMSO	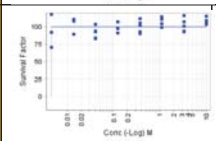	<	5	<5	0	100	-9.8522	-0.0808	+LIMK	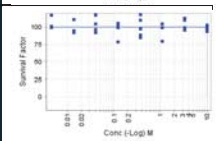
SB-743899	BDP-00006214	<	5	<5	0	100	-193.42	-0.5743	+DMSO	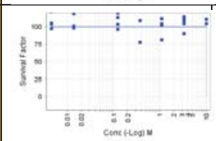	<	5	<5	0	100	-42.628	-0.5691	+LIMK	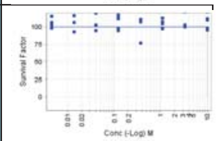
GW651576X	BDP-00006215	<	5	<5	0	100	-13.673	-0.6471	+DMSO	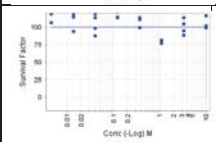	<	5	<5	0	100	-11.947	-0.028	+LIMK	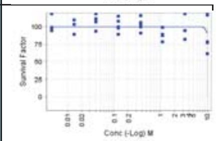
GW659893X	BDP-00006216	<	5	<5	0	100	-11.5	-0.3576	+DMSO	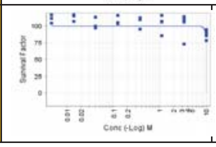	<	5	<5	0	100	-1.1272	0.3678	+LIMK	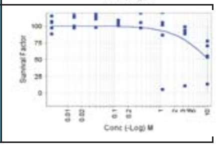

GW703087X	BDP-00006217	<	5	<5	0	100	-6.4791	0.7797	+DMSO	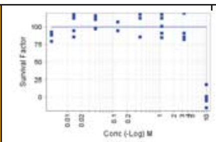	=	5.1474	7.121942	0	100	-4.7437	0.6664	+LIMK	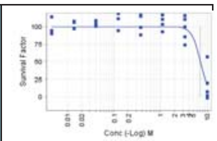
GW772405X	BDP-00006218	<	5	<5	0	100	-11.289	-0.7458	+DMSO	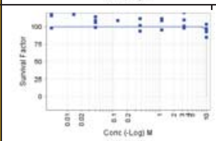	=	5.31	4.897392	0	100	-3.8292	0.7909	+LIMK	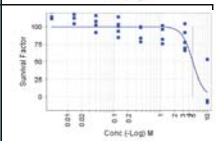
GW794726X	BDP-00006219	<	5	<5	0	100	-484.37	-0.4551	+DMSO	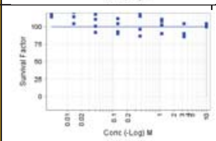	<	5	<5	0	100	-2.7018	0.0425	+LIMK	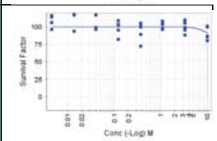
GW799251X	BDP-00006220	<	5	<5	0	100	-4.0758	0.282	+DMSO	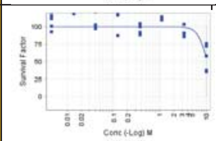	<	5	<5	0	100	-2.3216	0.1701	+LIMK	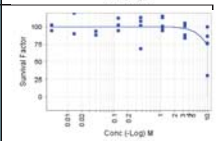
GW807930X	BDP-00006221	<	5	<5	0	100	-0.0759	0.0047	+DMSO	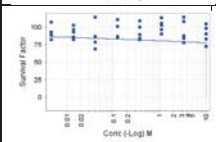	<	5	<5	0	100	-0.5385	0.3021	+LIMK	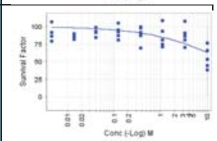
GSK1000163A	BDP-00006222	<	5	<5	0	100	-222.97	-0.6918	+DMSO	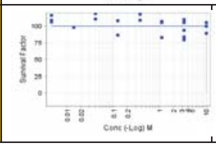	<	5	<5	0	100	-11.933	0.0671	+LIMK	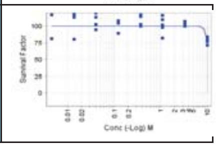

GSK938890A	BDP-00006224	=	5.0269	9.400083	0	100	-17.819	0.3707	+DMSO	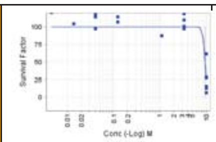	<	5	<5	0	100	-7.333	-0.0007	+LIMK	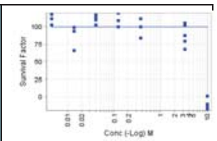
GW824645A	BDP-00006227	<	5	<5	0	100	-313.93	-0.5349	+DMSO	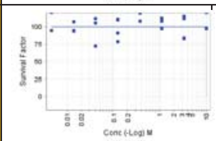	>	8.3372	>8.3372	0	100	1.0019	-0.019	+LIMK	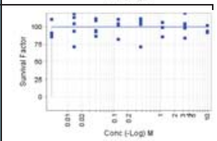
GW824645A	BDP-00006227	<	5	<5	0	100	-12.29	-0.1773	+DMSO	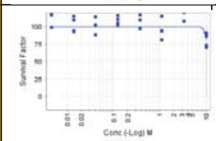	=	5.0146	9.668382	0	100	-17.008	0.4522	+LIMK	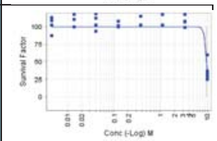
GW831090X	BDP-00006228	<	5	<5	0	100	-11.363	-0.3577	+DMSO	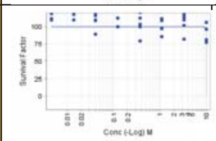	<	5	<5	0	100	-11.481	-0.0659	+LIMK	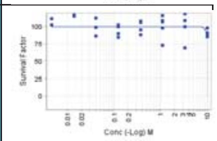
GW831091X	BDP-00006229	>	8.3372	>8.3372	0	100	11.167	-0.1619	+DMSO	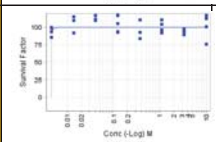	<	5	<5	0	100	-1.4624	0.0824	+LIMK	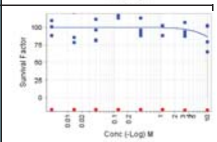
GW427984X	BDP-00006231	>	8.3372	>8.3372	0	100	2.1651	-0.6617	+DMSO	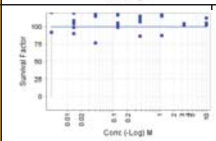	<	5	<5	0	100	-609.85	-0.7235	+LIMK	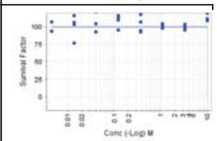

GW432441X	BDP-00006232	<	5	<5	0	100	-11.444	-0.0247	+DMSO	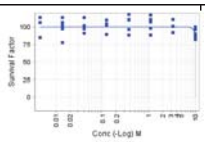	<	5	<5	0	100	-0.4143	0.0329	+LIMK	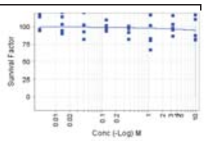
GW439255X	BDP-00006234	<	5	<5	0	100	-10.646	-0.2244	+DMSO	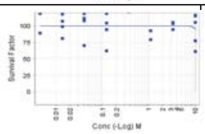	=	5.0281	9.372745	0	100	-1.2494	0.3881	+LIMK	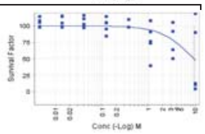
GW441806A	BDP-00006235	<	5	<5	0	100	-298.7	-0.471	+DMSO	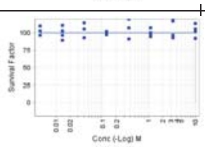	<	5	<5	0	100	-202.4	-0.2942	+LIMK	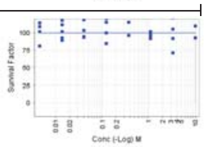
GW445012X	BDP-00006236	<	5	<5	0	100	-76.581	-1.891	+DMSO	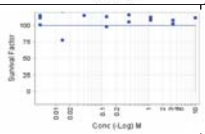	<	5	<5	0	100	-479.75	-0.8507	+LIMK	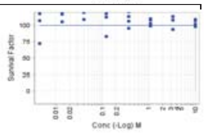
GW445014X	BDP-00006237	<	5	<5	0	100	-10.799	-0.1881	+DMSO	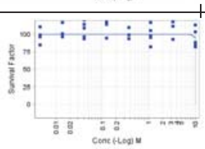	<	5	<5	0	100	-11.252	-0.111	+LIMK	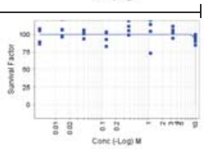
GW445015X	BDP-00006238	<	5	<5	0	100	-147.16	-0.524	+DMSO	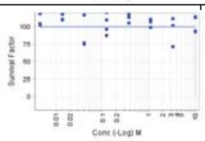	<	5	<5	0	100	-7.5516	-0.1773	+LIMK	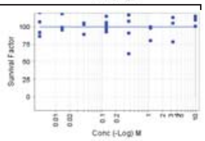

GW445017X	BDP-00006239	<	5	<5	0	100	-32.528	-0.2668	+DMSO	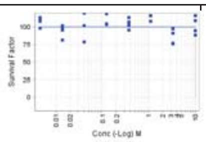	<	5	<5	0	100	-19.078	-0.6123	+LIMK	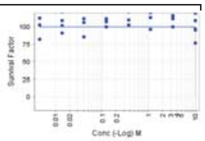
GW450241X	BDP-00006240	<	5	<5	0	100	-16.157	0.2664	+DMSO	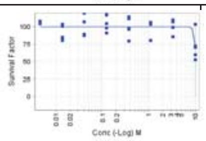	<	5	<5	0	100	-6.2267	0.5705	+LIMK	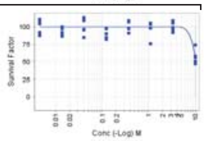
GW458344A	BDP-00006241	<	5	<5	0	100	-10.746	-0.1279	+DMSO	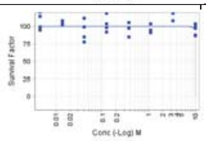	>	8.3372	>8.3372	0	100	0.506	0.0236	+LIMK	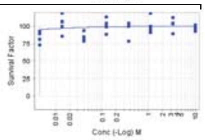
GW459057A	BDP-00006242	<	5	<5	0	100	-5.8528	-0.2901	+DMSO	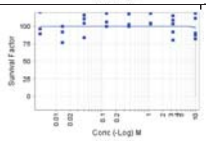	<	5	<5	0	100	-655.86	-1.2129	+LIMK	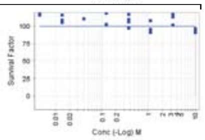
GW743024X	BDP-00006243	<	5	<5	0	100	-0.477	0.1578	+DMSO	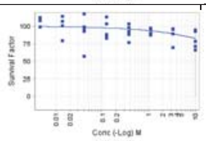	<	5	<5	0	100	-1.0919	0.0909	+LIMK	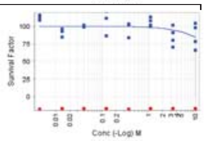
GW782907X	BDP-00006244	<	5	<5	0	100	-12.07	-0.1379	+DMSO	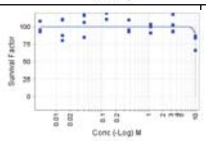	=	5.01	9.773205	0	100	-7.3528	0.6034	+LIMK	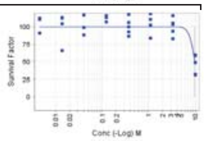

GW782912X	BDP-00006245	<	5	<5	0	100	-26.046	-0.7865	+DMSO	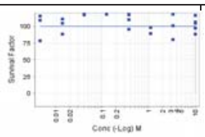	<	5	<5	0	100	-1.7806	-0.1393	+LIMK	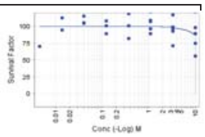
GW785974X	BDP-00006246	<	5	<5	0	100	-11.798	-0.2236	+DMSO	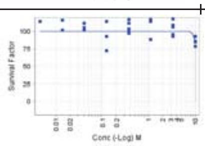	<	5	<5	0	100	-10.137	-0.0324	+LIMK	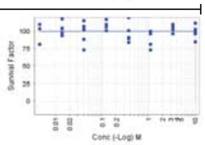
GW796920X	BDP-00006247	<	5	<5	0	100	-7.2772	-0.2132	+DMSO	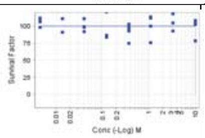	<	5	<5	0	100	-10.545	-0.0352	+LIMK	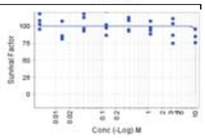
GW796921X	BDP-00006248	<	5	<5	0	100	-67.382	-0.1048	+DMSO	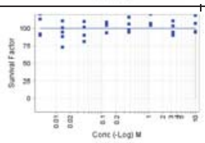	<	5	<5	0	100	-290.25	-0.3788	+LIMK	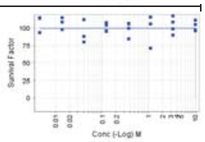
GW806776X	BDP-00006249	<	5	<5	0	100	-3.3516	-0.2153	+DMSO	[IMG]	<	5	<5	0	100	-0.4519	0.0326	+LIMK	[IMG]
GW701032X	BDP-00006250	<	5	<5	0	100	-7.0958	-0.3054	+DMSO	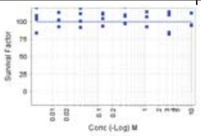	<	5	<5	0	100	-9.9286	-0.296	+LIMK	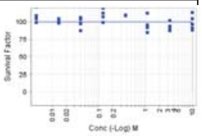

GW708893X	BDP-00006251	<	5	<5	0	100	-123.62	-0.0435	+DMSO	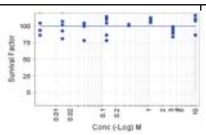	>	8.3372	>8.3372	0	100	10.514	-0.202	+LIMK	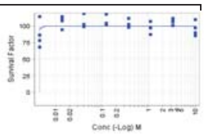
GW734508X	BDP-00006252	<	5	<5	0	100	-3.7797	-0.6028	+DMSO	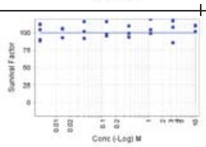	<	5	<5	0	100	-9.4452	-0.2024	+LIMK	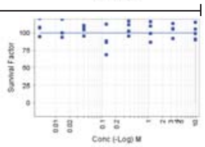
GW607117X	BDP-00006253	<	5	<5	0	100	-0.3855	0.0249	+DMSO	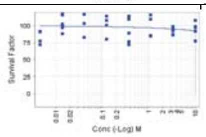	<	5	<5	0	100	-0.766	0.0564	+LIMK	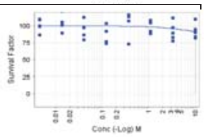
GW856804X	BDP-00006254	<	5	<5	0	100	-21.793	-0.3151	+DMSO	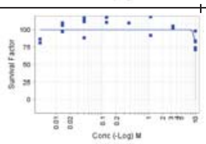	>	8.3372	>8.3372	0	100	4.229	-1.8474	+LIMK	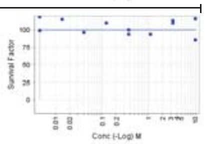
GSK237701A	BDP-00006256	<	5	<5	0	100	42.534	-15.208	+DMSO	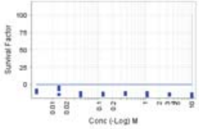	<	5	<5	0	100	3.7416	-13.038	+LIMK	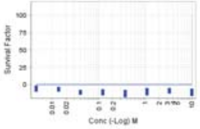
GSK326090A	BDP-00006259	<	5	<5	0	100	-11.792	-0.5584	+DMSO	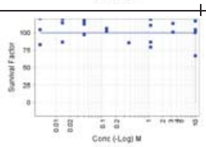	<	5	<5	0	100	-12.332	-0.2917	+LIMK	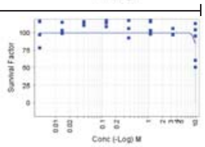

GSK326090A	BDP-00006259	<	5	<5	0	100	4.1568	-3.633	+DMSO	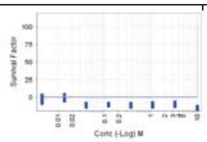	<	5	<5	0	100	8.2997	-8.0656	+LIMK	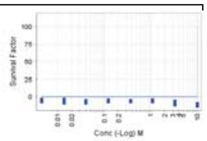
GSK571989A	BDP-00006260	<	5	<5	0	100	-283.05	-0.3121	+DMSO	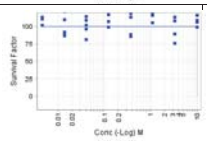	<	5	<5	0	100	-567.29	-0.8186	+LIMK	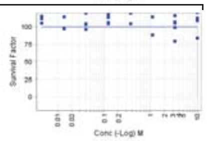
GSK571989A	BDP-00006260	<	5	<5	0	100	21.497	-2.6174	+DMSO	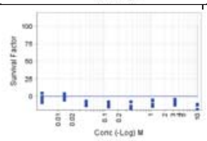	<	5	<5	0	100	7.3824	-4.7146	+LIMK	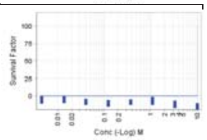
GSK579289A	BDP-00006261	=	5.0106	9.759433	0	100	-16.838	0.4828	+DMSO	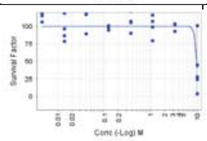	<	5	<5	0	100	-14.094	0.242	+LIMK	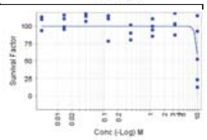
GSK579289A	BDP-00006261	<	5	<5	0	100	2.6639	-33.376	+DMSO	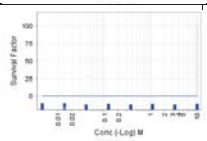	<	5	<5	0	100	7.5394	-26.932	+LIMK	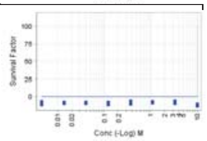
GSK180736A	BDP-00006265	<	5	<5	0	100	-41.064	-0.0971	+DMSO	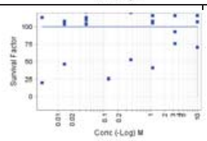	<	5	<5	0	100	-448.06	-1.9731	+LIMK	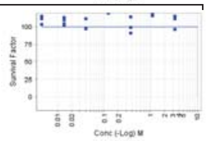

GSK270822A	BDP-00006266	<	5	<5	0	100	-1.6117	0.4831	+DMSO	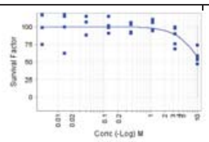	=	5.1442	7.17477	0	100	-0.9384	0.5453	+LIMK	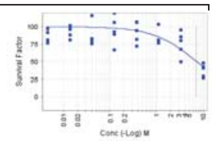
GSK299115A	BDP-00006267	<	5	<5	0	100	-1.608	-0.1682	+DMSO	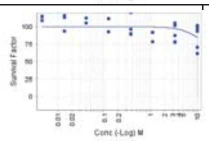	<	5	<5	0	100	-11.658	-0.3468	+LIMK	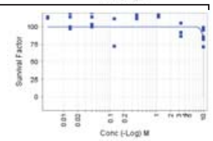
GSK466317A	BDP-00006269	<	5	<5	0	100	-2.7436	0.3186	+DMSO	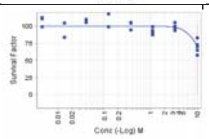	<	5	<5	0	100	-13.906	0.348	+LIMK	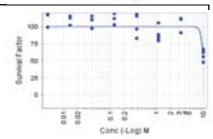
GW461104A	BDP-00006270	<	5	<5	0	100	-11.392	0.0811	+DMSO	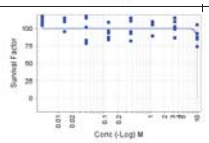	<	5	<5	0	100	-12.112	0.1731	+LIMK	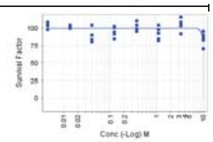
GW569530A	BDP-00006271	<	5	<5	0	100	-322.9	-0.1331	+DMSO	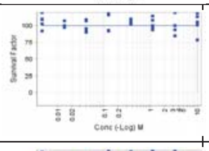	<	5	<5	0	100	-11.072	-0.0376	+LIMK	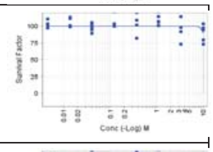
GR105659X	BDP-00006272	<	5	<5	0	100	-12.532	0.0327	+DMSO	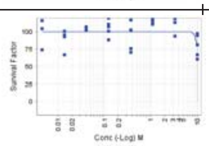	<	5	<5	0	100	-13.179	-0.1032	+LIMK	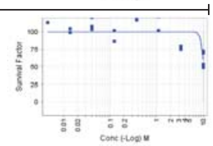

GW275616X	BDP-00006273	<	5	<5	0	100	-39.114	-0.6047	+DMSO	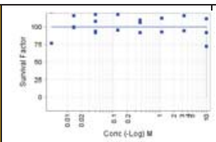	<	5	<5	0	100	-11.983	0.0183	+LIMK	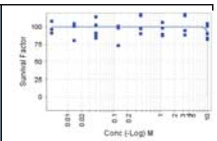
GW278681X	BDP-00006274	<	5	<5	0	100	-12.645	0.1399	+DMSO	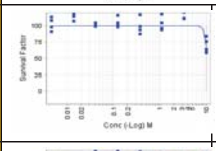	<	5	<5	0	100	-13.769	0.4044	+LIMK	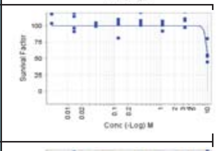
GW284408X	BDP-00006275	<	5	<5	0	100	-440.67	-0.252	+DMSO	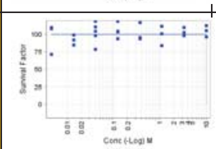	>	8.3372	>8.3372	0	100	11.217	0.0369	+LIMK	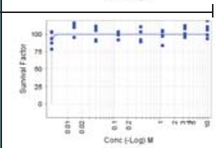
GW301789X	BDP-00006276	<	5	<5	0	100	-0.3039	0.0346	+DMSO	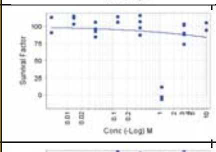	<	5	<5	0	100	-0.3554	0.132	+LIMK	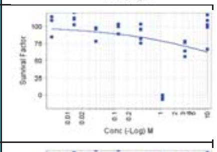
GW441756X	BDP-00006277	<	5	<5	0	100	-561.34	-1.1388	+DMSO	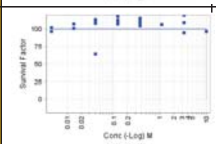	<	5	<5	0	100	-9.7525	-0.2968	+LIMK	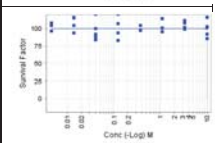
GW441756X	BDP-00006277	<	5	<5	0	100	-11.779	-0.069	+DMSO	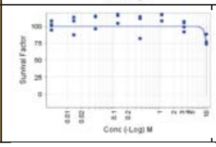	=	5.0499	8.913817	0	100	-3.7858	0.5774	+LIMK	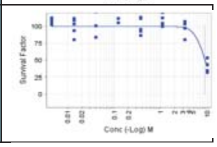

GW442130X	BDP-00006278	<	5	<5	0	100	-1.3151	-0.255	+DMSO	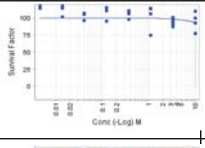	<	5	<5	0	100	-1.4452	0.5714	+LIMK	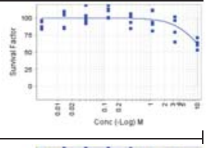
GW679410X	BDP-00006284	<	5	<5	0	100	-1.4402	0.0467	+DMSO	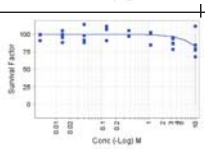	<	5	<5	0	100	-12.476	-0.1165	+LIMK	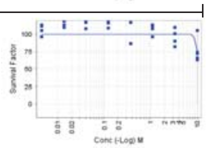
GW680975X	BDP-00006285	<	5	<5	0	100	-573.57	-0.3053	+DMSO	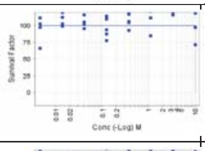	<	5	<5	0	100	-11.518	-0.1966	+LIMK	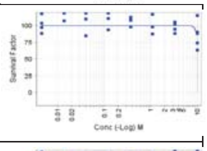
GW682841X	BDP-00006286	<	5	<5	0	100	-18.505	-1.6269	+DMSO	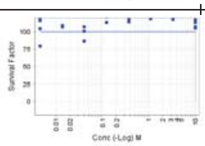	<	5	<5	0	100	-191.05	-1.3243	+LIMK	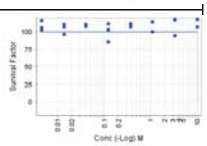
GW695874X	BDP-00006287	>	8.3372	>8.3372	0	100	4.9489	-3.2088	+DMSO	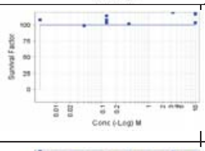	<	5	<5	0	100	-12.074	-1.3888	+LIMK	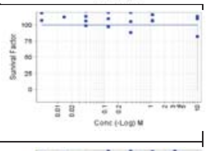
GW711782X	BDP-00006288	<	5	<5	0	100	-10.892	-0.3227	+DMSO	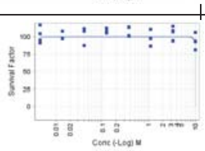	<	5	<5	0	100	-11.713	-0.2522	+LIMK	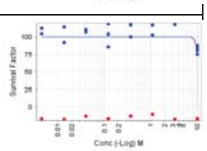

GW410563A	BDP-00006289	<	5	<5	0	100	-12.244	-0.0152	+DMSO	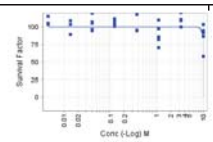	<	5	<5	0	100	-2.4144	0.1366	+LIMK	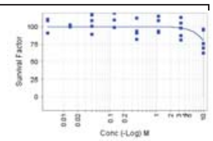
GW654652C	BDP-00006291	<	5	<5	0	100	-11.965	-0.5467	+DMSO	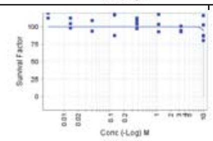	<	5	<5	0	100	-1.8026	-0.4931	+LIMK	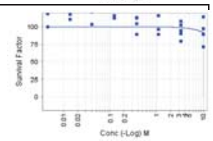
GW770220A	BDP-00006292	<	5	<5	0	100	-2.0148	-0.0694	+DMSO	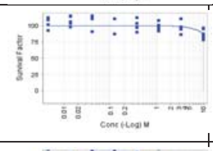	<	5	<5	0	100	-1.294	0.2736	+LIMK	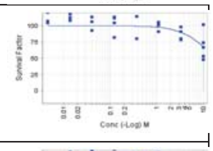
GW771127A	BDP-00006293	<	5	<5	0	100	-1.7564	0.2561	+DMSO	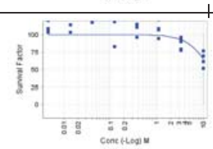	=	5.153	7.030964	0	100	-1.9906	0.5331	+LIMK	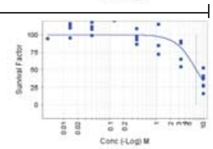
GSK953913A	BDP-00006294	<	5	<5	0	100	-22.429	-1.805	+DMSO	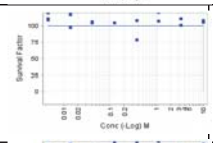	<	5	<5	0	100	-1.0649	-0.1208	+LIMK	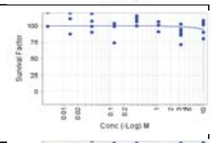
GSK980961A	BDP-00006295	<	5	<5	0	100	-3.5816	-0.1421	+DMSO	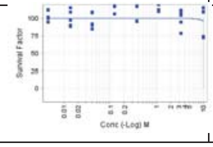	<	5	<5	0	100	-572.37	-0.3613	+LIMK	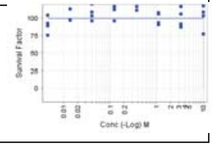

GW830365A	BDP-00006299	<	5	<5	0	100	-1.9632	0.1801	+DMSO	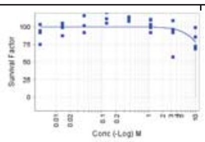	<	5	<5	0	100	-3.4352	0.2689	+LIMK	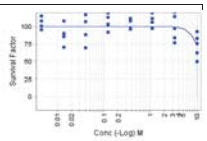
GW830900A	BDP-00006300	<	5	<5	0	100	-1.0065	0.0374	+DMSO	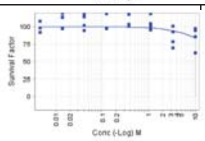	<	5	<5	0	100	-1.4701	0.5342	+LIMK	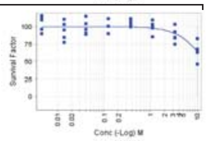
GSK248233A	BDP-00006301	<	5	<5	0	100	-2.2014	-0.0708	+DMSO	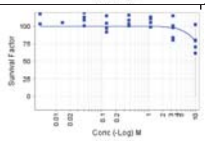	<	5	<5	0	100	-0.527	0.302	+LIMK	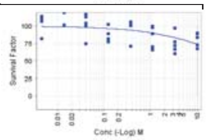
GSK269962B	BDP-00006302	<	5	<5	0	100	-0.328	0.1554	+DMSO	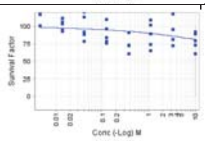	=	5.0131	9.703561	0	100	-0.5309	0.638	+LIMK	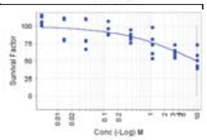
GW589961A	BDP-00006303	<	5	<5	0	100	-1.6352	0.0002	+DMSO	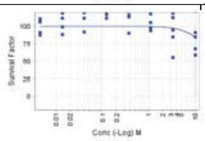	<	5	<5	0	100	-0.8574	0.0821	+LIMK	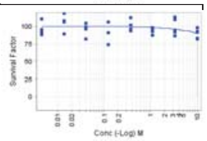
GW659386A	BDP-00006305	<	5	<5	0	100	-1.5594	0.1623	+DMSO	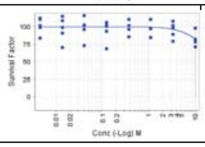	<	5	<5	0	100	-20.304	0.1711	+LIMK	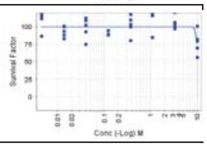

GW680908A	BDP-00006307	<	5	<5	0	100	-59.237	-0.2992	+DMSO	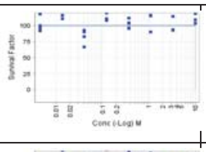	>	8.3372	>8.3372	0	100	0.0057	-0.0003	+LIMK	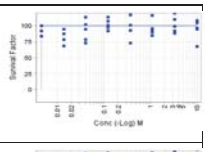
GW693917A	BDP-00006309	<	5	<5	0	100	-283.42	-2.5784	+DMSO	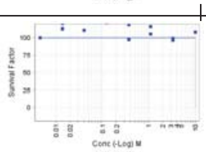	>	8.3372	>8.3372	0	100	4.8887	-2.2481	+LIMK	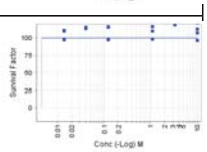
GW694590A	BDP-00006311	>	8.3372	>8.3372	0	100	2.4949	-0.0365	+DMSO	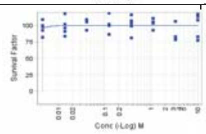	<	5	<5	0	100	-9.5533	-0.6785	+LIMK	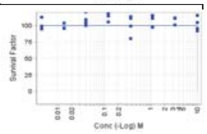
GW700494A	BDP-00006312	<	5	<5	0	100	-0.7415	0.3143	+DMSO	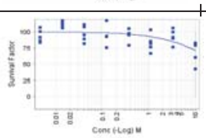	=	5.4295	3.719627	0	100	-27.052	0.8569	+LIMK	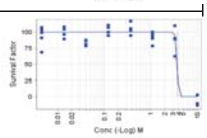
GSK1030059A	BDP-00006317	<	5	<5	0	100	-44.241	-2.1686	+DMSO	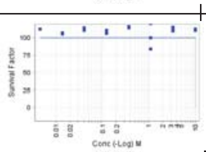	>	8.3372	>8.3372	0	100	4.9637	-2.911	+LIMK	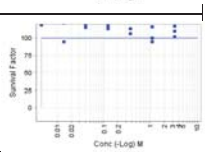
GSK1030061A	BDP-00006318	<	5	<5	0	100	-240.93	-0.8379	+DMSO	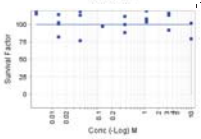	<	5	<5	0	100	-11.47	-0.4809	+LIMK	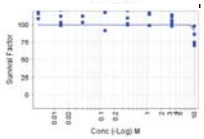

GSK1030062A	BDP-00006319	<	5	<5	0	100	-16.633	-0.5739	+DMSO	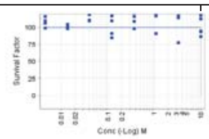	<	5	<5	0	100	-9.4005	-0.2767	+LIMK	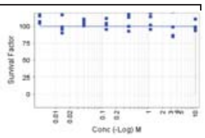
GSK204925A	BDP-00006320	<	5	<5	0	100	-11.161	-1.1518	+DMSO	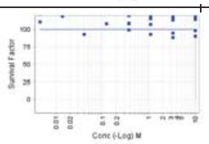	<	5	<5	0	100	-2.1984	-0.3718	+LIMK	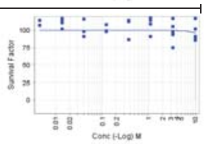
GW804482X	BDP-00006322	>	8.3372	>8.3372	0	100	10.864	-0.2269	+DMSO	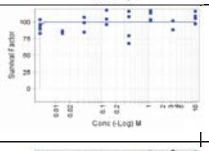	<	5	<5	0	100	-13.97	-1.1251	+LIMK	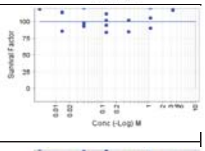
GW853609X	BDP-00006324	>	8.3372	>8.3372	0	100	3.618	-0.0371	+DMSO	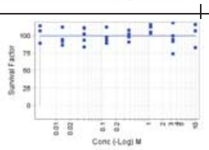	>	8.3372	>8.3372	0	100	0.5215	0.009	+LIMK	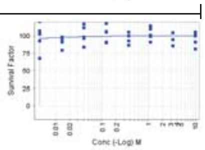
GW693481X	BDP-00006325	<	5	<5	0	100	-165.36	-1.6782	+DMSO	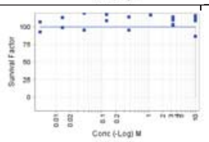	<	5	<5	0	100	-65.089	-0.7392	+LIMK	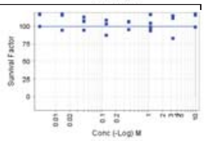
GW780159X	BDP-00006326	<	5	<5	0	100	-334.02	-0.385	+DMSO	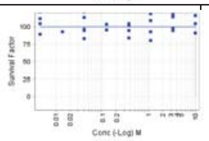	<	5	<5	0	100	-529.68	-0.6637	+LIMK	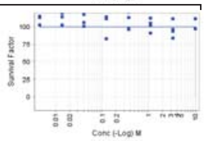

GW785804X	BDP-00006327	<	5	<5	0	100	-4.0497	0.0158	+DMSO	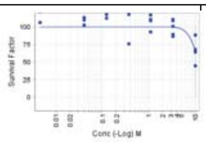	=	5.0047	9.89321	0	100	-16.904	0.5889	+LIMK	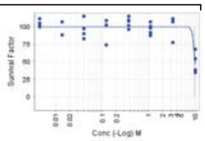
GW786460X	BDP-00006328	<	5	<5	0	100	-72.281	-0.4615	+DMSO	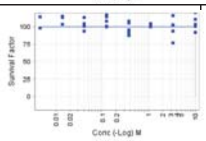	<	5	<5	0	100	-97.769	-0.4585	+LIMK	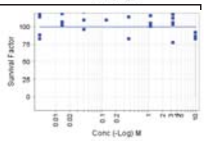
GSK554170A	BDP-00006329	<	5	<5	0	100	-1.1961	0.5857	+DMSO	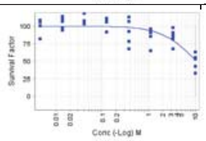	<	5	<5	0	100	-0.7901	0.5134	+LIMK	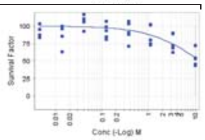
GSK561866B	BDP-00006330	<	5	<5	0	100	-160.39	-0.4834	+DMSO	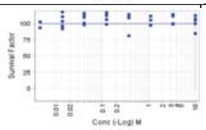	>	8.3372	>8.3372	0	100	3.5266	-0.532	+LIMK	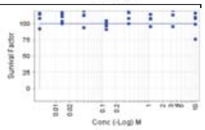
SB-759335-B	BDP-00006334	<	5	<5	0	100	-1.0418	-0.055	+DMSO	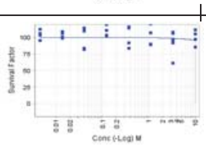	<	5	<5	0	100	-1.0332	0.1295	+LIMK	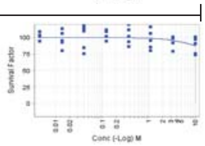
SB-431533	BDP-00006336	<	5	<5	0	100	-11.457	0.06	+DMSO	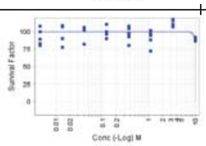	<	5	<5	0	100	-12.65	0.5663	+LIMK	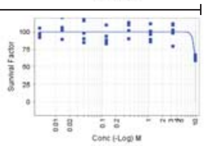

SB-431542-A	BDP-00006337	<	5	<5	0	100	-14.076	-1.2851	+DMSO	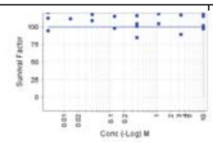	<	5	<5	0	100	-570.85	-1.0537	+LIMK	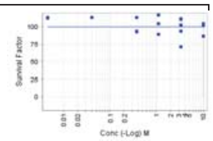
SKF-86055	BDP-00006338	<	5	<5	0	100	-3.1279	-0.0791	+DMSO	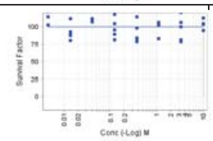	>	8.3372	>8.3372	0	100	1.7689	0.0266	+LIMK	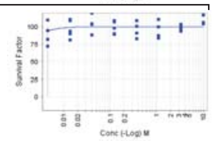
GR269666A	BDP-00006342	<	5	<5	0	100	-114.16	-0.3366	+DMSO	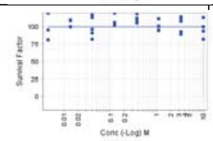	<	5	<5	0	100	-2.8179	-0.223	+LIMK	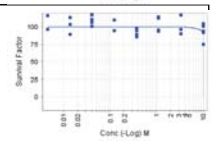
GW282449A	BDP-00006343	<	5	<5	0	100	-12.018	0.0773	+DMSO	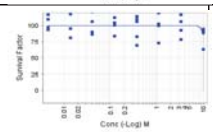	<	5	<5	0	100	-8.1945	-0.3032	+LIMK	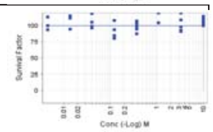
GW282974X	BDP-00006344	<	5	<5	0	100	-40.946	-0.1199	+DMSO	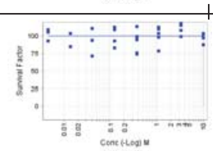	<	5	<5	0	100	-12.442	-0.5249	+LIMK	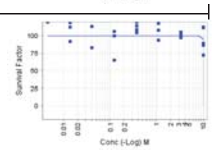
GW301888X	BDP-00006345	<	5	<5	0	100	-11.017	-0.2018	+DMSO	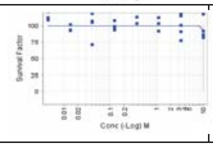	<	5	<5	0	100	-2.767	0.4983	+LIMK	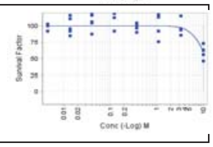

GW632580X	BDP-00006346	<	5	<5	0	100	-1.3227	0.2119	+DMSO	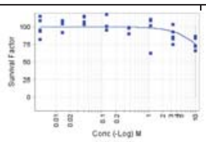	<	5	<5	0	100	-3.2917	0.4688	+LIMK	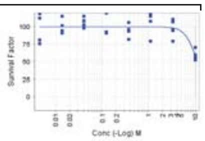
GW820759X	BDP-00006347	<	5	<5	0	100	-363.58	-0.414	+DMSO	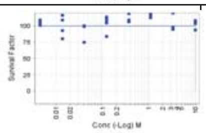	<	5	<5	0	100	-1.3201	0.0042	+LIMK	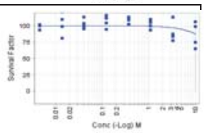
GW782612X	BDP-00006350	<	5	<5	0	100	-1.9043	0.4307	+DMSO	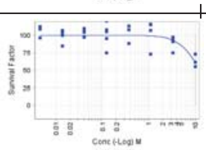	=	5.0623	8.663061	0	100	-3.6473	0.74	+LIMK	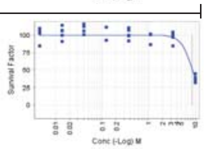
GW642125X	BDP-00006351	<	5	<5	0	100	-14.864	-0.2796	+DMSO	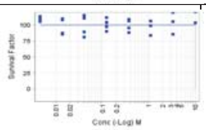	>	8.3372	>8.3372	0	100	3.5021	-0.4977	+LIMK	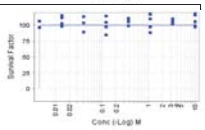
GW642138X	BDP-00006352	<	5	<5	0	100	-10.409	-0.324	+DMSO	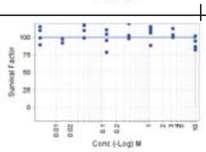	<	5	<5	0	100	-15.2	-1.6304	+LIMK	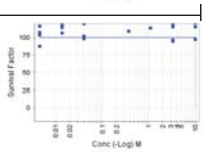
GW513184X	BDP-00006353	<	5	<5	0	100	-11.14	-0.8745	+DMSO	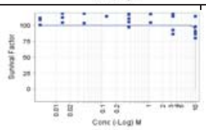	<	5	<5	0	100	-14.164	0.3055	+LIMK	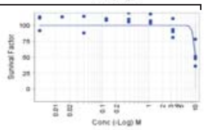

GW578748X	BDP-00006354	<	5	<5	0	100	-9.8927	-1.0689	+DMSO	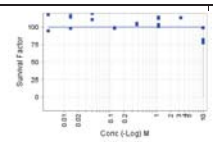	<	5	<5	0	100	-11.855	-0.417	+LIMK	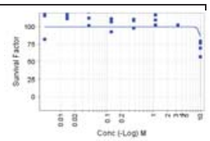
GW643971X	BDP-00006355	<	5	<5	0	100	-10.425	-0.0458	+DMSO	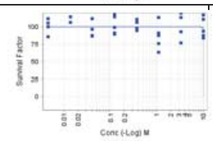	>	8.3372	>8.3372	0	100	0.0657	0.0001	+LIMK	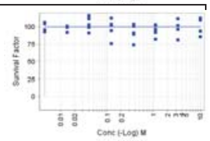
GW794607X	BDP-00006358	<	5	<5	0	100	-11.384	-0.1587	+DMSO	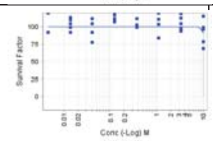	>	8.3372	>8.3372	0	100	2.6688	-0.3228	+LIMK	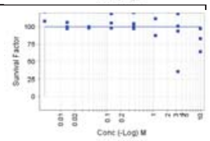
GW809885X	BDP-00006359	<	5	<5	0	100	-110.35	-0.3026	+DMSO	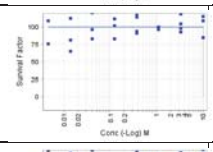	<	5	<5	0	100	-22.854	-1.041	+LIMK	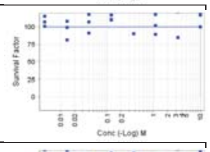
GW811168X	BDP-00006360	<	5	<5	0	100	-11.797	-2.5371	+DMSO	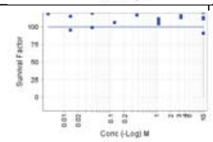	<	5	<5	0	100	-250.24	-0.5878	+LIMK	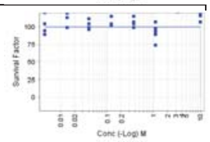
GW817394X	BDP-00006361	<	5	<5	0	100	-602.85	-0.4341	+DMSO	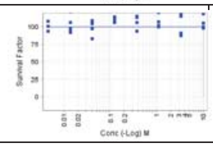	<	5	<5	0	100	-2.8746	0.0136	+LIMK	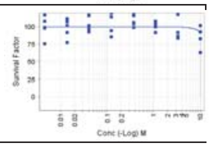

GW817396X	BDP-00006362	<	5	<5	0	100	-127.06	-0.3773	+DMSO	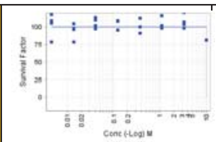	<	5	<5	0	100	-351.96	-1.1687	+LIMK	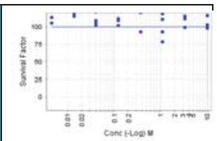
GW829874X	BDP-00006363	>	8.3372	>8.3372	0	100	2.4	-0.103	+DMSO	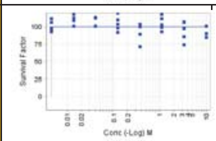	<	5	<5	0	100	-2.8192	-0.0891	+LIMK	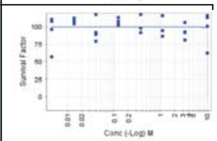
GW829877X	BDP-00006364	<	5	<5	0	100	-11.087	-0.1809	+DMSO	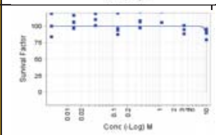	<	5	<5	0	100	-10.872	-0.0451	+LIMK	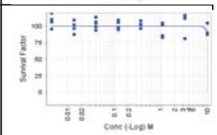
SB-772077-B	BDP-00006365	<	5	<5	0	100	-1.0921	0.2674	+DMSO	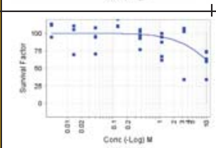	<	5	<5	0	100	-3.3794	0.2939	+LIMK	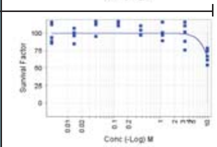
GW801372X	BDP-00006366	<	5	<5	0	100	-6.0427	-0.6452	+DMSO	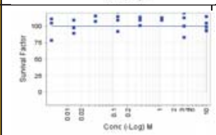	<	5	<5	0	100	-1.4624	-0.1188	+LIMK	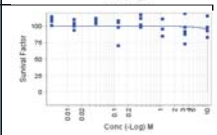
GW801372X	BDP-00006366	=	8.1644	0.006848	0	100	-1.1388	0.7215	+DMSO	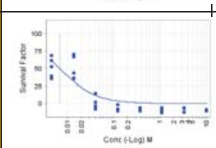	>	8.3372	>8.3372	0	100	-0.6224	0.2484	+LIMK	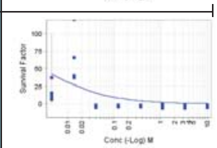

GW807982X	BDP-00006368	<	5	<5	0	100	-1.3312	-0.0378	+DMSO	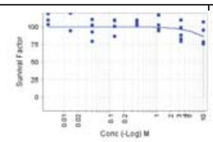	<	5	<5	0	100	-11.999	0.009	+LIMK	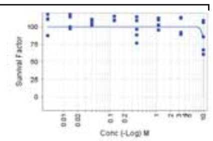
GW810576X	BDP-00006370	<	5	<5	0	100	27.616	-15.781	+DMSO	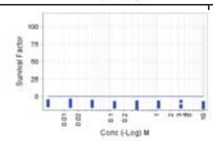	<	5	<5	0	100	4.9582	-5.5505	+LIMK	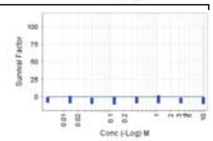
GW811761X	BDP-00006371	>	8.3372	>8.3372	0	100	2.0937	-0.5323	+DMSO	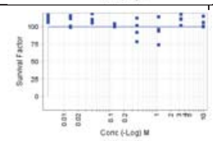	<	5	<5	0	100	-10.658	-0.2374	+LIMK	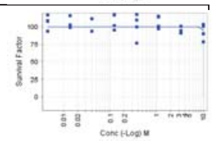
GW819077X	BDP-00006372	<	5	<5	0	100	-2.5623	-0.9282	+DMSO	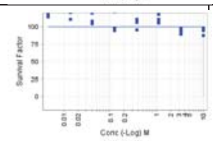	<	5	<5	0	100	-61.987	-0.6348	+LIMK	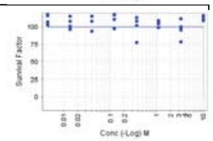
GW827099X	BDP-00006373	<	5	<5	0	100	-7.2642	-0.6929	+DMSO	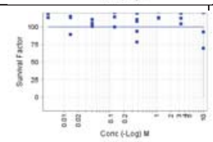	<	5	<5	0	100	-11.988	-0.349	+LIMK	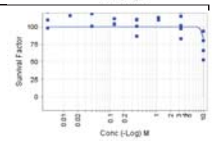
GW827102X	BDP-00006374	<	5	<5	0	100	-8.7561	0.0534	+DMSO	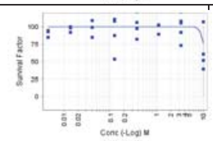	=	5.1133	7.703917	0	100	-1.6801	0.5838	+LIMK	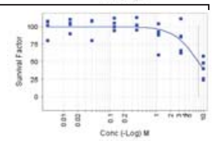

GW827105X	BDP-00006375	<	5	<5	0	100	-30.785	-0.8399	+DMSO	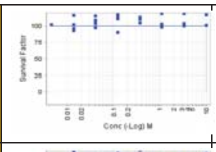	<	5	<5	0	100	-576.81	-0.4647	+LIMK	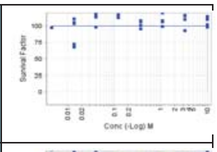
GW827396X	BDP-00006377	>	8.3372	>8.3372	0	100	4.3323	-1.8627	+DMSO	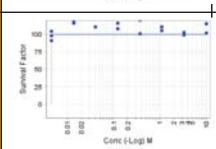	>	8.3372	>8.3372	0	100	1.6899	-0.3475	+LIMK	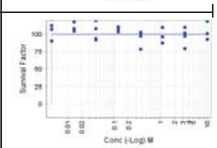
GW828525X	BDP-00006378	<	5	<5	0	100	-11.666	-0.6583	+DMSO	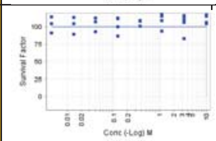	<	5	<5	0	100	-488.15	-0.4784	+LIMK	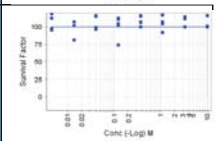
GW828529X	BDP-00006379	<	5	<5	0	100	-5.3261	-1.6453	+DMSO	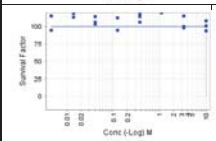	<	5	<5	0	100	-269.66	-0.3401	+LIMK	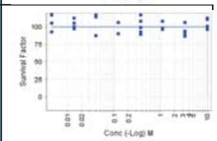
GW829055X	BDP-00006380	<	5	<5	0	100	-40.924	-1.1517	+DMSO	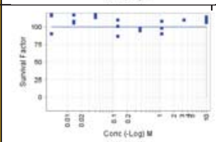	<	5	<5	0	100	-624.47	-1.1415	+LIMK	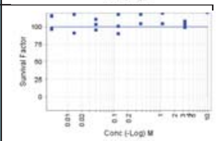
GW829115X	BDP-00006381	<	5	<5	0	100	-1.0403	-0.0691	+DMSO	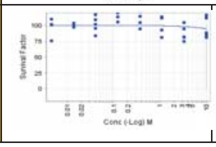	<	5	<5	0	100	-38.949	-0.2669	+LIMK	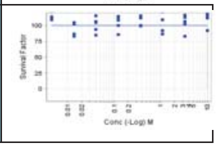

GW832467X	BDP-00006383	<	5	<5	0	100	-356.56	-0.4215	+DMSO	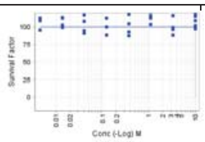	<	5	<5	0	100	-13.515	0.2399	+LIMK	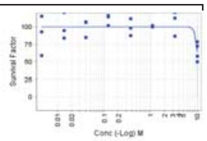
GW833373X	BDP-00006384	<	5	<5	0	100	-34.843	-1.5868	+DMSO	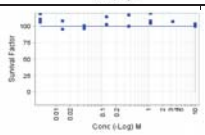	<	5	<5	0	100	-14.423	-0.8641	+LIMK	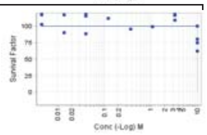
GW284372X	BDP-00006385	<	5	<5	0	100	-11.867	-0.0111	+DMSO	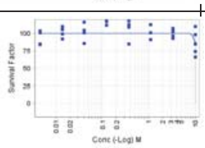	=	5.0743	8.42814	0	100	-4.2051	0.6193	+LIMK	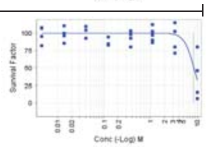
GW458787A	BDP-00006386	<	5	<5	0	100	-2.5188	-0.0198	+DMSO	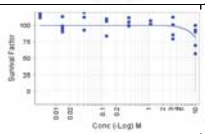	<	5	<5	0	100	-1.5248	0.5342	+LIMK	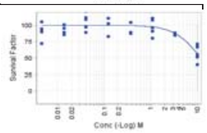
GW566221A	BDP-00006387	<	5	<5	0	100	-5.2971	-0.243	+DMSO	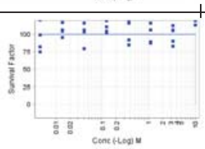	<	5	<5	0	100	-4.1931	-0.0951	+LIMK	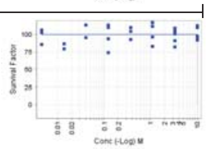
GW567808A	BDP-00006388	>	8.3372	>8.3372	0	100	1.2252	0.0065	+DMSO	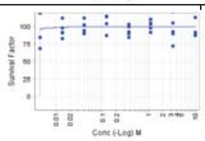	<	5	<5	0	100	-0.8885	0.1122	+LIMK	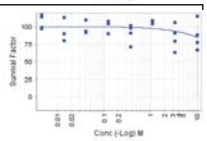

GW568377A	BDP-00006389	>	8.3372	>8.3372	0	100	2.8049	-0.452	+DMSO	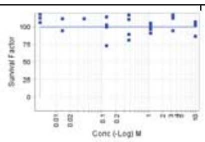	<	5	<5	0	100	-1.098	0.3224	+LIMK	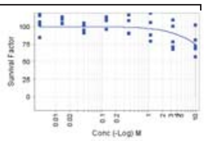
GW574782A	BDP-00006390	<	5	<5	0	100	-237.66	-0.5041	+DMSO	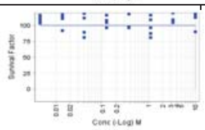	<	5	<5	0	100	-2.0841	-0.2669	+LIMK	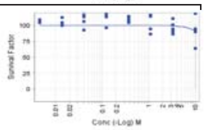
GW576609A	BDP-00006393	<	5	<5	0	100	-0.9397	0.3349	+DMSO	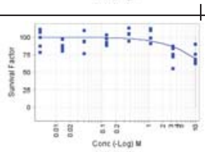	<	5	<5	0	100	-0.6375	0.2616	+LIMK	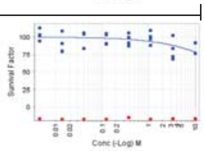
GW576924A	BDP-00006394	>	8.3372	>8.3372	0	100	2.5169	-0.3128	+DMSO	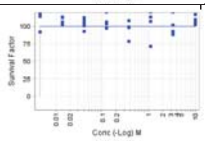	<	5	<5	0	100	-1.8735	-0.1116	+LIMK	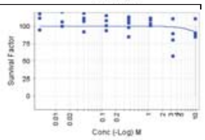
GW580496A	BDP-00006395	<	5	<5	0	100	-12.248	-0.0075	+DMSO	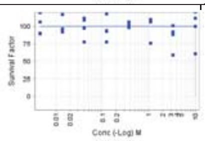	<	5	<5	0	100	-2.3235	0.1451	+LIMK	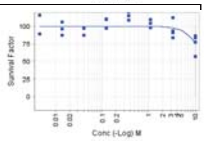
GW583373A	BDP-00006396	<	5	<5	0	100	-34.387	-0.0551	+DMSO	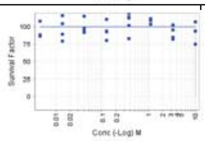	<	5	<5	0	100	-12.388	0.1243	+LIMK	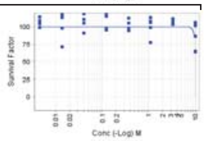

GW615311X	BDP-00006397	<	5	<5	0	100	-1.4412	0.1916	+DMSO	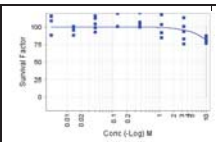	<	5	<5	0	100	-4.7589	0.4595	+LIMK	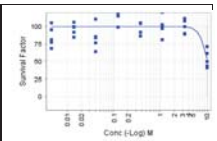
GW616030X	BDP-00006398	<	5	<5	0	100	-1.1524	0.0316	+DMSO	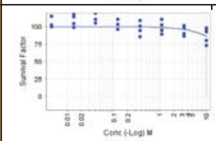	<	5	<5	0	100	-0.7808	0.3479	+LIMK	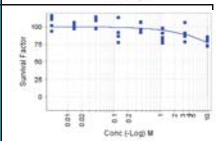
GW621823A	BDP-00006399	<	5	<5	0	100	-0.8227	0.2812	+DMSO	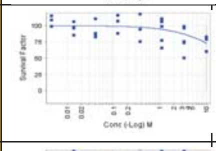	<	5	<5	0	100	-0.4161	0.3879	+LIMK	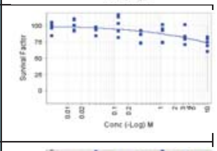
GW633459A	BDP-00006400	<	5	<5	0	100	-11.303	-0.0684	+DMSO	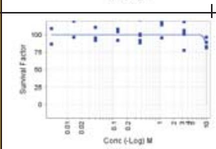	<	5	<5	0	100	-11.266	0.0175	+LIMK	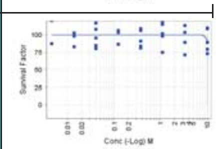
GSK2219385A	BDP-00006404	<	5	<5	0	100	-1.5903	0.6002	+DMSO	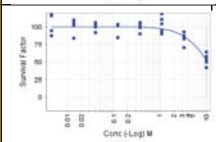	=	5.5622	2.740162	0	100	-0.6858	0.593	+LIMK	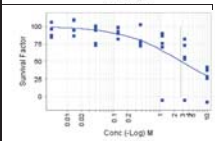
GSK1819799A	BDP-00006406	>	8.3372	>8.3372	0	100	3.3006	-0.611	+DMSO	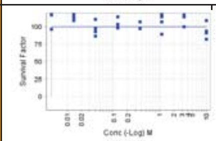	<	5	<5	0	100	-10.437	-0.122	+LIMK	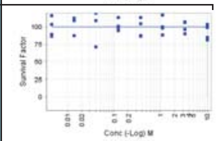

SB-437013	BDP-00006411	<	5	<5	0	100	-405.53	-0.0812	+DMSO	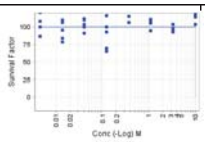	>	8.3372	>8.3372	0	100	1.6325	0.0661	+LIMK	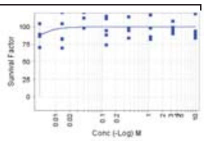
SB-630812	BDP-00006412	<	5	<5	0	100	-11.48	-0.1136	+DMSO	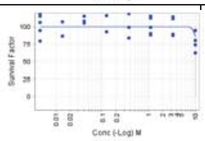	<	5	<5	0	100	-12.712	-0.0004	+LIMK	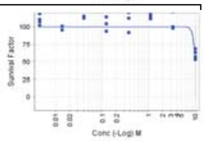
SB-633825	BDP-00006413	<	5	<5	0	100	-25.548	-0.3934	+DMSO	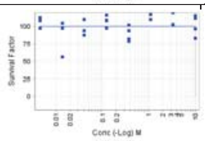	<	5	<5	0	100	-16.449	-0.4811	+LIMK	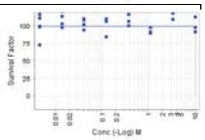
SB-633825	BDP-00006413	<	5	<5	0	100	-11.596	-0.9236	+DMSO	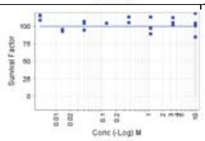	=	5.0249	9.442182	0	100	-1.9958	0.5516	+LIMK	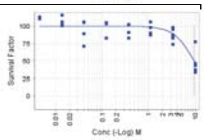
GW768505A	BDP-00006414	<	5	<5	0	100	-12.434	-0.4106	+DMSO	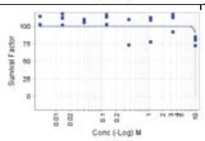	<	5	<5	0	100	-12.41	-0.0423	+LIMK	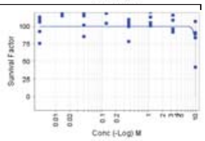
GW768505A	BDP-00006414	<	5	<5	0	100	-2.4918	0.1946	+DMSO	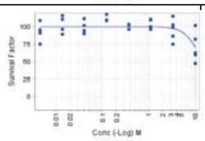	=	5.4766	3.337568	0	100	-27.763	0.7627	+LIMK	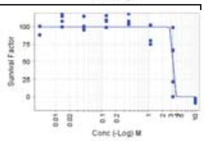

GW795486X	BDP-00006416	<	5	<5	0	100	-1.5819	-0.0095	+DMSO	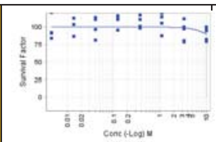	<	5	<5	0	100	-1.178	0.0642	+LIMK	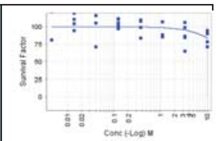
GW795493X	BDP-00006417	<	5	<5	0	100	-408.09	-0.1957	+DMSO	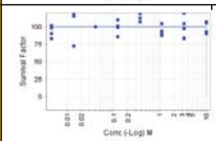	<	5	<5	0	100	-0.5537	0.156	+LIMK	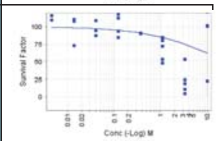
GW275944X	BDP-00006418	<	5	<5	0	100	-2.8267	0.067	+DMSO	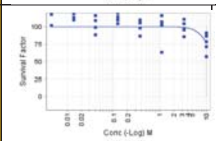	<	5	<5	0	100	-0.6529	0.378	+LIMK	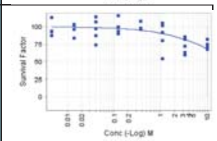
GW276655X	BDP-00006419	<	5	<5	0	100	-1.2163	-0.1417	+DMSO	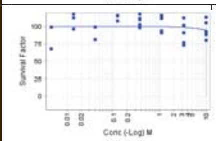	=	5.0059	9.865903	0	100	-17.005	0.4428	+LIMK	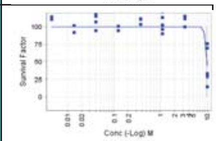
GW279320X	BDP-00006420	<	5	<5	0	100	-5.776	-0.1958	+DMSO	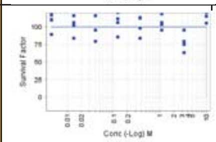	<	5	<5	0	100	-366.15	-0.3747	+LIMK	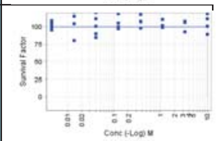
GW280670X	BDP-00006421	>	8.3372	>8.3372	0	100	0.2231	0.0132	+DMSO	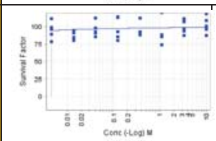	<	5	<5	0	100	-1.3832	0.131	+LIMK	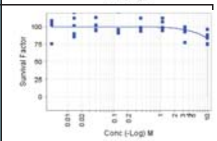

GW282536X	BDP-00006422	<	5	<5	0	100	-41.815	-0.1366	+DMSO	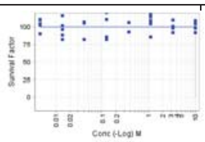	<	5	<5	0	100	-16.853	-0.1545	+LIMK	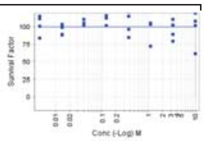
GW290597X	BDP-00006423	<	5	<5	0	100	-1.9106	-0.2824	+DMSO	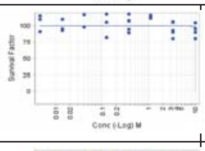	<	5	<5	0	100	-10.04	0.0026	+LIMK	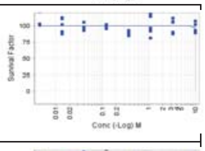
GW297361X	BDP-00006424	<	5	<5	0	100	-0.6082	0.2837	+DMSO	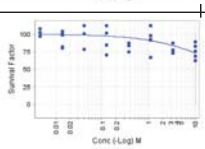	<	5	<5	0	100	-0.4628	0.4856	+LIMK	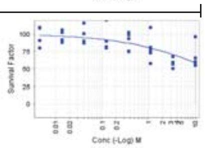
GW300653X	BDP-00006425	<	5	<5	0	100	-326.83	-0.5392	+DMSO	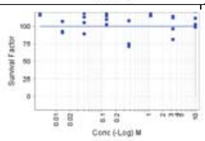	<	5	<5	0	100	-68.813	-0.1363	+LIMK	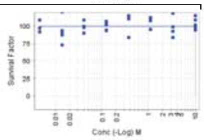
GW300657X	BDP-00006426	<	5	<5	0	100	-0.8939	0.0389	+DMSO	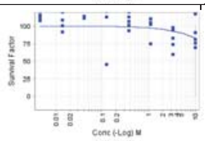	<	5	<5	0	100	-0.834	0.1011	+LIMK	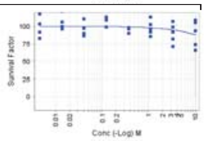
GW300660X	BDP-00006427	<	5	<5	0	100	-3.9035	-0.2825	+DMSO	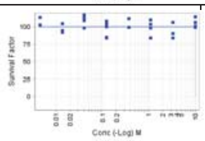	<	5	<5	0	100	-14.239	-0.8098	+LIMK	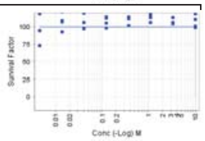

GW301784X	BDP-00006428	<	5	<5	0	100	-10.926	0.0107	+DMSO	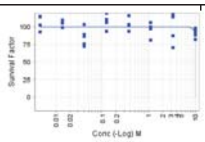	<	5	<5	0	100	-1.9255	0.5257	+LIMK	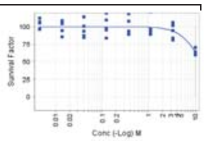
GW305178X	BDP-00006429	<	5	<5	0	100	-0.183	0.2263	+DMSO	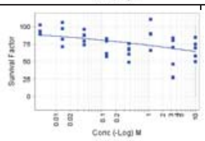	=	5.5093	3.095584	0	100	-0.3424	0.3388	+LIMK	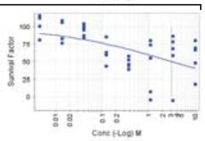
GW335962X	BDP-00006430	<	5	<5	0	100	-11.515	0.0547	+DMSO	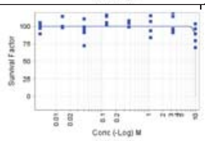	<	5	<5	0	100	-11.288	0.1125	+LIMK	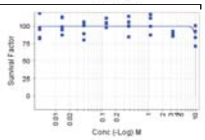
GW352430A	BDP-00006431	<	5	<5	0	100	-26.767	-0.37	+DMSO	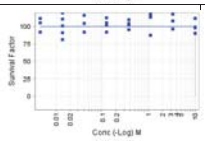	<	5	<5	0	100	-4.9194	-0.0124	+LIMK	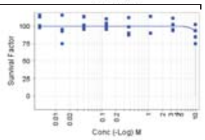
GW396574X	BDP-00006432	<	5	<5	0	100	-0.2618	0.1895	+DMSO	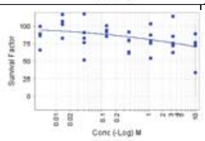	=	5.1942	6.393848	0	100	-0.248	0.3819	+LIMK	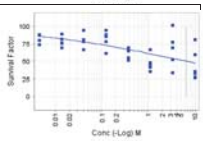
GW416469X	BDP-00006433	<	5	<5	0	100	-67.787	-0.2835	+DMSO	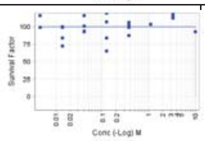	<	5	<5	0	100	-15.113	-0.669	+LIMK	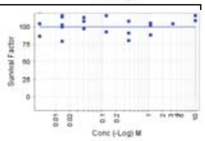

SB-242717	BDP-00006436	<	5	<5	0	100	-1.8649	0.2609	+DMSO	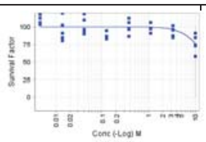	<	5	<5	0	100	-14.634	0.4514	+LIMK	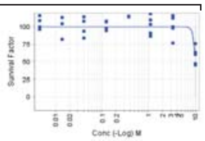
SB-242718	BDP-00006437	<	5	<5	0	100	-10.726	-0.129	+DMSO	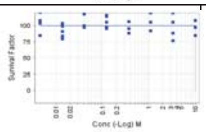	<	5	<5	0	100	-11.616	-1.1137	+LIMK	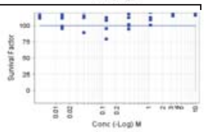
SB-242721	BDP-00006439	<	5	<5	0	100	-10.822	-0.2102	+DMSO	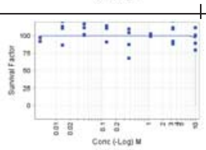	>	8.3372	>8.3372	0	100	10.676	-0.1775	+LIMK	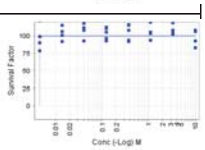
SB-251527	BDP-00006443	<	5	<5	0	100	-12.763	0.3491	+DMSO	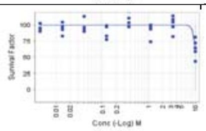	=	5.0627	8.655472	0	100	-4.9086	0.7783	+LIMK	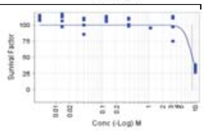
SB-253228	BDP-00006445	<	5	<5	0	100	-13.476	-0.5693	+DMSO	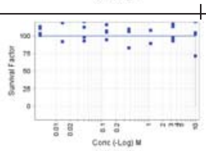	<	5	<5	0	100	-10.442	-0.7109	+LIMK	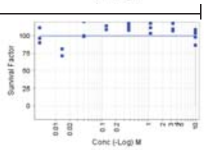
SB-253228	BDP-00006445	<	5	<5	0	100	-1.2028	0.3682	+DMSO	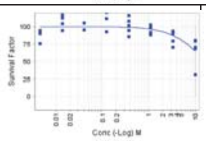	=	5.2189	6.041413	0	100	-3.0335	0.8055	+LIMK	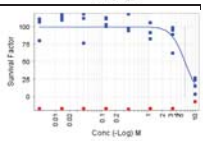

SB-254169	BDP-00006446	<	5	<5	0	100	-3.1989	-0.2307	+DMSO	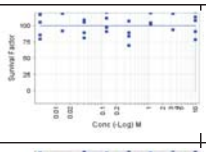	<	5	<5	0	100	-10.356	-0.2909	+LIMK	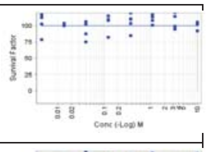
SB-264865	BDP-00006447	<	5	<5	0	100	-2.6479	-0.3003	+DMSO	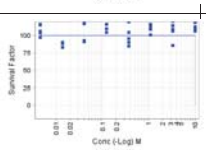	>	8.3372	>8.3372	0	100	0.4107	0.0209	+LIMK	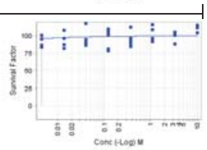
SB-264866	BDP-00006448	<	5	<5	0	100	-0.8646	0.0124	+DMSO	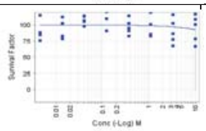	<	5	<5	0	100	-2.3825	-0.2186	+LIMK	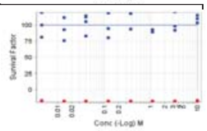
SB-278539	BDP-00006450	<	5	<5	0	100	-12.058	0.0154	+DMSO	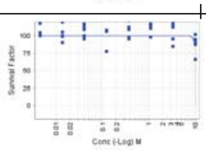	<	5	<5	0	100	-11.141	0.0144	+LIMK	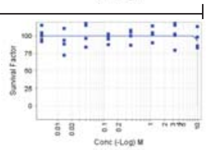
SB-278539	BDP-00006450	<	5	<5	0	100	-3.4354	0.3294	+DMSO	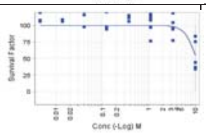	=	5.454	3.515766	0	100	-18.755	0.8248	+LIMK	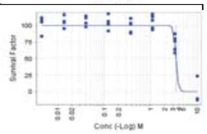
SB-285234-W	BDP-00006452	>	8.3372	>8.3372	0	100	1.2419	-0.1172	+DMSO	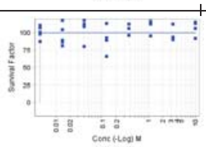	<	5	<5	0	100	-28.976	-0.7352	+LIMK	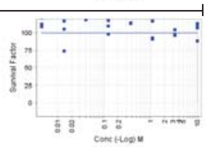

GSK317354A	BDP-00006453	<	5	<5	0	100	-10.839	-0.4222	+DMSO	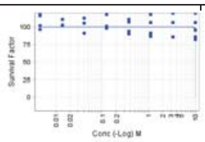	>	8.3372	>8.3372	0	100	0.5811	-0.0078	+LIMK	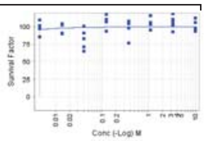
GW561436X	BDP-00006454	<	5	<5	0	100	-177.11	-0.267	+DMSO	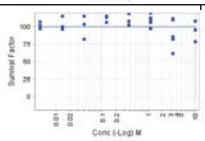	<	5	<5	0	100	-280.44	-0.8115	+LIMK	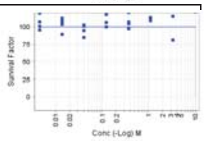
GW568326X	BDP-00006455	<	5	<5	0	100	-3.6548	-0.1218	+DMSO	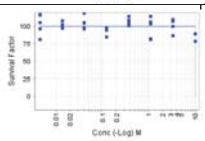	<	5	<5	0	100	-9.6165	-0.6606	+LIMK	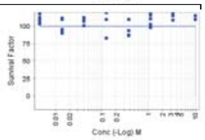
GW569293E	BDP-00006456	<	5	<5	0	100	-7.3786	-0.13	+DMSO	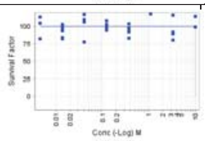	<	5	<5	0	100	-12.353	0.3551	+LIMK	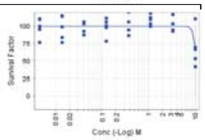
GW581744X	BDP-00006457	<	5	<5	0	100	-12.72	-1.053	+DMSO	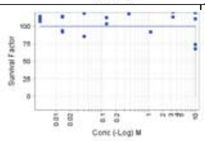	>	8.3372	>8.3372	0	100	2.5539	-1.297	+LIMK	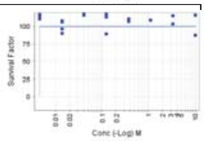
SB-210313	BDP-00006459	<	5	<5	0	100	-0.556	-0.0028	+DMSO	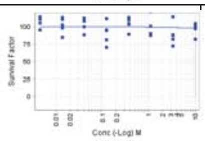	<	5	<5	0	100	-1.427	-0.018	+LIMK	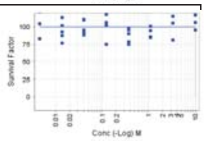

SB-216385	BDP-00006460	<	5	<5	0	100	-11.898	-0.0377	+DMSO	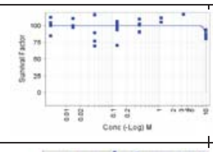	=	5.0071	9.836843	0	100	-16.581	0.6067	+LIMK	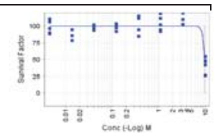
SKF-86002-A2	BDP-00006461	<	5	<5	0	100	-2.0507	-0.0613	+DMSO	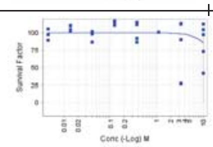	<	5	<5	0	100	-449.23	-0.3992	+LIMK	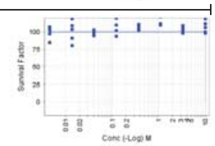
SB-223133	BDP-00006465	<	5	<5	0	100	-318.25	-0.2229	+DMSO	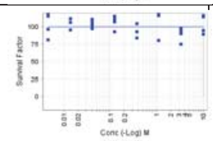	=	5.2752	5.306538	0	100	-3.6444	0.6522	+LIMK	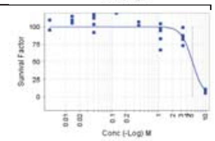
GSK200398A	BDP-00006468	<	5	<5	0	100	-20.323	0.6333	+DMSO	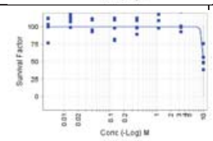	=	5.042	9.07858	0	100	-4.6633	0.7505	+LIMK	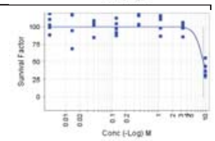
GSK238583A	BDP-00006469	<	5	<5	0	100	-1.1365	0.0505	+DMSO	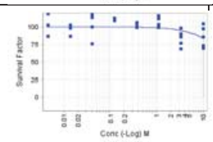	<	5	<5	0	100	-1.0713	0.5648	+LIMK	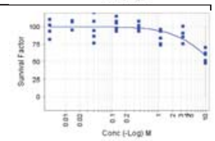
GSK259178A	BDP-00006470	<	5	<5	0	100	-10.795	-0.238	+DMSO	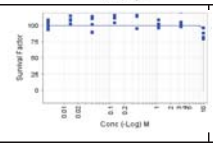	<	5	<5	0	100	-2.6966	0.4655	+LIMK	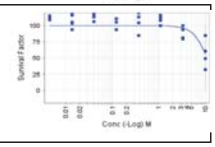

GSK259178A	BDP-00006470	<	5	<5	0	100	-1.6353	-0.0315	+DMSO	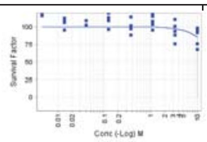	=	5.1717	6.73483	0	100	-1.7976	0.6456	+LIMK	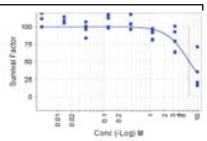
GW784752X	BDP-00006472	<	5	<5	0	100	-318.51	-1.2511	+DMSO	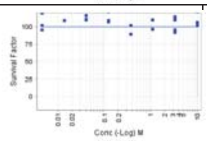	<	5	<5	0	100	-289.88	-0.6253	+LIMK	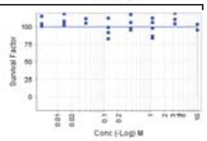
GW813360X	BDP-00006473	<	5	<5	0	100	-13.023	-0.2309	+DMSO	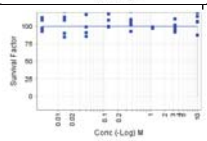	=	5.1278	7.450247	0	100	-2.3017	0.6127	+LIMK	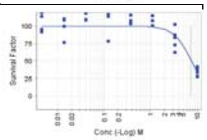
GW683003X	BDP-00006475	<	5	<5	0	100	-251.97	-0.2689	+DMSO	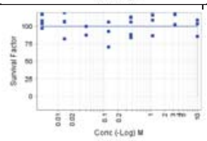	<	5	<5	0	100	-221.95	-0.6601	+LIMK	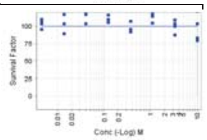
GW683109X	BDP-00006476	<	5	<5	0	100	-0.5718	0.1336	+DMSO	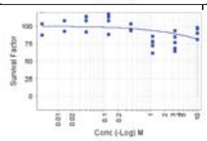	<	5	<5	0	100	-0.6634	0.2059	+LIMK	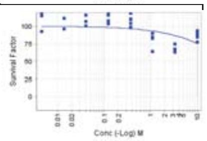
GW683768X	BDP-00006477	>	8.3372	>8.3372	0	100	-10.274	-0.3912	+DMSO	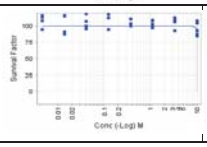	<	5	<5	0	100	-0.6579	0.1293	+LIMK	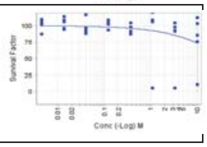

GW708336X	BDP-00006478	<	5	<5	0	100	-1.2415	0.0896	+DMSO	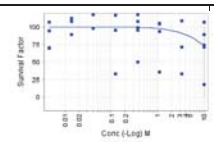	<	5	<5	0	100	-12.665	0.4428	+LIMK	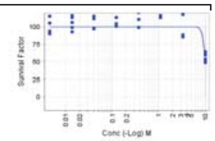
GW778894X	BDP-00006479	<	5	<5	0	100	26.727	-0.4195	+DMSO	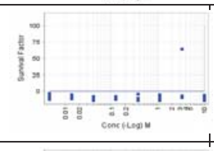	<	5	<5	0	100	4.5669	-2.8858	+LIMK	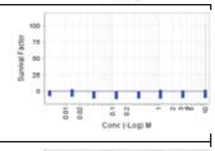
GW779439X	BDP-00006480	<	5	<5	0	100	380.98	-49.555	+DMSO	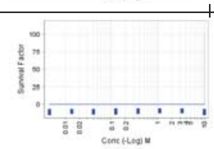	<	5	<5	0	100	4.798	-7.3225	+LIMK	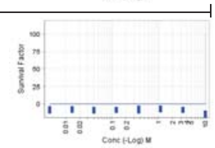
GW780056X	BDP-00006481	<	5	<5	0	100	4.3988	-12.488	+DMSO	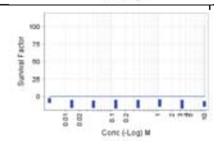	<	5	<5	0	100	56.696	-7.8628	+LIMK	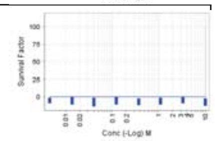
GW781673X	BDP-00006482	<	5	<5	0	100	-1.0329	0.303	+DMSO	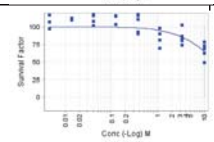	=	5.2858	5.178924	0	100	-1.6344	0.7171	+LIMK	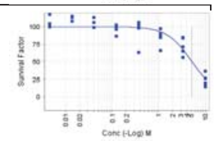
GI261520A	BDP-00006484	<	5	<5	0	100	-0.5599	0.1572	+DMSO	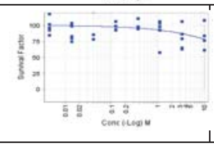	<	5	<5	0	100	-0.7486	0.5487	+LIMK	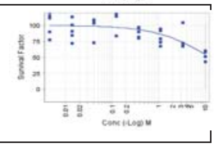

GW305074X	BDP-00006486	<	5	<5	0	100	-227.75	-1.3898	+DMSO	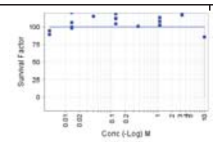	<	5	<5	0	100	-9.7162	-0.5103	+LIMK	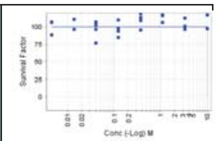
GW405841X	BDP-00006487	<	5	<5	0	100	-259.08	-0.4558	+DMSO	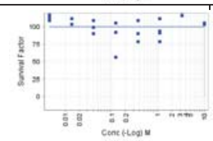	<	5	<5	0	100	-445.53	-0.6029	+LIMK	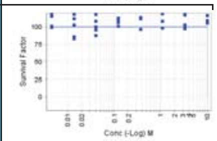
GW407323A	BDP-00006489	<	5	<5	0	100	-26.783	-1.3003	+DMSO	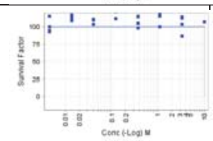	<	5	<5	0	100	-234.57	-0.0395	+LIMK	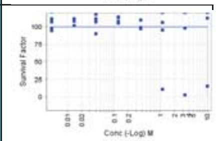
GW429374A	BDP-00006490	>	8.3372	>8.3372	0	100	4.016	-2.1815	+DMSO	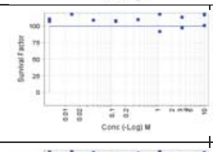	<	5	<5	0	100	-13.7	-1.5741	+LIMK	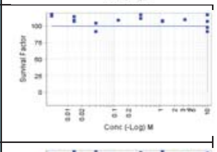
SB-476429-A	BDP-00006493	<	5	<5	0	100	-10.611	-0.5689	+DMSO	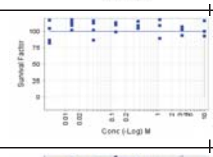	<	5	<5	0	100	-57.932	-1.2999	+LIMK	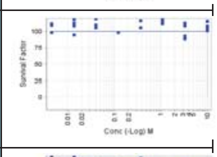
SB-610251-B	BDP-00006494	<	5	<5	0	100	-18.801	-0.6817	+DMSO	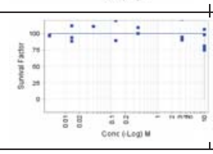	=	5.0116	9.736795	0	100	-17.693	0.4844	+LIMK	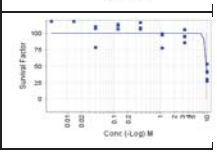

SB-614067-R	BDP-00006496	>	8.3372	>8.3372	0	100	2.8751	-0.0063	+DMSO	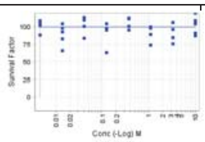	<	5	<5	0	100	-561.01	-0.1695	+LIMK	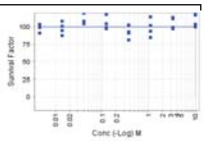
GW434756X	BDP-00006498	<	5	<5	0	100	-14.003	-1.4217	+DMSO	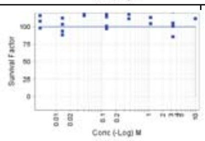	<	5	<5	0	100	-3.7542	-0.0132	+LIMK	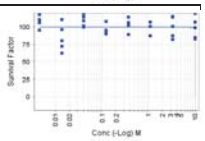
GSK182497A	BDP-00006499	<	5	<5	0	100	-100.1	-0.2625	+DMSO	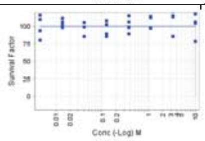	<	5	<5	0	100	-296.93	-0.1939	+LIMK	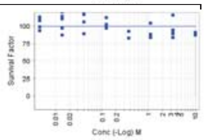
GSK192082A	BDP-00006500	<	5	<5	0	100	-1.3583	0.4804	+DMSO	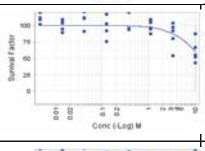	=	5.2557	5.550494	0	100	-2.2964	0.7014	+LIMK	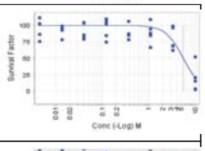
GSK300014A	BDP-00006502	<	5	<5	0	100	-29.046	-0.191	+DMSO	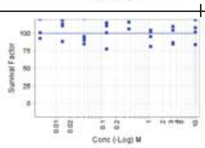	<	5	<5	0	100	-13.096	0.3512	+LIMK	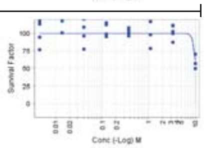
GSK969786A	BDP-00006503	>	8.3372	>8.3372	0	100	4.3614	-0.1302	+DMSO	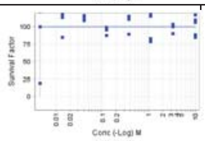	<	5	<5	0	100	-157.6	-1.6155	+LIMK	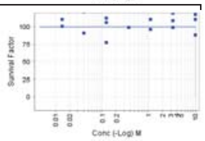

GW684626B	BDP-00006504	<	5	<5	0	100	-10.943	-0.1839	+DMSO	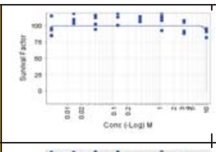	<	5	<5	0	100	-11.694	-0.137	+LIMK	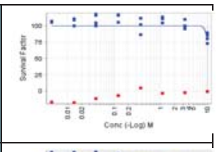
GW693881A	BDP-00006505	<	5	<5	0	100	-2.0369	0.4205	+DMSO	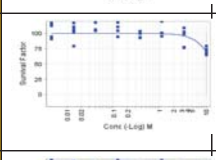	=	5.3225	4.758769	0	100	-4.4042	0.8801	+LIMK	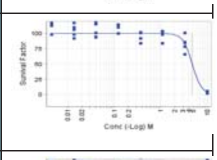
GW296115X	BDP-00006507	<	5	<5	0	100	-11.438	-0.9196	+DMSO	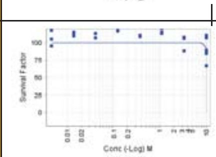	<	5	<5	0	100	-1.9485	-0.7199	+LIMK	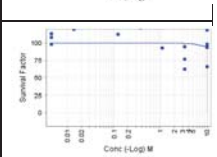
