# Supplementary material for: LIM kinase inhibitors disrupt mitotic microtubule organization and impair tumor cell proliferation
Source: Oncotarget. 2015 Nov 3;6(36):38469–86. doi: 10.18632/oncotarget.6288 (PMC4770715; doi:10.18632/oncotarget.6288)
Supplement: Supplementary file 6 [file oncotarget-06-38469-s006.pdf]

Supplemental Table 5. Physiochemical properties of CRT0105446 and CRT0105950.

Property	CRT0105446	CRT0105950
MW	421	393
cLogP	4.3	4.4
LIMK1 IC_{50}	8	0.3
LIMK2 IC_{50}	32	1
Microsome stability	Mouse 51%<br>Human 34%	Mouse 50%<br>Human 9%
Cytochrome p450	3A4, 2D6 1-10 µM<br>2C9, 2C19,1A2 >10 µM	3A4, 2D6 1-10 µM<br>2C9, 2C19,1A2 >10 µM
Mechanism of Action	ATP-competitive	ATP-competitive
