# Supplementary material for: LIM kinase inhibitors disrupt mitotic microtubule organization and impair tumor cell proliferation
Source: Oncotarget. 2015 Nov 3;6(36):38469–86. doi: 10.18632/oncotarget.6288 (PMC4770715; doi:10.18632/oncotarget.6288)
Supplement: Supplementary file 7 [file oncotarget-06-38469-s007.pdf]

Compound Concentration (μM)
10

Data shown as Percent Control
100% = no compound has competed
50-25% | 24-0%

KinaseScan Gene Symbol	Entrez Gene Symbol	CRT0105446 @ 10uM	CRT0105950 @ 10uM
AAK1	AAK1	78	51
ABL1(E255K)-phosphorylated	ABL1	78	79
ABL1(F317I)-nonphosphorylated	ABL1	100	93
ABL1(F317I)-phosphorylated	ABL1	76	87
ABL1(F317L)-nonphosphorylated	ABL1	92	86
ABL1(F317L)-phosphorylated	ABL1	89	67
ABL1(H396P)-nonphosphorylated	ABL1	71	27
ABL1(H396P)-phosphorylated	ABL1	82	72
ABL1(M351T)-phosphorylated	ABL1	83	66
ABL1(Q252H)-nonphosphorylated	ABL1	83	70
ABL1(Q252H)-phosphorylated	ABL1	81	74
ABL1(T315I)-nonphosphorylated	ABL1	100	82
ABL1(T315I)-phosphorylated	ABL1	99	79
ABL1(Y253F)-phosphorylated	ABL1	89	74
ABL1-nonphosphorylated	ABL1	82	43
ABL1-phosphorylated	ABL1	77	70
ABL2	ABL2	100	58
ACVR1	ACVR1	100	52
ACVR1B	ACVR1B	82	76
ACVR2A	ACVR2A	74	45
ACVR2B	ACVR2B	100	32
ACVRL1	ACVRL1	100	100
ADCK3	CABC1	98	56
ADCK4	ADCK4	85	58
AKT1	AKT1	100	100
AKT2	AKT2	100	100
AKT3	AKT3	100	100
ALK	ALK	89	20
AMPK-alpha1	PRKAA1	1.1	2.1
AMPK-alpha2	PRKAA2	3.9	2
ANKK1	ANKK1	99	69
ARK5	NUAK1	59	49
ASK1	MAP3K5	100	100
ASK2	MAP3K6	100	100

Screening Concentration (10 μM)
Number of Non-Mutant Kinases used in the screen 386

Compound Name	Selectivity Score Type	Number of Hits	Selectivity Score
**CRT0105446**	S(35)	**32**	0.083
	S(10)	15	0.039
	S(1)	**3**	0.008
**CRT0105950**	S(35)	149	0.386
	S(10)	63	0.163
	S(1)	17	0.044

AURKA	AURKA	100	18
AURKB	AURKB	80	0.15
AURKC	AURKC	88	3.8
AXL	AXL	87	43
BIKE	BMP2K	100	36
BLK	BLK	100	9.2
BMPR1A	BMPR1A	100	91
BMPR1B	BMPR1B	92	29
BMPR2	BMPR2	100	8.9
BMX	BMX	100	81
BRAF	BRAF	79	93
BRAF(V600E)	BRAF	64	83
BRK	PTK6	100	100
BRSK1	BRSK1	27	30
BRSK2	BRSK2	59	33
BTK	BTK	85	72
CAMK1	CAMK1	62	9.4
CAMK1D	CAMK1D	79	63
CAMK1G	CAMK1G	99	57
CAMK2A	CAMK2A	82	33
CAMK2B	CAMK2B	92	42
CAMK2D	CAMK2D	72	29
CAMK2G	CAMK2G	80	49
CAMK4	CAMK4	98	90
CAMKK1	CAMKK1	82	26
CAMKK2	CAMKK2	95	43
CASK	CASK	1.2	13
CDC2L1	CDC2L1	54	71
CDC2L2	CDC2L2	59	89
CDC2L5	CDC2L5	14	26
CDK11	CDC2L6	34	2.3
CDK2	CDK2	0.9	0.2
CDK3	CDK3	24	7.7
CDK4-cyclinD1	CDK4	20	3.7
CDK4-cyclinD3	CDK4	48	13
CDK5	CDK5	60	12
CDK7	CDK7	5.8	32
CDK8	CDK8	39	11
CDK9	CDK9	28	65

CDKL1	CDKL1	80	77
CDKL2	CDKL2	2.2	0.6
CDKL3	CDKL3	26	23
CDKL5	CDKL5	100	99
CHEK1	CHEK1	100	88
CHEK2	CHEK2	100	55
CIT	CIT	37	44
CLK1	CLK1	85	0.4
CLK2	CLK2	78	0.45
CLK3	CLK3	49	22
CLK4	CLK4	100	1.6
CSF1R	CSF1R	51	1.2
CSK	CSK	91	71
CSNK1A1	CSNK1A1	100	88
CSNK1A1L	CSNK1A1L	96	74
CSNK1D	CSNK1D	27	6.1
CSNK1E	CSNK1E	42	11
CSNK1G1	CSNK1G1	94	82
CSNK1G2	CSNK1G2	55	30
CSNK1G3	CSNK1G3	67	43
CSNK2A1	CSNK2A1	95	60
CSNK2A2	CSNK2A2	91	58
CTK	MATK	100	100
DAPK1	DAPK1	100	82
DAPK2	DAPK2	87	53
DAPK3	DAPK3	83	44
DCAMKL1	DCLK1	46	75
DCAMKL2	DCLK2	39	77
DCAMKL3	DCLK3	88	7.4
DDR1	DDR1	83	0.1
DDR2	DDR2	0.4	12
DLK	MAP3K12	100	100
DMPK	DMPK	100	100
DMPK2	CDC42BPG	93	71
DRAK1	STK17A	81	100
DRAK2	STK17B	100	100
DYRK1A	DYRK1A	76	30
DYRK1B	DYRK1B	40	5.6
DYRK2	DYRK2	81	79

EGFR	EGFR	100	53
EGFR(E746-A750del)	EGFR	100	78
EGFR(G719C)	EGFR	89	71
EGFR(G719S)	EGFR	94	75
EGFR(L747-E749del, A750P)	EGFR	98	64
EGFR(L747-S752del, P753S)	EGFR	92	66
EGFR(L747-T751del,Sins)	EGFR	100	68
EGFR(L858R)	EGFR	76	69
EGFR(L858R,T790M)	EGFR	91	89
EGFR(L861Q)	EGFR	100	65
EGFR(S752-I759del)	EGFR	84	69
EGFR(T790M)	EGFR	78	63
EIF2AK1	EIF2AK1	100	97
EPHA1	EPHA1	100	74
EPHA2	EPHA2	100	70
EPHA3	EPHA3	87	71
EPHA4	EPHA4	100	94
EPHA5	EPHA5	100	100
EPHA6	EPHA6	86	30
EPHA7	EPHA7	94	83
EPHA8	EPHA8	100	88
EPHB1	EPHB1	88	80
EPHB2	EPHB2	100	81
EPHB3	EPHB3	83	96
EPHB4	EPHB4	93	82
EPHB6	EPHB6	86	17
ERBB2	ERBB2	94	80
ERBB3	ERBB3	100	100
ERBB4	ERBB4	100	94
ERK1	MAPK3	100	96
ERK2	MAPK1	100	100
ERK3	MAPK6	100	97
ERK4	MAPK4	100	86
ERK5	MAPK7	86	93
ERK8	MAPK15	1.4	1.6
ERN1	ERN1	87	68
FAK	PTK2	100	33
FER	FER	100	57
FES	FES	87	93

FGFR1	FGFR1	98	5
FGFR2	FGFR2	100	17
FGFR3	FGFR3	96	4.8
FGFR3(G697C)	FGFR3	93	7.2
FGFR4	FGFR4	90	29
FGR	FGR	93	32
FLT1	FLT1	80	21
FLT3	FLT3	41	0.55
FLT3(D835H)	FLT3	15	0.95
FLT3(D835Y)	FLT3	20	1.2
FLT3(ITD)	FLT3	79	4.4
FLT3(K663Q)	FLT3	34	0.5
FLT3(N841I)	FLT3	22	0
FLT3(R834Q)	FLT3	88	18
FLT4	FLT4	61	26
FRK	FRK	98	59
FYN	FYN	99	49
GAK	GAK	92	22
GCN2(Kin.Dom.2,S808G)	EIF2AK4	92	94
GRK1	GRK1	70	59
GRK4	GRK4	100	89
GRK7	GRK7	58	16
GSK3A	GSK3A	56	32
GSK3B	GSK3B	82	86
HCK	HCK	100	94
HIPK1	HIPK1	100	65
HIPK2	HIPK2	92	55
HIPK3	HIPK3	98	69
HIPK4	HIPK4	75	16
HPK1	MAP4K1	100	22
HUNK	HUNK	89	49
ICK	ICK	18	19
IGF1R	IGF1R	100	3.6
IKK-alpha	CHUK	89	94
IKK-beta	IKBKB	88	88
IKK-epsilon	IKBKE	85	41
INSR	INSR	100	1
INSRR	INSRR	96	0.15
IRAK1	IRAK1	94	56

IRAK3	IRAK3	89	8.1
IRAK4	IRAK4	100	80
ITK	ITK	100	61
JAK1(JH1domain-catalytic)	JAK1	89	53
JAK1(JH2domain-pseudokinase)	JAK1	92	74
JAK2(JH1domain-catalytic)	JAK2	100	19
JAK3(JH1domain-catalytic)	JAK3	100	33
JNK1	MAPK8	34	40
JNK2	MAPK9	50	30
JNK3	MAPK10	36	25
KIT	KIT	55	3.2
KIT(A829P)	KIT	100	35
KIT(D816H)	KIT	89	46
KIT(D816V)	KIT	83	2.8
KIT(L576P)	KIT	36	0.4
KIT(V559D)	KIT	39	0.75
KIT(V559D,T670I)	KIT	74	36
KIT(V559D,V654A)	KIT	100	11
LATS1	LATS1	91	23
LATS2	LATS2	90	6
LCK	LCK	100	20
LIMK1	LIMK1	2	0.9
LIMK2	LIMK2	1.1	0
LKB1	STK11	100	79
LOK	STK10	100	51
LRRK2	LRRK2	91	54
LRRK2(G2019S)	LRRK2	86	56
LTK	LTK	100	100
LYN	LYN	100	26
LZK	MAP3K13	100	96
MAK	MAK	0.6	2
MAP3K1	MAP3K1	88	71
MAP3K15	MAP3K15	93	82
MAP3K2	MAP3K2	82	15
MAP3K3	MAP3K3	100	20
MAP3K4	MAP3K4	99	100
MAP4K2	MAP4K2	100	81
MAP4K3	MAP4K3	58	18
MAP4K4	MAP4K4	68	11

MAP4K5	MAP4K5	100	32
MAPKAPK2	MAPKAPK2	100	100
MAPKAPK5	MAPKAPK5	95	97
MARK1	MARK1	87	5.8
MARK2	MARK2	63	28
MARK3	MARK3	78	51
MARK4	MARK4	57	4
MAST1	MAST1	68	84
MEK1	MAP2K1	87	51
MEK2	MAP2K2	92	42
MEK3	MAP2K3	83	27
MEK4	MAP2K4	82	1.8
MEK5	MAP2K5	100	60
MEK6	MAP2K6	100	20
MELK	MELK	100	11
MERTK	MERTK	69	0.45
MET	MET	80	16
MET(M1250T)	MET	93	19
MET(Y1235D)	MET	96	14
MINK	MINK1	100	13
MKK7	MAP2K7	97	38
MKNK1	MKNK1	49	46
MKNK2	MKNK2	68	1.8
MLCK	MYLK3	90	14
MLK1	MAP3K9	86	13
MLK2	MAP3K10	69	40
MLK3	MAP3K11	100	43
MRCKA	CDC42BPA	77	84
MRCKB	CDC42BPB	93	78
MST1	STK4	100	3.4
MST1R	MST1R	100	92
MST2	STK3	100	0.7
MST3	STK24	72	40
MST4	MST4	61	37
MTOR	FRAP1	64	35
MUSK	MUSK	96	18
MYLK	MYLK	100	28
MYLK2	MYLK2	49	49
MYLK4	MYLK4	71	9.4

MYO3A	MYO3A	100	65
MYO3B	MYO3B	100	90
NDR1	STK38	100	86
NDR2	STK38L	82	80
NEK1	NEK1	100	81
NEK11	NEK11	100	84
NEK2	NEK2	100	82
NEK3	NEK3	100	88
NEK4	NEK4	91	77
NEK5	NEK5	99	65
NEK6	NEK6	95	100
NEK7	NEK7	100	100
NEK9	NEK9	92	96
NIM1	MGC42105	100	100
NLK	NLK	57	20
OSR1	OXSR1	100	84
p38-alpha	MAPK14	93	87
p38-beta	MAPK11	100	93
p38-delta	MAPK13	97	100
p38-gamma	MAPK12	100	100
PAK1	PAK1	57	97
PAK2	PAK2	80	76
PAK3	PAK3	4.6	4.1
PAK4	PAK4	82	79
PAK6	PAK6	93	38
PAK7	PAK7	95	93
PCTK1	PCTK1	11	63
PCTK2	PCTK2	17	52
PCTK3	PCTK3	7.7	68
PDGFRA	PDGFRA	66	48
PDGFRB	PDGFRB	51	1.6
PDPK1	PDPK1	100	28
PFCDPK1(P.falciparum)	PFB0815w	30	17
PFPK5(P.falciparum)	MAL13P1.279	100	84
PFTAIRE2	PFTK2	18	18
PFTK1	PFTK1	51	47
PHKG1	PHKG1	78	56
PHKG2	PHKG2	92	52
PIK3C2B	PIK3C2B	100	100

PIK3C2G	PIK3C2G	100	95
PIK3CA	PIK3CA	100	100
PIK3CA(C420R)	PIK3CA	100	88
PIK3CA(E542K)	PIK3CA	100	100
PIK3CA(E545A)	PIK3CA	100	100
PIK3CA(E545K)	PIK3CA	97	100
PIK3CA(H1047L)	PIK3CA	100	97
PIK3CA(H1047Y)	PIK3CA	94	93
PIK3CA(I800L)	PIK3CA	100	100
PIK3CA(M1043I)	PIK3CA	100	100
PIK3CA(Q546K)	PIK3CA	100	100
PIK3CB	PIK3CB	95	80
PIK3CD	PIK3CD	100	100
PIK3CG	PIK3CG	96	82
PIK4CB	PI4KB	80	2.9
PIM1	PIM1	96	76
PIM2	PIM2	100	100
PIM3	PIM3	95	72
PIP5K1A	PIP5K1A	100	82
PIP5K1C	PIP5K1C	60	100
PIP5K2B	PIP4K2B	100	44
PIP5K2C	PIP4K2C	3	2.3
PKAC-alpha	PRKACA	100	41
PKAC-beta	PRKACB	100	44
PKMYT1	PKMYT1	100	100
PKN1	PKN1	62	20
PKN2	PKN2	96	26
PKNB(M.tuberculosis)	pknB	82	19
PLK1	PLK1	100	100
PLK2	PLK2	99	9
PLK3	PLK3	97	44
PLK4	PLK4	89	43
PRKCD	PRKCD	90	76
PRKCE	PRKCE	72	55
PRKCH	PRKCH	100	49
PRKCI	PRKCI	81	79
PRKCQ	PRKCQ	74	13
PRKD1	PRKD1	88	18
PRKD2	PRKD2	74	26

PRKD3	PRKD3	98	11
PRKG1	PRKG1	100	72
PRKG2	PRKG2	100	73
PRKR	EIF2AK2	81	53
PRKX	PRKX	100	100
PRP4	PRPF4B	100	100
PYK2	PTK2B	100	2.2
QSK	KIAA0999	100	72
RAF1	RAF1	100	85
RET	RET	87	1.2
RET(M918T)	RET	91	0.35
RET(V804L)	RET	99	5.8
RET(V804M)	RET	68	3.4
RIOK1	RIOK1	97	65
RIOK2	RIOK2	16	2
RIOK3	RIOK3	94	66
RIPK1	RIPK1	90	17
RIPK2	RIPK2	80	18
RIPK4	RIPK4	100	99
RIPK5	DSTKY	86	45
ROCK1	ROCK1	86	79
ROCK2	ROCK2	95	77
ROS1	ROS1	100	100
RPS6KA4(Kin.Dom.1-N-terminal)	RPS6KA4	100	14
RPS6KA4(Kin.Dom.2-C-terminal)	RPS6KA4	57	72
RPS6KA5(Kin.Dom.1-N-terminal)	RPS6KA5	89	50
RPS6KA5(Kin.Dom.2-C-terminal)	RPS6KA5	86	89
RSK1(Kin.Dom.1-N-terminal)	RPS6KA1	56	0.05
RSK1(Kin.Dom.2-C-terminal)	RPS6KA1	43	27
RSK2(Kin.Dom.1-N-terminal)	RPS6KA3	37	0.1
RSK3(Kin.Dom.1-N-terminal)	RPS6KA2	71	0.15
RSK3(Kin.Dom.2-C-terminal)	RPS6KA2	93	88
RSK4(Kin.Dom.1-N-terminal)	RPS6KA6	59	0.4
RSK4(Kin.Dom.2-C-terminal)	RPS6KA6	16	2.9
S6K1	RPS6KB1	78	41
SBK1	SBK1	100	79
SgK110	SgK110	100	25
SGK3	SGK3	95	72
SIK	SIK1	84	6.4

SIK2	SIK2	78	7.1
SLK	SLK	100	95
SNARK	NUAK2	84	31
SNRK	SNRK	81	100
SRC	SRC	100	82
SRMS	SRMS	93	87
SRPK1	SRPK1	100	74
SRPK2	SRPK2	93	83
SRPK3	SRPK3	93	62
STK16	STK16	5	8.7
STK33	STK33	100	100
STK35	STK35	98	100
STK36	STK36	57	17
STK39	STK39	100	100
SYK	SYK	100	100
TAK1	MAP3K7	79	33
TAOK1	TAOK1	93	17
TAOK2	TAOK2	76	21
TAOK3	TAOK3	96	28
TBK1	TBK1	82	52
TEC	TEC	100	100
TESK1	TESK1	96	20
TGFBR1	TGFBR1	99	74
TGFBR2	TGFBR2	94	18
TIE1	TIE1	87	16
TIE2	TEK	87	44
TLK1	TLK1	100	62
TLK2	TLK2	99	56
TNIK	TNIK	89	10
TNK1	TNK1	67	3.7
TNK2	TNK2	91	25
TNNI3K	TNNI3K	93	43
TRKA	NTRK1	70	1
TRKB	NTRK2	99	6.4
TRKC	NTRK3	100	0.6
TRPM6	TRPM6	94	93
TSSK1B	TSSK1B	80	100
TTK	TTK	32	1.7
TXK	TXK	100	38

TYK2(JH1domain-catalytic)	TYK2	72	25
TYK2(JH2domain-pseudokinase)	TYK2	100	81
TYRO3	TYRO3	100	51
ULK1	ULK1	100	38
ULK2	ULK2	100	20
ULK3	ULK3	100	68
VEGFR2	KDR	73	45
VRK2	VRK2	95	72
WEE1	WEE1	87	48
WEE2	WEE2	100	100
YANK1	STK32A	69	88
YANK2	STK32B	100	100
YANK3	STK32C	100	100
YES	YES1	99	66
YSK1	STK25	100	22
YSK4	YSK4	90	53
ZAK	ZAK	100	9
ZAP70	ZAP70	86	99
