# Supplementary material for: LIM kinase inhibitors disrupt mitotic microtubule organization and impair tumor cell proliferation
Source: Oncotarget. 2015 Nov 3;6(36):38469–86. doi: 10.18632/oncotarget.6288 (PMC4770715; doi:10.18632/oncotarget.6288)
Supplement: Supplementary file 8 [file oncotarget-06-38469-s008.pdf]

Cell Line	Cosmic_ID	Primary_Site	Histology	GDSC_Desc1	GDSC_Desc2	CRT0105446_ALPHA	CRT0105446_BETA	CRT0105446_B	CRT0105446_IC_25	CRT0105446_IC_50	CRT0105446_IC_75	CRT0105446_IC_90	CRT0105446_AUC	CRT0105446_D	CRT0105446_IC_RESULTS_ID	CRT0105446_IC_50_LOW	CRT0105446_IC_50	CRT0105446_IC_50_HIGH
MC-CAR	683665	haematopoietic_and_lymphoid_tissue	lymphoid_neoplasm	blood	haematopoietic_neoplasm_other	0.856589	1.079212	0.103268	5.53575	7.020537	7.968626	9.264331	0.997738	0.0122	1258284	4.198063	7.020537	11.408789
PFSK-1	683667	central_nervous_system	primitive_neuroectodermal_tumour-medulloblastoma	nervous_system	medulloblastoma	0.784675	0.668406	0.153426	2.115014	4.333188	6.209747	8.417008	0.923755	0.0179	1049529	2.4463	4.333188	7.514613
A673	684052	soft_tissue	rhabdomyosarcoma	soft_tissue	rhabdomyosarcoma	0.837939	0.869986	0.077831	3.218704	4.810751	6.217145	7.805832	0.966391	0.0067	1049461	2.419655	4.810751	8.399203
ES3	684055	bone	Ewings_sarcoma-peripheral_primitive_neuroectodermal_tumour	bone	ewings_sarcoma	0.858408	0.847081	0.139398	2.763767	4.56296	5.906575	7.535387	0.955499	0.0039	1049370	2.894091	4.56296	7.633884
ES5	684057	bone	Ewings_sarcoma-peripheral_primitive_neuroectodermal_tumour	bone	ewings_sarcoma	0.848397	1.024533	0.089991	4.42115	6.020126	7.024775	8.406813	0.991649	0.006	1049372	3.613581	6.020126	10.283819
ES7	684059	bone	Ewings_sarcoma-peripheral_primitive_neuroectodermal_tumour	bone	ewings_sarcoma	0.788242	0.615887	0.138782	1.036993	3.182224	5.265435	7.612596	0.872796	0.0061	1049374	2.156258	3.182224	4.897815
EW-11	684062	bone	Ewings_sarcoma-peripheral_primitive_neuroectodermal_tumour	bone	ewings_sarcoma	0.836107	0.893019	0.110554	3.711892	5.469652	6.726159	8.319906	0.980163	0.0077	1049376	3.324437	5.469652	9.046868
NCI-H1395	684681	lung	carcinoma	lung	lung_NSCLC_adenocarcinoma	0.834967	0.827196	0.181261	2.541298	4.468792	5.821983	7.555575	0.953187	0.094	1036017	2.056016	4.468792	8.763332
COLO-829	687448	skin	malignant_melanoma	skin	melanoma	0.820799	0.719162	0.124401	1.622214	3.449101	5.138302	7.04969	0.90165	0.0031	1049364	2.45653	3.449101	5.194625
5637	687452	urinary_tract	carcinoma	urogenital_system	Bladder	0.825452	0.861624	0.059258	3.623681	5.429265	6.741868	8.401246	0.976274	0.0057	999977	3.558723	5.429265	8.840784
RT4	687455	urinary_tract	carcinoma	urogenital_system	Bladder	0.837044	0.948831	0.096715	3.882709	5.627656	6.769247	8.291802	0.983537	0.0137	1233313	3.474254	5.627656	9.610356
SW780	687457	urinary_tract	carcinoma	urogenital_system	Bladder	0.820252	0.732755	0.108239	2.474468	4.581322	6.183432	8.142552	0.946217	0.029	991336	2.80102	4.581322	8.227372
TCCSUP	687459	urinary_tract	carcinoma	urogenital_system	Bladder	0.845108	0.954104	0.074171	5.21876	6.909848	8.116538	9.618016	0.99451	0.0144	999987	4.142757	6.909848	11.333498
C-33-A	687505	cervix	carcinoma	urogenital_system	cervix	0.832423	0.845459	0.086708	2.963089	4.703457	6.040399	7.672967	0.961385	0.0016	1101546	3.209072	4.703457	7.577416
ME-180	687514	cervix	carcinoma	urogenital_system	cervix	0.832214	0.895409	0.074539	4.732581	6.52061	7.823153	9.422568	0.990555	0.0058	1233349	3.81564	6.52061	10.569917
8-MG-BA	687562	central_nervous_system	glioma	nervous_system	glioma	0.843757	0.81796	0.085898	1.959126	3.537522	4.997299	6.654526	0.930111	0.0046	1035880	2.678091	3.537522	5.032455
A172	687563	central_nervous_system	glioma	nervous_system	glioma	0.81113	0.758599	0.146955	2.458405	4.452215	6.051644	7.959424	0.945277	0.0073	1049437	2.770878	4.452215	7.463271
GB-1	687568	central_nervous_system	glioma	nervous_system	glioma	0.821636	0.714972	0.169828	1.978547	4.156676	5.862571	7.940588	0.928213	0.0271	1035907	2.423966	4.156676	7.866443
T98G	687586	central_nervous_system	glioma	nervous_system	glioma	0.827143	0.789526	0.117036	3.458962	5.452255	7.019447	8.859956	0.96715	0.0129	991341	3.339854	5.452255	8.88719
U-118-MG	687588	central_nervous_system	glioma	nervous_system	glioma	0.747527	0.547478	0.170795	-0.078186	2.401228	4.891726	7.633045	0.825542	0.0156	1035873	1.011025	2.401228	4.429386
U-87-MG	687590	central_nervous_system	glioma	nervous_system	glioma	0.706912	0.47419	0.124443	-0.203656	2.624903	5.588754	8.950296	0.828164	0.0182	991343	1.511282	2.624903	4.181955
YKG-1	687592	central_nervous_system	glioma	nervous_system	glioma	0.864609	0.988563	0.099515	4.620713	6.249667	7.310117	8.736326	0.993233	0.0044	1049360	3.655262	6.249667	10.484466
ChaGo-K-1	687596	lung	carcinoma	lung	lung_NSCLC_not_specified	0.800491	0.662308	0.071437	2.676064	4.810634	6.651862	8.819907	0.943606	0.0033	991371	3.300898	4.810634	7.597472
NCI-H720	687600	lung	carcinoid-endocrine_tumour	lung	lung_NSCLC_carcinoid	0.842381	1.084328	0.075365	5.815421	7.269618	8.248786	9.535411	0.998026	0.0065	1261056	4.370652	7.269618	11.68962
Calu-3	687777	lung	carcinoma	lung	lung_NSCLC_adenocarcinoma	0.827592	1.011508	0.124208	5.272125	6.878097	7.973603	9.397847	0.996071	0.019	999991	4.105901	6.878097	11.254303
COR-L23	687780	lung	carcinoma	lung	lung_NSCLC_large_cell	0.827656	0.942383	0.08836	4.225454	5.971861	7.140248	8.668478	0.988284	0.0013	1049423	3.686539	5.971861	10.005455
LK-2	687787	lung	carcinoma	lung	lung_NSCLC_squamous_cell_carcinoma	0.844218	1.035697	0.080233	4.830955	6.346859	7.439191	8.812407	0.993673	0.0115	1049480	2.973968	6.346859	10.832204
NCI-H1437	687794	lung	carcinoma	lung	lung_NSCLC_adenocarcinoma	0.834557	0.804111	0.087691	3.296789	5.219253	6.660935	8.436335	0.96463	0.0025	991451	3.365918	5.219253	8.674215
NCI-H1623	687798	lung	carcinoma	lung	lung_NSCLC_adenocarcinoma	0.83853	0.97814	0.129634	4.490192	6.141122	7.257099	8.729699	0.992107	0.0029	1049497	3.622155	6.141122	10.250105
NCI-H1648	687799	lung	carcinoma	lung	lung_NSCLC_adenocarcinoma	0.844075	0.973587	0.098216	4.682898	6.36331	7.464092	8.927651	0.992287	0.0064	1036019	3.844788	6.36331	10.570784
NCI-H1650	687800	lung	carcinoma	lung	lung_NSCLC_adenocarcinoma	0.839133	1.102459	0.066949	6.045606	7.464474	8.453907	9.740974	0.997909	0.0131	991453	4.638496	7.464474	11.708318
NCI-H1693	687802	lung	carcinoma	lung	lung_NSCLC_adenocarcinoma	0.842452	1.064442	0.106953	5.534766	7.012297	8.056329	9.399111	0.996585	0.0139	1000015	4.123834	7.012297	11.270516
NCI-H1770	687804	lung	carcinoma	lung	lung_NSCLC_not_specified	0.840079	1.054654	0.07981	5.248839	6.797532	7.814722	9.169311	0.996337	0.0018	1321972	4.067705	6.797532	11.118713
NCI-H1838	687807	lung	carcinoma	lung	lung_NSCLC_adenocarcinoma	0.837807	0.856289	0.162572	2.887892	4.844692	6.162283	7.851889	0.960667	0.0133	991455	2.890807	4.844692	9.10749
NCI-H2126	687814	lung	carcinoma	lung	lung_NSCLC_adenocarcinoma	0.850571	1.023871	0.133163	4.959346	6.512837	7.578264	8.963988	0.994074	0.0192	1049398	3.837941	6.512837	10.746624
NCI-H2170	687815	lung	carcinoma	lung	lung_NSCLC_squamous_cell_carcinoma	0.809437	0.664601	0.074308	3.131161	5.272454	7.111263	9.251615	0.956583	0.0034	991459	3.065971	5.272454	8.740042
NCI-H2228	687816	lung	carcinoma	lung	lung_NSCLC_adenocarcinoma	0.716129	0.353975	0.103072	0.25608	4.198137	8.144952	12.672881	0.871114	0.0075	991460	2.506037	4.198137	7.16709
NCI-H2347	687820	lung	carcinoma	lung	lung_NSCLC_adenocarcinoma	0.858702	1.055068	0.11897	5.449134	6.938968	7.972549	9.298778	0.996576	0.0369	1049507	4.098334	6.938968	11.23875
NCI-H2405	687821	lung	carcinoma	lung	lung_NSCLC_adenocarcinoma	0.835854	0.886044	0.100538	4.132819	6.102117	7.336569	8.98734	0.985522	0.023	1049508	3.578502	6.102117	10.810176
NCI-H661	687829	lung	carcinoma	lung	lung_NSCLC_large_cell	0.838134	1.007544	0.139837	4.544552	6.144305	7.217549	8.627891	0.992457	0.0096	1049512	3.591208	6.144305	10.385741
DMS-114	687983	lung	carcinoma	lung	lung_small_cell_carcinoma	0.721828	0.396454	0.121306	1.358378	5.127248	8.612977	12.747161	0.896515	0.0116	1101613	2.921008	5.127248	9.164298
DMS-273	687985	lung	carcinoma	lung	lung_small_cell_carcinoma	0.845275	0.986598	0.106573	4.531371	6.164838	7.253724	8.686179	0.991384	0.0167	991387	3.603064	6.164838	10.355684
NCI-H1048	687995	lung	carcinoma	lung	lung_small_cell_carcinoma	0.833064	0.924576	0.098534	3.178742	4.921487	6.076831	7.606835	0.971455	0.0045	1049495	3.151132	4.921487	8.64172
NCI-H1092	687997	lung	carcinoma	lung	lung_small_cell_carcinoma	0.83506	1.100324	0.079783	5.848305	7.296716	8.28207	9.584539	0.997947	0.0099	1323008	4.436287	7.296716	11.739481
NCI-H1694	688006	lung	carcinoma	lung	lung_small_cell_carcinoma	0.844487	1.00325	0.072129	4.884029	6.470225	7.567147	8.975179	0.994169	0.0027	1265312	3.970549	6.470225	10.439463
NCI-H187	688007	lung	carcinoma	lung	lung_small_cell_carcinoma	0.782315	0.577314	0.099686	2.490244	5.180163	7.346881	9.985158	0.932371	0.0107	1307086	3.203129	5.180163	8.966103
NCI-H1963	688010	lung	carcinoma	lung	lung_small_cell_carcinoma	0.845081	1.02627	0.085724	4.809973	6.400396	7.430245	8.796614	0.994527	0.003	1308710	3.74163	6.400396	10.776364
NCI-H2029	688011	lung	carcinoma	lung	lung_small_cell_carcinoma	0.693838	0.39437	0.144079	-0.332549	3.249045	6.944212	11.151554	0.846419	0.0239	1036026	1.42161	3.249045	5.760247
NCI-H2141	688014	lung	carcinoma	lung	lung_small_cell_carcinoma	0.857101	1.012893	0.078081	4.955212	6.560483	7.587629	8.964199	0.994099	0.0023	1266404	3.95077	6.560483	10.8867
NCI-H2227	688018	lung	carcinoma	lung	lung_small_cell_carcinoma	0.851882	1.01998	0.098823	5.219665	6.781753	7.807723	9.191043	0.996258	0.0038	1306862	4.072574	6.781753	11.126726
NCI-H345	688021	lung	carcinoma	lung	lung_small_cell_carcinoma	0.851074	1.062916	0.088751	5.61441	7.103683	8.1089	9.437768	0.997624	0.008	1266516	4.243678	7.103683	11.564654
NCI-H446	688023	lung	carcinoma	lung	lung_small_cell_carcinoma	0.846357	1.063155	0.098629	5.584878	7.029306	8.083312	9.427605	0.996966	0.0106	1307982	4.252109	7.029306	10.959593
NCI-H526	688025	lung	carcinoma	lung	lung_small_cell_carcinoma	0.858727	1.09623	0.083989	5.520269	6.922973	7.925815	9.176842	0.997542	0.0151	1263380	4.178626	6.922973	11.030359
NCI-H69	688027	lung	carcinoma	lung	lung_small_cell_carcinoma	0.84105	1.036298	0.073921	4.924963	6.506664	7.496499	8.863137	0.994287	0.001	1267076	3.96174	6.506664	10.762939
NCI-H82	688031	lung	carcinoma	lung	lung_small_cell_carcinoma	0.844535	1.087057	0.08391	5.558471	6.959514	7.971912	9.245987	0.99777	0.0102	1262400	4.317531	6.959514	10.940834
NCI-H2052	688058	pleura	mesothelioma	lung	mesothelioma	0.859558	0.975889	0.129827	4.028712	5.742976	6.802908	8.267234	0.987665	0.0132	1049436	3.306471	5.742976	10.215677
SK-N-DZ	688086	autonomic_ganglia	neuroblastoma	nervous_system	neuroblastoma	0.775876	0.559867	0.106702	2.504819	5.29834	7.645636	10.35851	0.9295	0.011	991328	3.197082	5.29834	8.979641
SK-N-FI	688087	autonomic_ganglia	neuroblastoma	nervous_system	neuroblastoma	0.825404	0.738448	0.148519	1.900398	3.892283	5.548326	7.461622	0.921693	0.0155	1049345	1.658508	3.892283	7.636208
VA-ES-BJ	688121	soft_tissue	epithelioid_sarcoma	soft_tissue	soft_tissue_other	0.72164	0.377942	0.082203	1.062589	4.671275	8.149489	12.216395	0.890064	0.0055	1101605	3.048935	4.671275	7.842264
SBC-5	713880	lung	carcinoma	lung	lung_small_cell_carcinoma	0.830921	0.765817	0.121802	2.204529	4.04754	5.619987	7.460773	0.935986	0.0169	1049334	2.748537	4.04754	6.468924
SBC-1	713885	lung	carcinoma	lung	lung_small_cell_carcinoma	0.849633	1.065456	0.08511	5.742312	7.205046	8.23177	9.542669	0.997881	0.0051	1255876	4.322367	7.205046	11.4266
LU-135	713899	lung	carcinoma	lung	lung_small_cell_carcinoma	0.860435	1.006553	0.068487	3.620957	5.240049	6.231937	7.620136	0.985095	0.003	1261280	3.35209	5.240049	9.140888
NCI-H2030	722045	lung	carcinoma	lung	lung_NSCLC_adenocarcinoma	0.846597	1.019094	0.113327	5.138218	6.717997	7.766975	9.155729	0.995818	0.0068	1049503	3.931533	6.717997	11.245299
NCI-H2122	722046	lung	carcinoma	lung	lung_NSCLC_adenocarcinoma	0.806925	0.635873	0.069183	0.823049	3.024329	4.777757	6.994813	0.854479	0.0046	991458	1.869624	3.024329	6.288404
NCI-H1734	722058	lung	carcinoma	lung	lung_NSCLC_adenocarcinoma	0.831449	0.905129	0.103489	4.585537	6.446354	7.705198	9.296845	0.990435	0.0234	1312356	3.857893	6.446354	10.693649
NCI-H650	722066	lung	carcinoma	lung	lung_NSCLC_adenocarcinoma	0.838553	0.987165	0.121102	3.765163	5.444966	6.503842	7.951672	0.984925	0.0027	1049511	3.211444	5.444966	9.473664
T-24	724812	urinary_tract	carcinoma	urogenital_system	Bladder	0.844811	0.844916	0.136164	2.831827	4.703297	6.02714	7.699716	0.955802	0.0089	1049351	2.965779	4.703297	8.270273
SK-N-AS	724828	autonomic_ganglia	neuroblastoma	nervous_system	neuroblastoma	0.85436	1.037344	0.098831	5.634158	7.195229	8.232384	9.588426	0.996455	0.0195	991327	4.306321	7.195229	11.676612
NCI-H1299	724831	lung	carcinoma	lung	lung_NSCLC_large_cell	0.831209	0.858182	0.07801	4.170358	6.138863	7.459487	9.14458	0.9832	0.007	1049496	3.799754	6.138863	10.428174
NCI-H2087	724834	lung	carcinoma	lung	lung_NSCLC_adenocarcinoma	0.769386	0.510375	0.07217	2.19474	4.854524	7.338417	10.184736	0.919399	0.0033	991457	3.335365	4.854524	7.49984
UM-UC-3	724838	urinary_tract	carcinoma	urogenital_system	Bladder	0.763732	0.435356	0.098411	0.699084	3.795215	6.844682	10.352123	0.872779	0.0103	991346	2.385818	3.795215	6.309897
SW756	724839	cervix	carcinoma	urogenital_system	cervix	0.649174	0.260797	0.119619	-0.91794	4.606023	10.3007	17.034536	0.878051	0.0091	1101574	2.427117	4.606023	8.706181
NCI-H727	724855	lung	carcinoid-endocrine_tumour	lung	lung_NSCLC_carcinoid	0.823065	0.809679	0.080405	3.00341	4.733369	6.189168	7.890918	0.954732	0.0073	991466	2.485073	4.733369	8.520051
Calu-6	724859	lung	carcinoma	lung	lung_NSCLC_adenocarcinoma	0.751189	0.541515	0.100082	-0.500889	1.852713	4.331215	7.092237	0.796541	0.0206	1049362	1.258953	1.852713	2.669744
LU-65	724863	lung	carcinoma	lung	lung_NSCLC_large_cell	0.836598	0.991045	0.066278	4.94635	6.590878	7.654718	9.105693	0.993915	0.0016	1266852	4.088761	6.590878	10.825312
NCI-H1355	724866	lung	carcinoma	lung	lung_NSCLC_adenocarcinoma	0.849052	0.970691	0.095586	4.260704	5.985967	7.063463	8.536968	0.988415	0.0048	1049397	3.530371	5.985967	10.292762
NCI-H1792	724868	lung	carcinoma	lung	lung_NSCLC_adenocarcinoma	0.84207	0.902393	0.115434	3.73788	5.598821	6.779433	8.369004	0.980336	0.0044	1049500	3.362516	5.598821	9.803314
HPAF-II	724869	pancreas	carcinoma	pancreas	pancreas	0.836518	1.036743	0.093869	5.456115	6.940559	8.011848	9.364157	0.996486	0.0114	1000005	4.151388	6.940559	11.175897
MIA-PaCa-2	724870	pancreas	carcinoma	pancreas	pancreas	0.8478	0.991063	0.055371	4.112675	5.77735	6.83737	8.267633	0.987252	0.0047	991445	3.687234	5.77735	9.838321
SHP-77	724872	lung	carcinoma	lung	lung_small_cell_carcinoma	0.803131	0.708332	0.104061	2.85782	4.992039	6.752707	8.784906	0.953137	0.0052	1049338	3.253927	4.992039	7.94105
NCI-H2009	724873	lung	carcinoma	lung	lung_NSCLC_adenocarcinoma	0.84634	0.885078	0.084383	4.009776	5.822906	7.095145	8.702784	0.983297	0.0039	1233353	3.619095	5.822906	9.642468
NCI-H2291	724874	lung	carcinoma	lung	lung_NSCLC_adenocarcinoma	0.852224	0.920367	0.09865	4.058103	5.870205	7.036769	8.576894	0.985121	0.0035	1049505	3.620089	5.870205	10.03608
SW1573	724878	lung	carcinoma	lung	lung_NSCLC_adenocarcinoma	0.846514	0.966901	0.058916	4.911588	6.552675	7.688565	9.146282	0.992593	0.0018	1101572	4.161132	6.552675	10.387303
SW900	724879	lung	carcinoma	lung	lung_NSCLC_squamous_cell_carcinoma	0.846074	1.063109	0.037377	5.735651	7.221107	8.240459	9.555529	0.99788	0.0015	991337	4.596452	7.221107	11.239073
TE-5	735784	oesophagus	carcinoma	aero_digestive_tract	oesophagus	0.827962	1.005514	0.064749	4.885229	6.48456	7.574471	9.02322	0.993717	0.0018	1035948	4.034596	6.48456	10.416369
HCC1954	749709	breast	carcinoma	breast	breast	0.844661	0.989262	0.104872	4.61047	6.254701	7.337783	8.759929	0.992234	0.0039	1049449	3.770639	6.254701	10.45131
HCC1143	749710	breast	carcinoma	breast	breast	0.827759	1.01114	0.0978	4.776267	6.401299	7.465308	8.889711	0.99305	0.0093	1233358	3.765451	6.401299	10.724131
HCC1187	749711	breast	carcinoma	breast	breast	0.752518	0.454576	0.179226	-0.479256	2.594024	5.604526	9.071634	0.830691	0.0229	1049445	1.19667	2.594024	4.888476
HCC1395	749712	breast	carcinoma	breast	breast	0.62916	0.254318	0.104367	0.474209	6.954919	13.21542	20.96296	0.912521	0.018	1101627	2.24499	6.954919	14.615603
HCC1937	749714	breast	carcinoma	breast	breast	0.848646	1.043617	0.084728	4.685822	6.275939	7.270287	8.634128	0.993582	0.0085	1049448	3.693559	6.275939	10.641183
HCC2157	749715	breast	carcinoma	breast	breast	0.832652	1.073981	0.089211	5.389116	6.904053	7.899901	9.222715	0.996918	0.0094	1261168	4.081649	6.904053	11.416147
HCC2218	749716	breast	carcinoma	breast	breast	0.844713	1.047396	0.112788	5.262424	6.773946	7.793123	9.14661	0.996263	0.0295	1307422	3.658483	6.773946	11.502079

HCC38	749717	breast	carcinoma	breast	breast	0.797771	0.659417	0.122786	1.986447	4.207752	6.136957	8.364029	0.923122	0.0174	1049450	2.036289	4.207752	7.844078
BB30-HNC	753531	upper_aerodigestive_tract	carcinoma	aero_digestive_tract	head_and_neck	0.82983	0.835946	0.089382	2.260956	3.865846	5.285685	6.934247	0.944598	0.0085	1035888	2.839707	3.865846	5.780838
BB49-HNC	753532	upper_aerodigestive_tract	carcinoma	aero_digestive_tract	head_and_neck	0.685912	0.323166	0.120336	-0.528677	3.732194	8.090335	13.10959	0.855948	0.0066	991354	2.209615	3.732194	6.494676
BB65-RCC	753533	kidney	carcinoma	kidney	kidney	0.803221	0.717282	0.156238	2.225382	4.360823	6.141613	8.161548	0.938648	0.0248	1035889	2.648309	4.360823	7.504926
BEN	753534	lung	carcinoma	lung	lung_NSCLC_not_specified	0.868463	1.032777	0.064378	5.189271	6.769268	7.795661	9.151477	0.99571	0.0044	991448	4.156236	6.769268	11.149826
BHY	753535	upper_aerodigestive_tract	carcinoma	aero_digestive_tract	head_and_neck	0.825291	0.800781	0.06724	2.853534	4.61474	6.051897	7.777833	0.957116	0.0064	991450	2.573049	4.61474	8.060531
BOKU	753536	cervix	carcinoma	urogenital_system	cervix	0.812344	0.621099	0.095684	3.007338	5.537972	7.502704	9.878415	0.950482	0.0112	1233325	3.395439	5.537972	9.356725
Ca9-22	753538	upper_aerodigestive_tract	carcinoma	aero_digestive_tract	head_and_neck	0.867127	0.997864	0.112237	3.737966	5.407362	6.384617	7.783018	0.986462	0.0156	1049342	3.183036	5.407362	9.64778
CAL-12T	753540	lung	carcinoma	lung	lung_NSCLC_not_specified	0.851661	1.002919	0.094425	4.438191	6.075382	7.120294	8.533344	0.990858	0.0032	1049347	3.684899	6.075382	10.16182
CAL-33	753541	upper_aerodigestive_tract	carcinoma	aero_digestive_tract	head_and_neck	0.750609	0.523839	0.112195	1.665668	4.351387	6.882782	9.767494	0.896126	0.0065	991340	2.207707	4.351387	7.566069
CP50-MEL-B	753545	skin	malignant_melanoma	skin	melanoma	0.832895	0.913176	0.122547	4.139511	5.963282	7.201326	8.800478	0.98778	0.0169	991355	3.523502	5.963282	10.194237
CP66-MEL	753546	skin	malignant_melanoma	skin	melanoma	0.792947	0.734718	0.051417	4.247287	6.350599	8.024569	10.027709	0.975933	0.0056	1233327	3.941326	6.350599	10.129604
CPC-N	753547	lung	carcinoma	lung	lung_small_cell_carcinoma	0.860756	1.045072	0.09352	4.457363	6.031969	6.989224	8.319612	0.992814	0.012	1258116	3.573859	6.031969	10.452555
CTV-1	753548	haematopoietic_and_lymphoid_tissue	haematopoietic_neoplasm	blood	acute_myeloid_leukaemia	0.84753	1.105711	0.067438	6.272127	7.670221	8.617314	9.867613	0.999017	0.0101	1257920	4.70519	7.670221	12.012172
D-542MG	753549	central_nervous_system	glioma	nervous_system	glioma	0.668295	0.267938	0.08871	-1.295602	4.452273	9.61525	15.928146	0.871806	0.0153	1035898	0.727251	4.452273	11.015609
DMS-79	753551	lung	carcinoma	lung	lung_small_cell_carcinoma	0.850813	1.08291	0.095773	5.978694	7.449374	8.428334	9.735357	0.997874	0.0236	1260944	4.52999	7.449374	11.835719
DSH1	753552	urinary_tract	carcinoma	urogenital_system	Bladder	0.834873	0.873195	0.128384	2.186883	3.779806	5.093789	6.665612	0.942151	0.0113	1035900	2.62148	3.779806	6.058196
EC-GI-10	753555	oesophagus	carcinoma	aero_digestive_tract	oesophagus	0.811365	0.702212	0.073063	3.201354	5.277225	6.964378	8.989348	0.963276	0.0089	1035901	3.530053	5.277225	8.383136
EPLC-272H	753556	lung	carcinoma	lung	lung_NSCLC_squamous_cell_carcinoma	0.841664	0.883736	0.098807	3.960766	5.793302	7.077962	8.693204	0.983685	0.0078	1049429	3.515712	5.793302	9.885226
HCE-4	753559	oesophagus	carcinoma	aero_digestive_tract	oesophagus	0.768069	0.567192	0.084716	2.844862	5.461742	7.698348	10.358847	0.940632	0.0092	999993	3.468629	5.461742	8.942255
HSC-2	753562	upper_aerodigestive_tract	carcinoma	aero_digestive_tract	head_and_neck	0.846586	0.990232	0.098014	4.282236	5.942189	6.987875	8.40783	0.98843	0.0118	1049464	3.57723	5.942189	10.266362
IM-9	753563	haematopoietic_and_lymphoid_tissue	lymphoid_neoplasm	blood	Myeloma	0.846018	1.027077	0.081159	4.964356	6.548906	7.552833	8.940834	0.994357	0.0015	1324352	3.963642	6.548906	10.859779
IST-SL1	753564	lung	carcinoma	lung	lung_small_cell_carcinoma	0.859291	0.596985	0.137381	-0.810276	1.223879	3.30408	5.534388	0.752083	0.0257	991361	0.548159	1.223879	1.894206
IST-SL2	753565	lung	carcinoma	lung	lung_small_cell_carcinoma	0.874525	1.008544	0.084156	3.971065	5.613529	6.644411	8.024372	0.987729	0.0022	1266180	3.390171	5.613529	9.585657
J82	753566	urinary_tract	carcinoma	urogenital_system	Bladder	0.828029	0.839358	0.109901	3.358359	5.229144	6.630782	8.348412	0.967362	0.0225	991419	2.418336	5.229144	9.528387
KNS-62	753569	lung	carcinoma	lung	lung_NSCLC_squamous_cell_carcinoma	0.84072	0.87221	0.08431	3.515859	5.311564	6.59549	8.230909	0.976509	0.0038	1101598	3.421593	5.311564	8.823446
KOSC-2	753570	upper_aerodigestive_tract	carcinoma	aero_digestive_tract	head_and_neck	0.829322	0.951063	0.100369	3.959447	5.749323	6.819263	8.327824	0.98697	0.0126	1000007	3.428532	5.749323	10.084865
KP-4	753572	pancreas	carcinoma	pancreas	pancreas	0.815887	0.706847	0.107695	2.294682	4.30799	6.009558	8.030314	0.933937	0.0093	991427	2.898047	4.30799	6.902706
KYSE-140	753573	oesophagus	carcinoma	aero_digestive_tract	oesophagus	0.742109	0.493981	0.081743	3.053767	6.113612	8.764019	11.982769	0.940929	0.0099	1049472	3.425872	6.113612	10.691474
KYSE-410	753574	oesophagus	carcinoma	aero_digestive_tract	oesophagus	0.813458	0.718572	0.085607	3.244143	5.392049	7.042993	9.064822	0.964786	0.0075	991430	3.408328	5.392049	8.864308
KYSE-520	753575	oesophagus	carcinoma	aero_digestive_tract	oesophagus	0.846045	1.014337	0.085265	3.534605	5.116483	6.141113	7.51834	0.982579	0.0064	1049476	3.252653	5.116483	8.679345
KYSE-70	753576	oesophagus	carcinoma	aero_digestive_tract	oesophagus	0.864537	1.087053	0.086351	5.644761	6.987745	8.029849	9.290001	0.99784	0.0365	1000010	4.009733	6.987745	10.922489
LB1047-RCC	753577	kidney	carcinoma	kidney	kidney	0.857733	0.971153	0.129206	3.445064	5.127232	6.201803	7.643046	0.980425	0.0156	1101599	3.052755	5.127232	9.000885
LB2241-RCC	753578	kidney	carcinoma	kidney	kidney	0.881301	1.134175	0.102936	1.606451	2.693754	3.719326	4.857123	0.912626	0.0207	1035920	2.243154	2.693754	3.531483
LB2518-MEL	753579	skin	malignant_melanoma	skin	melanoma	0.726417	0.200906	0.16743	-6.150711	0.54738	7.862291	15.871801	0.847745	0.0173	1035921	-2.000979	0.54738	3.60334
LB373-MEL-D	753581	skin	malignant_melanoma	skin	melanoma	0.781077	0.619319	0.088343	3.291236	5.86311	7.927795	10.372782	0.952946	0.0136	1233329	3.264787	5.86311	10.203389
LB771-HNC	753583	upper_aerodigestive_tract	carcinoma	aero_digestive_tract	head_and_neck	0.696412	0.416688	0.105611	-1.434137	1.809815	5.169309	9.019098	0.802463	0.0082	1035922	0.734921	1.809815	3.599547
LB831-BLC	753584	urinary_tract	carcinoma	urogenital_system	Bladder	0.825363	0.978865	0.066608	5.258211	6.877237	8.045812	9.509448	0.99576	0.0073	1035923	4.246345	6.877237	11.009494
LB996-RCC	753585	kidney	carcinoma	kidney	kidney	0.772104	0.63442	0.146859	0.604376	2.674232	4.735574	7.043679	0.856233	0.0091	1035924	1.756244	2.674232	4.046709
LCLC-103H	753586	lung	carcinoma	lung	lung_NSCLC_large_cell	0.852038	1.07034	0.075592	5.674491	7.162673	8.133326	9.438372	0.997855	0.009	991432	4.362234	7.162673	11.695441
LU-134-A	753588	lung	carcinoma	lung	lung_small_cell_carcinoma	0.851988	1.010288	0.085209	4.514801	6.186487	7.192048	8.613509	0.992926	0.0075	1265200	3.687062	6.186487	10.684498
LU-165	753589	lung	carcinoma	lung	lung_small_cell_carcinoma	0.851583	1.062775	0.076565	5.308267	6.847536	7.802905	9.126416	0.996178	0.002	1266292	4.162357	6.847536	11.170626
LXF-289	753592	lung	carcinoma	lung	lung_NSCLC_adenocarcinoma	0.829801	0.978947	0.056913	3.666899	5.277684	6.38483	7.830883	0.983119	0.0029	1101600	3.485847	5.277684	8.715117
MS-1	753594	lung	carcinoma	lung	lung_small_cell_carcinoma	0.84338	1.071231	0.090376	5.484605	6.976805	7.984929	9.31959	0.996432	0.0107	1263604	4.114671	6.976805	11.368718
MZ1-PC	753595	pancreas	carcinoma	pancreas	pancreas	0.829367	0.891697	0.073565	3.531097	5.544098	6.808675	8.458546	0.97077	0.0211	1035929	2.840284	5.544098	10.344751
MZ7-mel	753596	skin	malignant_melanoma	skin	melanoma	0.861129	0.917142	0.144372	2.586041	4.335379	5.470817	6.995699	0.953429	0.0145	1049393	2.708867	4.335379	7.846001
NCI-H1304	753599	lung	carcinoma	lung	lung_small_cell_carcinoma	0.865256	1.040781	0.0859	4.426131	6.017969	7.006139	8.349426	0.992735	0.0048	1259432	3.493663	6.017969	10.370564
NCI-H1563	753600	lung	carcinoma	lung	lung_NSCLC_adenocarcinoma	0.749563	0.515198	0.112195	0.687249	5.048511	8.082471	11.56798	0.89174	0.0349	1101672	1.085345	5.048511	10.753039
NCI-H292	753604	lung	carcinoma	lung	lung_NSCLC_adenocarcinoma	0.835304	1.082999	0.13835	5.46042	6.890187	7.906989	9.223535	0.996527	0.0456	1035971	4.144336	6.890187	10.918216
NCI-H510A	753605	lung	carcinoma	lung	lung_small_cell_carcinoma	0.864853	1.098254	0.094097	5.935512	7.322413	8.301455	9.557591	0.997951	0.0239	1308626	4.364357	7.322413	11.595029
PC-14	753608	lung	carcinoma	lung	lung_NSCLC_adenocarcinoma	0.836773	0.871263	0.072022	3.434343	5.163532	6.472909	8.065866	0.972599	0.001	1049527	3.462284	5.163532	8.254939
SK-MM-2	753612	haematopoietic_and_lymphoid_tissue	lymphoid_neoplasm	blood	Myeloma	0.846535	1.065606	0.078383	5.626665	7.098877	8.11633	9.433187	0.99771	0.0047	1325052	4.325977	7.098877	11.37168
TE-15	753614	oesophagus	carcinoma	aero_digestive_tract	oesophagus	0.836143	1.096851	0.074184	6.00959	7.393419	8.409499	9.696709	0.997867	0.0078	1258480	4.579467	7.393419	11.64888
SIMA	753620	autonomic_ganglia	neuroblastoma	nervous_system	neuroblastoma	0.6803	0.332485	0.106845	1.266852	5.734639	9.971318	15.021762	0.899581	0.0085	1233319	2.633733	5.734639	10.709891
TE-1	753621	oesophagus	carcinoma	aero_digestive_tract	oesophagus	0.843467	0.954712	0.118514	4.060066	5.762308	6.903211	8.392946	0.986884	0.0084	1049416	3.5085	5.762308	9.792158
TE-10	753622	oesophagus	carcinoma	aero_digestive_tract	oesophagus	0.851651	1.06673	0.087542	5.523209	7.028858	8.025997	9.364327	0.996401	0.0101	1035947	4.24998	7.028858	11.379239
TE-8	753623	oesophagus	carcinoma	aero_digestive_tract	oesophagus	0.829001	0.928588	0.105743	3.443686	5.200521	6.379264	7.933362	0.977429	0.0049	1049419	3.223329	5.200521	8.89003
CAPAN-1	753624	pancreas	carcinoma	pancreas	pancreas	0.846322	0.995186	0.14775	3.940798	5.644381	6.688128	8.141268	0.986338	0.0117	1049365	3.141005	5.644381	10.001995
OVCAR-3	905933	ovary	carcinoma	urogenital_system	ovary	0.836133	0.791421	0.052428	3.834736	5.711343	7.128616	8.880325	0.974387	0.0019	1036035	3.829639	5.711343	9.127198
PC-3	905934	prostate	carcinoma	urogenital_system	prostate	0.839184	1.004607	0.085241	4.718812	6.365107	7.426501	8.870328	0.992804	0.004	1049528	3.809594	6.365107	10.624423
DU-145	905935	prostate	carcinoma	urogenital_system	prostate	0.86429	1.044233	0.067109	5.260825	6.788671	7.803706	9.143275	0.996175	0.0099	1000001	3.701148	6.788671	11.373147
HCT-116	905936	large_intestine	carcinoma	digestive_system	large_intestine	0.695272	0.368304	0.095714	1.276916	5.108984	8.952215	13.389754	0.891204	0.0108	1049451	2.817887	5.108984	8.44704
HCT-15	905937	large_intestine	carcinoma	digestive_system	large_intestine	0.84127	0.969996	0.090256	3.834895	5.566922	6.629828	8.093012	0.98441	0.0135	1035976	3.421068	5.566922	9.736916
HL-60	905938	haematopoietic_and_lymphoid_tissue	haematopoietic_neoplasm	blood	acute_myeloid_leukaemia	0.83869	0.912752	0.106124	4.405179	6.222835	7.441265	9.011821	0.989567	0.0065	1254532	3.751546	6.222835	10.561559
HT-29	905939	large_intestine	carcinoma	digestive_system	large_intestine	0.801874	0.689049	0.099994	3.089094	5.357737	7.11893	9.245512	0.956985	0.0075	1049469	3.380408	5.357737	9.048081
K-562	905940	haematopoietic_and_lymphoid_tissue	haematopoietic_neoplasm	blood	chronic_myeloid_leukaemia	0.854888	0.985116	0.075417	3.584701	5.229732	6.247991	7.658893	0.982136	0.0044	1258228	3.340024	5.229732	9.077372
NCI-H226	905941	lung	carcinoma	lung	lung_NSCLC_squamous_cell_carcinoma	0.854459	1.041455	0.083471	5.186367	6.757602	7.755554	9.117548	0.996626	0.0058	1049504	4.006588	6.757602	11.337643
NCI-H23	905942	lung	carcinoma	lung	lung_NSCLC_adenocarcinoma	0.797452	0.718375	0.107346	2.05415	4.001574	5.752435	7.760886	0.929722	0.0165	1049506	2.357064	4.001574	6.51094
NCI-H460	905943	lung	carcinoma	lung	lung_NSCLC_large_cell	0.838065	0.916667	0.11914	3.795851	5.610202	6.794783	8.383368	0.981173	0.0088	1049509	3.341549	5.610202	9.850459
NCI-H522	905944	lung	carcinoma	lung	lung_NSCLC_adenocarcinoma	0.841788	1.072136	0.120577	5.224112	6.76189	7.701397	9.025939	0.996135	0.0261	1000020	3.958264	6.76189	11.313143
T47D	905945	breast	carcinoma	breast	breast	0.85277	0.967994	0.087447	2.891428	4.462073	5.551795	6.969867	0.966024	0.0022	1049352	3.053915	4.462073	7.522198
MCF7	905946	breast	carcinoma	breast	breast	0.864782	1.025165	0.104062	4.860082	6.429117	7.451256	8.811994	0.993539	0.0324	1036007	3.611598	6.429117	10.800458
786-0	905947	kidney	carcinoma	kidney	kidney	0.707321	1.418844	0.095182	-0.660891	0.210747	1.171426	2.222707	0.643382	0.0074	991388	-0.008811	0.210747	0.437674
A498	905948	kidney	carcinoma	kidney	kidney	0.819587	0.840847	0.095961	4.153107	5.988141	7.491864	9.275461	0.984249	0.0143	1035886	3.004498	5.988141	10.222493
A549	905949	lung	carcinoma	lung	lung_NSCLC_adenocarcinoma	0.733418	0.445643	0.092831	2.519039	6.150222	9.078112	12.739937	0.925129	0.0181	1049460	3.48987	6.150222	11.9516
ACHN	905950	kidney	carcinoma	kidney	kidney	0.891048	1.035191	0.127995	1.388156	2.634775	3.687912	4.935047	0.899086	0.013	991418	2.035901	2.634775	4.189346
BT-549	905951	breast	carcinoma	breast	breast	0.841305	0.965158	0.096823	4.37911	6.091069	7.231531	8.716294	0.988679	0.0096	1036036	3.676361	6.091069	10.241653
CCRF-CEM	905952	haematopoietic_and_lymphoid_tissue	haematopoietic_neoplasm	blood	lymphoblastic_leukemia	0.8557	0.942111	0.095645	3.848575	5.608233	6.752298	8.259554	0.983888	0.0032	1254196	3.417341	5.608233	9.693108
SK-MEL-28	905954	skin	malignant_melanoma	skin	melanoma	0.829251	0.816353	0.121948	2.919899	4.926018	6.378331	8.150166	0.959262	0.0361	991324	2.973371	4.926018	8.71243
SK-MEL-2	905955	skin	malignant_melanoma	skin	melanoma	0.822818	0.728236	0.115145	2.022307	3.94462	5.612742	7.569613	0.928202	0.0084	991323	2.639384	3.94462	6.331367
SK-MEL-5	905956	skin	malignant_melanoma	skin	melanoma	0.834157	1.012407	0.135239	4.441649	6.065767	7.101294	8.531003	0.991567	0.0643	1233332	3.055735	6.065767	10.894275
Hs-578-T	905957	breast	carcinoma	breast	breast	0.846345	0.918735	0.086639	3.771989	5.507623	6.69483	8.213007	0.981442	0.0064	1101640	3.495974	5.507623	9.21381
SK-OV-3	905959	ovary	carcinoma	urogenital_system	ovary	0.853213	0.906407	0.083561	4.099197	5.925327	7.096885	8.654222	0.985102	0.0055	1311964	3.727349	5.925327	10.170438
MDA-MB-231	905960	breast	carcinoma	breast	breast	0.844149	0.903148	0.087792	4.187089	6.08664	7.292298	8.88318	0.986947	0.0151	1036008	3.660455	6.08664	10.66384
COLO-205	905961	large_intestine	carcinoma	digestive_system	large_intestine	0.830302	1.059205	0.166485	5.521402	6.998918	8.052072	9.399981	0.996415	0.0872	1308122	4.153512	6.998918	11.340948
SW620	905962	large_intestine	carcinoma	digestive_system	large_intestine	0.829707	0.946684	0.051038	5.22711	6.99821	8.131034	9.639189	0.993588	0.0038	991338	4.398581	6.99821	11.349526
CAKI-1	905963	kidney	carcinoma	kidney	kidney	0.822292	0.70559	0.109838	1.629038	3.490217	5.216818	7.176471	0.904887	0.0118	991329	2.532016	3.490217	5.209841
RPMI-8226	905964	haematopoietic_and_lymphoid_tissue	haematopoietic_neoplasm	blood	Myeloma	0.872088	1.025367	0.07416	4.114257	5.775297	6.695698	8.042147	0.988452	0.0041	1258564	3.539107	5.775297	10.28184
SR	905965	haematopoietic_and_lymphoid_tissue	lymphoid_neoplasm	blood	lymphoid_neoplasm_other	0.833862	1.02082	0.092846	4.997884	6.601995	7.657399	9.070956	0.994443	0.0072	1267692	3.890272	6.601995	10.936052
NCI-H322M	905967	lung	carcinoma	lung	lung_NSCLC_adenocarcinoma	0.847062	1.08294	0.107432	5.819507	7.244169	8.265771	9.564871	0.997868	0.03	1324604	4.390418	7.244169	11.439228
IGROV-1	905968	ovary	carcinoma	urogenital_system	ovary	0.854198	0.912961	0.066024	4.490579	6.123468	7.415045	8.975005	0.989507	0.007	991417	3.203221	6.123468	10.500456
OVCAR-5	905969	ovary	carcinoma	urogenital_system	ovary	0.720067	0.451495	0.112548	2.339031	6.019936	9.025771	12.792865	0.919384	0.0176	1101692	3.44755	6.019936	11.234845
EKVX	905970	lung	carcinoma	lung	lung_NSCLC_adenocarcinoma	0.815018	0.882572	0.073517	4.560807	6.418283	7.746364	9.405243	0.989403	0.0091	1101588	3.731181	6.418283	10.527113

HCC2998	905971	large_intestine	carcinoma	digestive_system	large_intestine	0.851474	1.010761	0.101774	3.559477	5.16444	6.192171	7.575739	0.981355	0.0033	1049379	3.21451	5.16444	8.852198
HOP-62	905972	lung	carcinoma	lung	lung_NSCLC_adenocarcinoma	0.84474	0.926892	0.117554	4.296982	6.02961	7.301145	8.843072	0.98789	0.026	1233328	3.514748	6.02961	10.006771
HOP-92	905973	lung	carcinoma	lung	lung_NSCLC_large_cell	0.834277	0.801735	0.136179	1.99691	3.811773	5.235389	6.983342	0.930196	0.0256	1000004	2.525088	3.811773	6.629504
LOXIMVI	905974	skin	malignant_melanoma	skin	melanoma	0.759498	0.504329	0.158196	0.824636	3.821307	6.451119	9.506525	0.874736	0.0179	991363	1.627338	3.821307	7.423823
M14	905975	skin	malignant_melanoma	skin	melanoma	0.843219	0.842946	0.103214	3.294078	5.115489	6.520962	8.177256	0.965995	0.0098	1049484	2.375502	5.115489	9.406231
UACC-62	905976	skin	malignant_melanoma	skin	melanoma	0.851182	0.949121	0.116962	3.17907	4.897285	6.014521	7.511989	0.971717	0.0129	1049357	3.05591	4.897285	8.58038
UACC-257	905977	skin	malignant_melanoma	skin	melanoma	0.857898	1.019972	0.13851	4.812358	6.379014	7.436593	8.8213	0.993835	0.0349	1049421	3.717078	6.379014	10.68498
RXF393	905978	kidney	carcinoma	kidney	kidney	0.838806	1.03016	0.099421	4.963758	6.516736	7.584081	8.966689	0.994625	0.0124	1049407	3.906557	6.516736	10.704898
SN12C	905979	kidney	carcinoma	kidney	kidney	0.86483	1.021284	0.124606	4.176185	5.782983	6.801901	8.181213	0.988758	0.0197	999983	3.34982	5.782983	10.02314
TK10	905980	kidney	carcinoma	kidney	kidney	0.809435	0.665372	0.091553	2.032622	4.101569	5.981717	8.128739	0.922032	0.0242	1035950	2.258829	4.101569	6.792827
U031	905981	kidney	carcinoma	kidney	kidney	0.862267	0.912147	0.110894	3.463703	5.227752	6.390065	7.915296	0.976637	0.0037	1035876	3.265195	5.227752	9.173002
SNB75	905982	central_nervous_system	glioma	nervous_system	glioma	0.692989	0.354455	0.084353	2.161614	6.35337	10.253388	14.937848	0.920244	0.0066	1311684	3.655585	6.35337	11.38403
U251	905983	central_nervous_system	glioma	nervous_system	glioma	0.843037	0.962832	0.11155	3.750479	5.465555	6.535586	7.991138	0.982002	0.0184	999990	3.310738	5.465555	9.660625
SF539	905984	central_nervous_system	glioma	nervous_system	glioma	0.837407	1.077923	0.098838	5.642002	7.083205	8.114884	9.426323	0.997958	0.029	1035940	3.97917	7.083205	11.536508
SF295	905985	central_nervous_system	glioma	nervous_system	glioma	0.841328	0.838679	0.089137	3.39307	5.210955	6.559783	8.23002	0.972318	0.0085	1101555	3.363758	5.210955	8.5813
SF268	905986	central_nervous_system	glioma	nervous_system	glioma	0.844129	1.009742	0.087327	4.737479	6.343106	7.390363	8.809299	0.993082	0.0111	1035939	3.863567	6.343106	10.640089
KM12	905989	large_intestine	carcinoma	digestive_system	large_intestine	0.847988	0.897758	0.135331	3.452052	5.316502	6.514086	8.092599	0.973961	0.0158	1049386	3.200051	5.316502	9.278688
OVCAR-4	905990	ovary	carcinoma	urogenital_system	ovary	0.855692	1.085712	0.07386	5.843206	7.281056	8.24901	9.532614	0.997906	0.0078	1101603	4.509907	7.281056	11.576012
OVCAR-8	905991	ovary	carcinoma	urogenital_system	ovary	0.853744	0.912104	0.092084	4.227994	5.868128	7.143647	8.661983	0.98745	0.0072	1049523	2.897772	5.868128	9.855805
BxPC-3	906693	pancreas	carcinoma	pancreas	pancreas	0.842771	1.057763	0.070145	5.62775	7.131906	8.110174	9.434392	0.997503	0.0071	991470	4.283591	7.131906	11.713515
BHT-101	906696	thyroid	carcinoma	thyroid	thyroid	0.773731	0.514593	0.081955	0.938189	3.454962	5.947501	8.733843	0.882071	0.0055	1101671	2.496859	3.454962	5.08955
Becker	906746	central_nervous_system	glioma	nervous_system	glioma	0.830169	0.779427	0.073721	2.732965	4.605152	6.080255	7.884261	0.957459	0.0049	1035890	3.130845	4.605152	7.376273
BE-13	906763	haematopoietic_and_lymphoid_tissue	haematopoietic_neoplasm	blood	lymphoblastic_leukemia	0.8542	1.047153	0.135374	5.336464	6.841071	7.868671	9.226433	0.996491	0.0342	1309494	3.941801	6.841071	11.13419
ARH-77	906765	haematopoietic_and_lymphoid_tissue	haematopoietic_neoplasm	blood	Myeloma	0.840339	1.080677	0.100068	5.613634	7.092138	8.088526	9.404899	0.99773	0.0123	1324380	4.251586	7.092138	11.615003
AGS	906790	stomach	carcinoma	digestive_system	stomach	0.762508	0.507754	0.106669	0.482967	4.37913	7.189782	10.378175	0.862364	0.0088	991421	1.887874	4.37913	9.208603
ABC-1	906791	lung	carcinoma	lung	lung_NSCLC_adenocarcinoma	0.829089	0.894515	0.063577	4.673459	6.507579	7.777145	9.396575	0.989473	0.0041	1049463	3.956419	6.507579	10.760747
A2058	906792	skin	malignant_melanoma	skin	melanoma	0.788991	0.581544	0.08485	2.965693	5.576666	7.73119	10.319463	0.94738	0.0066	991402	3.53242	5.576666	9.396935
A375	906793	skin	malignant_melanoma	skin	melanoma	0.810356	0.569855	0.092653	1.233625	3.503299	5.708873	8.174728	0.881138	0.0064	1049458	2.553942	3.503299	5.200209
A253	906794	salivary_gland	carcinoma	aero_digestive_tract	head_and_neck	0.647721	0.287405	0.100369	1.326939	6.605165	11.605727	17.697554	0.907142	0.0088	1311992	3.063165	6.605165	12.679868
8305C	906795	thyroid	carcinoma	thyroid	thyroid	0.849096	0.902958	0.116474	3.693232	5.540383	6.745695	8.32664	0.980424	0.0046	1049428	3.295653	5.540383	9.626607
647-V	906797	urinary_tract	carcinoma	urogenital_system	Bladder	0.842177	0.97332	0.108152	4.092665	5.789464	6.855454	8.314843	0.986911	0.0185	1049424	3.494486	5.789464	10.051878
639-V	906798	urinary_tract	carcinoma	urogenital_system	Bladder	0.758284	0.479593	0.087367	2.304259	5.276705	8.006593	11.196393	0.919894	0.0052	991382	3.109607	5.276705	8.765502
697	906800	haematopoietic_and_lymphoid_tissue	haematopoietic_neoplasm	blood	lymphoblastic_leukemia	0.891809	1.272264	0.055583	2.385331	3.436929	4.267997	5.265491	0.962964	0.0039	1257752	2.790519	3.436929	4.942494
BT-20	906801	breast	carcinoma	breast	breast	0.845981	1.009191	0.082303	4.605329	6.220055	7.243497	8.639643	0.992762	0.0037	1049514	3.777129	6.220055	10.411904
ACN	906803	autonomic_ganglia	neuroblastoma	nervous_system	neuroblastoma	0.812448	0.700505	0.187901	2.18874	4.407625	6.170901	8.24996	0.928781	0.0226	1049361	2.54292	4.407625	8.177721
A2780	906804	ovary	carcinoma	urogenital_system	ovary	0.813585	0.692919	0.123882	2.325403	4.390385	6.153966	8.202154	0.933275	0.0041	1049447	2.902179	4.390385	7.076954
COR-L105	906805	lung	carcinoma	lung	lung_NSCLC_adenocarcinoma	0.833649	0.892458	0.137533	2.066017	3.652901	4.919974	6.466179	0.939232	0.0042	1049422	2.522482	3.652901	6.144925
CRO-AP2	906807	haematopoietic_and_lymphoid_tissue	lymphoid_neoplasm	blood	B_cell_lymphoma	0.843142	1.075604	0.071256	5.337556	6.842431	7.826041	9.135862	0.997043	0.0027	1307310	4.116066	6.842431	11.397013
COR-L88	906808	lung	carcinoma	lung	lung_small_cell_carcinoma	0.841825	1.076535	0.077636	6.031761	7.429172	8.47493	9.779125	0.997871	0.017	1261504	4.563096	7.429172	11.496169
COLO-824	906812	breast	carcinoma	breast	breast	0.841203	0.868603	0.097843	3.360169	5.155583	6.458684	8.093149	0.972058	0.0225	1035954	3.310692	5.155583	8.599395
COLO-800	906813	skin	malignant_melanoma	skin	melanoma	0.814394	0.640231	0.116052	1.979078	4.70081	6.714768	9.061253	0.916364	0.012	1035953	2.427968	4.70081	9.106288
COLO-792	906814	skin	malignant_melanoma	skin	melanoma	0.82236	0.701156	0.178558	0.937373	2.826371	4.560123	6.566425	0.864743	0.0092	991377	1.815925	2.826371	4.726716
COLO-741	906815	large_intestine	carcinoma	digestive_system	large_intestine	0.779385	0.513517	0.080469	2.057811	4.735539	7.200564	10.057693	0.911907	0.0053	991376	3.151898	4.735539	7.485149
COLO-680N	906817	oesophagus	carcinoma	aero_digestive_tract	oesophagus	0.851046	1.048121	0.059278	5.300271	6.859488	7.848766	9.196561	0.996019	0.0112	1035952	3.79724	6.859488	11.641178
COLO-679	906818	skin	malignant_melanoma	skin	melanoma	0.834528	0.893403	0.123574	4.057572	5.963288	7.210284	8.829489	0.984158	0.0107	1035951	3.485119	5.963288	10.386839
CHP-212	906820	autonomic_ganglia	neuroblastoma	nervous_system	neuroblastoma	0.824987	0.808933	0.087712	3.463174	5.357684	6.806033	8.567698	0.968311	0.0083	991374	3.049342	5.357684	8.915117
CFPAC-1	906821	pancreas	carcinoma	pancreas	pancreas	0.827283	0.780342	0.116329	3.267126	5.318897	6.876932	8.735955	0.964901	0.0366	991369	2.989622	5.318897	9.168137
CCF-STTG1	906823			nervous_system	glioma	0.681874	0.30407	0.073672	1.866586	6.517386	11.058213	16.419274	0.914587	0.0029	1233359	3.925019	6.517386	11.024048
Ca-Ski	906824	cervix	carcinoma	urogenital_system	cervix	0.729819	0.390157	0.090745	1.313374	4.962602	8.436501	12.522291	0.896723	0.0081	1233318	2.918146	4.962602	8.682555
CAL-120	906826	breast	carcinoma	breast	breast	0.773054	0.623294	0.087164	2.183119	4.455095	6.581795	9.042865	0.924452	0.0134	991331	2.990649	4.455095	7.106507
CAL-72	906827	bone	osteosarcoma	bone	osteosarcoma	0.902199	1.084791	0.059044	2.221413	3.485843	4.465987	5.646715	0.950018	0.007	991344	2.722546	3.485843	5.439166
CAL-62	906828	thyroid	carcinoma	thyroid	thyroid	0.817115	0.684346	0.131445	0.871385	2.726596	4.547675	6.582661	0.860145	0.0128	1049356	1.995409	2.726596	3.976715
C32	906830	skin	malignant_melanoma	skin	melanoma	0.783248	0.474859	0.074941	1.059159	3.79374	6.488041	9.5127	0.878802	0.0036	991311	2.706292	3.79374	5.694601
Daudi	906831	haematopoietic_and_lymphoid_tissue	lymphoid_neoplasm	blood	Burkitt_lymphoma	0.852606	0.98724	0.135351	3.853391	5.504337	6.57346	8.009792	0.987142	0.0176	1323932	3.186099	5.504337	9.501386
Daoy	906833	central_nervous_system	primitive_neuroectodermal_tumour-medulloblastoma	nervous_system	medulloblastoma	0.81662	0.787322	0.079204	3.479952	5.472201	6.938937	8.754638	0.967574	0.0046	991384	3.517178	5.472201	9.049142
D-283MED	906834	central_nervous_system	primitive_neuroectodermal_tumour-medulloblastoma	nervous_system	medulloblastoma	0.838671	1.063491	0.083361	5.331141	6.803445	7.817788	9.154109	0.996108	0.0082	1101612	4.103023	6.803445	10.923664
DBTRG-05MG	906835	central_nervous_system	glioma	nervous_system	glioma	0.846183	0.983803	0.103423	4.398178	6.085641	7.137937	8.581714	0.989949	0.0082	1049425	3.596352	6.085641	10.365917
DEL	906836	haematopoietic_and_lymphoid_tissue	lymphoid_neoplasm	blood	lymphoid_neoplasm_other	0.847288	1.061238	0.077189	5.47481	6.992358	7.981362	9.288431	0.996389	0.0013	1256968	4.308135	6.992358	11.241992
Detroit562	906837	upper_aerodigestive_tract	carcinoma	aero_digestive_tract	head_and_neck	0.833958	0.879897	0.06831	2.840354	4.387892	5.741332	7.307283	0.960184	0.0027	991385	3.213184	4.387892	6.425016
DG-75	906838	haematopoietic_and_lymphoid_tissue	lymphoid_neoplasm	blood	Burkitt_lymphoma	0.855824	1.053414	0.113838	5.195434	6.678522	7.684101	9.006464	0.996465	0.0187	1267412	3.978486	6.678522	10.777505
DK-MG	906839	central_nervous_system	glioma	nervous_system	glioma	0.836232	0.917313	0.113539	4.307121	6.126729	7.326957	8.912455	0.98855	0.0105	1049426	3.641146	6.126729	10.41553
DJM-1	906841	skin	adnexal_tumour	skin	skin_other	0.851027	0.909344	0.054689	3.938255	5.633918	6.870952	8.403521	0.983053	0.0017	1035899	3.735505	5.633918	9.052392
DoTc2-4510	906843	cervix	carcinoma	urogenital_system	cervix	0.835997	0.955756	0.17222	4.66529	6.341936	7.486082	8.998782	0.992365	0.0479	1035956	3.593646	6.341936	10.65124
DU-4475	906844	breast	carcinoma	breast	breast	0.855407	1.000664	0.08327	4.833031	6.434682	7.509628	8.917182	0.992999	0.002	1259768	3.823823	6.434682	10.645073
EB2	906846	haematopoietic_and_lymphoid_tissue	lymphoid_neoplasm	blood	Burkitt_lymphoma	0.851397	1.084394	0.07665	5.69701	7.178428	8.148299	9.437733	0.997996	0.0045	1325108	4.218571	7.178428	11.756875
EB-3	906847	haematopoietic_and_lymphoid_tissue	lymphoid_neoplasm	blood	Burkitt_lymphoma	0.84369	1.068832	0.085881	5.51709	6.958077	7.987899	9.305349	0.996675	0.0142	1324128	3.734219	6.958077	11.443191
ECC10	906848	stomach	carcinoma	digestive_system	stomach	0.850551	1.059199	0.107863	5.265967	6.743382	7.772959	9.097835	0.996412	0.0064	1261728	3.972287	6.743382	10.928879
EFM-19	906851	breast	carcinoma	breast	breast	0.83615	0.906873	0.062202	4.570054	6.26435	7.604765	9.176742	0.989559	0.0082	991391	2.946766	6.26435	10.024335
EFO-27	906852	ovary	carcinoma	urogenital_system	ovary	0.829132	0.722135	0.08684	2.592467	4.648096	6.280862	8.232723	0.942309	0.0084	1035958	2.375773	4.648096	8.692927
EGI-1	906853	biliary_tract	carcinoma	digestive_system	biliary_tract	0.849012	1.041454	0.080286	5.420371	6.963836	8.020336	9.386542	0.996346	0.0098	991392	4.127874	6.963836	11.371956
EM-2	906855	haematopoietic_and_lymphoid_tissue	haematopoietic_neoplasm	blood	chronic_myeloid_leukaemia	0.86047	1.073963	0.072666	5.635975	7.112148	8.094443	9.395852	0.997657	0.0045	1309382	4.315349	7.112148	11.478242
EoL-1-cell	906856	haematopoietic_and_lymphoid_tissue	haematopoietic_neoplasm	blood	haematopoietic_neoplasm_other	0.863378	1.067432	0.151075	5.104818	6.620366	7.597866	8.915483	0.996369	0.0543	1322980	3.814439	6.620366	11.194344
ETK-1	906861	biliary_tract	carcinoma	digestive_system	biliary_tract	0.852719	0.86321	0.112503	2.151719	3.765029	5.070377	6.652285	0.94354	0.0068	1035904	2.660474	3.765029	6.030149
EVSA-T	906862	breast	carcinoma	breast	breast	0.848468	0.825373	0.127884	2.886555	4.725865	6.126808	7.813458	0.95526	0.0098	1233335	3.035669	4.725865	7.958618
FADU	906863	upper_aerodigestive_tract	carcinoma	aero_digestive_tract	head_and_neck	0.850739	0.954351	0.110386	4.412231	6.090664	7.218592	8.666173	0.989285	0.0209	1000002	3.727409	6.090664	10.104716
FTC-133	906864	thyroid	carcinoma	thyroid	thyroid	0.842098	0.931617	0.104771	4.410078	6.091277	7.307498	8.837035	0.990325	0.0125	1049432	2.970185	6.091277	10.409004
G-361	906865	skin	malignant_melanoma	skin	melanoma	0.762376	0.481959	0.109842	0.388424	3.099439	5.826055	8.881794	0.851574	0.0017	1035962	2.153092	3.099439	4.670358
GAMG	906868	central_nervous_system	glioma	nervous_system	glioma	0.857531	0.984452	0.125649	4.077233	5.768423	6.814665	8.256742	0.987495	0.011	1049434	3.396473	5.768423	10.127587
GCIY	906869	stomach	carcinoma	digestive_system	stomach	0.885125	1.045126	0.121225	2.26755	3.700888	4.631652	5.900067	0.948304	0.0099	999992	2.577548	3.700888	6.599863
GDM-1	906870	haematopoietic_and_lymphoid_tissue	haematopoietic_neoplasm	blood	acute_myeloid_leukaemia	0.847494	0.947058	0.082029	3.938506	5.656323	6.813758	8.318734	0.984669	0.003	1266628	3.529596	5.656323	9.501708
GI-1	906871	central_nervous_system	glioma	nervous_system	glioma	0.849802	0.901431	0.118368	1.759883	3.262507	4.525828	6.018219	0.92984	0.03	1035908	2.196442	3.262507	5.339751
GI-ME-N	906872	autonomic_ganglia	neuroblastoma	nervous_system	neuroblastoma	0.877589	0.992284	0.077817	1.572579	2.806708	3.988324	5.276972	0.91403	0.0044	991358	2.35522	2.806708	3.601203
GMS-10	906873	central_nervous_system	glioma	nervous_system	glioma	0.84878	0.822568	0.084671	2.822005	4.561471	5.934079	7.596801	0.955291	0.001	1049435	3.179817	4.561471	7.167971
GOTO	906875	autonomic_ganglia	neuroblastoma	nervous_system	neuroblastoma	0.837366	0.915141	0.11033	3.797607	5.496385	6.781205	8.341895	0.981044	0.0149	1101621	2.463595	5.496385	9.846867
GR-ST	906877	haematopoietic_and_lymphoid_tissue	haematopoietic_neoplasm	blood	lymphoblastic_leukemia	0.855841	1.036675	0.148828	5.178529	6.687049	7.727277	9.080544	0.996352	0.0081	1255988	3.900607	6.687049	11.057968
GCT	906999	soft_tissue	malignant_fibrous_histiocytoma-pleomorphic_sarcoma	soft_tissue	soft_tissue_other	0.79205	0.695006	0.100586	3.02697	5.310274	7.067568	9.188158	0.958549	0.0155	1035965	3.394315	5.310274	8.875437
ESS-1	907000	endometrium	carcinoma	urogenital_system	endometrium	0.779115	0.48219	0.109699	0.887409	3.673551	6.327371	9.350119	0.871051	0.0149	1035959	2.49164	3.673551	5.889603
GT3TKB	907041	stomach	carcinoma	digestive_system	stomach	0.827311	0.734778	0.115356	2.477307	4.447992	6.088792	8.012472	0.94686	0.014	1035909	2.949883	4.447992	7.185739
H4	907042	central_nervous_system	glioma	nervous_system	glioma	0.856373	1.084341	0.08633	5.785728	7.161876	8.185491	9.452483	0.997817	0.0129	991401	4.047864	7.161876	11.473754
H9	907043	haematopoietic_and_lymphoid_tissue	lymphoid_neoplasm	blood	lymphoid_neoplasm_other	0.845865	0.976315	0.083145	4.78571	6.47294	7.617333	9.100152	0.992767	0.002	1254308	3.942218	6.47294	10.655309
HC-1	907044	haematopoietic_and_lymphoid_tissue	lymphoid_neoplasm	blood	hairy_cell_leukaemia	0.836059	0.999974	0.07589	4.824025	6.500836	7.537893	8.978919	0.993848	0.0021	1264780	3.938448	6.500836	11.002582
HCC1419	907045	breast	carcinoma	breast	breast	0.840925	0.960671	0.17195	4.813817	6.471644	7.686257	9.199279	0.992728	0.054	1101628	3.695022	6.471644	10.638473
HCC1569	907046	breast	carcinoma	breast	breast	0.862723	1.03234	0.105868	4.846185	6.38109	7.4088	8.748256	0.993755	0.0113	1049446	3.854362	6.38109	10.558337

HCC1806	907047	breast	carcinoma	breast	breast	0.84407	0.806287	0.079517	3.239713	5.061341	6.559453	8.318398	0.967012	0.0064	991403	3.359612	5.061341	7.979039
HCC70	907048	breast	carcinoma	breast	breast	0.844544	0.966102	0.061885	3.085594	4.583506	5.730955	7.143549	0.971086	0.0049	991405	3.285907	4.583506	7.063544
HCE-T	907049	upper_aerodigestive_tract	carcinoma	aero_digestive_tract	head_and_neck	0.836363	1.017101	0.095388	4.65575	6.324318	7.33509	8.741282	0.993111	0.0049	1049380	3.776394	6.324318	10.893176
HD-MY-Z	907050	haematopoietic_and_lymphoid_tissue	lymphoid_neoplasm	blood	Hodgkin_lymphoma	0.812583	0.733987	0.081517	2.799179	4.746547	6.410618	8.369938	0.953862	0.0071	1101633	3.24737	4.746547	7.361884
HEC-1	907051	endometrium	carcinoma	urogenital_system	endometrium	0.827539	0.893327	0.109468	4.17269	5.990082	7.300347	8.942615	0.985973	0.0125	1049452	3.228459	5.990082	10.252212
HEL	907053	haematopoietic_and_lymphoid_tissue	haematopoietic_neoplasm	blood	acute_myeloid_leukaemia	0.824979	0.881324	0.063798	4.583818	6.423195	7.726875	9.36338	0.989217	0.0042	991407	4.093851	6.423195	10.231474
HGC-27	907055	stomach	carcinoma	digestive_system	stomach	0.819639	0.793501	0.121825	2.368857	4.172875	5.70046	7.495232	0.945258	0.0107	1049454	2.756654	4.172875	6.674498
HH	907056	haematopoietic_and_lymphoid_tissue	lymphoid_neoplasm	blood	T_cell_leukemia	0.822421	0.829708	0.114068	3.066718	4.986291	6.374053	8.093603	0.962963	0.0042	1254420	3.139643	4.986291	8.39116
HLE	907057	liver	carcinoma	digestive_system	liver	0.902651	0.987871	0.094423	1.464808	3.009363	3.821633	5.12202	0.905756	0.0074	1049456	1.988121	3.009363	5.88079
HMV-II	907058	skin	malignant_melanoma	skin	melanoma	0.77227	0.508185	0.13627	1.480905	4.363686	6.962565	9.984363	0.897273	0.0204	991408	2.70001	4.363686	7.549462
HN	907059	upper_aerodigestive_tract	carcinoma	aero_digestive_tract	head_and_neck	0.762059	0.560557	0.067796	3.007311	5.611019	7.893375	10.594482	0.946031	0.0039	1000003	3.616778	5.611019	9.014064
HOS	907060	bone	osteosarcoma	bone	osteosarcoma	0.773242	0.563826	0.101874	1.167104	3.515923	5.784075	8.374321	0.877528	0.0108	1035980	2.507794	3.515923	5.374313
HSC-3	907061	upper_aerodigestive_tract	carcinoma	aero_digestive_tract	head_and_neck	0.835777	0.939711	0.088995	4.874857	6.589083	7.809806	9.332039	0.992792	0.0154	1000006	4.095314	6.589083	10.580548
HSC-4	907062	upper_aerodigestive_tract	carcinoma	aero_digestive_tract	head_and_neck	0.839512	0.877549	0.12744	3.871049	5.764669	7.072644	8.720236	0.981641	0.0137	1049465	2.965032	5.764669	10.33509
HT	907063	haematopoietic_and_lymphoid_tissue	lymphoid_neoplasm	blood	B_cell_lymphoma	0.826813	1.107632	0.062281	6.082447	7.494084	8.475941	9.766575	0.997905	0.0103	1267188	4.422927	7.494084	11.908811
HT-1080	907064	soft_tissue	fibrosarcoma	soft_tissue	fibrosarcoma	0.840709	0.887707	0.112863	3.035311	4.870833	6.091145	7.704017	0.965631	0.006	1049466	3.090131	4.870833	8.545074
HT-1197	907065	urinary_tract	carcinoma	urogenital_system	Bladder	0.840062	1.028694	0.106099	4.782267	6.378626	7.41821	8.80914	0.993623	0.0062	1049467	3.799705	6.378626	10.678316
HT-1376	907066	urinary_tract	carcinoma	urogenital_system	Bladder	0.843686	0.831439	0.07804	2.807466	4.53584	5.903533	7.543135	0.954536	0.0037	991412	3.117427	4.53584	7.260116
HT-144	907067	skin	malignant_melanoma	skin	melanoma	0.800084	0.619273	0.115202	1.641299	3.870697	5.866066	8.210175	0.901282	0.0172	1035911	2.636012	3.870697	6.317768
HT-3	907068	cervix	carcinoma	urogenital_system	cervix	0.813628	0.66652	0.10632	3.133848	5.607734	7.415032	9.591909	0.956765	0.0173	1101645	3.428145	5.607734	9.933045
HuCCT1	907069	biliary_tract	carcinoma	digestive_system	biliary_tract	0.803099	0.638389	0.070657	3.28263	5.581402	7.450762	9.695734	0.954641	0.0056	1035984	3.221313	5.581402	9.340114
HuH-7	907071	liver	carcinoma	digestive_system	liver	0.779407	0.636603	0.078251	2.572321	4.817431	6.789241	9.100363	0.938188	0.0051	1101649	3.244419	4.817431	7.638986
HuO9	907072	bone	osteosarcoma	bone	osteosarcoma	0.83604	0.948371	0.103511	3.371659	5.095968	6.198963	7.701846	0.978745	0.0014	1049471	3.160352	5.095968	8.994993
HUTU-80	907073	small_intestine	carcinoma	digestive_system	digestive_system_other	0.825543	0.757084	0.06532	2.996424	4.84656	6.401227	8.234797	0.962853	0.0015	991360	3.396816	4.84656	7.40847
IGR-1	907169	skin	malignant_melanoma	skin	melanoma	0.754176	0.528895	0.130699	0.011656	2.555003	5.152925	7.982136	0.825561	0.0074	1035985	1.259583	2.555003	4.403508
IMR-5	907170	autonomic_ganglia	neuroblastoma	nervous_system	neuroblastoma	0.850251	1.086114	0.092157	5.60759	7.046047	8.017087	9.310437	0.99803	0.0104	1309270	4.270355	7.046047	11.10702
IPC-298	907171	skin	malignant_melanoma	skin	melanoma	0.848998	0.927035	0.11691	3.065364	4.794409	5.924626	7.444662	0.968204	0.0057	1035987	3.068263	4.794409	8.50737
IST-MEL1	907172	skin	malignant_melanoma	skin	melanoma	0.594862	0.20046	0.084946	-0.003474	7.946962	16.050603	25.792722	0.918601	0.0117	1035912	1.532929	7.946962	17.531826
IST-MES1	907173	pleura	mesothelioma	lung	mesothelioma	0.840878	0.906585	0.061258	3.244976	4.910767	6.104127	7.623907	0.970828	0.0018	1035913	3.374559	4.910767	8.027219
JAR	907175	placenta	choriocarcinoma	urogenital_system	urogenital_system_other	0.843862	1.04361	0.088583	4.716448	6.15853	7.254115	8.609113	0.993535	0.0372	1035989	3.023236	6.15853	10.437716
JEG-3	907176	placenta	choriocarcinoma	urogenital_system	urogenital_system_other	0.843764	1.002325	0.113006	3.753062	5.438788	6.481384	7.921796	0.98444	0.0066	1049382	3.2606	5.438788	9.604584
JiyoyeP-2003	907268	haematopoietic_and_lymphoid_tissue	lymphoid_neoplasm	blood	Burkitt_lymphoma	0.85107	1.064259	0.087601	5.621898	7.086636	8.125875	9.449309	0.997606	0.0051	1324520	4.342057	7.086636	11.255728
JVM-2	907269	haematopoietic_and_lymphoid_tissue	lymphoid_neoplasm	blood	lymphoid_neoplasm_other	0.833758	1.039563	0.070688	5.242585	6.802138	7.836189	9.217904	0.996216	0.0039	1258648	4.123811	6.802138	10.995215
JVM-3	907270	haematopoietic_and_lymphoid_tissue	lymphoid_neoplasm	blood	lymphoid_neoplasm_other	0.8412	1.039276	0.117113	4.835653	6.391577	7.381369	8.729619	0.993736	0.0204	1257192	3.682782	6.391577	10.871678
KALS-1	907271	central_nervous_system	glioma	nervous_system	glioma	0.727173	0.404261	0.113571	1.063921	4.648128	8.023697	11.995137	0.900507	0.0118	1035916	2.692015	4.648128	8.203505
KARPAS-45	907272	haematopoietic_and_lymphoid_tissue	haematopoietic_neoplasm	blood	lymphoblastic_leukemia	0.848586	1.063019	0.089015	5.608366	7.077173	8.106645	9.432322	0.997593	0.0119	1265984	4.27536	7.077173	11.433494
KARPAS-299	907273	haematopoietic_and_lymphoid_tissue	lymphoid_neoplasm	blood	anaplastic_large_cell_lymphoma	0.819566	0.757272	0.101636	3.457659	5.564899	7.171124	9.072569	0.964869	0.0103	1254756	3.487017	5.564899	9.088811
KARPAS-422	907274	haematopoietic_and_lymphoid_tissue	lymphoid_neoplasm	blood	B_cell_lymphoma	0.852961	1.064704	0.069904	5.53395	7.029945	8.068172	9.401502	0.99665	0.0022	1267300	4.230673	7.029945	11.225558
KASUMI-1	907275	haematopoietic_and_lymphoid_tissue	haematopoietic_neoplasm	blood	acute_myeloid_leukaemia	0.849529	1.041455	0.103418	5.030543	6.54466	7.594519	8.960485	0.995591	0.0131	1266068	3.830308	6.54466	10.719889
KE-37	907277	haematopoietic_and_lymphoid_tissue	haematopoietic_neoplasm	blood	lymphoblastic_leukemia	0.861637	1.070119	0.101168	5.560699	7.087575	8.028647	9.34246	0.997573	0.0068	1261616	4.223431	7.087575	11.724926
KG-1	907278	haematopoietic_and_lymphoid_tissue	haematopoietic_neoplasm	blood	acute_myeloid_leukaemia	0.86297	1.070901	0.068579	5.612342	7.086698	8.057651	9.345897	0.997595	0.0036	1308990	4.26188	7.086698	11.523535
KINGS-1	907279	central_nervous_system	glioma	nervous_system	glioma	0.861351	0.960114	0.118449	3.2639	4.99475	6.024792	7.472721	0.975483	0.0078	1049383	3.05781	4.99475	9.193361
KMOE-2	907280	haematopoietic_and_lymphoid_tissue	haematopoietic_neoplasm	blood	acute_myeloid_leukaemia	0.85166	0.875674	0.092372	3.271661	5.10454	6.360721	7.951451	0.971038	0.0014	1254868	3.265538	5.10454	8.829561
KNS-42	907282	central_nervous_system	glioma	nervous_system	glioma	0.840887	0.950233	0.079493	4.653768	6.393347	7.551631	9.050065	0.990232	0.0037	1035917	3.980008	6.393347	10.818788
HuP-T3	907285	pancreas	carcinoma	pancreas	pancreas	0.845746	0.998858	0.075901	5.370486	6.95795	8.067933	9.469993	0.996062	0.0037	991415	4.23564	6.95795	11.181363
HuP-T4	907286	pancreas	carcinoma	pancreas	pancreas	0.853372	0.984878	0.086139	3.56772	5.254339	6.269045	7.687637	0.98008	0.0146	1101650	3.31025	5.254339	9.305642
HT55	907287	large_intestine	carcinoma	digestive_system	large_intestine	0.838657	1.005936	0.099063	4.751547	6.340033	7.435896	8.862884	0.992936	0.0206	1101646	3.788262	6.340033	10.342293
H-EMC-SS	907290	bone	chondrosarcoma	bone	chondrosarcoma	0.705511	0.377347	0.115587	1.311474	5.269188	9.02361	13.48472	0.899888	0.0111	1049453	3.008006	5.269188	9.384316
GP5d	907291	large_intestine	carcinoma	digestive_system	large_intestine	0.825573	0.848359	0.067441	4.148794	6.017902	7.383546	9.062238	0.982994	0.0039	991395	3.871046	6.017902	9.663136
DMS-53	907295	lung	carcinoma	lung	lung_small_cell_carcinoma	0.841632	0.888354	0.156149	2.604426	4.4165	5.616909	7.201805	0.952064	0.0306	1049427	2.698415	4.4165	8.192093
G-402	907298	soft_tissue	leiomyoblastoma	soft_tissue	soft_tissue_other	0.828814	0.85839	0.071842	1.572081	3.019993	4.468009	6.047947	0.907451	0.004	1035964	2.481073	3.019993	3.861165
G-401	907299	kidney	rhabdoid_tumour	kidney	kidney	0.853911	1.016696	0.080313	4.95269	6.576646	7.604722	9.002052	0.993583	0.0017	1049433	3.947675	6.576646	11.005819
KY821	907300	haematopoietic_and_lymphoid_tissue	haematopoietic_neoplasm	blood	leukemia	0.817583	1.05267	0.085463	5.421165	6.942513	7.985539	9.359965	0.99655	0.0102	1265424	4.183408	6.942513	11.199865
KU812	907311	haematopoietic_and_lymphoid_tissue	haematopoietic_neoplasm	blood	chronic_myeloid_leukaemia	0.851806	1.047898	0.100986	5.029333	6.546129	7.560905	8.91145	0.99591	0.011	1265872	3.912612	6.546129	10.819631
KU-19-19	907312	urinary_tract	carcinoma	urogenital_system	Bladder	0.875089	0.966097	0.07188	1.889689	3.206455	4.405232	5.764689	0.923806	0.002	991428	2.598263	3.206455	4.433576
KS-1	907313	central_nervous_system	glioma	nervous_system	glioma	0.827905	0.998284	0.122415	5.08103	6.627422	7.770421	9.216708	0.994414	0.029	1035918	3.431782	6.627422	10.928026
KP-N-YN	907314	autonomic_ganglia	neuroblastoma	nervous_system	neuroblastoma	0.831705	0.719736	0.163246	0.633741	2.414522	4.107518	6.026168	0.850951	0.0156	999996	1.524194	2.414522	4.026435
KYSE-150	907317	oesophagus	carcinoma	aero_digestive_tract	oesophagus	0.846161	0.977606	0.098493	4.397717	6.111905	7.172737	8.638182	0.989768	0.0018	1049473	3.685144	6.111905	10.54825
KYSE-180	907318	oesophagus	carcinoma	aero_digestive_tract	oesophagus	0.67161	0.214599	0.085773	-2.887182	3.648673	10.273006	17.992553	0.866942	0.0062	1000008	0.768163	3.648673	8.661299
KYSE-270	907319	oesophagus	carcinoma	aero_digestive_tract	oesophagus	0.80607	0.626519	0.105932	1.892623	4.178532	6.127693	8.425841	0.911139	0.0137	1000009	2.558363	4.178532	6.978724
KYSE-450	907320	oesophagus	carcinoma	aero_digestive_tract	oesophagus	0.77161	0.48391	0.073044	1.058064	3.75477	6.50585	9.559461	0.874758	0.0024	991431	2.755206	3.75477	5.36599
KYSE-510	907321	oesophagus	carcinoma	aero_digestive_tract	oesophagus	0.851559	0.926823	0.076525	4.769463	6.494011	7.65726	9.169235	0.991074	0.0124	1036001	3.877232	6.494011	10.653554
L-428	907322	haematopoietic_and_lymphoid_tissue	lymphoid_neoplasm	blood	Hodgkin_lymphoma	0.845828	1.085293	0.089789	5.925182	7.345443	8.375767	9.680725	0.997909	0.015	1262064	4.371081	7.345443	11.541505
L-540	907323	haematopoietic_and_lymphoid_tissue	lymphoid_neoplasm	blood	Hodgkin_lymphoma	0.854413	1.026181	0.107741	5.101849	6.679013	7.684081	9.062006	0.995705	0.0029	1255428	3.972209	6.679013	11.109597
J-RT3-T3-5	907391	haematopoietic_and_lymphoid_tissue	lymphoid_neoplasm	blood	lymphoblastic_T_cell_leukaemia	0.836059	0.731575	0.105891	2.296697	4.231344	5.830865	7.717345	0.93815	0.0028	1254644	2.867276	4.231344	6.73852
LAMA-84	907783	haematopoietic_and_lymphoid_tissue	haematopoietic_neoplasm	blood	chronic_myeloid_leukaemia	0.814451	0.884639	0.097984	4.173406	6.04001	7.348772	8.990041	0.98458	0.0078	1254980	3.734281	6.04001	9.990162
LC-2-ad	907786	lung	carcinoma	lung	lung_NSCLC_adenocarcinoma	0.688484	0.340687	0.092876	1.161764	5.365795	9.393427	14.166465	0.893165	0.0071	1035925	3.258017	5.365795	9.398821
LC4-1	907787	haematopoietic_and_lymphoid_tissue	haematopoietic_neoplasm	blood	lymphoblastic_leukemia	0.828322	1.039896	0.081537	5.233633	6.781359	7.842587	9.231893	0.996331	0.0023	1266964	4.051467	6.781359	11.059155
LNCaP-Clone-FGC	907788	prostate	carcinoma	urogenital_system	prostate	0.765856	0.540564	0.071611	1.868141	4.41546	6.775563	9.518519	0.907602	0.0127	1000011	3.062183	4.41546	6.927503
LOUCY	907789	haematopoietic_and_lymphoid_tissue	lymphoid_neoplasm	blood	lymphoblastic_T_cell_leukaemia	0.847643	1.103601	0.086065	5.823778	7.259186	8.236815	9.500176	0.998051	0.0151	1267524	4.444577	7.259186	11.704237
LoVo	907790	large_intestine	carcinoma	digestive_system	large_intestine	0.867111	0.940365	0.144095	2.624483	4.307996	5.423136	6.890926	0.958989	0.0095	1049482	2.67743	4.307996	7.69959
LP-1	907791	haematopoietic_and_lymphoid_tissue	lymphoid_neoplasm	blood	Myeloma	0.855265	1.06165	0.110261	5.096962	6.639877	7.577704	8.898781	0.996248	0.0239	1324072	3.852489	6.639877	11.302871
LS-123	907792	large_intestine	carcinoma	digestive_system	large_intestine	0.84396	0.885479	0.115502	3.305289	5.134732	6.34611	7.943981	0.971086	0.0173	991434	3.213039	5.134732	8.908647
LS-411N	907794	large_intestine	carcinoma	digestive_system	large_intestine	0.843482	1.08718	0.108075	5.715862	7.173483	8.140643	9.424284	0.99777	0.0185	1035927	4.323886	7.173483	11.478799
LS-513	907795	large_intestine	carcinoma	digestive_system	large_intestine	0.772372	0.599823	0.07748	3.580427	6.254415	8.318752	10.839622	0.956702	0.0076	991366	3.726371	6.254415	10.610542
LU-99A	907796	lung	carcinoma	lung	lung_NSCLC_large_cell	0.838754	0.943638	0.097473	4.507221	6.261036	7.406539	8.91143	0.989394	0.0057	991436	3.722627	6.261036	10.448007
MC116	907799	haematopoietic_and_lymphoid_tissue	lymphoid_neoplasm	blood	B_cell_lymphoma	0.853018	1.06317	0.083524	5.778827	7.277447	8.259832	9.572381	0.997825	0.0078	1255652	4.40348	7.277447	11.790375
MC-IXC	908118	autonomic_ganglia	neuroblastoma	nervous_system	neuroblastoma	0.856141	1.003599	0.158119	4.438822	6.13252	7.120799	8.546319	0.991455	0.018	1049485	3.438257	6.13252	10.950658
MDA-MB-175-VII	908120	breast	carcinoma	breast	breast	0.829988	0.821341	0.132484	3.326565	5.34962	6.709622	8.462385	0.967907	0.0137	1101665	3.257682	5.34962	9.491464
MDA-MB-361	908121	breast	carcinoma	breast	breast	0.814264	0.752694	0.109972	2.535648	4.503853	6.142775	8.051859	0.944765	0.0106	1036009	2.560232	4.503853	7.541602
MDA-MB-453	908122	breast	carcinoma	breast	breast	0.838375	0.873163	0.157234	2.606502	4.486694	5.716484	7.377409	0.950055	0.0765	1036010	2.746331	4.486694	8.387105
MDA-MB-468	908123	breast	carcinoma	breast	breast	0.794771	0.522917	0.10738	0.80368	3.311886	5.734994	8.483823	0.864081	0.0061	1101747	2.315008	3.311886	5.16761
MEL-HO	908124	skin	malignant_melanoma	skin	melanoma	0.845689	0.878757	0.092603	3.385991	5.177575	6.599096	8.215534	0.96834	0.0108	1036012	2.134878	5.177575	9.512244
MEL-JUSO	908125	skin	malignant_melanoma	skin	melanoma	0.817851	0.75002	0.133819	2.684439	4.82737	6.406656	8.350383	0.95146	0.021	1049486	3.008491	4.82737	8.35679
MEG-01	908126	haematopoietic_and_lymphoid_tissue	haematopoietic_neoplasm	blood	chronic_myeloid_leukaemia	0.862207	1.104745	0.076798	6.184855	7.598163	8.572221	9.83679	0.999011	0.0135	1257668	4.612789	7.598163	11.937494
MES-SA	908127	soft_tissue	sarcoma	urogenital_system	uterus	0.752994	0.508181	0.09257	1.053592	3.911874	6.633722	9.660458	0.87704	0.0117	1233350	1.836636	3.911874	7.088798
Mewo	908128	skin	malignant_melanoma	skin	melanoma	0.794287	0.496662	0.129777	-0.950519	1.648864	4.25154	7.160514	0.787587	0.0128	1036014	0.784011	1.648864	3.109528
MFE-280	908129	endometrium	carcinoma	urogenital_system	endometrium	0.857637	1.040382	0.095233	5.489412	7.029485	8.036733	9.385518	0.996484	0.0156	991441	4.212234	7.029485	11.481689
MFE-296	908130	endometrium	carcinoma	urogenital_system	endometrium	0.718626	0.387166	0.097166	1.534281	5.220047	8.757848	12.918403	0.90032	0.0092	1311544	3.17238	5.220047	8.971438
MG-63	908131	bone	osteosarcoma	bone	osteosarcoma	0.853251	0.906454	0.116539	2.759748	4.490082	5.6752	7.207136	0.963995	0.0028	1049488	2.863495	4.490082	7.888791
MHH-CALL-2	908132	haematopoietic_and_lymphoid_tissue	lymphoid_neoplasm	blood	B_cell_leukemia	0.848336	1.042573	0.141988	5.007967	6.578616	7.551312	8.912673	0.995981	0.0353	1323820	3.823336	6.578616	11.063801
MHH-ES-1	908134	bone	Ewings_sarcoma-peripheral_primitive_neuroectodermal_tumour	bone	ewings_sarcoma	0.742899	0.463518	0.117739	0.577908	3.484822	6.388958	9.722397	0.867851	0.0054	1049489	2.272677	3.484822	5.633392
MHH-NB-11	908135	autonomic_ganglia	neuroblastoma	nervous_system	neuroblastoma	0.833166	0.648415	0.122287	0.357592	2.322204	4.200174	6.297685	0.824497	0.0346	991444	1.694013	2.322204	3.668424

MHH-PREB-1	908136	haematopoietic_and_lymphoid_tissue	lymphoid_neoplasm	blood	B_cell_leukemia	0.833914	1.104492	0.102137	5.870512	7.271695	8.28274	9.579243	0.997918	0.033	1307758	4.294633	7.271695	11.479927
MKN1	908138	stomach	carcinoma	digestive_system	stomach	0.850897	1.02633	0.104131	4.858562	6.421529	7.442356	8.814826	0.994186	0.0066	1049490	3.852055	6.421529	10.699952
MKN28	908139	stomach	carcinoma	digestive_system	stomach	0.83657	0.903821	0.143636	3.538453	5.344255	6.532847	8.089425	0.978072	0.0253	1049491	2.978285	5.344255	9.723266
ML-2	908141	haematopoietic_and_lymphoid_tissue	haematopoietic_neoplasm	blood	acute_myeloid_leukaemia	0.854879	1.087538	0.092296	5.814068	7.222802	8.20199	9.474418	0.997948	0.0159	1323204	4.338106	7.222802	11.457855
MLMA	908142	haematopoietic_and_lymphoid_tissue	lymphoid_neoplasm	blood	hairy_cell_leukaemia	0.836677	0.877267	0.10109	3.791406	5.701199	6.960578	8.605	0.980672	0.0076	991447	3.479097	5.701199	9.9708
MN-60	908143	haematopoietic_and_lymphoid_tissue	lymphoid_neoplasm	blood	B_cell_leukemia	0.843289	1.072395	0.091822	5.644004	7.116755	8.116966	9.450343	0.997609	0.0091	1309606	4.299435	7.116755	11.394655
MOLT-13	908146	haematopoietic_and_lymphoid_tissue	lymphoid_neoplasm	blood	lymphoblastic_T_cell_leukaemia	0.851574	1.053081	0.070647	5.371144	6.919176	7.920522	9.270836	0.996253	0.0056	1264668	4.144379	6.919176	11.40213
MOLT-16	908147	haematopoietic_and_lymphoid_tissue	lymphoid_neoplasm	blood	lymphoblastic_T_cell_leukaemia	0.856546	1.014265	0.15838	4.432899	6.10485	7.057226	8.457392	0.992621	0.0408	1267608	3.308013	6.10485	11.05805
MONO-MAC-6	908148	haematopoietic_and_lymphoid_tissue	haematopoietic_neoplasm	blood	acute_myeloid_leukaemia	0.860105	1.078182	0.084165	5.202216	6.720489	7.663906	8.961826	0.996368	0.012	1262288	3.937742	6.720489	11.26611
Mo-T	908149	haematopoietic_and_lymphoid_tissue	lymphoid_neoplasm	blood	hairy_cell_leukaemia	0.81339	0.68048	0.116118	1.867634	3.950999	5.72247	7.818239	0.919251	0.009	1255092	2.649263	3.950999	6.534855
MPP-89	908150	pleura	mesothelioma	lung	mesothelioma	0.839693	0.966745	0.083459	4.940805	6.627853	7.785038	9.273274	0.994266	0.0111	1035928	3.963231	6.627853	10.913878
MRK-nu-1	908151	breast	carcinoma	breast	breast	0.843977	1.032027	0.08424	5.109288	6.671088	7.723519	9.102028	0.995862	0.0033	1265536	4.04617	6.671088	10.989504
MSTO-211H	908152	pleura	mesothelioma	lung	mesothelioma	0.851314	0.959885	0.124752	4.025759	5.793254	6.876832	8.353267	0.986772	0.0106	1000012	3.352782	5.793254	10.391106
MV-4-11	908156	haematopoietic_and_lymphoid_tissue	haematopoietic_neoplasm	blood	leukemia	0.825828	0.761961	0.112796	1.854494	3.687008	5.230146	7.054982	0.919617	0.007	1255204	2.564121	3.687008	5.928528
NALM-6	908158		lymphoid neoplasm	blood	B_cell_leukemia	0.848786	1.033195	0.098555	4.472076	6.06628	7.031341	8.394573	0.992822	0.0054	1323344	3.551137	6.06628	10.279472
NB69	908440	autonomic_ganglia	neuroblastoma	nervous_system	neuroblastoma	0.831782	0.954813	0.116212	4.771861	6.456221	7.631163	9.129003	0.992342	0.0101	991449	3.655037	6.456221	10.675157
NCI-H520	908443	lung	carcinoma	lung	lung_NSCLC_squamous_cell_carcinoma	0.857022	1.065875	0.074989	4.752237	6.305745	7.250989	8.563993	0.993537	0.0302	1036033	3.40311	6.305745	11.017849
NCI-SNU-1	908444	stomach	carcinoma	digestive_system	stomach	0.850962	0.970646	0.10304	3.097167	4.729991	5.791527	7.229342	0.973688	0.0067	1323372	3.053601	4.729991	8.160086
NCI-SNU-5	908445	stomach	carcinoma	digestive_system	stomach	0.851052	1.016451	0.105748	5.306463	6.806131	7.921061	9.298087	0.996153	0.0109	1255316	3.873394	6.806131	10.940091
NCI-SNU-16	908446	stomach	carcinoma	digestive_system	stomach	0.844402	1.032836	0.118022	5.034549	6.566514	7.611628	8.977386	0.995204	0.0072	1323064	3.861695	6.566514	10.854405
NH-12	908447	autonomic_ganglia	neuroblastoma	nervous_system	neuroblastoma	0.841198	0.900262	0.105743	3.558576	5.352403	6.579014	8.177946	0.976375	0.0096	1049519	3.013091	5.352403	9.378683
NKM-1	908448	haematopoietic_and_lymphoid_tissue	haematopoietic_neoplasm	blood	acute_myeloid_leukaemia	0.842375	1.061516	0.107455	5.318142	6.798844	7.831564	9.160449	0.996126	0.0167	1256660	4.036601	6.798844	11.048687
NMC-G1	908449	central_nervous_system	glioma	nervous_system	glioma	0.876906	1.043518	0.134093	2.425122	4.05893	4.888176	6.192827	0.956565	0.0098	1049399	2.54517	4.05893	8.079193
no-11	908450	central_nervous_system	glioma	nervous_system	glioma	0.846121	1.03323	0.12846	4.849599	6.437472	7.428673	8.79368	0.99394	0.0246	1049402	3.672591	6.437472	11.121453
NOMO-1	908451	haematopoietic_and_lymphoid_tissue	haematopoietic_neoplasm	blood	acute_myeloid_leukaemia	0.838813	1.062776	0.11095	5.435304	6.943368	7.980497	9.325058	0.99658	0.0114	1256548	4.093243	6.943368	11.308847
no-10	908452	central_nervous_system	glioma	nervous_system	glioma	0.880182	1.033225	0.111584	1.881723	3.194713	4.239588	5.515826	0.933601	0.0057	1049401	2.386383	3.194713	5.071596
NTERA-S-cl-D1	908454	testis	germ_cell_tumour	urogenital_system	testis	0.767954	0.499525	0.077535	2.082616	4.936926	7.487151	10.50016	0.915933	0.0074	1035935	3.168674	4.936926	8.362148
NUGC-3	908455	stomach	carcinoma	digestive_system	stomach	0.856862	1.040485	0.122129	5.250204	6.777843	7.816175	9.166161	0.996514	0.0502	1049520	3.882518	6.777843	11.217571
NCI-H747	908457	large_intestine	carcinoma	digestive_system	large_intestine	0.837663	0.868843	0.083641	3.977423	5.859779	7.162001	8.813186	0.981498	0.008	991370	3.687603	5.859779	9.936583
NCI-H716	908458	large_intestine	carcinoma	digestive_system	large_intestine	0.828492	0.965361	0.104695	4.564362	6.302407	7.445296	8.958348	0.989809	0.0031	1256856	3.753252	6.302407	10.66555
NCI-H596	908459	lung	carcinoma	lung	lung_NSCLC_adenocarcinoma	0.847153	1.054903	0.0861	5.347285	6.873561	7.861474	9.178761	0.996199	0.007	1049510	4.166981	6.873561	11.364431
NCI-H441	908460	lung	carcinoma	lung	lung_NSCLC_adenocarcinoma	0.799917	0.59322	0.068568	3.198946	5.623074	7.72305	10.201365	0.949869	0.0037	991461	3.635681	5.623074	8.992852
NCI-N87	908461	stomach	carcinoma	digestive_system	stomach	0.810748	0.670952	0.078463	3.066875	5.204695	7.051861	9.171672	0.955713	0.005	1036034	3.166494	5.204695	8.282948
NCI-H2452	908462	pleura	mesothelioma	lung	mesothelioma	0.842889	0.866253	0.071777	3.816248	5.62334	6.94153	8.552094	0.980061	0.004	1000019	3.627581	5.62334	9.133338
NCI-H1793	908463	lung	carcinoma	lung	lung_NSCLC_adenocarcinoma	0.856901	0.982429	0.126898	2.262905	4.125845	5.033069	6.510825	0.942299	0.0192	1000017	2.188457	4.125845	8.856156
NCI-H358	908465	lung	carcinoma	lung	lung_NSCLC_adenocarcinoma	0.820223	0.814645	0.11333	3.093202	5.037909	6.459184	8.227668	0.961101	0.0155	1036032	3.232638	5.037909	8.665653
NCI-H1155	908467	lung	carcinoma	lung	lung_NSCLC_large_cell	0.83889	0.965875	0.068579	4.162176	5.861851	6.952882	8.430636	0.987974	0.0012	1262624	3.710457	5.861851	9.885879
NCI-H28	908470	pleura	mesothelioma	lung	mesothelioma	0.841189	0.849446	0.131025	2.290554	4.06696	5.360254	7.007912	0.952544	0.0254	1049441	2.526913	4.06696	7.287314
NCI-H1581	908471	lung	carcinoma	lung	lung_NSCLC_large_cell	0.753143	0.44253	0.109151	0.715512	3.752831	6.744216	10.170495	0.874802	0.0066	1000014	2.445273	3.752831	6.087154
NCI-H1573	908472	lung	carcinoma	lung	lung_NSCLC_adenocarcinoma	0.844501	0.879741	0.075052	3.177219	4.834832	6.139497	7.697464	0.967257	0.0038	991452	3.228559	4.834832	7.692371
NCI-H1666	908473	lung	carcinoma	lung	lung_NSCLC_adenocarcinoma	0.655916	0.300548	0.064544	1.769505	6.517129	11.232174	16.829373	0.9115	0.0049	1036020	3.477933	6.517129	11.245278
NCI-H1703	908474	lung	carcinoma	lung	lung_NSCLC_adenocarcinoma	0.61664	0.21848	0.096976	-0.339161	6.461963	13.296066	21.473578	0.901573	0.0094	1049499	2.7288	6.461963	12.769119
NCI-H1755	908475	lung	carcinoma	lung	lung_NSCLC_adenocarcinoma	0.833037	0.958482	0.141391	3.634755	5.321315	6.463841	7.969407	0.980955	0.0482	1000016	2.668031	5.321315	9.599424
NCI-H1993	908476	lung	carcinoma	lung	lung_NSCLC_adenocarcinoma	0.838267	1.081983	0.083434	5.88412	7.335228	8.333028	9.653902	0.997748	0.0138	1000018	4.491096	7.335228	11.732657
NCI-H630	908482	large_intestine	carcinoma	digestive_system	large_intestine	0.846844	1.057285	0.05948	4.798327	6.390437	7.317682	8.657134	0.993604	0.0016	1000021	3.932492	6.390437	10.82336
NCI-H524	908483	lung	carcinoma	lung	lung_small_cell_carcinoma	0.861202	1.080295	0.103208	5.572352	7.067014	8.017905	9.318688	0.99762	0.0093	1263996	4.217976	7.067014	11.566882
ONS-76	909248	central_nervous_system	primitive_neuroectodermal_tumour-medulloblastoma	nervous_system	medulloblastoma	0.837411	1.022906	0.096651	4.859701	6.415409	7.495066	8.894523	0.993682	0.0028	1049405	3.906053	6.415409	10.438146
OPM-2	909249	haematopoietic_and_lymphoid_tissue	haematopoietic_neoplasm	blood	Myeloma	0.845624	1.052334	0.066746	5.067295	6.640792	7.623508	8.973116	0.996017	0.0012	1266740	3.984873	6.640792	11.065813
OS-RC-2	909250	kidney	carcinoma	kidney	kidney	0.837374	0.859359	0.052794	4.681266	6.552673	7.862625	9.500958	0.988932	0.005	1035936	4.186537	6.552673	10.506766
P12-ICHIKAWA	909251	haematopoietic_and_lymphoid_tissue	lymphoid_neoplasm	blood	lymphoblastic_T_cell_leukaemia	0.847823	1.053966	0.090743	4.914396	6.474711	7.448279	8.792545	0.995257	0.0032	1265760	3.871072	6.474711	10.870048
P30-OHK	909252	haematopoietic_and_lymphoid_tissue	lymphoid_neoplasm	blood	lymphoblastic_leukemia	0.860543	1.106727	0.095878	5.78918	7.161872	8.148861	9.395014	0.997949	0.0326	1307870	4.231018	7.161872	11.374335
P31-FUJ	909253	haematopoietic_and_lymphoid_tissue	haematopoietic_neoplasm	blood	acute_myeloid_leukaemia	0.840373	1.077851	0.086194	5.78349	7.244611	8.248274	9.56731	0.997901	0.0153	1261840	4.349956	7.244611	11.550433
PA-1	909255	ovary	germ_cell_tumour	urogenital_system	ovary	0.823577	0.797229	0.115236	1.456713	3.085633	4.625741	6.350468	0.894746	0.0157	1036042	2.193947	3.085633	4.528523
OCUB-M	909256	breast	carcinoma	breast	breast	0.807145	0.681262	0.080335	2.789867	4.944432	6.679426	8.790808	0.950362	0.0065	999998	3.262214	4.944432	8.143454
OC-314	909257	ovary	carcinoma	urogenital_system	ovary	0.841394	0.935842	0.057022	4.794425	6.507031	7.731314	9.249562	0.991116	0.0018	1049403	4.117226	6.507031	10.373313
PF-382	909260	haematopoietic_and_lymphoid_tissue	lymphoid_neoplasm	blood	lymphoblastic_T_cell_leukaemia	0.858692	1.080974	0.093843	5.693174	7.147801	8.118917	9.401526	0.997902	0.0119	1262176	4.3241	7.147801	11.499938
Raji	909262	haematopoietic_and_lymphoid_tissue	lymphoid_neoplasm	blood	Burkitt_lymphoma	0.830793	1.088508	0.106423	5.800592	7.23022	8.220329	9.514832	0.997912	0.0216	1324324	4.388344	7.23022	11.441442
RCM-1	909263	large_intestine	carcinoma	digestive_system	large_intestine	0.841613	1.01981	0.117635	4.648465	6.195945	7.258988	8.647583	0.992743	0.0065	1049326	3.680008	6.195945	10.247807
RD	909264	soft_tissue	rhabdomyosarcoma	soft_tissue	rhabdomyosarcoma	0.853963	0.890533	0.100841	1.384681	3.451173	4.326729	5.953715	0.901529	0.004	1049327	1.901592	3.451173	7.9178
REH	909696	haematopoietic_and_lymphoid_tissue	lymphoid_neoplasm	blood	B_cell_leukemia	0.849188	1.056927	0.073846	4.943641	6.509394	7.46479	8.791692	0.99547	0.0041	1261952	3.929783	6.509394	10.920232
RKO	909698	large_intestine	carcinoma	digestive_system	large_intestine	0.690496	0.360262	0.081274	1.733128	6.118484	9.790908	14.431845	0.906938	0.0132	1049406	2.989558	6.118484	12.331218
RMG-I	909699	ovary	carcinoma	urogenital_system	ovary	0.83925	1.033919	0.183259	5.165561	6.709284	7.749292	9.132739	0.996423	0.0497	1101549	3.843576	6.709284	11.242633
RPMI-2650	909700	upper_aerodigestive_tract	carcinoma	aero_digestive_tract	head_and_neck	0.833664	0.981168	0.069702	4.426995	6.105193	7.208505	8.672369	0.98939	0.0038	999980	3.822737	6.105193	10.073732
RPMI-6666	909701	haematopoietic_and_lymphoid_tissue	lymphoid_neoplasm	blood	Hodgkin_lymphoma	0.833679	1.040755	0.102827	5.516383	7.003385	8.082598	9.42877	0.996668	0.0049	1323708	4.152262	7.003385	11.109068
RPMI-8402	909702	haematopoietic_and_lymphoid_tissue	lymphoid_neoplasm	blood	lymphoblastic_T_cell_leukaemia	0.842639	1.087935	0.091996	5.823915	7.243672	8.264368	9.564703	0.997925	0.0215	1306974	4.139676	7.243672	11.591596
RS4-11	909703	haematopoietic_and_lymphoid_tissue	haematopoietic_neoplasm	blood	leukemia	0.848257	1.060788	0.081746	4.172936	5.76036	6.689332	8.032922	0.989789	0.0092	1263268	3.3882	5.76036	10.052607
RT-112	909704	urinary_tract	carcinoma	urogenital_system	Bladder	0.840655	0.848309	0.074466	3.259159	5.061482	6.376678	8.030177	0.967867	0.0035	991318	3.337211	5.061482	8.258442
RVH-421	909706	skin	malignant_melanoma	skin	melanoma	0.796849	0.637538	0.081903	2.981617	5.449373	7.475228	9.806327	0.942492	0.0135	991319	2.953482	5.449373	9.373788
Saos-2	909707	bone	osteosarcoma	bone	osteosarcoma	0.843106	0.836835	0.073119	3.773532	5.661277	7.038547	8.71618	0.977956	0.0116	991321	3.662277	5.661277	9.240263
SAS	909708	upper_aerodigestive_tract	carcinoma	aero_digestive_tract	head_and_neck	0.837069	0.867196	0.106532	2.55649	4.239565	5.532976	7.137847	0.951852	0.0035	1049332	2.889423	4.239565	7.034735
SCC-9	909709	upper_aerodigestive_tract	carcinoma	aero_digestive_tract	head_and_neck	0.849592	1.002443	0.10897	5.165138	6.755977	7.843996	9.254804	0.995725	0.0156	1049336	4.048182	6.755977	11.069187
SCH	909711	stomach	other	digestive_system	stomach	0.804922	0.740329	0.118939	2.429886	4.753751	6.4254	8.439542	0.937369	0.0283	1049337	2.270564	4.753751	9.221743
SF126	909712	central_nervous_system	glioma	nervous_system	glioma	0.694482	0.316081	0.092553	-0.182738	4.167697	8.606999	13.75253	0.880811	0.006	1233330	2.543582	4.167697	7.092632
SH-4	909713	skin	malignant_melanoma	skin	melanoma	0.675354	0.273878	0.105449	0.36101	5.77061	10.994204	17.299802	0.892487	0.0089	1035941	3.134234	5.77061	10.927399
SJRH30	909716	soft_tissue	rhabdomyosarcoma	soft_tissue	rhabdomyosarcoma	0.784981	0.567251	0.081918	2.587685	5.165482	7.382931	10.02734	0.931899	0.0077	991322	3.33006	5.165482	8.600579
SJSA-1	909717	bone	other	bone	bone_other	0.862804	0.932822	0.097054	3.900579	5.706762	6.850539	8.375399	0.984122	0.0055	1035942	3.454909	5.706762	9.908104
SK-CO-1	909718	large_intestine	carcinoma	digestive_system	large_intestine	0.841288	0.84703	0.119628	2.719713	4.568724	5.899466	7.558147	0.958248	0.0054	1101745	2.91009	4.568724	8.062733
SK-HEP-1	909719	liver	carcinoma	digestive_system	liver	0.831227	0.758941	0.075372	2.180423	3.917776	5.491612	7.313628	0.93512	0.0029	1049340	2.938434	3.917776	5.716957
SK-LMS-1	909720	soft_tissue	sarcoma	soft_tissue	soft_tissue_other	0.836976	0.859989	0.143252	3.387617	5.322982	6.68659	8.377379	0.971597	0.0358	1233331	3.077736	5.322982	9.222085
SK-LU-1	909721	lung	carcinoma	lung	lung_NSCLC_adenocarcinoma	0.84325	0.944273	0.089541	3.533333	5.241272	6.372399	7.867933	0.980071	0.0077	1049341	3.336654	5.241272	9.015392
SKM-1	909722	haematopoietic_and_lymphoid_tissue	haematopoietic_neoplasm	blood	haematopoietic_neoplasm_other	0.840624	1.058672	0.168187	5.185386	6.663346	7.693055	9.039123	0.996336	0.0416	1255764	3.915089	6.663346	10.818091
SK-MEL-1	909723	skin	malignant_melanoma	skin	melanoma	0.846497	1.068739	0.115371	4.438307	5.999031	6.937993	8.274801	0.992979	0.0203	1324576	3.408585	5.999031	10.45326
SK-MEL-3	909724	skin	malignant_melanoma	skin	melanoma	0.754746	0.648131	0.108746	-0.844063	1.093471	3.158456	5.409777	0.739222	0.0055	1049343	0.60573	1.093471	1.646494
SK-MEL-24	909725	skin	malignant_melanoma	skin	melanoma	0.669182	0.252825	0.165927	-3.479396	2.055103	7.967958	14.780572	0.847237	0.0136	991372	0.139281	2.055103	4.86143
SK-MEL-30	909726	skin	malignant_melanoma	skin	melanoma	0.748467	0.446064	0.116278	1.459244	4.704746	7.711053	11.275047	0.894184	0.0177	991325	2.93801	4.704746	8.077865
SK-MES-1	909728	lung	carcinoma	lung	lung_NSCLC_squamous_cell_carcinoma	0.836209	0.992594	0.084695	4.71987	6.401081	7.457618	8.876307	0.992819	0.006	1049344	3.84327	6.401081	10.953315
SK-MG-1	909729	central_nervous_system	glioma	nervous_system	glioma	0.737041	0.431989	0.095802	1.836418	5.198899	8.33821	12.029207	0.911866	0.012	1233333	3.139707	5.198899	8.810615
SK-NEP-1	909730	kidney	Wilms_tumour	kidney	kidney	0.859932	1.042433	0.120817	5.246325	6.798142	7.802991	9.143828	0.996229	0.0088	1307198	3.94677	6.798142	11.339151
SK-PN-DW	909731	bone	Ewings_sarcoma-peripheral_primitive_neuroectodermal_tumour	bone	ewings_sarcoma	0.851798	1.030116	0.15828	5.251439	6.788045	7.826226	9.184439	0.996231	0.0303	1257304	3.987141	6.788045	11.1341
SK-UT-1	909732	soft_tissue	leiomyosarcoma	soft_tissue	soft_tissue_other	0.863373	0.906351	0.131266	2.049495	3.565626	4.829398	6.329174	0.940061	0.0063	1049412	2.506379	3.565626	5.620338
SNG-M	909735	endometrium	carcinoma	urogenital_system	endometrium	0.866704	0.764347	0.11243	0.880077	2.533891	4.069178	5.804556	0.855192	0.0267	1311572	1.902974	2.533891	3.796374
SNU-387	909736	liver	carcinoma	digestive_system	liver	0.780127	0.632337	0.120995	2.862144	5.725619	7.710014	10.159533	0.948284	0.0328	1101566	3.267128	5.725619	10.66566
SNU-423	909737	liver	carcinoma	digestive_system	liver	0.742795	0.489442	0.110337	1.775904	5.193579	8.028803	11.396296	0.905385	0.0347	1101568	2.55766	5.193579	9.903227

SNU-449	909738	liver	carcinoma	digestive_system	liver	0.861493	0.909957	0.065521	2.639388	4.207293	5.413436	6.891811	0.954514	0.0056	1101569	3.006611	4.207293	6.546543
SNU-C2B	909740	large_intestine	carcinoma	digestive_system	large_intestine	0.818794	0.733359	0.100259	2.512953	4.463942	6.107486	8.062164	0.947364	0.0027	991373	3.041911	4.463942	7.09047
SUP-T1	909743	haematopoietic_and_lymphoid_tissue	lymphoid_neoplasm	blood	lymphoblastic_T_cell_leukaemia	0.849753	1.054495	0.087361	5.147965	6.648275	7.659095	8.993449	0.996239	0.0038	1264892	4.03954	6.648275	10.700054
SW13	909744	adrenal_gland	adrenal_cortical_carcinoma	kidney	adrenal_gland	0.83353	1.027948	0.070587	5.425121	6.928591	8.024716	9.411108	0.996537	0.0069	999984	4.00054	6.928591	11.085974
SW1088	909745	central_nervous_system	glioma	nervous_system	glioma	0.802	0.803873	0.077526	4.446936	6.538734	8.111552	9.968963	0.986467	0.0214	1035864	3.446073	6.538734	11.081799
SW1116	909746	large_intestine	carcinoma	digestive_system	large_intestine	0.847058	1.066245	0.113743	5.805528	7.270725	8.275443	9.598629	0.997831	0.0296	1049348	4.340586	7.270725	11.666694
SW1417	909747	large_intestine	carcinoma	digestive_system	large_intestine	0.843976	1.066225	0.084885	5.587001	7.015403	8.036485	9.329813	0.997472	0.0159	1035865	4.148873	7.015403	11.141398
SW1463	909748	large_intestine	carcinoma	digestive_system	large_intestine	0.779245	0.567167	0.073492	2.562856	5.016929	7.220856	9.779649	0.931593	0.0061	991333	3.388767	5.016929	7.762384
SW1710	909749	urinary_tract	carcinoma	urogenital_system	Bladder	0.833717	0.809346	0.108147	2.541563	4.302095	5.72016	7.418832	0.953532	0.019	1035869	2.810646	4.302095	7.078185
SW1783	909750	central_nervous_system	glioma	nervous_system	glioma	0.817695	0.733486	0.15936	2.145264	4.213228	5.873663	7.854321	0.935261	0.0312	1035868	1.971402	4.213228	7.918783
SW48	909751	large_intestine	carcinoma	digestive_system	large_intestine	0.825782	0.842655	0.10954	2.560856	4.327446	5.672472	7.32943	0.947761	0.0026	1049350	2.923682	4.327446	7.285929
SW626	909753	ovary	carcinoma	urogenital_system	ovary	0.852578	1.012215	0.105067	4.621648	6.233033	7.294945	8.697556	0.992785	0.0134	1101573	3.672224	6.233033	10.546478
SW684	909754	soft_tissue	fibrosarcoma	soft_tissue	fibrosarcoma	0.847195	0.978511	0.126835	4.222972	5.913364	7.001386	8.482488	0.989628	0.0187	1049413	3.352378	5.913364	10.085592
SW837	909755	large_intestine	carcinoma	digestive_system	large_intestine	0.827732	0.953843	0.096268	4.805464	6.529376	7.728858	9.256494	0.992683	0.0032	991339	3.926365	6.529376	10.771424
SW872	909756	soft_tissue	liposarcoma	soft_tissue	soft_tissue_other	0.815878	0.763463	0.090049	3.46846	5.513243	7.091263	8.980854	0.965179	0.0064	1035945	3.407025	5.513243	9.043552
SW948	909757	large_intestine	carcinoma	digestive_system	large_intestine	0.856101	1.02091	0.060638	5.346765	6.923153	7.940147	9.319115	0.996055	0.0088	999985	4.26138	6.923153	11.344479
SW962	909758	vulva	carcinoma	urogenital_system	urogenital_system_other	0.846446	1.068502	0.091357	5.676938	7.082072	8.145451	9.453301	0.998151	0.0158	1049415	4.178657	7.082072	11.244122
SW982	909759	soft_tissue	synovial_sarcoma	soft_tissue	soft_tissue_other	0.829694	0.957083	0.119795	4.318466	6.018276	7.13094	8.624879	0.988843	0.0265	1035946	3.59091	6.018276	10.233844
T84	909761	large_intestine	carcinoma	digestive_system	large_intestine	0.847305	1.032615	0.096348	5.013565	6.548641	7.561713	8.937074	0.99563	0.0086	1049353	3.933817	6.548641	10.88673
TGBC1TKB	909769	biliary_tract	carcinoma	digestive_system	biliary_tract	0.834379	0.962278	0.109887	4.167087	5.935881	7.047322	8.575139	0.987723	0.0059	1035949	3.527277	5.935881	10.318433
TGBC11TKB	909770	stomach	carcinoma	digestive_system	stomach	0.839848	1.031742	0.093327	4.928344	6.459442	7.531951	8.90642	0.994065	0.0073	1324548	3.876283	6.459442	10.437348
THP-1	909771	haematopoietic_and_lymphoid_tissue	haematopoietic_neoplasm	blood	acute_myeloid_leukaemia	0.836052	1.032996	0.071814	4.913045	6.468942	7.484284	8.849256	0.993905	0.0021	1308822	3.989667	6.468942	10.614523
TUR	909773	haematopoietic_and_lymphoid_tissue	lymphoid_neoplasm	blood	B_cell_lymphoma	0.859309	1.077981	0.107777	5.244136	6.685713	7.677218	8.980838	0.996352	0.0258	1307534	3.979998	6.685713	10.730261
TYK-nu	909774	ovary	carcinoma	urogenital_system	ovary	0.863152	1.038235	0.058413	1.751584	2.927247	4.078079	5.314891	0.920004	0.0029	1101581	2.527853	2.927247	3.591843
U-2-OS	909776	bone	osteosarcoma	bone	osteosarcoma	0.852643	0.972011	0.0901	4.638744	6.262666	7.434443	8.895405	0.992314	0.0217	1035874	3.863796	6.262666	10.063011
U-698-M	909777	haematopoietic_and_lymphoid_tissue	lymphoid_neoplasm	blood	B_cell_leukemia	0.867012	1.091376	0.07017	4.873475	6.291208	7.311713	8.601535	0.994156	0.0042	1263072	2.726438	6.291208	10.973962
UACC-893	909778	breast	carcinoma	breast	breast	0.840923	1.005159	0.140613	5.18196	6.760774	7.879424	9.307797	0.995755	0.0168	1035875	4.012315	6.760774	11.029264
UMC-11	909779	lung	carcinoid-endocrine_tumour	lung	lung_NSCLC_carcinoid	0.754744	0.553187	0.117512	1.269373	3.692163	6.105979	8.832911	0.885744	0.0116	991345	2.439234	3.692163	5.665545
VM-CUB-1	909780	urinary_tract	carcinoma	urogenital_system	Bladder	0.631697	0.24453	0.101184	-0.25249	5.593771	11.599584	18.728329	0.889129	0.0069	1311600	2.976095	5.593771	10.043063
VMRC-RCZ	909781	kidney	carcinoma	kidney	kidney	0.850251	0.985022	0.134623	4.480093	6.162953	7.222843	8.678985	0.990095	0.0207	1049358	3.549075	6.162953	10.700263
WM-115	909784	skin	malignant_melanoma	skin	melanoma	0.813806	0.728599	0.106663	1.802665	3.647992	5.332798	7.279742	0.918987	0.0073	1049359	2.526295	3.647992	5.606748
WSU-NHL	909785	haematopoietic_and_lymphoid_tissue	lymphoid_neoplasm	blood	lymphoid_neoplasm_other	0.861334	1.069913	0.088682	3.423582	5.035558	6.023823	7.383738	0.983189	0.0042	1307646	3.086871	5.035558	8.819183
YAPC	909904	pancreas	carcinoma	pancreas	pancreas	0.811838	0.782116	0.068404	3.585984	5.534557	7.017835	8.834505	0.96971	0.0066	991351	3.617736	5.534557	8.87194
YH-13	909905	central_nervous_system	glioma	nervous_system	glioma	0.738533	0.432151	0.082535	2.698572	6.361065	9.348851	13.036532	0.929974	0.0097	1101606	3.576603	6.361065	11.940606
ZR-75-30	909907	breast	carcinoma	breast	breast	0.681695	0.319418	0.086753	-0.769464	3.588539	8.026798	13.158066	0.857708	0.0095	991352	1.940754	3.588539	6.411865
RCC10RGB	909974	kidney	carcinoma	kidney	kidney	0.847543	0.994036	0.067978	3.855883	5.507097	6.501974	7.907452	0.986639	0.0031	1035937	3.515241	5.507097	9.645387
KURAMOCHI	909975	ovary	carcinoma	urogenital_system	ovary	0.832908	0.942861	0.116058	4.13844	5.959898	7.122586	8.655046	0.987227	0.0274	1049388	3.44354	5.959898	10.47568
KM-H2	909976	haematopoietic_and_lymphoid_tissue	lymphoid_neoplasm	blood	Hodgkin_lymphoma	0.836178	1.030595	0.132723	5.097202	6.646822	7.703998	9.104489	0.99586	0.0132	1323176	3.911457	6.646822	10.916859
HuO-3N1	909977	bone	osteosarcoma	bone	osteosarcoma	0.854314	0.969788	0.115858	4.147484	5.878758	6.918569	8.3744	0.987335	0.0062	1049470	3.457394	5.878758	10.378778
OE19	910079	oesophagus	carcinoma	aero_digestive_tract	oesophagus	0.837985	1.004778	0.123295	4.59919	6.278258	7.331882	8.774809	0.992239	0.0241	1036038	3.628043	6.278258	10.948294
NCI-H838	910399	lung	carcinoma	lung	lung_NSCLC_adenocarcinoma	0.755231	0.479059	0.141838	-0.127013	2.828398	5.484782	8.650795	0.833125	0.0271	1101683	1.255671	2.828398	6.015157
Ramos-2G6-4C10	910401	haematopoietic_and_lymphoid_tissue	lymphoid_neoplasm	blood	Burkitt_lymphoma	0.850891	1.080336	0.126629	4.910331	6.397308	7.345534	8.645955	0.995311	0.0289	1256268	3.58554	6.397308	10.881571
RPMI-8866	910544	haematopoietic_and_lymphoid_tissue	haematopoietic_neoplasm	blood	chronic_myeloid_leukaemia	0.84946	1.049699	0.153425	3.054725	4.762443	5.615814	6.982173	0.973654	0.0143	1256436	2.705989	4.762443	9.120628
QIMR-WIL	910545	haematopoietic_and_lymphoid_tissue	haematopoietic_neoplasm	blood	acute_myeloid_leukaemia	0.844156	1.086241	0.07848	5.860909	7.323292	8.29594	9.593686	0.997807	0.0051	1256772	4.457301	7.323292	11.803157
PSN1	910546	pancreas	carcinoma	pancreas	pancreas	0.828769	0.900914	0.089368	4.372928	6.186673	7.440241	9.015545	0.988299	0.022	1035857	3.732744	6.186673	10.181299
OAW-42	910548	ovary	carcinoma	urogenital_system	ovary	0.832185	0.934893	0.092214	4.651011	6.428523	7.684124	9.24138	0.989631	0.0103	1101687	3.926105	6.428523	10.53231
OE33	910549	oesophagus	other	aero_digestive_tract	oesophagus	0.833221	0.777551	0.037456	1.894811	3.557762	5.124561	6.860256	0.920294	0.0026	991468	2.625926	3.557762	5.022675
CW-2	910554	large_intestine	carcinoma	digestive_system	large_intestine	0.842381	0.963075	0.145402	4.458421	6.145652	7.326703	8.839561	0.989909	0.0432	991356	2.814292	6.145652	10.793819
CMK	910566			blood	acute_myeloid_leukaemia	0.849739	1.050512	0.100033	5.291366	6.795331	7.846418	9.173393	0.996323	0.0046	1323232	4.023715	6.795331	11.031657
CHP-126	910567	autonomic_ganglia	neuroblastoma	nervous_system	neuroblastoma	0.840492	0.995231	0.080504	4.325861	5.982937	7.047375	8.483861	0.990189	0.0034	1325220	3.543114	5.982937	10.213056
CGTH-W-1	910568	thyroid	carcinoma	thyroid	thyroid	0.796408	0.667349	0.132565	2.086293	4.301632	6.210344	8.420523	0.925548	0.018	1035891	2.745944	4.301632	7.204712
COLO-320-HSR	910569	large_intestine	carcinoma	digestive_system	large_intestine	0.844368	1.095632	0.066333	5.839579	7.261281	8.252335	9.545976	0.997832	0.0044	1258396	4.413602	7.261281	11.480306
ATN-1	910687	haematopoietic_and_lymphoid_tissue	lymphoid_neoplasm	blood	lymphoblastic_T_cell_leukaemia	0.862464	1.029859	0.085339	3.674597	5.307627	6.235462	7.598235	0.98566	0.0057	1322252	3.212568	5.307627	9.563017
CESS	910688	haematopoietic_and_lymphoid_tissue	haematopoietic_neoplasm	blood	acute_myeloid_leukaemia	0.847967	1.047299	0.109391	5.249589	6.751612	7.794912	9.140617	0.996327	0.0076	1323736	3.809102	6.751612	11.108209
COLO-678	910689	large_intestine	carcinoma	digestive_system	large_intestine	0.810811	0.812415	0.062327	4.712936	6.83482	8.281639	10.082572	0.987106	0.0091	991381	4.147024	6.83482	11.300777
COLO-684	910691	endometrium	carcinoma	urogenital_system	endometrium	0.841514	0.928326	0.097243	4.667474	6.476855	7.665258	9.239869	0.990078	0.0046	1257388	3.865153	6.476855	11.023509
COLO-668	910692	lung	carcinoma	lung	lung_small_cell_carcinoma	0.924746	1.59021	0.105507	-1.598814	-0.901274	-0.145201	0.609654	0.461677	0.0097	1035892	-1.389615	-0.901274	-0.569573
LN-405	910694	central_nervous_system	glioma	nervous_system	glioma	0.862049	0.900854	0.151633	3.286318	5.110462	6.27263	7.843792	0.973736	0.0801	1049481	2.9828	5.110462	9.145709
TGBC24TKB	910695	biliary_tract	carcinoma	digestive_system	biliary_tract	0.816212	0.813002	0.109326	2.280044	4.036622	5.494163	7.230265	0.943743	0.0184	1000000	2.792759	4.036622	6.526317
A388	910697	skin	carcinoma	skin	skin_other	0.884771	0.795472	0.078128	0.900039	2.660707	3.756161	5.353267	0.859703	0.0105	1035885	1.853007	2.660707	5.565655
BFTC-909	910698	kidney	carcinoma	kidney	kidney	0.664597	0.296229	0.07972	-0.34389	4.352026	9.227399	14.804998	0.873933	0.0047	1000013	2.220342	4.352026	7.713989
C2BBe1	910700	large_intestine	carcinoma	digestive_system	large_intestine	0.852837	0.890409	0.075426	3.728945	5.466674	6.714441	8.272481	0.979327	0.003	991475	3.567551	5.466674	8.945631
SCC-25	910701	upper_aerodigestive_tract	carcinoma	aero_digestive_tract	head_and_neck	0.83638	0.957554	0.109294	3.851144	5.582059	6.696327	8.210523	0.98362	0.0064	1035861	3.405148	5.582059	9.646752
AsPC-1	910702	pancreas	carcinoma	pancreas	pancreas	0.844496	0.999858	0.109736	4.763044	6.349911	7.448555	8.863466	0.99278	0.0036	1049475	3.80113	6.349911	10.312852
CA46	910703	haematopoietic_and_lymphoid_tissue	lymphoid_neoplasm	blood	Burkitt_lymphoma	0.855381	1.081067	0.095784	5.614035	7.053207	8.074581	9.374975	0.997744	0.0169	1324436	4.235647	7.053207	11.389661
AU565	910704	breast	carcinoma	breast	breast	0.822188	0.914329	0.120215	3.860184	5.702053	6.853446	8.440124	0.983113	0.0061	1049477	3.256177	5.702053	10.399195
BALL-1	910705	haematopoietic_and_lymphoid_tissue	lymphoid_neoplasm	blood	B_cell_leukemia	0.847757	1.109776	0.08155	6.059479	7.466442	8.415954	9.678659	0.997815	0.0196	1262736	4.554988	7.466442	11.76278
BL-41	910706	haematopoietic_and_lymphoid_tissue	lymphoid_neoplasm	blood	Burkitt_lymphoma	0.859799	1.10626	0.073097	5.999041	7.411628	8.368075	9.62801	0.99801	0.0049	1259264	4.531011	7.411628	11.727861
BV-173	910710	haematopoietic_and_lymphoid_tissue	haematopoietic_neoplasm	blood	chronic_myeloid_leukaemia	0.863579	1.086157	0.1147	3.206163	4.880904	5.664913	6.95227	0.97832	0.0122	1256156	2.791211	4.880904	9.345326
IA-LM	910779	lung	carcinoma	lung	lung_NSCLC_large_cell	0.831852	0.768692	0.101006	3.24574	5.336246	6.830572	8.675482	0.962904	0.0094	1101597	3.330915	5.336246	9.071774
TGW	910780	autonomic_ganglia	neuroblastoma	nervous_system	neuroblastoma	0.863921	1.096833	0.05773	5.776567	7.187222	8.154745	9.413238	0.997921	0.0046	1257472	4.443019	7.187222	11.37089
AN3-CA	910781	endometrium	carcinoma	urogenital_system	endometrium	0.834055	0.968178	0.066967	3.916131	5.574062	6.721814	8.173387	0.986425	0.0039	991422	3.582806	5.574062	9.135339
A204	910784	soft_tissue	rhabdomyosarcoma	soft_tissue	rhabdomyosarcoma	0.674407	0.186237	0.107512	-4.625902	2.550418	10.35141	19.205418	0.867345	0.006	1035972	0.990093	2.550418	4.898071
NY	910849	bone	osteosarcoma	bone	osteosarcoma	0.65256	0.292792	0.094605	1.331554	6.432892	11.395547	17.369338	0.906213	0.0078	1049521	3.570918	6.432892	11.762463
C3A	910850	liver	carcinoma	digestive_system	liver	0.852221	0.904931	0.060645	3.947246	5.695689	6.887162	8.416749	0.98285	0.0043	991314	3.721247	5.695689	9.42646
A427	910851	lung	carcinoma	lung	lung_NSCLC_not_specified	0.831201	0.839467	0.047255	3.897168	5.708154	7.055373	8.711969	0.979194	0.0021	1101639	3.77809	5.708154	9.111525
CAL-85-1	910852	breast	carcinoma	breast	breast	0.840713	0.834229	0.087934	3.166136	4.995037	6.373456	8.02643	0.96417	0.0087	991348	3.254845	4.995037	8.108439
CHL-1	910853	skin	malignant_melanoma	skin	melanoma	0.816369	0.604313	0.101122	1.041645	3.470979	5.30495	7.638903	0.869379	0.0032	1035944	1.961433	3.470979	6.846501
RL	910861	haematopoietic_and_lymphoid_tissue	lymphoid_neoplasm	blood	B_cell_lymphoma	0.848976	1.053574	0.095693	5.158999	6.704051	7.700929	9.064025	0.996363	0.0058	1323036	3.958626	6.704051	11.20179
NCI-H1651	910900	lung	carcinoma	lung	lung_NSCLC_adenocarcinoma	0.83941	0.807246	0.126888	2.374937	4.221976	5.604792	7.323255	0.950816	0.0079	1049498	2.74828	4.221976	7.33666
RPMI-7951	910903	skin	malignant_melanoma	skin	melanoma	0.805547	0.537868	0.070561	2.367986	5.087163	7.432887	10.194197	0.928604	0.0079	991317	3.150421	5.087163	8.658265
SCC-4	910904	upper_aerodigestive_tract	carcinoma	aero_digestive_tract	head_and_neck	0.836077	1.024714	0.154148	4.760366	6.385291	7.394871	8.794418	0.993338	0.0409	999981	3.624975	6.385291	11.085735
SNU-C1	910905	large_intestine	carcinoma	digestive_system	large_intestine	0.842353	1.064936	0.115222	5.43211	6.896927	7.952577	9.287927	0.9965	0.0077	1323568	4.094696	6.896927	10.972499
ST486	910906	haematopoietic_and_lymphoid_tissue	lymphoid_neoplasm	blood	Burkitt_lymphoma	0.847576	1.077372	0.091081	5.394324	6.855339	7.86711	9.180651	0.996401	0.018	1263184	4.107364	6.855339	11.058074
SW1990	910907	pancreas	carcinoma	pancreas	pancreas	0.8031	0.623598	0.100185	2.486387	4.838624	6.819067	9.156419	0.935542	0.0083	991334	3.090418	4.838624	7.941374
SCC-15	910911	upper_aerodigestive_tract	carcinoma	aero_digestive_tract	head_and_neck	0.81911	0.889989	0.114085	4.035509	5.832138	7.131771	8.755119	0.983412	0.0119	1049335	3.057113	5.832138	10.109067
Capan-2	910915	pancreas	carcinoma	pancreas	pancreas	0.845857	1.06004	0.084819	5.381419	6.834847	7.857939	9.181339	0.996425	0.0064	1049368	4.218344	6.834847	10.741841
CAL-27	910916	upper_aerodigestive_tract	carcinoma	aero_digestive_tract	head_and_neck	0.854514	1.030421	0.076758	5.001108	6.557084	7.588628	8.960127	0.995126	0.0058	1049346	3.962947	6.557084	10.726599
BC-1	910919	haematopoietic_and_lymphoid_tissue	lymphoid_neoplasm	blood	B_cell_lymphoma	0.841464	0.998556	0.117484	4.959787	6.515689	7.635091	9.054134	0.993954	0.0186	1324464	3.514519	6.515689	10.808386
A704	910920	kidney	carcinoma	kidney	kidney	0.874546	0.956216	0.060487	2.068235	3.397966	4.599925	5.951895	0.937614	0.0045	1101586	2.733465	3.397966	4.658691
A101D	910921	skin	malignant_melanoma	skin	melanoma	0.650334	0.255498	0.121331	-2.379221	3.130976	8.969889	15.713964	0.870491	0.0067	1035882	1.035831	3.130976	6.339813
769-P	910922	kidney	carcinoma	kidney	kidney	0.906767	1.031051	0.120707	1.430341	2.734786	3.722387	4.969966	0.897566	0.0181	991386	2.048324	2.734786	4.766155
23132-87	910924	stomach	carcinoma	digestive_system	stomach	0.846615	0.891335	0.119307	3.544226	5.374645	6.628249	8.225809	0.975623	0.0118	1233310	3.041679	5.374645	9.416752

A431	910925	skin	carcinoma	skin	skin_other	0.882152	1.055744	0.084019	3.198577	4.771475	5.794633	7.119171	0.972329	0.0069	1035981	2.402137	4.771475	9.323321
BFTC-905	910926	urinary_tract	carcinoma	urogenital_system	Bladder	0.734993	0.348565	0.066713	0.518762	4.274425	8.071364	12.370934	0.872349	0.004	1036016	2.958415	4.274425	6.626094
CAL-51	910927	breast	carcinoma	breast	breast	0.847223	1.005125	0.092124	4.503223	6.140532	7.17211	8.580269	0.991413	0.0063	1035872	3.697291	6.140532	10.356098
RERF-LC-MS	910931	lung	carcinoma	lung	lung_NSCLC_adenocarcinoma	0.844541	0.926833	0.121381	3.667358	5.444305	6.621469	8.173698	0.981964	0.0031	1049328	3.241691	5.444305	9.65235
GAK	910932	skin	malignant_melanoma	skin	melanoma	0.875677	1.019414	0.143458	1.624295	2.928628	4.015986	5.306833	0.914182	0.0522	1035906	2.143116	2.928628	4.678408
AM-38	910933	central_nervous_system	glioma	nervous_system	glioma	0.824199	0.808435	0.154011	2.458643	4.424743	5.83409	7.614592	0.950233	0.0925	1035887	2.720316	4.424743	8.203426
A4-Fuk	910934	haematopoietic_and_lymphoid_tissue	lymphoid_neoplasm	blood	B_cell_lymphoma	0.83309	1.091872	0.109696	5.749479	7.167932	8.178208	9.465926	0.998022	0.0224	1308458	4.333398	7.167932	11.302668
A3-KAW	910935	stomach	carcinoma	blood	B_cell_lymphoma	0.844506	1.099143	0.093244	5.93404	7.343448	8.35433	9.628074	0.997897	0.0217	1309830	4.434318	7.343448	11.564626
DOK	910936	upper_aerodigestive_tract	other	aero_digestive_tract	head_and_neck	0.851747	0.939932	0.088928	4.539867	6.281301	7.458109	8.960489	0.989771	0.0054	991389	3.822785	6.281301	10.371259
COR-L279	910937			lung	lung_small_cell_carcinoma	0.843672	1.002015	0.080547	3.595282	5.307077	6.276128	7.686323	0.983378	0.0051	1324156	3.267319	5.307077	9.520069
NEC8	910942	testis	germ_cell_tumour	urogenital_system	testis	0.816545	0.745716	0.132918	2.395152	4.380461	5.999276	7.913505	0.944591	0.0178	1101685	2.824522	4.380461	7.195125
CAS-1	910943	central_nervous_system	glioma	nervous_system	glioma	0.800537	0.757907	0.128424	1.314799	3.111926	4.706641	6.590869	0.899351	0.0234	1049363	2.160964	3.111926	5.17825
ALL-PO	910944	haematopoietic_and_lymphoid_tissue	lymphoid_neoplasm	blood	lymphoblastic_leukemia	0.857088	0.908477	0.123644	2.123158	3.913047	5.138663	6.707126	0.935953	0.0076	1254084	1.90591	3.913047	7.539575
S-117	910946	soft_tissue	sarcoma	thyroid	thyroid	0.852366	0.886737	0.12582	2.943006	4.792594	5.988189	7.595431	0.96665	0.0307	1233316	2.93036	4.792594	8.817186
OCI-AML2	910947	haematopoietic_and_lymphoid_tissue	haematopoietic_neoplasm	blood	acute_myeloid_leukaemia	0.833113	1.003615	0.074449	3.390435	4.968454	6.006564	7.426828	0.979864	0.0021	1322868	3.286564	4.968454	8.434724
MFM-223	910948	breast	carcinoma	breast	breast	0.850931	1.07579	0.049508	2.458622	3.682729	4.770933	6.020894	0.957246	0.0068	991443	2.955809	3.682729	4.941552
CAL-54	910952	kidney	carcinoma	kidney	kidney	0.697225	0.269933	0.108139	-2.043407	2.986698	8.186827	14.161331	0.848404	0.0057	999988	1.400192	2.986698	5.717436
EFO-21	911905	ovary	carcinoma	urogenital_system	ovary	0.835499	1.080851	0.095181	5.720028	7.183919	8.195353	9.518154	0.99778	0.0223	1035957	4.221268	7.183919	11.521826
LS-1034	917486	large_intestine	carcinoma	digestive_system	large_intestine	0.825147	0.983527	0.136337	4.468086	6.147059	7.283805	8.767663	0.989912	0.0091	991364	3.57106	6.147059	10.444376
22RV1	924100	prostate	carcinoma	urogenital_system	prostate	0.815185	0.76328	0.102966	3.277473	5.346028	6.906362	8.839366	0.961034	0.0084	1049513	3.343921	5.346028	8.966881
8505C	924102	thyroid	carcinoma	thyroid	thyroid	0.82449	0.828363	0.089308	3.083951	4.965466	6.327657	8.057069	0.962246	0.0031	1101615	3.268323	4.965466	8.498781
BCPAP	924104	thyroid	carcinoma	thyroid	thyroid	0.878547	0.866284	0.10879	1.059577	2.470102	3.83873	5.333096	0.869635	0.0107	1036011	1.976002	2.470102	3.301405
BPH-1	924105	prostate	hyperplasia	urogenital_system	prostate	0.847878	0.868139	0.090352	3.221172	5.035729	6.296112	7.897903	0.968831	0.0058	991463	3.267135	5.035729	8.566899
CAL-39	924107	vulva	carcinoma	urogenital_system	cervix	0.833759	0.977286	0.114827	4.887579	6.598685	7.723029	9.215502	0.992966	0.0028	1049354	3.82423	6.598685	11.154993
CaR-1	924108	large_intestine	carcinoma	digestive_system	large_intestine	0.83776	1.034989	0.151827	5.276114	6.830718	7.842242	9.219394	0.996104	0.0447	1049369	4.001071	6.830718	11.4504
EFE-184	924109	endometrium	carcinoma	urogenital_system	endometrium	0.818457	0.847471	0.061627	4.184493	6.049184	7.379756	9.033831	0.983366	0.0064	1233334	3.908836	6.049184	9.812181
HDLM-2	924110	haematopoietic_and_lymphoid_tissue	lymphoid_neoplasm	blood	Hodgkin_lymphoma	0.853979	1.074317	0.06421	3.773144	5.404809	6.26191	7.586329	0.986733	0.0042	1257556	3.348583	5.404809	9.757209
HO-1-N-1	924111	upper_aerodigestive_tract	carcinoma	aero_digestive_tract	head_and_neck	0.839537	1.041375	0.056471	5.122604	6.736128	7.711549	9.089304	0.995776	0.0034	991409	4.144774	6.736128	11.250371
HTC-C3	924151	thyroid	carcinoma	thyroid	thyroid	0.851011	1.073642	0.062775	4.942876	6.414593	7.418281	8.721514	0.995167	0.0017	991414	3.937674	6.414593	10.53919
KGN	924186	ovary	other	urogenital_system	ovary	0.80881	0.682764	0.122064	1.886905	3.944684	5.724084	7.808865	0.921311	0.0205	999994	2.631249	3.944684	6.428046
KLE	924187	endometrium	carcinoma	urogenital_system	endometrium	0.852559	1.032724	0.11539	5.305324	6.837393	7.878848	9.250792	0.996105	0.0287	999995	4.128746	6.837393	10.950438
KNS-81-FD	924188	central_nervous_system	glioma	nervous_system	glioma	0.851373	0.940413	0.117453	3.806885	5.568763	6.679723	8.191443	0.982548	0.0061	1049387	3.352799	5.568763	9.727931
K5	924238	thyroid	carcinoma	thyroid	thyroid	0.792453	0.647768	0.150394	1.220744	3.389414	5.400228	7.618768	0.882182	0.0153	1035915	1.639403	3.389414	5.956468
L-363	924239	haematopoietic_and_lymphoid_tissue	lymphoid_neoplasm	blood	Myeloma	0.851293	1.102075	0.080499	5.901602	7.325669	8.291256	9.555998	0.997947	0.0104	1262512	4.493426	7.325669	11.584121
MDA-MB-415	924240	breast	carcinoma	breast	breast	0.842685	0.909652	0.127869	3.592741	5.446298	6.637158	8.209429	0.979042	0.0061	1101666	3.257476	5.446298	9.60347
NCI-H1975	924244	lung	carcinoma	lung	lung_NSCLC_adenocarcinoma	0.676777	0.358118	0.083822	1.758318	5.998383	10.531219	15.630008	0.912469	0.0163	1233352	0.616382	5.998383	11.333042
SW954	924247	vulva	carcinoma	urogenital_system	urogenital_system_other	0.852217	0.992297	0.110382	4.782984	6.416196	7.531719	8.965617	0.993386	0.0081	1049414	3.780939	6.416196	10.609666
MKN7	924250	stomach	carcinoma	digestive_system	stomach	0.858244	1.024116	0.120395	4.98382	6.550968	7.573384	8.952704	0.994173	0.0049	1049494	3.858753	6.550968	10.99496
MDA-MB-157	925338	breast	carcinoma	breast	breast	0.843396	0.942849	0.126189	4.039706	5.704218	6.921931	8.443206	0.987229	0.0411	991438	2.290663	5.704218	10.203376
MMAC-SF	925339	skin	malignant_melanoma	skin	melanoma	0.824243	0.727709	0.160449	1.717991	3.722993	5.336346	7.315012	0.915024	0.0064	1049391	2.324268	3.722993	6.947948
MKN45	925340	stomach	carcinoma	digestive_system	stomach	0.862448	0.991171	0.082758	4.369644	6.055075	7.105197	8.546907	0.989232	0.001	1049493	3.715541	6.055075	10.440098
NCI-H810	925341	lung	carcinoma	lung	lung_NSCLC_large_cell	0.867374	1.008186	0.162262	4.270997	5.436389	6.69916	8.004753	0.990307	0.1152	1049515	1.628976	5.436389	10.308147
NBsusSR	925342	autonomic_ganglia	neuroblastoma	nervous_system	neuroblastoma	0.839761	1.045558	0.079065	5.519148	7.077196	8.089317	9.457077	0.996657	0.0059	1309718	4.286161	7.077196	11.556697
MFH-ino	925343	soft_tissue	malignant_fibrous_histiocytoma-pleomorphic_sarcoma	soft_tissue	soft_tissue_other	0.826265	0.721671	0.152884	1.66428	3.635372	5.294553	7.242376	0.907687	0.037	1311796	2.331965	3.635372	6.358068
NOS-1	925345	bone	osteosarcoma	bone	osteosarcoma	0.846956	1.057888	0.082339	5.375547	6.898854	7.891983	9.226533	0.996385	0.0078	1035934	4.151774	6.898854	11.374083
PANC-03-27	925346	pancreas	carcinoma	pancreas	pancreas	0.865007	0.999383	0.094909	4.551596	6.154179	7.240171	8.646668	0.992337	0.0116	1049524	3.682597	6.154179	10.202959
PANC-08-13	925347	pancreas	carcinoma	pancreas	pancreas	0.834059	1.048937	0.092931	5.011287	6.555455	7.550022	8.891497	0.995415	0.0038	1049525	3.95397	6.555455	11.062249
PANC-10-05	925348	pancreas	carcinoma	pancreas	pancreas	0.845706	1.123703	0.057646	6.121807	7.506819	8.472862	9.732564	0.998995	0.0063	991472	4.691398	7.506819	11.627355
RO82-W-1	930083	thyroid	carcinoma	thyroid	thyroid	0.852767	0.921818	0.091141	3.169279	4.86971	6.052708	7.564872	0.970809	0.0056	991316	3.192917	4.86971	8.170011
SiHa	930297	cervix	carcinoma	urogenital_system	cervix	0.731543	0.422334	0.141169	1.024534	4.497349	7.814374	11.731563	0.884049	0.0209	1101556	2.648525	4.497349	7.939407
SKG-IIIa	930298	cervix	carcinoma	urogenital_system	cervix	0.786693	0.655926	0.107885	3.244313	5.782748	7.660952	9.942349	0.957434	0.0131	1101558	3.458712	5.782748	10.071911
TT	930299	thyroid	carcinoma	thyroid	thyroid	0.816994	0.760631	0.107289	3.420349	5.477207	7.111895	9.008745	0.965257	0.0226	1233322	2.892323	5.477207	9.306823
TE-9	946353	oesophagus	carcinoma	aero_digestive_tract	oesophagus	0.848981	0.882754	0.090317	2.89675	4.561891	5.860188	7.441431	0.960488	0.0031	1049420	3.118227	4.561891	7.245969
TE-11	946354	oesophagus	carcinoma	aero_digestive_tract	oesophagus	0.853245	0.962417	0.080226	3.992231	5.637405	6.744645	8.188441	0.986738	0.0043	1049417	3.580955	5.637405	9.432809
TE-6	946355	oesophagus	carcinoma	aero_digestive_tract	oesophagus	0.839994	0.846656	0.118246	2.165971	3.778515	5.169942	6.791595	0.940035	0.0029	1049418	2.671472	3.778515	5.880515
TE-12	946356	oesophagus	carcinoma	aero_digestive_tract	oesophagus	0.839575	1.044676	0.07408	4.544982	6.120679	7.1292	8.499569	0.992649	0.0038	1263492	3.701974	6.120679	10.190329
TC-YIK	946357	cervix	carcinoma	urogenital_system	cervix	0.858269	0.968543	0.130484	2.9537	4.594891	5.635799	7.070147	0.969111	0.0169	1323988	2.858235	4.594891	8.057459
YT	946358	haematopoietic_and_lymphoid_tissue	lymphoid_neoplasm	blood	lymphoid_neoplasm_other	0.846224	1.031886	0.080293	5.284338	6.80927	7.878836	9.258732	0.996657	0.009	1257836	4.107782	6.80927	11.136855
BT-474	946359	breast	carcinoma	breast	breast	0.844195	0.923594	0.108856	4.130967	5.936039	7.09058	8.633065	0.986839	0.0041	1049517	3.567675	5.936039	10.42586
OAW-28	946360	ovary	carcinoma	urogenital_system	ovary	0.821722	0.907599	0.075134	3.19431	4.845017	6.066456	7.615204	0.970453	0.0021	1049522	3.311682	4.845017	7.842671
LCLC-97TM1	946361	lung	carcinoma	lung	lung_NSCLC_large_cell	0.870743	0.994789	0.141688	4.659854	6.305875	7.320744	8.701154	0.992784	0.0237	1049478	3.669919	6.305875	10.894696
D-247MG	946367	central_nervous_system	glioma	nervous_system	glioma	0.726179	0.407771	0.118563	1.390748	4.946824	8.305884	12.209756	0.898076	0.0093	1035894	2.70976	4.946824	8.766987
D-263MG	946368	central_nervous_system	glioma	nervous_system	glioma	0.82229	0.81518	0.158612	1.315247	2.919634	4.412786	6.121505	0.891648	0.0404	1035895	1.99498	2.919634	4.530133
D-336MG	946369	central_nervous_system	glioma	nervous_system	glioma	0.846273	0.906682	0.064927	3.810303	5.640575	6.766862	8.301526	0.982916	0.0038	1035896	3.548354	5.640575	9.868803
D-392MG	946370	central_nervous_system	glioma	nervous_system	glioma	0.812971	0.801283	0.112833	2.982075	4.874898	6.340677	8.115092	0.960249	0.0075	1049366	3.098184	4.874898	7.984966
D-423MG	946372	central_nervous_system	glioma	nervous_system	glioma	0.850184	0.919452	0.113484	3.40325	5.120968	6.357111	7.886773	0.974398	0.0062	1049367	3.229194	5.120968	8.651492
D-502MG	946373	central_nervous_system	glioma	nervous_system	glioma	0.844285	0.87744	0.112622	1.837738	3.553954	4.941365	6.509902	0.922725	0.0175	1035897	1.635348	3.553954	7.275331
D-566MG	946377	central_nervous_system	glioma	nervous_system	glioma	0.831592	0.788291	0.071686	3.147401	5.034036	6.491201	8.285701	0.961119	0.0052	1101587	3.331182	5.034036	8.224401
CAMA-1	946382	breast	carcinoma	breast	breast	0.854762	1.042264	0.086755	4.403237	5.99843	6.964905	8.321285	0.992302	0.0371	1035881	3.480813	5.99843	10.424547
CTB-1	949088	haematopoietic_and_lymphoid_tissue	lymphoid_neoplasm	blood	B_cell_lymphoma	0.843998	1.100341	0.07356	6.264425	7.66065	8.637173	9.89596	0.999016	0.0151	1262848	4.761071	7.66065	11.864335
Caov-4	949090	ovary	carcinoma	urogenital_system	ovary	0.675753	0.283287	0.13327	-0.786905	4.335884	9.535544	15.688561	0.8725	0.0177	1233326	2.205819	4.335884	8.223506
M059J	949094	central_nervous_system	glioma	nervous_system	glioma	0.855613	0.933303	0.080351	3.999218	5.723661	6.914961	8.453201	0.985779	0.0063	991437	3.550729	5.723661	9.515958
HAL-01	949153	haematopoietic_and_lymphoid_tissue	lymphoid_neoplasm	blood	lymphoblastic_leukemia	0.838153	1.111157	0.065778	5.706022	7.139582	8.059219	9.315513	0.99795	0.0124	1258004	4.300703	7.139582	11.438085
OMC-1	949154	cervix	carcinoma	urogenital_system	cervix	0.768467	0.55953	0.103155	2.698138	5.559443	7.903488	10.690353	0.937646	0.0277	1101602	3.300534	5.559443	9.70848
ES8	949155	bone	Ewings_sarcoma-peripheral_primitive_neuroectodermal_tumour	bone	ewings_sarcoma	0.810179	0.727999	0.124743	1.578022	3.468141	5.25167	7.232739	0.90231	0.0223	1101593	1.542178	3.468141	6.120318
ES4	949156	bone	Ewings_sarcoma-peripheral_primitive_neuroectodermal_tumour	bone	ewings_sarcoma	0.829456	0.863675	0.140239	3.235924	5.161171	6.399429	8.068286	0.969291	0.0203	1049371	3.018997	5.161171	9.685502
ES6	949157	bone	Ewings_sarcoma-peripheral_primitive_neuroectodermal_tumour	bone	ewings_sarcoma	0.846669	0.805256	0.082432	3.027774	4.833671	6.236514	7.929277	0.959695	0.0023	1101591	3.272731	4.833671	7.732448
ES1	949158	bone	Ewings_sarcoma-peripheral_primitive_neuroectodermal_tumour	bone	ewings_sarcoma	0.787849	0.570875	0.140364	1.60012	4.08552	6.334186	8.96418	0.897251	0.0076	1101590	2.567605	4.08552	6.901712
EW-7	949160	bone	Ewings_sarcoma-peripheral_primitive_neuroectodermal_tumour	bone	ewings_sarcoma	0.86237	1.037464	0.097116	5.135625	6.671002	7.656431	8.988304	0.99612	0.0098	1264556	4.02349	6.671002	11.042839
EW-3	949161	bone	Ewings_sarcoma-peripheral_primitive_neuroectodermal_tumour	bone	ewings_sarcoma	0.843374	1.017553	0.12835	5.200002	6.791512	7.860028	9.259306	0.996029	0.0098	1257080	3.818802	6.791512	11.470598
EW-1	949163	bone	Ewings_sarcoma-peripheral_primitive_neuroectodermal_tumour	bone	ewings_sarcoma	0.790507	0.709018	0.12373	2.628191	4.781959	6.533424	8.636415	0.944564	0.0197	1101594	2.422806	4.781959	8.481723
EW-18	949164	bone	Ewings_sarcoma-peripheral_primitive_neuroectodermal_tumour	bone	ewings_sarcoma	0.845193	1.078739	0.111885	5.493291	6.908267	7.937999	9.233526	0.996661	0.0307	1264444	4.113306	6.908267	11.138701
EW-16	949165	bone	Ewings_sarcoma-peripheral_primitive_neuroectodermal_tumour	bone	ewings_sarcoma	0.858426	1.067402	0.068072	5.5892	7.09192	8.041689	9.345133	0.997681	0.0028	1049377	4.304931	7.09192	11.42222
EW-13	949166	bone	Ewings_sarcoma-peripheral_primitive_neuroectodermal_tumour	bone	ewings_sarcoma	0.855054	1.053615	0.079996	5.702643	7.189293	8.236713	9.571825	0.997669	0.0049	1321944	4.298701	7.189293	11.528749
EW-22	949167	bone	Ewings_sarcoma-peripheral_primitive_neuroectodermal_tumour	bone	ewings_sarcoma	0.691774	0.457881	0.127908	-1.792617	1.17086	4.157479	7.671275	0.755507	0.0112	1035905	0.084308	1.17086	2.923417
EW-24	949168	bone	Ewings_sarcoma-peripheral_primitive_neuroectodermal_tumour	bone	ewings_sarcoma	0.8531	1.032986	0.109844	5.022256	6.587109	7.597191	8.950089	0.995271	0.0176	1323428	3.868422	6.587109	10.961383
LAN-6	949170	autonomic_ganglia	neuroblastoma	nervous_system	neuroblastoma	0.822129	0.803107	0.093024	0.249928	1.787861	3.353029	5.052913	0.806959	0.026	1035919	1.339936	1.787861	2.477541
NB10	949171	autonomic_ganglia	neuroblastoma	nervous_system	neuroblastoma	0.770694	0.577079	0.149042	1.241721	3.792904	6.065202	8.689054	0.885358	0.0177	1101601	1.748981	3.792904	7.094994
NB12	949172	autonomic_ganglia	neuroblastoma	nervous_system	neuroblastoma	0.621833	0.554198	0.056645	-2.649279	-0.214641	2.669696	5.845802	0.690068	0.0034	1035930	-0.657632	-0.214641	0.265405
NB6	949173	autonomic_ganglia	neuroblastoma	nervous_system	neuroblastoma	0.834468	0.878915	0.149126	2.907785	4.814359	6.053019	7.745477	0.956591	0.0668	1035933	2.130968	4.814359	9.633753
NB7	949174	autonomic_ganglia	neuroblastoma	nervous_system	neuroblastoma	0.784034	0.559311	0.107678	1.592698	4.040306	6.345838	8.969365	0.895355	0.0095	1049396	2.502698	4.040306	6.565291
NB17	949175	autonomic_ganglia	neuroblastoma	nervous_system	neuroblastoma	0.81883	0.760528	0.150705	2.658079	4.840013	6.434132	8.344948	0.955283	0.0927	1035932	2.879755	4.840013	8.764124
NB5	949176	autonomic_ganglia	neuroblastoma	nervous_system	neuroblastoma	0.7509	0.452253	0.169995	-0.180457	2.85668	5.870306	9.331349	0.842907	0.0126	1049395	1.566531	2.85668	4.981427

NB13	949177	autonomic_ganglia	neuroblastoma	nervous_system	neuroblastoma	0.844345	0.984099	0.143642	2.803426	4.451463	5.496396	6.950029	0.964395	0.022	1049394	2.783337	4.451463	7.897951
NB14	949178	autonomic_ganglia	neuroblastoma	nervous_system	neuroblastoma	0.8591	0.853555	0.093086	0.862113	2.51905	3.786315	5.384218	0.856776	0.0419	1035931	1.687274	2.51905	5.10983
NB1	949179	autonomic_ganglia	neuroblastoma	nervous_system	neuroblastoma	0.796688	0.58501	0.07992	1.737014	4.017678	6.162492	8.66161	0.905694	0.0054	1311712	2.795516	4.017678	6.320867
RH-1	971773	soft_tissue	rhabdomyosarcoma	soft_tissue	rhabdomyosarcoma	0.854025	0.869929	0.155295	2.020863	3.685221	4.964969	6.553918	0.942567	0.004	1049329	2.36787	3.685221	6.305426
RH-18	971774	soft_tissue	rhabdomyosarcoma	soft_tissue	rhabdomyosarcoma	0.833189	0.857289	0.138188	3.244339	5.098505	6.471846	8.161137	0.968635	0.0108	1049330	2.477939	5.098505	9.026635
MZ2-MEL	971777	skin	malignant_melanoma	skin	melanoma	0.862428	0.875343	0.104703	1.808371	3.325639	4.662624	6.192998	0.932245	0.0093	1049392	2.447853	3.325639	4.988396
