# Supplementary material for: LIM kinase inhibitors disrupt mitotic microtubule organization and impair tumor cell proliferation
Source: Oncotarget. 2015 Nov 3;6(36):38469–86. doi: 10.18632/oncotarget.6288 (PMC4770715; doi:10.18632/oncotarget.6288)
Supplement: Supplementary file 9 [file oncotarget-06-38469-s009.pdf]

Cell Line	Cosmic_ID	Primary_Site	Histology	GDSC_Desc1	GDSC_Desc2	CRT0105950_ALPHA	CRT0105950_BETA	CRT0105950_B	CRT0105950_IC_25	CRT0105950_IC_50	CRT0105950_IC_75	CRT0105950_IC_90	CRT0105950_AUC	CRT0105950_D	CRT0105950_IC_RESULTS_ID	CRT0105950_IC_50_LOW	CRT0105950_IC_50	CRT0105950_IC_50_HIGH
MC-CAR	683665	haematopoietic_and_lymphoid_tissue	lymphoid_neoplasm	blood	haematopoietic_neoplasm_other	0.863617	1.002489	0.098128	2.214633	3.606791	4.660869	5.97234	0.948514	0.0029	1258283	2.633892	3.606791	5.868883
PFSK-1	683667	central_nervous_system	primitive_neuroectodermal_tumour-medulloblastoma	nervous_system	medulloblastoma	0.853371	0.889163	0.162782	1.876232	3.596436	4.814113	6.375171	0.931903	0.0411	1049325	2.270522	3.596436	6.828317
A673	684052	soft_tissue	rhabdomyosarcoma	soft_tissue	rhabdomyosarcoma	0.887337	1.086492	0.073819	-0.266091	0.808965	1.922941	3.075358	0.737175	0.001	1049257	0.614677	0.808965	1.010699
ES3	684055	bone	Ewings_sarcoma-peripheral_primitive_neuroectodermal_tumour	bone	ewings_sarcoma	0.777153	0.648493	0.14497	-0.723126	1.199067	3.21886	5.425615	0.755051	0.0078	1049166	0.556093	1.199067	1.907048
ES5	684057	bone	Ewings_sarcoma-peripheral_primitive_neuroectodermal_tumour	bone	ewings_sarcoma	0.841345	0.833415	0.092221	-0.096407	1.337329	2.830611	4.420277	0.774567	0.0043	1049168	1.028847	1.337329	1.668033
ES7	684059	bone	Ewings_sarcoma-peripheral_primitive_neuroectodermal_tumour	bone	ewings_sarcoma	0.96285	0.911639	0.141819	-0.061088	1.216863	2.474944	3.790677	0.767263	0.0102	1049170	0.774233	1.216863	1.698372
EW-11	684062	bone	Ewings_sarcoma-peripheral_primitive_neuroectodermal_tumour	bone	ewings_sarcoma	0.826081	0.763787	0.110374	-0.03717	1.555996	3.205268	4.961414	0.791581	0.0049	1049172	1.158791	1.555996	2.023012
NCI-H1395	684681	lung	carcinoma	lung	lung_NSCLC_adenocarcinoma	0.688854	0.269641	0.168999	-3.627516	1.337195	6.765118	12.89068	0.817994	0.0075	1035822	-0.195538	1.337195	3.263065
COLO-829	687448	skin	malignant_melanoma	skin	melanoma	0.833064	0.771297	0.127327	0.820171	2.444711	4.040235	5.785029	0.852518	0.0092	1049160	1.856072	2.444711	3.410221
5637	687452	urinary_tract	carcinoma	urogenital_system	Bladder	0.991831	0.974283	0.056256	0.754688	1.927254	3.073326	4.256733	0.84568	0.0031	999931	1.745426	1.927254	2.229275
RT4	687455	urinary_tract	carcinoma	urogenital_system	Bladder	0.849441	1.046623	0.095422	4.5511	6.138004	7.102484	8.440258	0.992375	0.0124	1233259	3.601492	6.138004	10.443245
SW780	687457	urinary_tract	carcinoma	urogenital_system	Bladder	0.963943	1.344039	0.09714	0.977142	1.834662	2.680578	3.557587	0.862897	0.0089	991164	1.598968	1.834662	2.121385
TCCSUP	687459	urinary_tract	carcinoma	urogenital_system	Bladder	0.784895	0.555271	0.06791	1.844388	4.255129	6.518917	9.089024	0.918925	0.0046	999941	3.008719	4.255129	6.506321
C-33-A	687505	cervix	carcinoma	urogenital_system	cervix	0.973229	1.171612	0.086834	1.155795	2.207337	3.025426	4.021471	0.8742	0.0015	1101341	1.823727	2.207337	3.58663
ME-180	687514	cervix	carcinoma	urogenital_system	cervix	0.905586	1.029466	0.071585	1.341883	2.497918	3.637718	4.856459	0.898004	0.001	1233295	2.194291	2.497918	2.99313
8-MG-BA	687562	central_nervous_system	glioma	nervous_system	glioma	0.862021	0.734208	0.087814	0.316239	1.957259	3.623417	5.410008	0.82262	0.0046	1035685	1.578121	1.957259	2.48961
A172	687563	central_nervous_system	glioma	nervous_system	glioma	0.798852	0.594449	0.149047	1.310644	3.638134	5.751091	8.212612	0.888383	0.0114	1049233	2.382544	3.638134	6.11827
GB-1	687568	central_nervous_system	glioma	nervous_system	glioma	0.871293	0.902194	0.165884	0.901245	2.326423	3.589609	5.036096	0.872319	0.0177	1035712	1.591007	2.326423	3.806693
T98G	687586	central_nervous_system	glioma	nervous_system	glioma	0.884138	0.864742	0.113254	1.328758	2.809319	4.12946	5.652377	0.891467	0.0047	991169	2.155557	2.809319	4.248511
U-118-MG	687588	central_nervous_system	glioma	nervous_system	glioma	0.803403	0.615579	0.16396	-2.122699	-0.146311	1.956498	4.179983	0.664366	0.0045	1035678	-0.805862	-0.146311	0.495178
U-87-MG	687590	central_nervous_system	glioma	nervous_system	glioma	0.725106	0.451093	0.117364	-1.186259	1.685242	4.741388	8.155011	0.790195	0.0027	991171	0.944129	1.685242	2.679013
YKG-1	687592	central_nervous_system	glioma	nervous_system	glioma	0.883575	1.114897	0.10267	2.656446	4.524704	5.138063	6.423023	0.969541	0.0112	1049156	2.439813	4.524704	9.626805
ChaGo-K-1	687596	lung	carcinoma	lung	lung_NSCLC_not_specified	0.84557	0.769497	0.070095	1.64661	3.281048	4.866532	6.59926	0.907829	0.002	991199	2.612015	3.281048	4.405482
NCI-H720	687600	lung	carcinoid-endocrine_tumour	lung	lung_NSCLC_carcinoid	0.847253	1.07573	0.07513	5.27001	6.698693	7.76636	9.080516	0.996486	0.0066	1261055	3.830715	6.698693	10.688936
Calu-3	687777	lung	carcinoma	lung	lung_NSCLC_adenocarcinoma	0.852204	0.952554	0.116622	2.149857	3.590832	4.792952	6.21525	0.93759	0.0108	999945	2.606221	3.590832	5.52871
COR-L23	687780	lung	carcinoma	lung	lung_NSCLC_large_cell	0.91135	1.123337	0.09528	1.610036	2.855341	3.698935	4.840144	0.916942	0.0075	1049219	2.179164	2.855341	5.270524
LK-2	687787	lung	carcinoma	lung	lung_NSCLC_squamous_cell_carcinoma	0.929753	1.276785	0.07623	1.641091	2.87073	3.341452	4.310535	0.919736	0.0037	1049276	2.123809	2.87073	5.347832
NCI-H1437	687794	lung	carcinoma	lung	lung_NSCLC_adenocarcinoma	0.985018	1.120723	0.093271	0.550042	1.56673	2.584229	3.619826	0.815672	0.006	991279	1.341584	1.56673	1.795341
NCI-H1623	687798	lung	carcinoma	lung	lung_NSCLC_adenocarcinoma	0.896141	1.063024	0.132585	0.886122	2.024752	3.120517	4.314124	0.868875	0.0082	1049293	1.585634	2.024752	2.702983
NCI-H1648	687799	lung	carcinoma	lung	lung_NSCLC_adenocarcinoma	0.794673	0.656529	0.100765	0.715748	2.693335	4.605545	6.759335	0.851464	0.0109	1035824	1.799175	2.693335	4.139725
NCI-H1650	687800	lung	carcinoma	lung	lung_NSCLC_adenocarcinoma	0.837933	0.842966	0.060556	3.015114	4.711381	6.063971	7.678283	0.963546	0.0018	991281	3.302896	4.711381	7.401971
NCI-H1693	687802	lung	carcinoma	lung	lung_NSCLC_adenocarcinoma	0.815076	0.795609	0.111467	1.350016	2.959278	4.509645	6.250632	0.895862	0.0146	999969	2.227628	2.959278	4.177544
NCI-H1770	687804	lung	carcinoma	lung	lung_NSCLC_not_specified	1.083782	1.849539	0.085061	1.181132	1.779	2.35407	2.934371	0.882456	0.0061	1321971	1.601992	1.779	2.041732
NCI-H1838	687807	lung	carcinoma	lung	lung_NSCLC_adenocarcinoma	0.855455	0.93213	0.156499	2.372181	3.991931	5.155438	6.646326	0.947458	0.0235	991283	2.552504	3.991931	6.973494
NCI-H2126	687814	lung	carcinoma	lung	lung_NSCLC_adenocarcinoma	0.894313	1.007745	0.134685	0.902697	2.099873	3.267962	4.531978	0.861691	0.0111	1049194	1.645017	2.099873	2.808906
NCI-H2170	687815	lung	carcinoma	lung	lung_NSCLC_squamous_cell_carcinoma	0.865356	0.906786	0.071072	2.682128	4.187447	5.444468	6.917238	0.956353	0.0011	991287	3.095706	4.187447	6.25316
NCI-H2228	687816	lung	carcinoma	lung	lung_NSCLC_adenocarcinoma	0.852844	0.880689	0.099123	0.833345	2.207621	3.599179	5.097285	0.85373	0.0052	991288	1.782953	2.207621	2.807342
NCI-H2347	687820	lung	carcinoma	lung	lung_NSCLC_adenocarcinoma	0.910804	1.215787	0.126681	1.635706	3.124518	3.566087	4.646091	0.924652	0.0582	1049303	2.042044	3.124518	7.716382
NCI-H2405	687821	lung	carcinoma	lung	lung_NSCLC_adenocarcinoma	0.818555	0.760533	0.085954	1.634465	3.320764	4.964118	6.78778	0.910917	0.005	1049304	2.537615	3.320764	4.681456
NCI-H661	687829	lung	carcinoma	lung	lung_NSCLC_large_cell	0.831144	0.698343	0.141213	1.157366	3.051804	4.794541	6.774753	0.879653	0.0059	1049308	2.120678	3.051804	4.889823
DMS-114	687983	lung	carcinoma	lung	lung_small_cell_carcinoma	0.793244	0.656256	0.113862	0.172584	2.088299	4.044554	6.190344	0.816704	0.0022	1101408	1.514761	2.088299	2.907204
DMS-273	687985	lung	carcinoma	lung	lung_small_cell_carcinoma	0.888302	1.036846	0.105495	1.69979	3.038846	4.013079	5.276105	0.915591	0.013	991215	2.26829	3.038846	5.592821
NCI-H1048	687995	lung	carcinoma	lung	lung_small_cell_carcinoma	0.875043	1.025585	0.098871	1.333541	2.529444	3.680391	4.942126	0.902126	0.003	1049291	2.088924	2.529444	3.2647
NCI-H1092	687997	lung	carcinoma	lung	lung_small_cell_carcinoma	0.866088	1.046547	0.079371	2.770084	4.194501	5.174396	6.456386	0.969892	0.0067	1323007	2.945138	4.194501	6.792768
NCI-H1694	688006	lung	carcinoma	lung	lung_small_cell_carcinoma	0.919105	0.918864	0.073564	0.52589	1.793711	3.079437	4.411703	0.835382	0.0031	1265311	1.545508	1.793711	2.087859
NCI-H187	688007	lung	carcinoma	lung	lung_small_cell_carcinoma	1.025468	1.441803	0.094381	1.134574	2.045243	2.491898	3.228392	0.876343	0.0021	1307085	1.568765	2.045243	3.543495
NCI-H1963	688010	lung	carcinoma	lung	lung_small_cell_carcinoma	1.161707	1.831984	0.088332	0.190487	0.784073	1.362936	1.929928	0.765287	0.0032	1308709	0.599677	0.784073	0.973007
NCI-H2029	688011	lung	carcinoma	lung	lung_small_cell_carcinoma	0.72595	0.486672	0.136716	1.669279	4.874937	7.707261	11.079532	0.902542	0.0189	1035831	2.444446	4.874937	8.965529
NCI-H2141	688014	lung	carcinoma	lung	lung_small_cell_carcinoma	0.871844	1.143634	0.087493	3.684969	5.268438	6.021311	7.258274	0.98622	0.0199	1266403	2.744136	5.268438	9.999363
NCI-H2227	688018	lung	carcinoma	lung	lung_small_cell_carcinoma	0.843503	1.086498	0.106535	5.855999	7.249339	8.273287	9.560265	0.997955	0.0297	1306861	4.315051	7.249339	11.408952
NCI-H345	688021	lung	carcinoma	lung	lung_small_cell_carcinoma	0.841861	1.091568	0.093942	5.903894	7.335382	8.340143	9.619034	0.997933	0.0164	1266515	4.430243	7.335382	11.654355
NCI-H446	688023	lung	carcinoma	lung	lung_small_cell_carcinoma	0.858367	0.951398	0.093866	2.858173	4.495861	5.577212	7.026108	0.96974	0.0022	1307981	2.929581	4.495861	7.985351
NCI-H526	688025	lung	carcinoma	lung	lung_small_cell_carcinoma	1.442207	2.140537	0.081924	0.714371	1.199969	1.649503	2.05782	0.800383	0.0082	1263379	1.067175	1.199969	1.328551
NCI-H69	688027	lung	carcinoma	lung	lung_small_cell_carcinoma	0.844369	0.99637	0.074804	4.266925	5.905453	6.92327	8.331371	0.989141	0.0014	1267075	3.63664	5.905453	10.059593
NCI-H82	688031	lung	carcinoma	lung	lung_small_cell_carcinoma	0.912865	0.980671	0.079233	0.969555	2.179648	3.361523	4.625738	0.87686	0.0019	1262399	1.862076	2.179648	2.713354
NCI-H2052	688058	pleura	mesothelioma	lung	mesothelioma	0.890516	0.978256	0.124016	1.476576	2.768214	3.932622	5.243939	0.913348	0.0052	1049232	2.106104	2.768214	4.066873
SK-N-DZ	688086	autonomic_ganglia	neuroblastoma	nervous_system	neuroblastoma	0.881488	0.812348	0.094154	0.94902	2.655925	4.22987	5.973536	0.866003	0.0038	991156	1.608881	2.655925	4.575619
SK-N-FI	688087	autonomic_ganglia	neuroblastoma	nervous_system	neuroblastoma	0.847648	0.793304	0.146302	1.188517	2.861114	4.359666	6.089423	0.887574	0.0098	1049141	1.953392	2.861114	4.615335
VA-ES-BJ	688121	soft_tissue	epithelioid_sarcoma	soft_tissue	soft_tissue_other	0.89579	1.010045	0.078921	0.856723	2.025882	3.208801	4.455297	0.857543	0.0028	1101400	1.781821	2.025882	2.33704
SBC-5	713880	lung	carcinoma	lung	lung_small_cell_carcinoma	0.786554	0.535426	0.123878	1.172982	3.731777	6.08051	8.816065	0.881752	0.0163	1049130	2.452968	3.731777	6.227692
SBC-1	713885	lung	carcinoma	lung	lung_small_cell_carcinoma	0.848218	0.823251	0.084171	1.930147	3.500322	4.954911	6.586404	0.926855	0.003	1255875	2.691895	3.500322	4.925893
LU-135	713899	lung	carcinoma	lung	lung_small_cell_carcinoma	1.006444	1.441454	0.069509	1.128481	1.987929	2.613419	3.394562	0.886115	0.0031	1261279	1.573672	1.987929	2.871661
NCI-H2030	722045	lung	carcinoma	lung	lung_NSCLC_adenocarcinoma	0.892347	1.109697	0.113416	1.647713	2.790719	3.795461	4.953113	0.918574	0.0064	1049299	2.211426	2.790719	4.059241
NCI-H2122	722046	lung	carcinoma	lung	lung_NSCLC_adenocarcinoma	0.882517	0.856233	0.066749	1.61997	3.335588	4.269542	5.797914	0.908384	0.0028	991286	2.281611	3.335588	6.164389
NCI-H1734	722058	lung	carcinoma	lung	lung_NSCLC_adenocarcinoma	0.864669	0.962118	0.08322	2.4087	3.892198	5.038215	6.436784	0.95176	0.008	1312355	2.815146	3.892198	6.080968
NCI-H650	722066	lung	carcinoma	lung	lung_NSCLC_adenocarcinoma	0.885633	1.045957	0.127423	1.48406	2.67546	3.780427	5.016067	0.903718	0.03	1049307	2.09602	2.67546	3.718646
T-24	724812	urinary_tract	carcinoma	urogenital_system	Bladder	0.877614	0.952487	0.137455	1.383911	2.741106	3.922547	5.301823	0.89961	0.0104	1049147	2.046303	2.741106	4.2548
SK-N-AS	724828	autonomic_ganglia	neuroblastoma	nervous_system	neuroblastoma	0.926219	0.863867	0.091294	0.512854	1.867467	3.223614	4.645056	0.827395	0.0046	991155	1.551382	1.867467	2.297704
NCI-H1299	724831	lung	carcinoma	lung	lung_NSCLC_large_cell	0.881087	0.86434	0.073733	1.818427	3.27536	4.62623	6.13109	0.921747	0.0013	1049292	2.628512	3.27536	4.497382
NCI-H2087	724834	lung	carcinoma	lung	lung_NSCLC_adenocarcinoma	0.906433	0.982186	0.070053	0.440851	1.623685	2.840289	4.100459	0.809896	0.0015	991285	1.433389	1.623685	1.822546
UM-UC-3	724838	urinary_tract	carcinoma	urogenital_system	Bladder	0.948222	0.967183	0.092236	0.730129	1.945295	3.110386	4.351271	0.840526	0.0045	991174	1.63956	1.945295	2.509163
SW756	724839	cervix	carcinoma	urogenital_system	cervix	0.849237	0.861681	0.105774	1.76872	3.275199	4.645802	6.198735	0.92257	0.0018	1101369	2.476488	3.275199	4.767588
NCI-H727	724855	lung	carcinoid-endocrine_tumour	lung	lung_NSCLC_carcinoid	0.809618	0.778991	0.077497	0.548933	2.11316	3.735093	5.477275	0.833162	0.002	991294	1.800838	2.11316	2.533438
Calu-6	724859	lung	carcinoma	lung	lung_NSCLC_adenocarcinoma	0.764571	0.591455	0.103119	-0.263342	1.932887	4.319238	6.846954	0.805209	0.0169	1049158	0.561118	1.932887	2.864286
LU-65	724863	lung	carcinoma	lung	lung_NSCLC_large_cell	0.826831	1.08912	0.068491	5.675953	7.137536	8.114276	9.41474	0.997882	0.0048	1266851	4.336647	7.137536	11.622545
NCI-H1355	724866	lung	carcinoma	lung	lung_NSCLC_adenocarcinoma	0.765772	0.630917	0.09436	0.383034	2.366215	4.45673	6.722469	0.833024	0.0021	1049193	1.863508	2.366215	3.019258
NCI-H1792	724868	lung	carcinoma	lung	lung_NSCLC_adenocarcinoma	0.835481	0.895847	0.115724	2.075972	3.590852	4.881616	6.408597	0.938161	0.0054	1049296	2.614765	3.590852	5.540166
HPAF-II	724869	pancreas	carcinoma	pancreas	pancreas	0.813105	0.561761	0.10108	0.003976	2.292192	4.434111	6.916742	0.82442	0.0129	999959	1.149467	2.292192	4.188688
MIA-PaCa-2	724870	pancreas	carcinoma	pancreas	pancreas	0.85053	0.924282	0.052036	0.411562	1.690099	3.027284	4.435642	0.809216	0.0015	991273	1.535101	1.690099	1.856041
SHP-77	724872	lung	carcinoma	lung	lung_small_cell_carcinoma	0.875914	1.027287	0.10685	1.592953	2.823358	3.945079	5.205296	0.908016	0.0122	1049134	2.266624	2.823358	3.969718
NCI-H2009	724873	lung	carcinoma	lung	lung_NSCLC_adenocarcinoma	0.889712	0.776169	0.089191	0.377836	1.949384	3.390732	5.011465	0.817677	0.0057	1233299	1.389076	1.949384	3.066419
NCI-H2291	724874	lung	carcinoma	lung	lung_NSCLC_adenocarcinoma	0.822273	0.788194	0.100221	2.243677	4.020781	5.501941	7.269292	0.93848	0.0035	1049301	2.849257	4.020781	6.416257
SW1573	724878	lung	carcinoma	lung	lung_NSCLC_adenocarcinoma	0.929518	0.634474	0.06019	-0.381901	1.432157	3.283288	5.191266	0.769714	0.0012	1101367	1.196657	1.432157	1.681429
SW900	724879	lung	carcinoma	lung	lung_NSCLC_squamous_cell_carcinoma	0.805305	0.56138	0.037215	2.184931	4.434158	6.661312	9.117103	0.924865	0.001	991165	3.453855	4.434158	5.93843
TE-5	735784	oesophagus	carcinoma	aero_digestive_tract	oesophagus	0.85362	1.018432	0.066025	4.103546	5.741127	6.720547	8.08229	0.988328	0.0028	1035753	3.552399	5.741127	9.810551
HCC1954	749709	breast	carcinoma	breast	breast	0.824506	0.855994	0.104086	3.733121	5.608018	6.937797	8.616472	0.976834	0.0016	1049245	3.525811	5.608018	9.299187
HCC1143	749710	breast	carcinoma	breast	breast	0.8747	0.969039	0.095326	2.012756	3.343606	4.526111	5.873841	0.934919	0.0046	1233304	2.586574	3.343606	4.755316
HCC1187	749711	breast	carcinoma	breast	breast	0.89846	0.886106	0.176985	0.836269	2.425631	3.519185	5.003329	0.856904	0.0254	1049241	1.2345	2.425631	5.290544
HCC1395	749712	breast	carcinoma	breast	breast	0.835584	0.743743	0.089081	1.999596	3.819464	5.381225	7.217523	0.927438	0.0021	1101422	2.739919	3.819464	6.006451
HCC1937	749714	breast	carcinoma	breast	breast	0.852728	0.950372	0.082881	3.704506	5.398109	6.526449	8.001025	0.982492	0.0059	1049244	3.47626	5.398109	9.016517
HCC2157	749715	breast	carcinoma	breast	breast	1.056508	1.514808	0.092023	0.895069	1.636016	2.347207	3.075822	0.867578	0.01	1261167	1.394245	1.636016	1.992257
HCC2218	749716	breast	carcinoma	breast	breast	0.835456	1.028434	0.105518	5.063171	6.595256	7.663369	9.048375	0.995295	0.0044	1307421	3.941529	6.595256	10.67197

HCC38	749717	breast	carcinoma	breast	breast	0.861531	0.94725	0.112916	3.908968	5.643092	6.795269	8.270063	0.984758	0.0042	1049246	3.32448	5.643092	9.702489
BB30-HNC	753531	upper_aerodigestive_tract	carcinoma	aero_digestive_tract	head_and_neck	0.892224	0.877135	0.08619	0.67121	2.147074	3.65546	5.20403	0.844544	0.0028	1035693	1.231172	2.147074	3.491524
BB49-HNC	753532	upper_aerodigestive_tract	carcinoma	aero_digestive_tract	head_and_neck	0.780797	0.611192	0.117049	-1.250359	0.759331	2.899891	5.198958	0.718954	0.0026	991182	0.281033	0.759331	1.236559
BB65-RCC	753533	kidney	carcinoma	kidney	kidney	0.794086	0.615456	0.163406	0.851529	3.04743	5.089958	7.456988	0.875369	0.034	1035694	1.772921	3.04743	5.243374
BEN	753534	lung	carcinoma	lung	lung_NSCLC_not_specified	0.835438	0.825627	0.062243	2.632241	4.314966	5.707053	7.369953	0.956304	0.0021	991276	3.109954	4.314966	6.59882
BHY	753535	upper_aerodigestive_tract	carcinoma	aero_digestive_tract	head_and_neck	0.866045	0.851228	0.070797	1.533002	2.968605	4.379197	5.915043	0.904052	0.0069	991278	2.449497	2.968605	3.866305
BOKU	753536	cervix	carcinoma	urogenital_system	cervix	0.857457	0.900078	0.076195	2.975275	4.624005	5.834839	7.364905	0.96647	0.0024	1233271	3.184515	4.624005	7.487219
Ca9-22	753538	upper_aerodigestive_tract	carcinoma	aero_digestive_tract	head_and_neck	1.007715	1.383298	0.114426	1.122198	2.119928	2.59024	3.405491	0.884871	0.0145	1049138	1.481058	2.119928	4.011144
CAL-12T	753540	lung	carcinoma	lung	lung_NSCLC_not_specified	0.901179	1.224438	0.096868	1.623516	2.649587	3.549009	4.575203	0.917743	0.0063	1049143	2.22375	2.649587	3.512887
CAL-33	753541	upper_aerodigestive_tract	carcinoma	aero_digestive_tract	head_and_neck	0.811095	0.597691	0.109759	0.219468	2.302102	4.407841	6.70211	0.821754	0.0026	991168	1.721697	2.302102	3.240355
CP50-MEL-B	753545	skin	malignant_melanoma	skin	melanoma	0.737533	0.3968	0.117502	-0.900037	2.399961	5.807517	9.649026	0.835104	0.0043	991183	1.401063	2.399961	4.013008
CP66-MEL	753546	skin	malignant_melanoma	skin	melanoma	0.714616	0.588693	0.045229	0.691856	2.90385	5.369069	7.977437	0.856916	0.0015	1233273	2.056557	2.90385	3.852667
CPC-N	753547	lung	carcinoma	lung	lung_small_cell_carcinoma	0.888766	0.912883	0.087773	0.794248	2.173674	3.453504	4.875952	0.854901	0.0029	1258115	1.583377	2.173674	3.339662
CTV-1	753548	haematopoietic_and_lymphoid_tissue	haematopoietic_neoplasm	blood	acute_myeloid_leukaemia	1.065571	1.313355	0.062756	0.791682	1.706051	2.557189	3.468627	0.836579	0.0011	1257919	1.422187	1.706051	2.564456
D-542MG	753549	central_nervous_system	glioma	nervous_system	glioma	0.742355	0.434358	0.087297	0.800438	3.900845	6.920579	10.380897	0.874955	0.0078	1035703	2.482629	3.900845	6.444737
DMS-79	753551	lung	carcinoma	lung	lung_small_cell_carcinoma	0.855081	0.997062	0.083723	4.254713	5.893711	6.937652	8.346362	0.988946	0.0011	1260943	3.624456	5.893711	9.91367
DSH1	753552	urinary_tract	carcinoma	urogenital_system	Bladder	0.892198	0.91076	0.126959	0.225734	1.523566	2.84401	4.228366	0.791271	0.0048	1035705	1.141709	1.523566	1.919867
EC-GI-10	753555	oesophagus	carcinoma	aero_digestive_tract	oesophagus	0.89131	0.98739	0.07609	1.483558	3.264624	3.717534	5.076086	0.898678	0.0144	1035706	1.869357	3.264624	6.932361
EPLC-272H	753556	lung	carcinoma	lung	lung_NSCLC_squamous_cell_carcinoma	0.884933	0.912657	0.103701	1.030382	2.395408	3.611801	5.015477	0.880006	0.0104	1049225	1.726922	2.395408	3.911255
HCE-4	753559	oesophagus	carcinoma	aero_digestive_tract	oesophagus	0.870243	0.9766	0.083379	1.715752	3.034488	4.20351	5.544042	0.915215	0.0079	999947	2.427643	3.034488	4.441501
HSC-2	753562	upper_aerodigestive_tract	carcinoma	aero_digestive_tract	head_and_neck	0.872195	0.877137	0.100635	1.08433	2.494349	3.847302	5.343249	0.870841	0.0074	1049260	2.022637	2.494349	3.40502
IM-9	753563	haematopoietic_and_lymphoid_tissue	lymphoid_neoplasm	blood	Myeloma	0.849832	1.096314	0.08586	3.413798	5.060895	5.85348	7.140995	0.983331	0.0054	1324351	2.999578	5.060895	9.47116
IST-SL1	753564	lung	carcinoma	lung	lung_small_cell_carcinoma	0.849254	0.694915	0.122831	0.37842	2.31322	4.22932	6.304437	0.827787	0.0053	991189	1.272866	2.31322	3.954262
IST-SL2	753565	lung	carcinoma	lung	lung_small_cell_carcinoma	0.975362	1.425747	0.086932	1.422617	2.300251	2.995782	3.818732	0.911548	0.0027	1266179	1.950139	2.300251	3.610865
J82	753566	urinary_tract	carcinoma	urogenital_system	Bladder	0.857983	0.89241	0.100417	2.493414	4.131261	5.357079	6.870664	0.949856	0.0096	991247	2.85894	4.131261	6.914448
KNS-62	753569	lung	carcinoma	lung	lung_NSCLC_squamous_cell_carcinoma	0.811049	0.690305	0.093118	1.806912	3.722844	5.539888	7.594961	0.918828	0.0132	1101393	2.516214	3.722844	5.681024
KOSC-2	753570	upper_aerodigestive_tract	carcinoma	aero_digestive_tract	head_and_neck	0.825519	0.810064	0.100358	1.160013	2.719956	4.260241	5.959777	0.882275	0.0158	999961	2.116647	2.719956	3.708552
KP-4	753572	pancreas	carcinoma	pancreas	pancreas	0.906224	1.198726	0.107521	2.532513	4.410245	4.75475	5.920576	0.964408	0.0197	991255	2.434832	4.410245	9.577234
KYSE-140	753573	oesophagus	carcinoma	aero_digestive_tract	oesophagus	0.824276	0.677389	0.073661	2.630355	4.651534	6.412002	8.469693	0.943942	0.0014	1049268	3.273607	4.651534	7.057574
KYSE-410	753574	oesophagus	carcinoma	aero_digestive_tract	oesophagus	0.865572	1.090788	0.083619	3.774428	5.258261	6.299461	7.604213	0.984229	0.005	991258	2.407938	5.258261	9.996701
KYSE-520	753575	oesophagus	carcinoma	aero_digestive_tract	oesophagus	0.862259	1.110303	0.094561	3.559141	5.211683	5.947625	7.231076	0.9851	0.0267	1049272	2.990107	5.211683	10.187918
KYSE-70	753576	oesophagus	carcinoma	aero_digestive_tract	oesophagus	0.835787	0.909334	0.07628	4.121376	5.894772	7.102623	8.669571	0.985282	0.0038	999964	3.72588	5.894772	9.877177
LB1047-RCC	753577	kidney	carcinoma	kidney	kidney	0.825171	0.62123	0.12372	-2.029122	-0.095551	1.955806	4.114468	0.678938	0.0042	1101394	-0.629833	-0.095551	0.421962
LB2241-RCC	753578	kidney	carcinoma	kidney	kidney	0.915612	0.893136	0.09656	0.420907	1.737064	3.060092	4.451199	0.815226	0.0017	1035725	1.442786	1.737064	2.105067
LB2518-MEL	753579	skin	malignant_melanoma	skin	melanoma	0.745583	0.442235	0.15212	-0.445801	2.602349	5.637234	9.12523	0.829575	0.0087	1035726	1.43243	2.602349	4.650907
LB373-MEL-D	753581	skin	malignant_melanoma	skin	melanoma	0.79957	0.602187	0.080259	1.82869	4.031996	6.120856	8.47483	0.913905	0.0017	1233275	2.886657	4.031996	5.999875
LB771-HNC	753583	upper_aerodigestive_tract	carcinoma	aero_digestive_tract	head_and_neck	0.742568	0.338454	0.11049	-1.331964	2.809405	6.880472	11.59135	0.840345	0.0117	1035727	1.036804	2.809405	5.939682
LB831-BLC	753584	urinary_tract	carcinoma	urogenital_system	Bladder	0.887495	0.64805	0.067351	0.648921	2.529413	4.293668	6.243725	0.850002	0.005	1035728	2.029259	2.529413	3.93177
LB996-RCC	753585	kidney	carcinoma	kidney	kidney	0.770492	0.494539	0.1536	-2.340298	0.183831	2.976777	5.943164	0.722977	0.0245	1035729	-1.027312	0.183831	1.082524
LCLC-103H	753586	lung	carcinoma	lung	lung_NSCLC_large_cell	0.875542	0.950839	0.074428	1.122132	2.389058	3.647298	5.004212	0.877174	0.0074	991260	2.07798	2.389058	2.920447
LU-134-A	753588	lung	carcinoma	lung	lung_small_cell_carcinoma	0.801341	0.91732	0.083125	0.120894	1.440574	2.83957	4.32702	0.799942	0.003	1265199	1.157447	1.440574	1.730896
LU-165	753589	lung	carcinoma	lung	lung_small_cell_carcinoma	0.847701	0.989267	0.076692	4.219851	5.854882	6.918577	8.358809	0.98884	0.0018	1266291	3.653604	5.854882	9.833925
LXF-289	753592	lung	carcinoma	lung	lung_NSCLC_adenocarcinoma	1.009469	1.180539	0.057022	1.057872	2.08648	2.782414	3.700571	0.871278	0.0019	1101395	1.729339	2.08648	3.064878
MS-1	753594	lung	carcinoma	lung	lung_small_cell_carcinoma	1.111238	1.639416	0.091315	0.800383	1.499667	2.175974	2.876108	0.831155	0.0082	1263603	1.268062	1.499667	1.874333
MZ1-PC	753595	pancreas	carcinoma	pancreas	pancreas	0.840798	0.984544	0.07474	0.989339	2.361691	3.651391	5.019884	0.870914	0.0087	1035734	1.626315	2.361691	3.522765
MZ7-mel	753596	skin	malignant_melanoma	skin	melanoma	0.879262	0.915908	0.141615	0.935658	2.295321	3.55572	4.972535	0.859359	0.0067	1049189	1.742626	2.295321	3.403649
NCI-H1304	753599	lung	carcinoma	lung	lung_small_cell_carcinoma	0.847866	0.920134	0.084106	3.019919	4.650548	5.863402	7.367273	0.971273	0.0022	1259431	3.105459	4.650548	7.40451
NCI-H1563	753600	lung	carcinoma	lung	lung_NSCLC_adenocarcinoma	0.636552	0.262825	0.107089	0.461976	6.154092	11.817693	18.689408	0.906985	0.0123	1101467	3.277659	6.154092	11.549617
NCI-H292	753604	lung	carcinoma	lung	lung_NSCLC_adenocarcinoma	0.822498	0.921194	0.127371	-0.047228	1.259138	2.634629	4.105274	0.776122	0.0063	1035776	0.843918	1.259138	1.689597
NCI-H510A	753605	lung	carcinoma	lung	lung_small_cell_carcinoma	0.872304	1.088737	0.082839	2.712306	4.153907	5.024691	6.252289	0.970238	0.005	1308625	2.857779	4.153907	7.146234
PC-14	753608	lung	carcinoma	lung	lung_NSCLC_adenocarcinoma	0.803258	0.678065	0.074012	2.405166	4.379901	6.20223	8.267028	0.935061	0.003	1049323	3.154831	4.379901	6.375641
SK-MM-2	753612	haematopoietic_and_lymphoid_tissue	lymphoid_neoplasm	blood	Myeloma	0.873644	1.057898	0.079489	2.190964	3.440388	4.484938	5.712898	0.951486	0.0046	1325051	2.665143	3.440388	5.074348
TE-15	753614	oesophagus	carcinoma	aero_digestive_tract	oesophagus	0.848353	1.059012	0.072635	3.854202	5.449154	6.405257	7.758802	0.985984	0.0051	1258479	2.86341	5.449154	9.974792
SIMA	753620	autonomic_ganglia	neuroblastoma	nervous_system	neuroblastoma	1.062345	1.264516	0.100481	0.506012	1.396854	2.218726	3.062552	0.798242	0.0009	1233265	1.140728	1.396854	1.82517
TE-1	753621	oesophagus	carcinoma	aero_digestive_tract	oesophagus	0.849637	0.824247	0.117869	1.567135	3.148235	4.580763	6.217642	0.907215	0.0041	1049212	2.330406	3.148235	4.726986
TE-10	753622	oesophagus	carcinoma	aero_digestive_tract	oesophagus	0.954103	1.301076	0.08583	1.466504	2.380475	3.230155	4.152864	0.906371	0.0025	1035752	2.088658	2.380475	3.008169
TE-8	753623	oesophagus	carcinoma	aero_digestive_tract	oesophagus	0.882461	0.96323	0.114987	0.824607	2.067467	3.307929	4.643519	0.856243	0.0106	1049215	1.647993	2.067467	2.633552
CAPAN-1	753624	pancreas	carcinoma	pancreas	pancreas	0.847735	0.930296	0.127486	3.813671	5.609029	6.764901	8.307235	0.982227	0.0082	1049161	3.371482	5.609029	9.737786
OVCAR-3	905933	ovary	carcinoma	urogenital_system	ovary	0.768732	0.526287	0.053869	2.886853	5.598234	8.021845	10.879029	0.936165	0.0027	1035840	3.635885	5.598234	8.940378
PC-3	905934	prostate	carcinoma	urogenital_system	prostate	0.886609	1.094598	0.087288	2.03706	3.197608	4.249899	5.433142	0.93911	0.0058	1049324	2.570029	3.197608	4.335639
DU-145	905935	prostate	carcinoma	urogenital_system	prostate	0.893133	1.12929	0.065368	1.970342	3.090116	4.085355	5.21483	0.938537	0.0019	999955	2.573246	3.090116	4.159024
HCT-116	905936	large_intestine	carcinoma	digestive_system	large_intestine	0.826095	0.964328	0.094497	3.971236	5.688066	6.803241	8.29118	0.986378	0.017	1049247	3.511115	5.688066	9.791817
HCT-15	905937	large_intestine	carcinoma	digestive_system	large_intestine	0.899565	0.858449	0.094602	0.370489	1.727086	3.123101	4.574212	0.804742	0.011	1035781	1.408895	1.727086	2.046733
HL-60	905938	haematopoietic_and_lymphoid_tissue	haematopoietic_neoplasm	blood	acute_myeloid_leukaemia	1.031984	0.783654	0.10539	-1.976574	-0.544362	0.903902	2.33606	0.576882	0.0011	1254531	-0.879998	-0.544362	-0.226886
HT-29	905939	large_intestine	carcinoma	digestive_system	large_intestine	0.845614	0.981162	0.094847	4.382282	5.983163	7.092083	8.521406	0.989455	0.0046	1049265	3.649015	5.983163	9.737946
K-562	905940	haematopoietic_and_lymphoid_tissue	haematopoietic_neoplasm	blood	chronic_myeloid_leukaemia	0.993036	1.290671	0.073267	1.050374	1.934762	2.805563	3.706226	0.869546	0.0014	1258227	1.744594	1.934762	2.176
NCI-H226	905941	lung	carcinoma	lung	lung_NSCLC_squamous_cell_carcinoma	0.878358	1.177198	0.086445	2.122378	3.371566	4.197264	5.322545	0.954373	0.0117	1049300	2.497493	3.371566	6.017825
NCI-H23	905942	lung	carcinoma	lung	lung_NSCLC_adenocarcinoma	0.831069	0.738134	0.095534	0.49219	2.159625	3.83821	5.665313	0.845269	0.0015	1049302	1.676446	2.159625	2.872288
NCI-H460	905943	lung	carcinoma	lung	lung_NSCLC_large_cell	0.843316	0.844918	0.126542	0.843877	2.311593	3.762336	5.350359	0.851558	0.0113	1049305	1.784035	2.311593	3.117734
NCI-H522	905944	lung	carcinoma	lung	lung_NSCLC_adenocarcinoma	0.871962	1.07027	0.126052	2.762846	4.530904	5.263051	6.595612	0.968765	0.0526	999974	2.616784	4.530904	9.145475
T47D	905945	breast	carcinoma	breast	breast	0.817878	0.775925	0.087673	0.747983	2.319754	3.942002	5.69164	0.847093	0.0009	1049148	1.908489	2.319754	2.884315
MCF7	905946	breast	carcinoma	breast	breast	0.904802	0.852437	0.088462	0.844811	2.365858	3.755185	5.316574	0.851241	0.0047	1035812	1.514492	2.365858	3.964563
786-0	905947	kidney	carcinoma	kidney	kidney	1.078668	1.61771	0.091989	-0.664825	0.01545	0.68992	1.357385	0.605075	0.0062	991216	-0.141198	0.01545	0.172944
A498	905948	kidney	carcinoma	kidney	kidney	0.663856	0.258411	0.09568	-1.209991	4.124217	9.728098	16.237668	0.877835	0.0089	1035691	2.419223	4.124217	7.166488
A549	905949	lung	carcinoma	lung	lung_NSCLC_adenocarcinoma	0.834987	0.630172	0.086356	0.479408	2.429532	4.39625	6.522522	0.838715	0.0044	1049256	1.94603	2.429532	3.198365
ACHN	905950	kidney	carcinoma	kidney	kidney	0.889923	1.097487	0.129545	0.945915	2.036996	3.107791	4.261765	0.861436	0.0206	991246	1.668506	2.036996	2.582191
BT-549	905951	breast	carcinoma	breast	breast	0.796832	0.577538	0.093689	0.902643	3.13187	5.313797	7.767108	0.868047	0.0034	1035841	2.320408	3.13187	4.557918
CCRF-CEM	905952	haematopoietic_and_lymphoid_tissue	haematopoietic_neoplasm	blood	lymphoblastic_leukemia	1.193589	1.419474	0.099676	-0.395079	0.360597	1.108928	1.827404	0.680823	0.0041	1254195	0.102746	0.360597	0.578335
SK-MEL-28	905954	skin	malignant_melanoma	skin	melanoma	0.870384	0.806003	0.107407	0.57377	2.083623	3.567118	5.171614	0.837548	0.0139	991152	1.644165	2.083623	2.790456
SK-MEL-2	905955	skin	malignant_melanoma	skin	melanoma	0.857511	0.976191	0.111427	1.647514	2.973619	4.149428	5.508338	0.922767	0.0074	991151	2.278139	2.973619	4.309585
SK-MEL-5	905956	skin	malignant_melanoma	skin	melanoma	0.814209	0.746911	0.126063	2.232385	4.159749	5.767651	7.656542	0.936226	0.0188	1233278	2.804667	4.159749	6.779007
Hs-578-T	905957	breast	carcinoma	breast	breast	1.028854	1.12813	0.091718	0.637743	1.655391	2.553032	3.528411	0.82764	0.0095	1101435	1.311036	1.655391	2.345582
SK-OV-3	905959	ovary	carcinoma	urogenital_system	ovary	0.863627	0.962564	0.081151	2.473205	3.919063	5.069521	6.47418	0.953168	0.003	1311963	2.874279	3.919063	6.024523
MDA-MB-231	905960	breast	carcinoma	breast	breast	0.868118	0.972056	0.083053	1.9306	3.257056	4.43648	5.787057	0.928238	0.0157	1035813	2.575014	3.257056	4.60167
COLO-205	905961	large_intestine	carcinoma	digestive_system	large_intestine	0.852004	0.961782	0.157236	4.297812	6.03132	7.161099	8.662517	0.988657	0.0218	1308121	3.429156	6.03132	10.422587
SW620	905962	large_intestine	carcinoma	digestive_system	large_intestine	0.81868	0.605125	0.046678	1.798106	3.975383	6.007484	8.300472	0.908504	0.0005	991166	2.706348	3.975383	6.19896
CAKI-1	905963	kidney	carcinoma	kidney	kidney	0.958515	1.038326	0.113523	0.440338	1.557534	2.650382	3.793573	0.808869	0.0191	991157	1.254424	1.557534	1.926379
RPMI-8226	905964	haematopoietic_and_lymphoid_tissue	haematopoietic_neoplasm	blood	Myeloma	1.101366	1.460551	0.072859	0.880686	1.665794	2.272344	2.950919	0.84389	0.0015	1258563	1.446478	1.665794	2.192716
SR	905965	haematopoietic_and_lymphoid_tissue	lymphoid_neoplasm	blood	lymphoid_neoplasm_other	0.882109	0.941148	0.095476	0.898855	2.175063	3.44485	4.803215	0.868698	0.0041	1267691	1.799277	2.175063	2.748257
NCI-H322M	905967	lung	carcinoma	lung	lung_NSCLC_adenocarcinoma	0.848417	1.030519	0.104546	3.355838	4.982206	5.917684	7.291549	0.979566	0.027	1324603	3.078288	4.982206	8.92248
IGROV-1	905968	ovary	carcinoma	urogenital_system	ovary	0.724303	0.703003	0.068388	0.810202	2.647132	4.650101	6.822074	0.853055	0.0056	991245	1.937899	2.647132	3.286833
OVCAR-5	905969	ovary	carcinoma	urogenital_system	ovary	0.762069	0.501155	0.102727	0.426579	3.02334	5.654608	8.604252	0.850093	0.0038	1101487	2.13963	3.02334	4.48307
EKVX	905970	lung	carcinoma	lung	lung_NSCLC_adenocarcinoma	0.904493	1.232403	0.071772	1.865887	3.145647	3.750973	4.782622	0.935756	0.0074	1101383	2.382632	3.145647	5.999355

HCC2998	905971	large_intestine	carcinoma	digestive_system	large_intestine	0.851913	0.988853	0.100591	3.84505	5.565049	6.561182	7.987005	0.985219	0.0031	1049175	3.373458	5.565049	9.916584
HOP-62	905972	lung	carcinoma	lung	lung_NSCLC_adenocarcinoma	0.866919	0.997948	0.112866	2.29076	3.788898	4.809712	6.166142	0.94897	0.016	1233274	2.642982	3.788898	6.682708
HOP-92	905973	lung	carcinoma	lung	lung_NSCLC_large_cell	0.882797	0.832925	0.13194	0.668394	2.136825	3.58529	5.140282	0.843907	0.0129	999958	1.620424	2.136825	2.911908
LOXIMVI	905974	skin	malignant_melanoma	skin	melanoma	0.912996	1.067004	0.149278	1.258328	2.433593	3.471925	4.655504	0.885053	0.0069	991191	1.870058	2.433593	3.685922
M14	905975	skin	malignant_melanoma	skin	melanoma	0.83262	0.817301	0.100138	2.302242	4.031557	5.449633	7.145794	0.946656	0.0036	1049280	2.837561	4.031557	6.413751
UACC-62	905976	skin	malignant_melanoma	skin	melanoma	0.958662	1.200028	0.114157	1.078001	2.056813	2.984496	3.972669	0.870271	0.0055	1049153	1.733658	2.056813	2.587345
UACC-257	905977	skin	malignant_melanoma	skin	melanoma	0.802958	0.672703	0.126534	1.898068	3.991092	5.835779	7.993598	0.918485	0.0051	1049217	2.598336	3.991092	6.404268
RXF393	905978	kidney	carcinoma	kidney	kidney	0.867183	0.903066	0.093338	1.999442	3.447878	4.739176	6.209046	0.942902	0.0042	1049203	2.588995	3.447878	4.975315
SN12C	905979	kidney	carcinoma	kidney	kidney	0.87249	0.75896	0.121608	1.252104	3.016363	4.526142	6.324337	0.886264	0.0107	999937	2.080983	3.016363	5.220622
TK10	905980	kidney	carcinoma	kidney	kidney	0.779548	0.643797	0.085072	0.233508	2.197582	4.310651	6.560076	0.826335	0.0038	1035755	1.477651	2.197582	2.944925
U031	905981	kidney	carcinoma	kidney	kidney	0.840436	0.839295	0.112852	0.757741	2.216205	3.696439	5.288546	0.846724	0.0042	1035681	1.77573	2.216205	2.848091
SNB75	905982	central_nervous_system	glioma	nervous_system	glioma	0.807464	0.632629	0.079686	1.35344	3.383383	5.35244	7.56728	0.892056	0.0026	1311683	2.579687	3.383383	4.808225
U251	905983	central_nervous_system	glioma	nervous_system	glioma	0.870245	1.060828	0.108865	2.482909	4.052487	4.905171	6.199773	0.957331	0.0101	999944	2.689979	4.052487	7.881602
SF539	905984	central_nervous_system	glioma	nervous_system	glioma	0.714265	0.355937	0.09334	0.614394	4.504205	8.358829	12.833157	0.889727	0.0089	1035745	2.670995	4.504205	7.697658
SF295	905985	central_nervous_system	glioma	nervous_system	glioma	0.856217	0.850031	0.090071	1.914454	3.515424	4.843368	6.430002	0.932085	0.0089	1101350	2.563904	3.515424	5.5457
SF268	905986	central_nervous_system	glioma	nervous_system	glioma	0.848101	0.810238	0.090413	1.203595	2.736751	4.219544	5.856401	0.885391	0.0088	1035744	2.190203	2.736751	3.683412
KM12	905989	large_intestine	carcinoma	digestive_system	large_intestine	0.629639	0.860063	0.145049	-1.879576	-0.346456	1.408282	3.380637	0.597351	0.0101	1049182	-0.855206	-0.346456	0.197948
OVCAR-4	905990	ovary	carcinoma	urogenital_system	ovary	0.884739	1.165678	0.076466	2.152831	3.253685	4.226534	5.329797	0.950426	0.0118	1101398	2.627913	3.253685	4.396408
OVCAR-8	905991	ovary	carcinoma	urogenital_system	ovary	0.868727	1.020344	0.090377	2.384296	3.816133	4.856726	6.173959	0.956187	0.0044	1049319	2.711046	3.816133	6.446792
BxPC-3	906693	pancreas	carcinoma	pancreas	pancreas	0.82022	0.949917	0.067186	4.311967	6.02211	7.211247	8.761078	0.989204	0.0028	991298	3.799439	6.02211	9.981929
BHT-101	906696	thyroid	carcinoma	thyroid	thyroid	0.724433	0.441482	0.079761	-0.580725	2.311153	5.414463	8.84442	0.827087	0.0025	1101466	1.612661	2.311153	3.26407
Becker	906746	central_nervous_system	glioma	nervous_system	glioma	0.877009	0.634209	0.0734	0.980569	2.928462	4.784905	6.828556	0.868359	0.0036	1035695	2.30071	2.928462	4.152129
BE-13	906763	haematopoietic_and_lymphoid_tissue	haematopoietic_neoplasm	blood	lymphoblastic_leukemia	0.840092	0.676667	0.130024	0.58689	2.460818	4.260375	6.260454	0.86014	0.0022	1309493	1.674722	2.460818	3.751197
ARH-77	906765	haematopoietic_and_lymphoid_tissue	haematopoietic_neoplasm	blood	Myeloma	0.848733	1.032898	0.098035	4.704366	6.265662	7.281762	8.64686	0.993225	0.0087	1324379	3.738153	6.265662	10.679338
AGS	906790	stomach	carcinoma	digestive_system	stomach	0.997459	1.493961	0.106886	1.230913	2.002042	2.73218	3.505218	0.88417	0.0213	991249	1.765471	2.002042	2.418125
ABC-1	906791	lung	carcinoma	lung	lung_NSCLC_adenocarcinoma	0.87943	0.869006	0.061179	1.955071	3.421356	4.734483	6.225045	0.929194	0.0014	1049259	2.742159	3.421356	4.76686
A2058	906792	skin	malignant_melanoma	skin	melanoma	0.872334	0.917956	0.084749	1.652623	3.021694	4.305383	5.734898	0.916458	0.006	991230	2.440671	3.021694	4.134805
A375	906793	skin	malignant_melanoma	skin	melanoma	0.832666	0.874672	0.090816	0.273467	1.646366	3.070399	4.579483	0.798647	0.0059	1049254	1.366455	1.646366	1.966924
A253	906794	salivary_gland	carcinoma	aero_digestive_tract	head_and_neck	0.881944	0.923811	0.097531	1.65931	3.396894	4.303948	5.772858	0.916976	0.0062	1311991	2.154202	3.396894	6.927123
8305C	906795	thyroid	carcinoma	thyroid	thyroid	0.790852	0.54952	0.116689	0.561663	2.953697	5.263975	7.869472	0.861302	0.003	1049224	2.019867	2.953697	4.625475
647-V	906797	urinary_tract	carcinoma	urogenital_system	Bladder	0.856674	0.940589	0.1091	1.336191	2.666819	3.939413	5.356135	0.89525	0.0119	1049220	2.121675	2.666819	3.659637
639-V	906798	urinary_tract	carcinoma	urogenital_system	Bladder	0.859165	0.846767	0.081693	1.623629	3.097138	4.518821	6.083962	0.908889	0.0016	991210	2.508089	3.097138	4.123984
697	906800	haematopoietic_and_lymphoid_tissue	haematopoietic_neoplasm	blood	lymphoblastic_leukemia	0.981136	1.010336	0.052786	0.230122	1.422393	2.600034	3.834036	0.792253	0.0006	1257751	0.9629	1.422393	2.060093
BT-20	906801	breast	carcinoma	breast	breast	0.849662	0.930938	0.082324	3.711066	5.421686	6.629015	8.146222	0.981179	0.0021	1049310	3.457806	5.421686	8.826562
ACN	906803	autonomic_ganglia	neuroblastoma	nervous_system	neuroblastoma	0.777469	0.56369	0.194357	0.794301	3.33507	5.713819	8.480816	0.866594	0.0363	1049157	1.840092	3.33507	6.1135
A2780	906804	ovary	carcinoma	urogenital_system	ovary	0.978201	0.990376	0.124754	0.030405	1.181092	2.339828	3.518227	0.764861	0.0035	1049243	0.823508	1.181092	1.507858
COR-L105	906805	lung	carcinoma	lung	lung_NSCLC_adenocarcinoma	0.887616	0.944298	0.143358	1.344324	2.820647	3.89053	5.293996	0.897417	0.0111	1049218	1.838126	2.820647	5.444547
CRO-AP2	906807	haematopoietic_and_lymphoid_tissue	lymphoid_neoplasm	blood	B_cell_lymphoma	0.904497	1.299642	0.075331	2.218626	3.664238	4.326499	5.398373	0.960137	0.0058	1307309	1.93975	3.664238	8.100823
COR-L88	906808	lung	carcinoma	lung	lung_small_cell_carcinoma	0.849089	1.043409	0.071671	5.031822	6.561519	7.562329	8.908753	0.995227	0.0037	1261503	3.998579	6.561519	10.606187
COLO-824	906812	breast	carcinoma	breast	breast	0.897619	1.099606	0.098564	1.913127	3.69231	4.213984	5.463842	0.938375	0.0148	1035759	2.220486	3.69231	7.718361
COLO-800	906813	skin	malignant_melanoma	skin	melanoma	0.984178	1.049514	0.111682	-0.151204	0.936386	2.036006	3.146045	0.736246	0.0076	1035758	0.632562	0.936386	1.235095
COLO-792	906814	skin	malignant_melanoma	skin	melanoma	0.911481	1.22299	0.181137	0.517168	1.480594	2.438143	3.445879	0.815776	0.0523	991205	1.075789	1.480594	1.976369
COLO-741	906815	large_intestine	carcinoma	digestive_system	large_intestine	0.831966	0.712858	0.078292	1.496485	3.277411	4.993983	6.907071	0.895711	0.0029	991204	2.56663	3.277411	4.541513
COLO-680N	906817	oesophagus	carcinoma	aero_digestive_tract	oesophagus	0.722923	0.440073	0.035732	3.621702	6.798091	9.826726	13.364921	0.949007	0.0008	1035757	4.386813	6.798091	10.595016
COLO-679	906818	skin	malignant_melanoma	skin	melanoma	0.75386	0.394864	0.128524	-0.81141	2.563918	5.974284	9.807898	0.832484	0.0122	1035756	1.463058	2.563918	4.40624
CHP-212	906820	autonomic_ganglia	neuroblastoma	nervous_system	neuroblastoma	1.1177	1.113445	0.084162	-0.078078	0.91642	1.854087	2.771404	0.739231	0.0013	991202	0.699513	0.91642	1.133372
CFPAC-1	906821	pancreas	carcinoma	pancreas	pancreas	0.836445	0.825831	0.109542	1.828563	3.430921	4.857882	6.500382	0.922894	0.0094	991197	2.525894	3.430921	5.123069
CCF-STTG1	906823			nervous_system	glioma	0.775792	0.536134	0.071849	0.869434	3.215456	5.617529	8.26678	0.869527	0.001	1233305	2.469183	3.215456	4.375222
Ca-Ski	906824	cervix	carcinoma	urogenital_system	cervix	0.912078	1.154847	0.088341	2.41187	3.723961	4.52269	5.648485	0.961933	0.0103	1233264	2.692109	3.723961	6.499683
CAL-120	906826	breast	carcinoma	breast	breast	0.82052	0.683344	0.081269	0.490002	2.28055	4.127855	6.107136	0.838789	0.0023	991159	1.868939	2.28055	2.84344
CAL-72	906827	bone	osteosarcoma	bone	osteosarcoma	0.965434	1.193138	0.056706	1.321976	2.299323	3.231404	4.228776	0.89505	0.0055	991172	2.066535	2.299323	2.959184
CAL-62	906828	thyroid	carcinoma	thyroid	thyroid	0.916572	1.070156	0.129276	1.27497	2.653375	3.408056	4.602065	0.883592	0.0098	1049152	1.72258	2.653375	5.147824
C32	906830	skin	malignant_melanoma	skin	melanoma	0.871781	0.92971	0.070931	1.102182	2.382144	3.678077	5.049519	0.878074	0.0005	991139	2.082975	2.382144	2.829686
Daudi	906831	haematopoietic_and_lymphoid_tissue	lymphoid_neoplasm	blood	Burkitt_lymphoma	0.846702	0.982543	0.12921	3.928496	5.569657	6.667451	8.119187	0.98742	0.0022	1323931	3.278497	5.569657	9.545072
Daoy	906833	central_nervous_system	primitive_neuroectodermal_tumour-medulloblastoma	nervous_system	medulloblastoma	0.877429	0.748091	0.07856	0.532322	2.120266	3.736162	5.451263	0.831122	0.0028	991212	1.789352	2.120266	2.572194
D-283MED	906834	central_nervous_system	primitive_neuroectodermal_tumour-medulloblastoma	nervous_system	medulloblastoma	1.111102	1.307936	0.081764	0.571288	1.424173	2.193838	2.980168	0.80641	0.001	1101407	1.212525	1.424173	1.718292
DBTRG-05MG	906835	central_nervous_system	glioma	nervous_system	glioma	0.845236	0.915639	0.103346	2.898957	4.571758	5.736435	7.234907	0.965586	0.01	1049221	3.045544	4.571758	7.912248
DEL	906836	haematopoietic_and_lymphoid_tissue	lymphoid_neoplasm	blood	lymphoid_neoplasm_other	0.871983	1.092293	0.08779	3.893318	5.389528	6.327254	7.616198	0.986769	0.0133	1256967	2.333804	5.389528	9.759317
Detroit562	906837	upper_aerodigestive_tract	carcinoma	aero_digestive_tract	head_and_neck	0.854593	0.734889	0.074135	1.132112	2.98166	4.791001	6.722003	0.875245	0.0093	991213	1.656067	2.98166	5.089356
DG-75	906838	haematopoietic_and_lymphoid_tissue	lymphoid_neoplasm	blood	Burkitt_lymphoma	0.852822	0.828763	0.10951	1.898999	3.547034	4.958149	6.613508	0.932691	0.0012	1267411	2.507051	3.547034	5.537934
DK-MG	906839	central_nervous_system	glioma	nervous_system	glioma	0.791747	0.468408	0.110821	-0.943347	1.721865	4.47287	7.471108	0.800445	0.0018	1049222	1.046518	1.721865	2.619602
DJM-1	906841	skin	adnexal_tumour	skin	skin_other	0.876174	1.05091	0.054561	2.37392	3.595964	4.691127	5.93746	0.953107	0.0019	1035704	2.925126	3.595964	4.789219
DoTc2-4510	906843	cervix	carcinoma	urogenital_system	cervix	0.827551	0.930866	0.162312	3.869477	5.69568	6.897858	8.486554	0.982844	0.0286	1035761	3.282438	5.69568	10.131896
DU-4475	906844	breast	carcinoma	breast	breast	0.902148	0.959756	0.087862	1.414474	2.751964	3.871582	5.204988	0.904067	0.0051	1259767	2.158662	2.751964	4.535967
EB2	906846	haematopoietic_and_lymphoid_tissue	lymphoid_neoplasm	blood	Burkitt_lymphoma	0.839807	1.07856	0.074571	5.750222	7.167679	8.204923	9.518633	0.997996	0.0025	1325107	4.32169	7.167679	11.197419
EB-3	906847	haematopoietic_and_lymphoid_tissue	lymphoid_neoplasm	blood	Burkitt_lymphoma	0.849742	1.034553	0.084762	5.445024	6.936696	7.974961	9.320034	0.996675	0.0073	1324127	4.137937	6.936696	11.180129
ECC10	906848	stomach	carcinoma	digestive_system	stomach	0.918504	0.734215	0.110168	-0.519309	1.149801	2.715976	4.432371	0.764519	0.0048	1261727	0.575276	1.149801	2.001674
EFM-19	906851	breast	carcinoma	breast	breast	0.978214	1.437725	0.05696	1.679433	2.677537	3.160534	3.953267	0.924506	0.0009	991219	2.218719	2.677537	4.762159
EFO-27	906852	ovary	carcinoma	urogenital_system	ovary	0.939186	1.277412	0.085686	1.519761	2.604691	3.255352	4.20368	0.912017	0.0025	1035763	2.063663	2.604691	4.586178
EGI-1	906853	biliary_tract	carcinoma	digestive_system	biliary_tract	0.9437	1.090855	0.079756	1.328351	2.433901	3.441497	4.554797	0.888889	0.0064	991220	2.093975	2.433901	3.399271
EM-2	906855	haematopoietic_and_lymphoid_tissue	haematopoietic_neoplasm	blood	chronic_myeloid_leukaemia	0.947072	1.341636	0.071604	1.612324	2.542077	3.320253	4.220233	0.92435	0.0017	1309381	2.1942	2.542077	3.671147
EoL-1-cell	906856	haematopoietic_and_lymphoid_tissue	haematopoietic_neoplasm	blood	haematopoietic_neoplasm_other	1.164932	1.477995	0.145737	0.018505	0.794369	1.444649	2.133457	0.737657	0.005	1322979	0.419904	0.794369	1.493722
ETK-1	906861	biliary_tract	carcinoma	digestive_system	biliary_tract	0.854033	0.978279	0.11108	2.874845	4.56539	5.569131	6.991814	0.967725	0.0054	1035709	2.921894	4.56539	8.431445
EVSA-T	906862	breast	carcinoma	breast	breast	0.899424	1.116658	0.127989	1.702938	3.011347	3.841561	4.998893	0.919344	0.0077	1233281	2.212374	3.011347	5.900005
FADU	906863	upper_aerodigestive_tract	carcinoma	aero_digestive_tract	head_and_neck	0.832141	0.686997	0.1068	1.64842	3.576006	5.333189	7.36283	0.901543	0.0089	999956	2.527953	3.576006	5.509875
FTC-133	906864	thyroid	carcinoma	thyroid	thyroid	0.81496	0.653276	0.101224	1.580472	3.604782	5.454747	7.588855	0.907885	0.0047	1049228	2.536963	3.604782	5.606259
G-361	906865	skin	malignant_melanoma	skin	melanoma	0.862189	0.98276	0.114689	1.466054	2.746979	3.940893	5.277071	0.905376	0.0087	1035767	2.193432	2.746979	3.826763
GAMG	906868	central_nervous_system	glioma	nervous_system	glioma	0.804421	0.725501	0.128967	1.427711	3.282045	4.966444	6.927482	0.900086	0.0199	1049230	2.277373	3.282045	5.258853
GCIY	906869	stomach	carcinoma	digestive_system	stomach	0.854564	1.005838	0.123252	0.220803	1.401659	2.628387	3.923494	0.783503	0.0078	999946	1.08206	1.401659	1.73908
GDM-1	906870	haematopoietic_and_lymphoid_tissue	haematopoietic_neoplasm	blood	acute_myeloid_leukaemia	0.866231	1.028762	0.084338	2.218177	3.529691	4.618508	5.919767	0.948022	0.0051	1266627	2.582226	3.529691	5.465363
GI-1	906871	central_nervous_system	glioma	nervous_system	glioma	0.812875	0.818971	0.107623	0.908238	2.440039	3.959893	5.641389	0.875641	0.0042	1035713	1.84533	2.440039	3.392799
GI-ME-N	906872	autonomic_ganglia	neuroblastoma	nervous_system	neuroblastoma	0.916164	0.940685	0.079583	-0.459402	0.767522	2.036551	3.331293	0.714636	0.008	991186	0.51975	0.767522	0.99784
GMS-10	906873	central_nervous_system	glioma	nervous_system	glioma	0.860124	0.948841	0.085327	1.011637	2.280654	3.55716	4.929056	0.868354	0.001	1049231	1.958563	2.280654	2.76163
GOTO	906875	autonomic_ganglia	neuroblastoma	nervous_system	neuroblastoma	0.946222	0.919165	0.110869	-0.60483	0.634983	1.918574	3.220853	0.701479	0.0089	1101416	0.276647	0.634983	0.951395
GR-ST	906877	haematopoietic_and_lymphoid_tissue	haematopoietic_neoplasm	blood	lymphoblastic_leukemia	0.855877	0.739751	0.15152	0.322966	2.03345	3.676951	5.50498	0.832191	0.0102	1255987	1.327697	2.03345	3.184113
GCT	906999	soft_tissue	malignant_fibrous_histiocytoma-pleomorphic_sarcoma	soft_tissue	soft_tissue_other	0.968796	0.975196	0.098518	0.355202	1.529017	2.698615	3.904388	0.802425	0.0068	1035770	1.251115	1.529017	1.819449
ESS-1	907000	endometrium	carcinoma	urogenital_system	endometrium	0.936053	1.218489	0.099584	1.448852	2.496018	3.321139	4.323293	0.902609	0.0045	1035764	2.065268	2.496018	4.079194
GT3TKB	907041	stomach	carcinoma	digestive_system	stomach	0.873055	0.671372	0.113331	-0.020652	1.911985	3.790973	5.860704	0.808928	0.0102	1035714	1.151484	1.911985	3.341821
H4	907042	central_nervous_system	glioma	nervous_system	glioma	0.831605	0.621761	0.082581	1.035163	3.0435	5.018241	7.207211	0.870854	0.0036	991229	2.309621	3.0435	4.297908
H9	907043	haematopoietic_and_lymphoid_tissue	lymphoid_neoplasm	blood	lymphoid_neoplasm_other	1.021927	1.618815	0.084402	1.257293	2.962762	2.349087	2.974927	0.883581	0.0025	1254307	1.564738	2.962762	8.169135
HC-1	907044	haematopoietic_and_lymphoid_tissue	lymphoid_neoplasm	blood	hairy_cell_leukaemia	0.837442	0.8348	0.076206	2.997259	4.721227	6.106598	7.749775	0.964082	0.0022	1264779	3.239533	4.721227	7.438345
HCC1419	907045	breast	carcinoma	breast	breast	0.787607	0.544484	0.165444	0.855675	4.187636	6.782165	9.713351	0.87366	0.054	1101423	1.88106	4.187636	8.554433
HCC1569	907046	breast	carcinoma	breast	breast	0.826588	0.789195	0.105932	0.401857	1.93995	3.525284	5.222702	0.822145	0.0037	1049242	1.534651	1.93995	2.460496

HCC1806	907047	breast	carcinoma	breast	breast	0.881446	1.135232	0.078804	1.942963	3.109429	4.070909	5.218255	0.9409	0.0082	991231	2.480488	3.109429	4.599062
HCC70	907048	breast	carcinoma	breast	breast	0.760672	0.586006	0.064902	1.457508	3.696183	5.99981	8.517428	0.896886	0.0046	991233	2.297692	3.696183	5.328617
HCE-T	907049	upper_aerodigestive_tract	carcinoma	aero_digestive_tract	head_and_neck	0.984227	1.2384	0.10086	0.575249	1.76828	2.143224	3.005356	0.810495	0.0068	1049176	0.87358	1.76828	4.877458
HD-MY-Z	907050	haematopoietic_and_lymphoid_tissue	lymphoid_neoplasm	blood	Hodgkin_lymphoma	0.863514	0.754513	0.086708	1.170029	2.924723	4.261211	5.988048	0.874589	0.0117	1101428	2.057157	2.924723	5.557457
HEC-1	907051	endometrium	carcinoma	urogenital_system	endometrium	0.875331	1.025283	0.105508	1.746646	2.987167	4.115312	5.386486	0.920769	0.0032	1049248	2.363369	2.987167	4.158227
HEL	907053	haematopoietic_and_lymphoid_tissue	haematopoietic_neoplasm	blood	acute_myeloid_leukaemia	0.998394	1.112646	0.061134	0.907503	1.944945	2.889532	3.907893	0.855974	0.0014	991235	1.73979	1.944945	2.521253
HGC-27	907055	stomach	carcinoma	digestive_system	stomach	0.892602	1.002955	0.121473	1.090333	2.378647	3.421381	4.691869	0.878616	0.007	1049250	1.719173	2.378647	4.162548
HH	907056	haematopoietic_and_lymphoid_tissue	lymphoid_neoplasm	blood	T_cell_leukemia	0.841809	0.776306	0.116423	1.328223	3.013186	4.542178	6.304219	0.890595	0.006	1254419	2.210645	3.013186	4.689747
HLE	907057	liver	carcinoma	digestive_system	liver	0.883722	0.723813	0.092452	0.206723	1.846952	3.514862	5.281442	0.809638	0.0039	1049252	1.494557	1.846952	2.293125
HMV-II	907058	skin	malignant_melanoma	skin	melanoma	0.847323	0.893768	0.124627	0.979687	2.367435	3.710042	5.197337	0.8654	0.0068	991236	1.851604	2.367435	3.278543
HN	907059	upper_aerodigestive_tract	carcinoma	aero_digestive_tract	head_and_neck	0.75897	0.495765	0.066219	1.4435	4.065303	6.663864	9.592775	0.89209	0.0026	999957	3.008223	4.065303	5.839814
HOS	907060	bone	osteosarcoma	bone	osteosarcoma	0.980568	0.741468	0.103993	-1.044928	0.529735	2.020466	3.573041	0.682983	0.0087	1035785	-0.066647	0.529735	1.160094
HSC-3	907061	upper_aerodigestive_tract	carcinoma	aero_digestive_tract	head_and_neck	0.917966	1.19105	0.085352	1.531229	2.713139	3.411357	4.441228	0.910823	0.0091	999960	2.120582	2.713139	5.248652
HSC-4	907062	upper_aerodigestive_tract	carcinoma	aero_digestive_tract	head_and_neck	0.863838	0.918228	0.130932	1.343868	2.724072	4.00193	5.445663	0.899894	0.0169	1049261	2.050027	2.724072	3.922618
HT	907063	haematopoietic_and_lymphoid_tissue	lymphoid_neoplasm	blood	B_cell_lymphoma	0.844737	1.048759	0.055183	5.41405	6.94712	7.948757	9.296726	0.996557	0.0008	1267187	4.228603	6.94712	11.201176
HT-1080	907064	soft_tissue	fibrosarcoma	soft_tissue	fibrosarcoma	0.820349	0.640662	0.115823	0.322078	2.271379	4.24065	6.397423	0.825766	0.0051	1049262	1.654507	2.271379	3.194304
HT-1197	907065	urinary_tract	carcinoma	urogenital_system	Bladder	0.850289	0.819217	0.104315	2.537068	4.263356	5.642471	7.305167	0.95326	0.0025	1049263	2.963884	4.263356	6.812672
HT-1376	907066	urinary_tract	carcinoma	urogenital_system	Bladder	0.845758	0.745343	0.076932	0.941891	2.600858	4.260263	6.069213	0.8589	0.0025	991240	2.070225	2.600858	3.448891
HT-144	907067	skin	malignant_melanoma	skin	melanoma	0.831691	0.530792	0.111905	0.013238	2.380521	4.72223	7.31056	0.82283	0.0081	1035716	1.688422	2.380521	3.591284
HT-3	907068	cervix	carcinoma	urogenital_system	cervix	0.869187	1.141383	0.100386	1.695124	2.803479	3.797385	4.933147	0.919029	0.0092	1101440	2.294103	2.803479	3.95008
HuCCT1	907069	biliary_tract	carcinoma	digestive_system	biliary_tract	0.8845	0.866836	0.069693	1.645467	3.089318	4.443028	5.949443	0.911441	0.0098	1035789	2.512558	3.089318	4.177025
HuH-7	907071	liver	carcinoma	digestive_system	liver	0.733818	0.819195	0.078031	-0.101012	1.428277	3.124716	4.942784	0.780951	0.004	1101444	1.075051	1.428277	1.771453
HuO9	907072	bone	osteosarcoma	bone	osteosarcoma	0.959301	1.058029	0.109738	-0.248178	0.834031	1.936758	3.05322	0.749695	0.0065	1049267	0.498632	0.834031	1.162797
HUTU-80	907073	small_intestine	carcinoma	digestive_system	digestive_system_other	0.943999	1.086912	0.066944	0.583975	1.640275	2.709647	3.811378	0.821383	0.0019	991188	1.470581	1.640275	1.824762
IGR-1	907169	skin	malignant_melanoma	skin	melanoma	0.887196	0.768522	0.130426	-1.028758	0.547087	2.129371	3.782552	0.685243	0.0035	1035790	0.060589	0.547087	1.070187
IMR-5	907170	autonomic_ganglia	neuroblastoma	nervous_system	neuroblastoma	0.878758	1.195544	0.09152	2.183245	4.000515	4.348285	5.524669	0.957646	0.0093	1309269	2.392022	4.000515	8.825898
IPC-298	907171	skin	malignant_melanoma	skin	melanoma	0.95577	1.103301	0.118522	0.527136	1.574065	2.612045	3.686823	0.817714	0.0029	1035792	1.259538	1.574065	1.943914
IST-MEL1	907172	skin	malignant_melanoma	skin	melanoma	0.89813	0.993152	0.072685	1.853906	3.144928	4.268444	5.552861	0.922875	0.0087	1035717	2.534621	3.144928	4.485237
IST-MES1	907173	pleura	mesothelioma	lung	mesothelioma	0.953564	1.274823	0.060935	1.350014	2.255909	3.154067	4.089432	0.894872	0.0014	1035718	2.074587	2.255909	2.520389
JAR	907175	placenta	choriocarcinoma	urogenital_system	urogenital_system_other	0.962537	0.932828	0.081478	0.3128	1.534974	2.759477	4.020184	0.796326	0.0066	1035794	1.285312	1.534974	1.79905
JEG-3	907176	placenta	choriocarcinoma	urogenital_system	urogenital_system_other	0.87385	1.01573	0.114482	1.74479	3.069738	4.149096	5.44583	0.917944	0.0052	1049178	2.308747	3.069738	4.805034
JiyoyeP-2003	907268	haematopoietic_and_lymphoid_tissue	lymphoid_neoplasm	blood	Burkitt_lymphoma	0.844002	1.005263	0.088039	3.089139	4.768544	5.722899	7.127703	0.974894	0.0052	1324519	3.038888	4.768544	8.893466
JVM-2	907269	haematopoietic_and_lymphoid_tissue	lymphoid_neoplasm	blood	lymphoid_neoplasm_other	0.858565	0.93284	0.069635	2.092833	3.49093	4.737526	6.154103	0.935226	0.0021	1258647	2.713598	3.49093	4.955027
JVM-3	907270	haematopoietic_and_lymphoid_tissue	lymphoid_neoplasm	blood	lymphoid_neoplasm_other	0.96324	0.972084	0.109602	0.384091	1.583701	2.753413	3.976381	0.805803	0.005	1257191	1.271366	1.583701	2.025725
KALS-1	907271	central_nervous_system	glioma	nervous_system	glioma	0.839467	0.743181	0.112596	1.51599	3.313997	4.890609	6.727204	0.907913	0.0164	1035721	2.32731	3.313997	5.204911
KARPAS-45	907272	haematopoietic_and_lymphoid_tissue	haematopoietic_neoplasm	blood	lymphoblastic_leukemia	1.25026	1.737426	0.08538	0.498151	1.116697	1.693199	2.21859	0.789479	0.0026	1265983	0.93331	1.116697	1.292408
KARPAS-299	907273	haematopoietic_and_lymphoid_tissue	lymphoid_neoplasm	blood	anaplastic_large_cell_lymphoma	0.866873	0.895228	0.097392	1.601425	3.019062	4.331878	5.814606	0.913214	0.0027	1254755	2.372237	3.019062	4.211765
KARPAS-422	907274	haematopoietic_and_lymphoid_tissue	lymphoid_neoplasm	blood	B_cell_lymphoma	0.841928	1.083391	0.069704	5.799481	7.231645	8.256396	9.570407	0.997962	0.0026	1267299	4.450596	7.231645	11.383261
KASUMI-1	907275	haematopoietic_and_lymphoid_tissue	haematopoietic_neoplasm	blood	acute_myeloid_leukaemia	1.141527	1.398899	0.099503	-0.490012	0.289844	1.060363	1.810881	0.684862	0.0009	1266067	0.064228	0.289844	0.507468
KE-37	907277	haematopoietic_and_lymphoid_tissue	haematopoietic_neoplasm	blood	lymphoblastic_leukemia	1.200896	1.514845	0.099833	0.459239	1.348026	1.586748	2.13491	0.776071	0.0052	1261615	0.810502	1.348026	2.831994
KG-1	907278	haematopoietic_and_lymphoid_tissue	haematopoietic_neoplasm	blood	acute_myeloid_leukaemia	0.91966	1.233186	0.068696	1.778737	3.587874	3.836909	4.940666	0.935438	0.0034	1308989	1.942274	3.587874	9.131652
KINGS-1	907279	central_nervous_system	glioma	nervous_system	glioma	0.825441	0.744565	0.119321	1.363785	3.109758	4.76361	6.632167	0.893472	0.0071	1049179	2.284271	3.109758	4.522425
KMOE-2	907280	haematopoietic_and_lymphoid_tissue	haematopoietic_neoplasm	blood	acute_myeloid_leukaemia	1.417053	1.667664	0.094847	0.002421	0.625386	1.219167	1.768851	0.705673	0.002	1254867	0.439483	0.625386	0.7923
KNS-42	907282	central_nervous_system	glioma	nervous_system	glioma	0.81243	0.663065	0.08013	1.966974	3.963408	5.797193	7.902298	0.918277	0.0031	1035722	2.901315	3.963408	5.877014
HuP-T3	907285	pancreas	carcinoma	pancreas	pancreas	0.822101	0.642095	0.078646	-0.32593	1.56311	3.524603	5.611062	0.785807	0.004	991243	1.222454	1.56311	1.946189
HuP-T4	907286	pancreas	carcinoma	pancreas	pancreas	0.851801	0.973481	0.080622	4.615085	6.297278	7.385395	8.831444	0.992348	0.0156	1101445	3.892964	6.297278	10.378239
HT55	907287	large_intestine	carcinoma	digestive_system	large_intestine	0.878952	1.051314	0.096259	1.374162	2.529389	3.667471	4.890261	0.896047	0.0083	1101441	2.132237	2.529389	3.162611
H-EMC-SS	907290	bone	chondrosarcoma	bone	chondrosarcoma	0.856453	0.873871	0.108415	-0.783813	0.573948	1.991605	3.468097	0.693647	0.0023	1049249	0.261254	0.573948	0.891871
GP5d	907291	large_intestine	carcinoma	digestive_system	large_intestine	0.858925	0.848783	0.065775	1.929576	3.410294	4.826248	6.387051	0.927437	0.0011	991223	2.757454	3.410294	4.555201
DMS-53	907295	lung	carcinoma	lung	lung_small_cell_carcinoma	0.875891	0.883094	0.154528	1.316644	2.775458	4.06225	5.54644	0.893389	0.0221	1049223	2.00151	2.775458	4.384049
G-402	907298	soft_tissue	leiomyoblastoma	soft_tissue	soft_tissue_other	0.885087	1.153909	0.069234	0.328681	1.335958	2.383608	3.46999	0.791383	0.0019	1035769	1.16803	1.335958	1.509887
G-401	907299	kidney	rhabdoid_tumour	kidney	kidney	1.001014	1.142536	0.087609	0.675173	1.674217	2.650485	3.658667	0.82981	0.0073	1049229	1.457323	1.674217	1.9436
KY821	907300	haematopoietic_and_lymphoid_tissue	haematopoietic_neoplasm	blood	leukemia	0.804309	0.92461	0.082032	-0.586409	0.711069	2.093538	3.556248	0.719293	0.0026	1265423	0.457262	0.711069	0.953719
KU812	907311	haematopoietic_and_lymphoid_tissue	haematopoietic_neoplasm	blood	chronic_myeloid_leukaemia	0.95792	1.26999	0.101433	0.987595	1.907052	2.795863	3.739148	0.866988	0.0083	1265871	1.638437	1.907052	2.305925
KU-19-19	907312	urinary_tract	carcinoma	urogenital_system	Bladder	1.089795	1.338303	0.07669	0.714845	1.550377	2.305448	3.085807	0.821603	0.0054	991256	1.350027	1.550377	1.906179
KS-1	907313	central_nervous_system	glioma	nervous_system	glioma	0.916946	0.699999	0.118801	-0.811849	0.870003	2.579375	4.354791	0.743346	0.0093	1035723	0.360992	0.870003	1.354671
KP-N-YN	907314	autonomic_ganglia	neuroblastoma	nervous_system	neuroblastoma	0.806676	0.625181	0.175793	-0.229215	1.818115	3.825922	6.06424	0.801953	0.0409	999950	0.857165	1.818115	3.307443
KYSE-150	907317	oesophagus	carcinoma	aero_digestive_tract	oesophagus	0.911062	1.061297	0.099529	1.454563	2.60343	3.681136	4.865669	0.90346	0.002	1049269	2.157793	2.60343	3.457463
KYSE-180	907318	oesophagus	carcinoma	aero_digestive_tract	oesophagus	0.853913	1.108627	0.089066	4.462882	5.804486	6.831923	8.111223	0.992642	0.0274	999962	2.724108	5.804486	10.748916
KYSE-270	907319	oesophagus	carcinoma	aero_digestive_tract	oesophagus	0.933936	1.140995	0.100161	1.24417	2.312668	3.280826	4.354556	0.883325	0.0043	999963	1.949769	2.312668	3.104033
KYSE-450	907320	oesophagus	carcinoma	aero_digestive_tract	oesophagus	0.849821	0.820843	0.07947	1.019792	2.518796	4.014344	5.644629	0.863607	0.01	991259	2.123268	2.518796	3.179186
KYSE-510	907321	oesophagus	carcinoma	aero_digestive_tract	oesophagus	0.892062	0.982646	0.07183	1.183765	2.462671	3.637776	4.944945	0.880537	0.001	1035806	1.888656	2.462671	3.532379
L-428	907322	haematopoietic_and_lymphoid_tissue	lymphoid_neoplasm	blood	Hodgkin_lymphoma	0.852812	1.027062	0.083277	5.054291	6.648337	7.662651	9.045689	0.995314	0.0014	1262063	4.038009	6.648337	10.996716
L-540	907323	haematopoietic_and_lymphoid_tissue	lymphoid_neoplasm	blood	Hodgkin_lymphoma	0.911821	1.235158	0.111497	1.836856	3.571955	3.811506	4.892036	0.937112	0.0088	1255427	2.079582	3.571955	8.062988
J-RT3-T3-5	907391	haematopoietic_and_lymphoid_tissue	lymphoid_neoplasm	blood	lymphoblastic_T_cell_leukaemia	1.270495	1.713978	0.113	0.011736	0.627613	1.225642	1.791554	0.707824	0.005	1254643	0.401675	0.627613	0.827889
LAMA-84	907783	haematopoietic_and_lymphoid_tissue	haematopoietic_neoplasm	blood	chronic_myeloid_leukaemia	0.955761	1.066537	0.102904	1.131781	2.394676	3.13576	4.226472	0.878995	0.0101	1254979	1.782521	2.394676	4.549743
LC-2-ad	907786	lung	carcinoma	lung	lung_NSCLC_adenocarcinoma	0.837082	0.991445	0.088238	-0.103233	1.095952	2.365208	3.706114	0.748292	0.0024	1035730	0.861573	1.095952	1.344571
LC4-1	907787	haematopoietic_and_lymphoid_tissue	haematopoietic_neoplasm	blood	lymphoblastic_leukemia	0.868343	1.068089	0.083394	4.221471	5.636043	6.712245	8.012632	0.989286	0.0037	1266963	2.480389	5.636043	10.172591
LNCaP-Clone-FGC	907788	prostate	carcinoma	urogenital_system	prostate	0.87833	0.877758	0.064818	1.029585	2.386514	3.754997	5.201886	0.866696	0.0037	999965	2.098179	2.386514	2.822783
LOUCY	907789	haematopoietic_and_lymphoid_tissue	lymphoid_neoplasm	blood	lymphoblastic_T_cell_leukaemia	0.922267	0.953986	0.078826	0.859603	2.09782	3.324767	4.618706	0.871625	0.0028	1267523	1.773057	2.09782	2.55438
LoVo	907790	large_intestine	carcinoma	digestive_system	large_intestine	0.867964	0.973755	0.149676	0.300035	1.544173	2.781781	4.121027	0.804332	0.0128	1049278	0.800445	1.544173	2.4433
LP-1	907791	haematopoietic_and_lymphoid_tissue	lymphoid_neoplasm	blood	Myeloma	0.870121	0.999536	0.105478	3.885715	5.54493	6.583908	8.004044	0.986925	0.0131	1324071	2.93063	5.54493	10.041335
LS-123	907792	large_intestine	carcinoma	digestive_system	large_intestine	0.849513	0.994372	0.111723	2.737834	4.360567	5.386922	6.810042	0.96307	0.0141	991262	2.827429	4.360567	7.796547
LS-411N	907794	large_intestine	carcinoma	digestive_system	large_intestine	0.837787	0.725255	0.115532	0.512614	1.915947	3.198204	5.229083	0.834488	0.0404	1035732	-0.206261	1.915947	5.232792
LS-513	907795	large_intestine	carcinoma	digestive_system	large_intestine	0.754306	0.775707	0.073447	-0.360949	1.229678	2.95201	4.815866	0.754258	0.0006	991194	0.979993	1.229678	1.481217
LU-99A	907796	lung	carcinoma	lung	lung_NSCLC_large_cell	0.953291	0.98971	0.097581	0.570744	1.745878	2.890799	4.095066	0.822951	0.0031	991264	1.455685	1.745878	2.199683
MC116	907799	haematopoietic_and_lymphoid_tissue	lymphoid_neoplasm	blood	B_cell_lymphoma	0.83195	1.052114	0.084667	5.423224	6.900898	7.972052	9.340332	0.996426	0.0185	1255651	3.808055	6.900898	11.346419
MC-IXC	908118	autonomic_ganglia	neuroblastoma	nervous_system	neuroblastoma	1.099757	1.225661	0.160133	-0.047342	0.883748	1.743603	2.628891	0.733234	0.0022	1049281	0.497975	0.883748	1.314007
MDA-MB-175-VII	908120	breast	carcinoma	breast	breast	0.928458	1.077712	0.12949	1.267944	2.537213	3.374615	4.534414	0.883381	0.0061	1101460	1.843493	2.537213	4.729937
MDA-MB-361	908121	breast	carcinoma	breast	breast	0.796623	0.670238	0.110472	2.407161	4.65146	6.49559	8.698601	0.932731	0.0113	1035814	3.020507	4.65146	7.791468
MDA-MB-453	908122	breast	carcinoma	breast	breast	0.885751	0.959474	0.15997	1.331947	2.678907	3.85563	5.215316	0.894583	0.0244	1035815	1.972038	2.678907	4.222116
MDA-MB-468	908123	breast	carcinoma	breast	breast	0.938324	1.011104	0.106916	0.537226	1.686206	2.834146	4.031392	0.820799	0.0055	1101542	1.396286	1.686206	2.038525
MEL-HO	908124	skin	malignant_melanoma	skin	melanoma	0.845288	0.845015	0.087247	2.324466	4.153582	5.44493	7.095455	0.946463	0.0038	1035817	2.508681	4.153582	7.597587
MEL-JUSO	908125	skin	malignant_melanoma	skin	melanoma	0.798239	0.600676	0.127412	0.188719	2.299729	4.424598	6.787522	0.819693	0.0029	1049282	1.656478	2.299729	3.307714
MEG-01	908126	haematopoietic_and_lymphoid_tissue	haematopoietic_neoplasm	blood	chronic_myeloid_leukaemia	0.897935	1.159926	0.071139	3.004456	4.485874	5.411057	6.635003	0.972215	0.0052	1257667	2.22945	4.485874	8.909029
MES-SA	908127	soft_tissue	sarcoma	urogenital_system	uterus	0.917435	1.191583	0.08592	2.005727	3.79425	4.117172	5.248145	0.945217	0.0059	1233296	2.264695	3.79425	8.491735
Mewo	908128	skin	malignant_melanoma	skin	melanoma	0.808055	0.448169	0.126056	-1.864032	0.913045	3.803878	6.923935	0.760373	0.0067	1035819	0.186516	0.913045	1.713262
MFE-280	908129	endometrium	carcinoma	urogenital_system	endometrium	0.866283	1.086213	0.102939	2.7328	4.364404	5.080627	6.349483	0.9698	0.0324	991269	2.752035	4.364404	8.765575
MFE-296	908130	endometrium	carcinoma	urogenital_system	endometrium	0.876448	0.924089	0.093094	1.681849	3.125814	4.32217	5.750614	0.918733	0.0071	1311543	2.397951	3.125814	4.937083
MG-63	908131	bone	osteosarcoma	bone	osteosarcoma	0.890229	0.926055	0.12403	0.666987	2.056975	3.195184	4.575967	0.853403	0.0113	1049284	1.19586	2.056975	3.944572
MHH-CALL-2	908132	haematopoietic_and_lymphoid_tissue	lymphoid_neoplasm	blood	B_cell_leukemia	0.840181	1.007535	0.138299	4.263608	5.929975	6.948505	8.366742	0.989378	0.0237	1323819	3.355579	5.929975	10.486712
MHH-ES-1	908134	bone	Ewings_sarcoma-peripheral_primitive_neuroectodermal_tumour	bone	ewings_sarcoma	0.848432	0.696167	0.11854	-0.721283	1.010811	2.787599	4.674976	0.745056	0.0037	1049285	0.541152	1.010811	1.494967
MHH-NB-11	908135	autonomic_ganglia	neuroblastoma	nervous_system	neuroblastoma	1.076858	1.123045	0.122106	-0.153968	0.841702	1.839555	2.863767	0.720699	0.0231	991272	0.398712	0.841702	1.247554

MHH-PREB-1	908136	haematopoietic_and_lymphoid_tissue	lymphoid_neoplasm	blood	B_cell_leukemia	0.978395	1.44879	0.09718	1.264534	2.052927	2.830906	3.642622	0.892997	0.012	1307757	1.78312	2.052927	2.463337
MKN1	908138	stomach	carcinoma	digestive_system	stomach	0.878562	1.122599	0.105908	2.136052	3.81731	4.431091	5.687134	0.951538	0.008	1049286	2.405094	3.81731	8.375085
MKN28	908139	stomach	carcinoma	digestive_system	stomach	0.857897	1.094088	0.15299	3.017846	4.833398	5.559649	6.905363	0.973732	0.0451	1049287	2.477493	4.833398	10.264764
ML-2	908141	haematopoietic_and_lymphoid_tissue	haematopoietic_neoplasm	blood	acute_myeloid_leukaemia	0.883289	1.104785	0.087241	1.246691	2.330271	3.407681	4.563447	0.8921	0.0037	1323203	1.992573	2.330271	2.827836
MLMA	908142	haematopoietic_and_lymphoid_tissue	lymphoid_neoplasm	blood	hairy_cell_leukaemia	0.988654	1.190748	0.100083	1.165926	2.293816	2.884945	3.842917	0.873832	0.0041	991275	1.698526	2.293816	4.177745
MN-60	908143	haematopoietic_and_lymphoid_tissue	lymphoid_neoplasm	blood	B_cell_leukemia	0.840293	1.077981	0.093614	5.60498	7.088201	8.071389	9.386094	0.997609	0.0132	1309605	4.201247	7.088201	11.695787
MOLT-13	908146	haematopoietic_and_lymphoid_tissue	lymphoid_neoplasm	blood	lymphoblastic_T_cell_leukaemia	1.292795	1.969681	0.070128	0.698939	1.236335	1.75421	2.241896	0.811072	0.0031	1264667	1.114879	1.236335	1.351657
MOLT-16	908147	haematopoietic_and_lymphoid_tissue	lymphoid_neoplasm	blood	lymphoblastic_T_cell_leukaemia	1.104931	1.365869	0.156868	0.094282	0.912202	1.702635	2.481517	0.778885	0.0043	1267607	0.515467	0.912202	1.304276
MONO-MAC-6	908148	haematopoietic_and_lymphoid_tissue	haematopoietic_neoplasm	blood	acute_myeloid_leukaemia	1.16601	1.193697	0.077011	-0.922169	-0.013466	0.881274	1.749446	0.629651	0.001	1262287	-0.207031	-0.013466	0.1732
Mo-T	908149	haematopoietic_and_lymphoid_tissue	lymphoid_neoplasm	blood	hairy_cell_leukaemia	0.917999	1.229055	0.115505	1.199434	2.195855	3.098894	4.110511	0.885109	0.0094	1255091	1.801806	2.195855	3.168338
MPP-89	908150	pleura	mesothelioma	lung	mesothelioma	0.869127	0.980966	0.079275	1.134301	2.37083	3.580544	4.891425	0.895897	0.0048	1035733	1.982179	2.37083	3.013764
MRK-nu-1	908151	breast	carcinoma	breast	breast	0.853037	1.077084	0.089466	5.683136	7.1298	8.138805	9.438876	0.997974	0.0101	1265535	4.243249	7.1298	11.382162
MSTO-211H	908152	pleura	mesothelioma	lung	mesothelioma	0.846461	1.038935	0.127501	4.727267	6.26427	7.290288	8.653289	0.99344	0.0303	999966	3.630265	6.26427	10.536356
MV-4-11	908156	haematopoietic_and_lymphoid_tissue	haematopoietic_neoplasm	blood	leukemia	1.132186	1.064523	0.110959	-2.013346	-0.989105	0.022386	1.013232	0.487759	0.0018	1255203	-1.265802	-0.989105	-0.731039
NALM-6	908158		lymphoid neoplasm	blood	B_cell_leukemia	1.09316	1.564439	0.101721	0.904686	2.225124	2.53393	3.317457	0.862202	0.0071	1323343	0.976644	2.225124	5.870291
NB69	908440	autonomic_ganglia	neuroblastoma	nervous_system	neuroblastoma	1.158862	1.276694	0.1162	0.068501	0.919771	1.747762	2.541602	0.741764	0.0061	991277	0.657076	0.919771	1.166236
NCI-H520	908443	lung	carcinoma	lung	lung_NSCLC_squamous_cell_carcinoma	0.878186	1.18055	0.063348	2.548062	4.090446	4.697091	5.840861	0.964662	0.0019	1035838	2.80376	4.090446	8.177453
NCI-SNU-1	908444	stomach	carcinoma	digestive_system	stomach	0.843271	1.007127	0.101986	3.731081	5.359694	6.387194	7.793041	0.984998	0.007	1323371	3.281667	5.359694	9.20989
NCI-SNU-5	908445	stomach	carcinoma	digestive_system	stomach	0.838081	0.925021	0.099585	4.151677	5.9295	7.126422	8.674711	0.986889	0.001	1255315	3.63856	5.9295	9.978994
NCI-SNU-16	908446	stomach	carcinoma	digestive_system	stomach	0.826661	0.94129	0.114397	4.005182	5.78923	6.977056	8.517276	0.986721	0.0015	1323063	3.4505	5.78923	10.110828
NH-12	908447	autonomic_ganglia	neuroblastoma	nervous_system	neuroblastoma	0.872694	0.885292	0.101746	0.34917	1.735156	3.103994	4.582791	0.810917	0.0023	1049315	1.268806	1.735156	2.439933
NKM-1	908448	haematopoietic_and_lymphoid_tissue	haematopoietic_neoplasm	blood	acute_myeloid_leukaemia	0.974036	1.094014	0.106188	0.57957	1.711813	2.774579	3.911719	0.82554	0.0061	1256659	1.246306	1.711813	2.583877
NMC-G1	908449	central_nervous_system	glioma	nervous_system	glioma	0.854309	0.984629	0.137664	2.677442	4.310955	5.313537	6.720775	0.962379	0.0187	1049195	2.71631	4.310955	7.883642
no-11	908450	central_nervous_system	glioma	nervous_system	glioma	0.856947	0.962436	0.135638	2.680468	4.326199	5.387259	6.821214	0.962232	0.0306	1049198	2.726511	4.326199	7.753349
NOMO-1	908451	haematopoietic_and_lymphoid_tissue	haematopoietic_neoplasm	blood	acute_myeloid_leukaemia	1.060193	0.847897	0.114353	-0.930352	0.387503	1.697432	2.986991	0.68506	0.0052	1256547	-0.023557	0.387503	0.760115
no-10	908452	central_nervous_system	glioma	nervous_system	glioma	0.872289	0.990093	0.113262	1.885848	3.265008	4.377084	5.710713	0.931648	0.0081	1049197	2.400839	3.265008	5.057099
NTERA-S-cl-D1	908454	testis	germ_cell_tumour	urogenital_system	testis	1.053511	0.939874	0.077652	-0.82564	0.385194	1.471879	2.489345	0.659857	0.0051	1035740	0.026381	0.385194	0.726423
NUGC-3	908455	stomach	carcinoma	digestive_system	stomach	0.834004	1.063643	0.140246	4.588555	6.122918	7.124649	8.48264	0.99357	0.1054	1049316	3.278796	6.122918	10.548379
NCI-H747	908457	large_intestine	carcinoma	digestive_system	large_intestine	0.853721	0.749639	0.081749	1.288808	2.952542	4.562552	6.330568	0.885849	0.0045	991198	2.345999	2.952542	4.071277
NCI-H716	908458	large_intestine	carcinoma	digestive_system	large_intestine	0.87975	0.488238	0.120704	-2.32849	0.140226	2.588378	5.192058	0.69644	0.0051	1256855	-0.448656	0.140226	0.758417
NCI-H596	908459	lung	carcinoma	lung	lung_NSCLC_adenocarcinoma	0.845566	1.001599	0.086188	4.348683	5.971641	7.0153	8.411461	0.989779	0.0044	1049306	3.637681	5.971641	10.101333
NCI-H441	908460	lung	carcinoma	lung	lung_NSCLC_adenocarcinoma	0.826067	0.744293	0.063723	1.431887	3.092841	4.748843	6.557531	0.889775	0.0007	991289	2.567362	3.092841	3.92398
NCI-N87	908461	stomach	carcinoma	digestive_system	stomach	0.825962	0.776747	0.077161	3.086792	4.998859	6.492001	8.309454	0.966403	0.0034	1035839	3.334494	4.998859	8.011425
NCI-H2452	908462	pleura	mesothelioma	lung	mesothelioma	0.838732	0.786122	0.067036	1.779386	3.376862	4.929356	6.638564	0.916904	0.0005	999973	2.707803	3.376862	4.501264
NCI-H1793	908463	lung	carcinoma	lung	lung_NSCLC_adenocarcinoma	0.792842	0.589401	0.131316	1.736532	4.137886	6.293148	8.807533	0.903266	0.0226	999971	2.712618	4.137886	6.734387
NCI-H358	908465	lung	carcinoma	lung	lung_NSCLC_adenocarcinoma	0.855448	1.02994	0.109552	0.330713	1.489587	2.693568	3.961083	0.79605	0.0052	1035837	1.193119	1.489587	1.823909
NCI-H1155	908467	lung	carcinoma	lung	lung_NSCLC_large_cell	0.846299	1.078665	0.07346	4.589694	6.047287	7.091918	8.409048	0.993018	0.0061	1262623	2.548437	6.047287	10.455271
NCI-H28	908470	pleura	mesothelioma	lung	mesothelioma	0.9079	1.069008	0.121531	0.386572	1.487023	2.597687	3.759122	0.828893	0.0048	1049237	1.100801	1.487023	1.914719
NCI-H1581	908471	lung	carcinoma	lung	lung_NSCLC_large_cell	0.78181	0.459822	0.114981	-0.18047	2.673127	5.513241	8.704837	0.83603	0.0099	999968	1.761149	2.673127	4.306441
NCI-H1573	908472	lung	carcinoma	lung	lung_NSCLC_adenocarcinoma	0.876161	1.043369	0.072912	1.913193	3.112907	4.235183	5.479745	0.927682	0.0024	991280	2.586633	3.112907	4.089164
NCI-H1666	908473	lung	carcinoma	lung	lung_NSCLC_adenocarcinoma	0.886136	0.930736	0.060321	1.622935	2.945396	4.193162	5.568545	0.913591	0.0022	1035825	2.476624	2.945396	3.895141
NCI-H1703	908474	lung	carcinoma	lung	lung_NSCLC_adenocarcinoma	0.703851	0.400306	0.090781	1.25509	4.699557	8.125464	12.102391	0.891401	0.0029	1049295	3.047388	4.699557	7.481527
NCI-H1755	908475	lung	carcinoma	lung	lung_NSCLC_adenocarcinoma	0.952169	1.169141	0.135001	0.511427	1.711293	2.367716	3.374808	0.815337	0.0108	999970	0.905922	1.711293	5.132338
NCI-H1993	908476	lung	carcinoma	lung	lung_NSCLC_adenocarcinoma	0.838221	1.078217	0.075994	5.680289	7.182121	8.155401	9.476533	0.997748	0.0066	999972	4.37949	7.182121	11.727072
NCI-H630	908482	large_intestine	carcinoma	digestive_system	large_intestine	0.862721	1.302284	0.066799	1.191689	2.100496	3.039262	4.026371	0.88034	0.0078	999975	1.914511	2.100496	2.323421
NCI-H524	908483	lung	carcinoma	lung	lung_small_cell_carcinoma	1.173819	1.977481	0.106131	0.318971	0.861771	1.387866	1.912835	0.745649	0.0055	1263995	0.631073	0.861771	1.086649
ONS-76	909248	central_nervous_system	primitive_neuroectodermal_tumour-medulloblastoma	nervous_system	medulloblastoma	0.888442	1.028415	0.099972	1.617818	2.834259	3.930491	5.169304	0.910607	0.0057	1049201	2.293385	2.834259	3.95815
OPM-2	909249	haematopoietic_and_lymphoid_tissue	haematopoietic_neoplasm	blood	Myeloma	0.876585	1.065992	0.075363	3.201345	4.84885	5.657052	6.966176	0.978103	0.0065	1266739	2.614223	4.84885	9.348971
OS-RC-2	909250	kidney	carcinoma	kidney	kidney	0.867241	1.210546	0.04998	1.555579	2.538527	3.541364	4.600034	0.913304	0.0029	1035741	2.312158	2.538527	2.838315
P12-ICHIKAWA	909251	haematopoietic_and_lymphoid_tissue	lymphoid_neoplasm	blood	lymphoblastic_T_cell_leukaemia	0.864984	1.069134	0.092246	4.167935	5.634296	6.677135	7.994495	0.988999	0.004	1265759	1.82741	5.634296	10.333899
P30-OHK	909252	haematopoietic_and_lymphoid_tissue	lymphoid_neoplasm	blood	lymphoblastic_leukemia	1.070503	1.632009	0.087224	0.344139	1.036782	1.676463	2.347518	0.778562	0.0029	1307869	0.789009	1.036782	1.375331
P31-FUJ	909253	haematopoietic_and_lymphoid_tissue	haematopoietic_neoplasm	blood	acute_myeloid_leukaemia	0.898361	1.168243	0.086565	2.379487	4.02436	4.547657	5.708802	0.961728	0.0117	1261839	2.602072	4.02436	8.57029
PA-1	909255	ovary	germ_cell_tumour	urogenital_system	ovary	1.488307	1.747546	0.128118	-0.36878	0.12114	0.733245	1.190379	0.618604	0.0323	1035847	-0.474586	0.12114	0.426003
OCUB-M	909256	breast	carcinoma	breast	breast	0.911647	1.058011	0.080175	1.831442	3.044118	4.064118	5.250729	0.924979	0.0049	999952	2.459924	3.044118	4.419997
OC-314	909257	ovary	carcinoma	urogenital_system	ovary	0.915687	0.953026	0.058332	1.474292	2.732065	3.95699	5.264398	0.903452	0.0018	1049199	2.376581	2.732065	3.388231
PF-382	909260	haematopoietic_and_lymphoid_tissue	lymphoid_neoplasm	blood	lymphoblastic_T_cell_leukaemia	0.867525	1.04238	0.095781	2.91177	4.478377	5.419766	6.748323	0.97183	0.0142	1262175	2.878318	4.478377	7.902235
Raji	909262	haematopoietic_and_lymphoid_tissue	lymphoid_neoplasm	blood	Burkitt_lymphoma	0.844665	1.027343	0.097738	4.797084	6.389171	7.395414	8.774472	0.994001	0.005	1324323	3.789291	6.389171	10.665458
RCM-1	909263	large_intestine	carcinoma	digestive_system	large_intestine	0.829404	0.752804	0.118309	0.965446	2.632682	4.260402	6.056056	0.86221	0.0033	1049122	2.010053	2.632682	3.695205
RD	909264	soft_tissue	rhabdomyosarcoma	soft_tissue	rhabdomyosarcoma	0.881376	1.005354	0.097817	1.068363	2.262516	3.447022	4.720958	0.875632	0.0017	1049123	1.907	2.262516	2.809736
REH	909696	haematopoietic_and_lymphoid_tissue	lymphoid_neoplasm	blood	B_cell_leukemia	0.908797	0.900925	0.073881	-0.111043	1.226278	2.670244	4.131475	0.784553	0.0009	1261951	0.771876	1.226278	1.693697
RKO	909698	large_intestine	carcinoma	digestive_system	large_intestine	0.907022	1.16952	0.076342	1.558263	2.587049	3.57827	4.650981	0.910483	0.007	1049202	2.266289	2.587049	3.193135
RMG-I	909699	ovary	carcinoma	urogenital_system	ovary	0.815179	0.765478	0.182873	2.623949	4.811813	6.41123	8.369221	0.956079	0.0459	1101344	2.669742	4.811813	9.116949
RPMI-2650	909700	upper_aerodigestive_tract	carcinoma	aero_digestive_tract	head_and_neck	0.927457	1.014798	0.067762	0.871975	2.017431	3.176544	4.38001	0.855111	0.0023	999934	1.817829	2.017431	2.261442
RPMI-6666	909701	haematopoietic_and_lymphoid_tissue	lymphoid_neoplasm	blood	Hodgkin_lymphoma	0.843382	0.968387	0.100311	4.345629	6.059436	7.161118	8.637139	0.989458	0.0027	1323707	3.558847	6.059436	10.3796
RPMI-8402	909702	haematopoietic_and_lymphoid_tissue	lymphoid_neoplasm	blood	lymphoblastic_T_cell_leukaemia	0.861164	1.061405	0.08554	4.25954	5.7708	6.784352	8.103574	0.990065	0.0072	1306973	2.430378	5.7708	10.617507
RS4-11	909703	haematopoietic_and_lymphoid_tissue	haematopoietic_neoplasm	blood	leukemia	1.102329	1.082708	0.079517	-0.687153	0.323598	1.327351	2.314242	0.657698	0.0021	1263267	0.128787	0.323598	0.506367
RT-112	909704	urinary_tract	carcinoma	urogenital_system	Bladder	0.831027	0.856955	0.073342	-0.727217	0.655013	2.114587	3.65181	0.696699	0.0007	991146	0.44073	0.655013	0.868978
RVH-421	909706	skin	malignant_melanoma	skin	melanoma	0.897495	1.005197	0.081133	1.586076	2.817801	3.95632	5.217354	0.907398	0.01	991147	2.350625	2.817801	3.741072
Saos-2	909707	bone	osteosarcoma	bone	osteosarcoma	0.832792	0.968617	0.072332	1.260895	2.52347	3.810992	5.190171	0.892675	0.0043	991149	2.176989	2.52347	3.027228
SAS	909708	upper_aerodigestive_tract	carcinoma	aero_digestive_tract	head_and_neck	0.929239	1.109503	0.110496	1.681984	3.304323	3.941082	5.11104	0.924762	0.0123	1049128	1.939273	3.304323	7.038802
SCC-9	909709	upper_aerodigestive_tract	carcinoma	aero_digestive_tract	head_and_neck	0.819278	0.694224	0.106529	0.258726	2.038822	3.84668	5.816924	0.818655	0.0046	1049132	1.559051	2.038822	2.723931
SCH	909711	stomach	other	digestive_system	stomach	0.868975	0.84095	0.116694	1.319313	2.986708	4.245887	5.860523	0.89136	0.0142	1049133	2.058717	2.986708	5.633199
SF126	909712	central_nervous_system	glioma	nervous_system	glioma	0.856041	0.675336	0.094386	0.893608	2.811578	4.589987	6.600105	0.87344	0.01	1233276	2.061075	2.811578	4.452431
SH-4	909713	skin	malignant_melanoma	skin	melanoma	0.865351	0.828464	0.098239	0.904531	2.372355	3.816252	5.376229	0.855957	0.0047	1035746	1.954255	2.372355	3.059062
SJRH30	909716	soft_tissue	rhabdomyosarcoma	soft_tissue	rhabdomyosarcoma	1.091843	1.219154	0.078584	0.199602	1.104063	1.97886	2.839049	0.76678	0.0014	991150	0.915578	1.104063	1.291939
SJSA-1	909717	bone	other	bone	bone_other	0.848206	0.850437	0.094017	1.382093	2.86725	4.271632	5.83755	0.900732	0.0012	1035747	2.251748	2.86725	3.973808
SK-CO-1	909718	large_intestine	carcinoma	digestive_system	large_intestine	0.878483	1.089652	0.129326	0.623566	1.718643	2.817031	3.992466	0.849766	0.0164	1101540	1.300753	1.718643	2.261586
SK-HEP-1	909719	liver	carcinoma	digestive_system	liver	0.843227	0.943889	0.073108	3.18168	4.826499	5.944756	7.408633	0.972955	0.0015	1049136	3.2594	4.826499	8.202528
SK-LMS-1	909720	soft_tissue	sarcoma	soft_tissue	soft_tissue_other	0.923164	1.091283	0.134545	0.818949	1.909726	2.959497	4.081121	0.852251	0.0092	1233277	1.52086	1.909726	2.525951
SK-LU-1	909721	lung	carcinoma	lung	lung_NSCLC_adenocarcinoma	0.89282	1.060192	0.08715	1.322324	2.458364	3.561034	4.755722	0.89369	0.0028	1049137	2.08959	2.458364	3.07727
SKM-1	909722	haematopoietic_and_lymphoid_tissue	haematopoietic_neoplasm	blood	haematopoietic_neoplasm_other	1.047724	0.965447	0.161386	-1.94175	-0.788261	0.384363	1.544083	0.549588	0.002	1255763	-1.282939	-0.788261	-0.339587
SK-MEL-1	909723	skin	malignant_melanoma	skin	melanoma	0.861145	0.972892	0.108364	3.243142	4.986395	5.993231	7.442176	0.976391	0.005	1324575	3.025108	4.986395	9.246649
SK-MEL-3	909724	skin	malignant_melanoma	skin	melanoma	0.814051	0.616516	0.109678	0.118117	2.157665	4.227485	6.478547	0.81447	0.0069	1049139	1.61264	2.157665	2.985971
SK-MEL-24	909725	skin	malignant_melanoma	skin	melanoma	0.789012	0.555639	0.155049	-0.362382	1.940429	4.262887	6.820184	0.80754	0.0063	991200	1.081125	1.940429	3.188454
SK-MEL-30	909726	skin	malignant_melanoma	skin	melanoma	0.807295	0.457069	0.11384	-0.047656	2.811302	5.595372	8.700795	0.833447	0.0113	991153	1.88706	2.811302	4.643293
SK-MES-1	909728	lung	carcinoma	lung	lung_NSCLC_squamous_cell_carcinoma	0.833415	0.829254	0.086209	2.03437	3.586732	5.047752	6.693937	0.933773	0.0067	1049140	2.732583	3.586732	4.965165
SK-MG-1	909729	central_nervous_system	glioma	nervous_system	glioma	0.853886	0.869212	0.086778	1.150795	2.558822	3.952781	5.473363	0.886065	0.0026	1233279	2.1049	2.558822	3.285896
SK-NEP-1	909730	kidney	Wilms_tumour	kidney	kidney	1.015462	1.530158	0.122446	0.504584	1.236873	1.975277	2.713801	0.800231	0.009	1307197	0.993368	1.236873	1.468174
SK-PN-DW	909731	bone	Ewings_sarcoma-peripheral_primitive_neuroectodermal_tumour	bone	ewings_sarcoma	0.797195	0.686816	0.154952	1.968739	4.199192	5.998948	8.17373	0.9197	0.0208	1257303	2.605332	4.199192	7.54276
SK-UT-1	909732	soft_tissue	leiomyosarcoma	soft_tissue	soft_tissue_other	0.91856	1.102681	0.134107	1.194901	2.371259	3.316657	4.463062	0.882021	0.0101	1049208	1.79303	2.371259	4.259818
SNG-M	909735	endometrium	carcinoma	urogenital_system	endometrium	0.895706	1.032148	0.099062	1.509591	2.697195	3.821407	5.049493	0.901828	0.008	1311571	2.242631	2.697195	3.510157
SNU-387	909736	liver	carcinoma	digestive_system	liver	0.838088	0.76233	0.10652	1.552736	3.253562	4.833584	6.618475	0.908373	0.0067	1101361	2.372886	3.253562	4.824383
SNU-423	909737	liver	carcinoma	digestive_system	liver	0.8373	0.72789	0.100266	2.184663	4.126001	5.768999	7.683832	0.934083	0.0216	1101363	2.819108	4.126001	6.511272

SNU-449	909738	liver	carcinoma	digestive_system	liver	0.805041	0.527454	0.062931	1.324767	3.92054	6.428519	9.224657	0.885693	0.0031	1101364	2.061134	3.92054	6.405821
SNU-C2B	909740	large_intestine	carcinoma	digestive_system	large_intestine	0.772176	0.637317	0.101093	-0.496837	1.437745	3.489783	5.711254	0.776901	0.0046	991201	1.00929	1.437745	1.932735
SUP-T1	909743	haematopoietic_and_lymphoid_tissue	lymphoid_neoplasm	blood	lymphoblastic_T_cell_leukaemia	0.884609	1.126399	0.089374	2.436228	4.33295	4.830696	6.105249	0.961488	0.0085	1264891	2.4262	4.33295	9.181552
SW13	909744	adrenal_gland	adrenal_cortical_carcinoma	kidney	adrenal_gland	0.848661	0.663266	0.066934	-0.153385	1.638995	3.499624	5.460079	0.797658	0.0014	999938	1.347137	1.638995	1.952919
SW1088	909745	central_nervous_system	glioma	nervous_system	glioma	0.845363	0.67673	0.066081	0.069516	1.842277	3.669128	5.615703	0.80291	0.008	1035669	1.556863	1.842277	2.224272
SW1116	909746	large_intestine	carcinoma	digestive_system	large_intestine	0.862078	1.064153	0.112127	3.889439	5.552618	6.404122	7.738855	0.986642	0.0222	1049144	3.179486	5.552618	10.155905
SW1417	909747	large_intestine	carcinoma	digestive_system	large_intestine	0.826805	0.74405	0.079303	1.607522	3.307956	4.959012	6.791704	0.903768	0.0022	1035670	2.627462	3.307956	4.52306
SW1463	909748	large_intestine	carcinoma	digestive_system	large_intestine	0.858481	0.930515	0.070927	4.49468	6.232695	7.411358	8.924392	0.989417	0.0112	991161	3.938037	6.232695	10.256636
SW1710	909749	urinary_tract	carcinoma	urogenital_system	Bladder	0.857416	0.763443	0.104689	-0.375854	1.178998	2.798963	4.508006	0.758063	0.0183	1035674	0.819934	1.178998	1.54033
SW1783	909750	central_nervous_system	glioma	nervous_system	glioma	0.783322	0.562706	0.172	0.650849	3.109318	5.5491	8.265313	0.872078	0.059	1035673	0.836809	3.109318	6.223595
SW48	909751	large_intestine	carcinoma	digestive_system	large_intestine	0.99534	1.088367	0.11471	0.289729	1.348557	2.327636	3.36697	0.786443	0.0057	1049146	1.010292	1.348557	1.922419
SW626	909753	ovary	carcinoma	urogenital_system	ovary	0.845832	1.00518	0.100133	4.296015	5.882021	6.960755	8.370244	0.989035	0.0057	1101368	3.145568	5.882021	10.187834
SW684	909754	soft_tissue	fibrosarcoma	soft_tissue	fibrosarcoma	0.866715	0.840237	0.134328	1.619549	3.353167	4.695158	6.355537	0.919667	0.0302	1049209	2.149658	3.353167	6.140073
SW837	909755	large_intestine	carcinoma	digestive_system	large_intestine	0.837203	0.862042	0.097506	1.530388	3.004602	4.408479	5.974727	0.905759	0.0045	991167	2.382267	3.004602	4.115755
SW872	909756	soft_tissue	liposarcoma	soft_tissue	soft_tissue_other	0.902195	0.626264	0.096633	-0.714289	1.160331	3.046164	5.03533	0.747963	0.0147	1035750	0.759768	1.160331	1.622099
SW948	909757	large_intestine	carcinoma	digestive_system	large_intestine	0.84166	0.971461	0.060057	3.808053	5.386235	6.493208	7.925438	0.984731	0.0059	999939	3.612462	5.386235	8.669307
SW962	909758	vulva	carcinoma	urogenital_system	urogenital_system_other	0.772655	0.520938	0.087763	0.495077	2.984562	5.464452	8.249749	0.871871	0.005	1049211	1.954013	2.984562	4.606936
SW982	909759	soft_tissue	synovial_sarcoma	soft_tissue	soft_tissue_other	0.847723	0.597374	0.120617	-1.018011	0.910179	3.029174	5.237497	0.740821	0.0385	1035751	0.017517	0.910179	1.49542
T84	909761	large_intestine	carcinoma	digestive_system	large_intestine	0.850179	0.993814	0.098015	3.987842	5.629439	6.680327	8.09387	0.986316	0.0081	1049149	3.454048	5.629439	9.53804
TGBC1TKB	909769	biliary_tract	carcinoma	digestive_system	biliary_tract	0.830167	0.790177	0.11073	2.617368	4.534434	5.968014	7.745687	0.955517	0.0061	1035754	2.972526	4.534434	7.674829
TGBC11TKB	909770	stomach	carcinoma	digestive_system	stomach	0.841026	1.00328	0.092279	3.714255	5.401477	6.403582	7.817429	0.985475	0.0042	1324547	3.309801	5.401477	9.629812
THP-1	909771	haematopoietic_and_lymphoid_tissue	haematopoietic_neoplasm	blood	acute_myeloid_leukaemia	0.855611	1.048151	0.075609	4.57153	6.138696	7.128562	8.470464	0.992741	0.0076	1308821	3.672428	6.138696	10.455614
TUR	909773	haematopoietic_and_lymphoid_tissue	lymphoid_neoplasm	blood	B_cell_lymphoma	0.948183	1.420644	0.100896	0.83422	2.40641	2.118739	2.862843	0.841513	0.0088	1307533	1.055281	2.40641	7.268364
TYK-nu	909774	ovary	carcinoma	urogenital_system	ovary	0.937155	1.372233	0.060696	0.742838	1.57865	2.430917	3.303751	0.832545	0.0046	1101376	1.442202	1.57865	1.716086
U-2-OS	909776	bone	osteosarcoma	bone	osteosarcoma	0.920282	1.132632	0.083289	0.053673	1.123751	1.947581	2.934992	0.747853	0.0139	1035679	0.766259	1.123751	1.766433
U-698-M	909777	haematopoietic_and_lymphoid_tissue	lymphoid_neoplasm	blood	B_cell_leukemia	1.084424	1.602773	0.067966	1.184488	1.921726	2.424582	3.054016	0.878218	0.0012	1263071	1.662836	1.921726	2.673315
UACC-893	909778	breast	carcinoma	breast	breast	0.840687	0.668434	0.140414	0.769916	2.711435	4.510377	6.549026	0.857208	0.011	1035680	1.888164	2.711435	4.284946
UMC-11	909779	lung	carcinoid-endocrine_tumour	lung	lung_NSCLC_carcinoid	0.816176	0.650886	0.113943	0.564224	2.507296	4.439903	6.569974	0.844612	0.0071	991173	1.854712	2.507296	3.577679
VM-CUB-1	909780	urinary_tract	carcinoma	urogenital_system	Bladder	0.861784	0.868729	0.095004	2.030303	3.600637	4.907175	6.453541	0.933354	0.0026	1311599	2.656057	3.600637	5.658861
VMRC-RCZ	909781	kidney	carcinoma	kidney	kidney	0.837828	0.755823	0.132759	1.974025	3.810642	5.389721	7.224109	0.926569	0.0023	1049154	2.5866	3.810642	6.04982
WM-115	909784	skin	malignant_melanoma	skin	melanoma	0.882219	0.902347	0.106238	1.328568	2.741878	4.017867	5.467533	0.899589	0.0061	1049155	2.1197	2.741878	4.072861
WSU-NHL	909785	haematopoietic_and_lymphoid_tissue	lymphoid_neoplasm	blood	lymphoid_neoplasm_other	1.084906	1.720523	0.091204	0.80984	1.456934	2.085844	2.711274	0.8616	0.0069	1307645	1.235108	1.456934	1.705751
YAPC	909904	pancreas	carcinoma	pancreas	pancreas	0.874316	0.880043	0.06422	1.810612	3.225551	4.57062	6.042925	0.92314	0.0012	991179	2.641939	3.225551	4.237868
YH-13	909905	central_nervous_system	glioma	nervous_system	glioma	0.883925	0.730957	0.079662	0.22992	1.861124	3.455689	5.168268	0.809911	0.0038	1101401	1.46906	1.861124	2.553988
ZR-75-30	909907	breast	carcinoma	breast	breast	0.834477	0.892219	0.07774	3.646254	5.459294	6.671398	8.285016	0.978264	0.0028	991180	3.528457	5.459294	9.271688
RCC10RGB	909974	kidney	carcinoma	kidney	kidney	0.842119	0.92258	0.066614	1.699302	3.043187	4.359514	5.810718	0.920696	0.0014	1035742	2.541601	3.043187	3.894367
KURAMOCHI	909975	ovary	carcinoma	urogenital_system	ovary	0.892395	1.075888	0.103698	2.10412	3.420314	4.398931	5.629234	0.942673	0.0061	1049184	2.528167	3.420314	5.524113
KM-H2	909976	haematopoietic_and_lymphoid_tissue	lymphoid_neoplasm	blood	Hodgkin_lymphoma	0.85259	0.911397	0.126725	3.584755	5.415586	6.566202	8.11841	0.978966	0.0016	1323175	3.220507	5.415586	9.873142
HuO-3N1	909977	bone	osteosarcoma	bone	osteosarcoma	0.861577	0.860513	0.115561	1.229953	2.685253	4.075567	5.625946	0.886756	0.0033	1049266	2.085028	2.685253	3.747308
OE19	910079	oesophagus	carcinoma	aero_digestive_tract	oesophagus	0.832328	0.965953	0.121155	4.14547	5.883766	6.960815	8.437404	0.987351	0.0193	1035843	3.454452	5.883766	10.178342
NCI-H838	910399	lung	carcinoma	lung	lung_NSCLC_adenocarcinoma	0.872529	0.823939	0.145823	1.314497	2.889284	4.306333	5.93341	0.892859	0.0281	1101478	2.055275	2.889284	4.492295
Ramos-2G6-4C10	910401	haematopoietic_and_lymphoid_tissue	lymphoid_neoplasm	blood	Burkitt_lymphoma	1.026019	1.155819	0.1207	0.670645	1.751219	2.54803	3.520924	0.83383	0.0054	1256267	1.241629	1.751219	2.989381
RPMI-8866	910544	haematopoietic_and_lymphoid_tissue	haematopoietic_neoplasm	blood	chronic_myeloid_leukaemia	1.073457	1.219129	0.150047	-0.429546	0.483101	1.394542	2.287117	0.678546	0.0011	1256435	0.170587	0.483101	0.791878
QIMR-WIL	910545	haematopoietic_and_lymphoid_tissue	haematopoietic_neoplasm	blood	acute_myeloid_leukaemia	1.044634	1.426101	0.077294	1.061007	1.855894	2.589718	3.357592	0.865587	0.0021	1256771	1.664066	1.855894	2.326185
PSN1	910546	pancreas	carcinoma	pancreas	pancreas	0.956823	0.829331	0.079956	-0.115744	1.270165	2.642002	4.061916	0.760349	0.0017	1035662	1.011156	1.270165	1.598968
OAW-42	910548	ovary	carcinoma	urogenital_system	ovary	0.850099	0.978252	0.093017	1.170424	2.408499	3.652911	4.989232	0.881086	0.016	1101482	2.041186	2.408499	2.956938
OE33	910549	oesophagus	other	aero_digestive_tract	oesophagus	0.892472	1.240002	0.037188	2.339869	3.340337	4.274458	5.300183	0.958711	0.0012	991296	2.865884	3.340337	4.177342
CW-2	910554	large_intestine	carcinoma	digestive_system	large_intestine	0.852489	0.964484	0.139456	2.389676	4.003013	5.07279	6.515146	0.951848	0.0533	991184	2.453476	4.003013	7.481051
CMK	910566			blood	acute_myeloid_leukaemia	0.859969	0.99082	0.099468	3.64643	5.346449	6.354765	7.776479	0.983046	0.0031	1323231	3.241248	5.346449	9.525518
CHP-126	910567	autonomic_ganglia	neuroblastoma	nervous_system	neuroblastoma	0.93574	1.345054	0.089663	1.85124	3.537999	3.735637	4.639916	0.942543	0.0157	1325219	1.342387	3.537999	9.110893
CGTH-W-1	910568	thyroid	carcinoma	thyroid	thyroid	0.842325	0.613862	0.13037	-0.251319	1.80179	3.795723	6.014583	0.804558	0.0078	1035696	1.090834	1.80179	2.970743
COLO-320-HSR	910569	large_intestine	carcinoma	digestive_system	large_intestine	1.057004	1.658407	0.068308	0.790122	1.456978	2.124581	2.787494	0.830501	0.004	1258395	1.320158	1.456978	1.582897
ATN-1	910687	haematopoietic_and_lymphoid_tissue	lymphoid_neoplasm	blood	lymphoblastic_T_cell_leukaemia	1.034238	1.694535	0.086001	0.673232	1.331897	1.991934	2.651154	0.833218	0.0035	1322251	1.121662	1.331897	1.550719
CESS	910688	haematopoietic_and_lymphoid_tissue	haematopoietic_neoplasm	blood	acute_myeloid_leukaemia	0.838855	1.014145	0.108525	4.794234	6.398413	7.446641	8.845996	0.993792	0.0053	1323735	3.76668	6.398413	10.821196
COLO-678	910689	large_intestine	carcinoma	digestive_system	large_intestine	0.798601	0.67215	0.055923	1.604632	3.466859	5.330354	7.390835	0.903643	0.0011	991209	2.81139	3.466859	4.534808
COLO-684	910691	endometrium	carcinoma	urogenital_system	endometrium	0.952416	1.231181	0.100077	1.542092	2.626078	3.397155	4.387491	0.914058	0.0062	1257387	2.105716	2.626078	4.749547
COLO-668	910692	lung	carcinoma	lung	lung_small_cell_carcinoma	0.990713	1.759464	0.100004	-0.361341	0.273353	0.916806	1.565474	0.645114	0.0166	1035697	0.112348	0.273353	0.428775
LN-405	910694	central_nervous_system	glioma	nervous_system	glioma	0.856155	1.031335	0.160077	4.312397	5.921234	6.887707	8.247381	0.99139	0.0399	1049277	3.318099	5.921234	10.433993
TGBC24TKB	910695	biliary_tract	carcinoma	digestive_system	biliary_tract	0.880143	0.920138	0.10749	1.810441	3.512889	4.818265	6.335933	0.926071	0.0135	999954	1.92135	3.512889	6.587298
A388	910697	skin	carcinoma	skin	skin_other	0.77709	0.717675	0.077082	1.205558	2.999345	4.877652	6.884556	0.879876	0.0105	1035690	1.957312	2.999345	4.138624
BFTC-909	910698	kidney	carcinoma	kidney	kidney	0.833615	0.849351	0.076051	0.764171	2.186153	3.647864	5.204843	0.847611	0.0023	999967	1.865949	2.186153	2.625709
C2BBe1	910700	large_intestine	carcinoma	digestive_system	large_intestine	0.948323	1.037965	0.078132	1.10845	2.375785	3.117722	4.257522	0.872338	0.0046	991303	1.763927	2.375785	4.102676
SCC-25	910701	upper_aerodigestive_tract	carcinoma	aero_digestive_tract	head_and_neck	0.889574	0.964001	0.109565	1.093167	2.354441	3.5795	4.903609	0.879805	0.005	1035666	1.918545	2.354441	3.093074
AsPC-1	910702	pancreas	carcinoma	pancreas	pancreas	0.852658	0.9663	0.110498	1.995759	3.400505	4.603077	6.010462	0.929973	0.0046	1049271	2.512203	3.400505	5.086533
CA46	910703	haematopoietic_and_lymphoid_tissue	lymphoid_neoplasm	blood	Burkitt_lymphoma	0.850805	0.951937	0.093336	3.071174	4.760559	5.816809	7.295346	0.971166	0.0109	1324435	3.098894	4.760559	8.340906
AU565	910704	breast	carcinoma	breast	breast	0.852375	0.985411	0.118527	4.397962	6.05204	7.14687	8.582724	0.989985	0.0039	1049273	3.503106	6.05204	10.248555
BALL-1	910705	haematopoietic_and_lymphoid_tissue	lymphoid_neoplasm	blood	B_cell_leukemia	0.936394	1.347857	0.0724	1.645117	2.867683	3.192423	4.083444	0.921773	0.0026	1262735	2.088556	2.867683	5.410517
BL-41	910706	haematopoietic_and_lymphoid_tissue	lymphoid_neoplasm	blood	Burkitt_lymphoma	0.847046	1.091842	0.079298	4.936604	6.440888	7.394018	8.703974	0.996184	0.0109	1259263	3.434855	6.440888	11.284587
BV-173	910710	haematopoietic_and_lymphoid_tissue	haematopoietic_neoplasm	blood	chronic_myeloid_leukaemia	1.233938	1.461194	0.114216	0.169881	0.90088	1.592184	2.248503	0.746603	0.008	1256155	0.505152	0.90088	1.228071
IA-LM	910779	lung	carcinoma	lung	lung_NSCLC_large_cell	0.794516	0.625003	0.096191	0.930157	2.964846	5.000539	7.277763	0.869996	0.0029	1101392	2.241638	2.964846	4.168017
TGW	910780	autonomic_ganglia	neuroblastoma	nervous_system	neuroblastoma	1.504381	2.509917	0.057171	0.748731	1.190938	1.519459	1.861388	0.802738	0.007	1257471	1.05801	1.190938	1.766569
AN3-CA	910781	endometrium	carcinoma	urogenital_system	endometrium	0.827468	0.706908	0.061518	2.027299	3.863915	5.581255	7.524832	0.923355	0.0007	991250	2.950885	3.863915	5.501217
A204	910784	soft_tissue	rhabdomyosarcoma	soft_tissue	rhabdomyosarcoma	0.939111	0.658428	0.116619	-0.718738	1.065205	2.798699	4.625755	0.736457	0.0156	1035777	0.55086	1.065205	1.652058
NY	910849	bone	osteosarcoma	bone	osteosarcoma	0.799508	0.572579	0.08865	0.855206	3.081144	5.287987	7.758498	0.864602	0.0032	1049317	2.32554	3.081144	4.395075
C3A	910850	liver	carcinoma	digestive_system	liver	0.821277	0.780012	0.058772	2.114769	3.763721	5.329683	7.080038	0.932711	0.0026	991142	2.957907	3.763721	5.136996
A427	910851	lung	carcinoma	lung	lung_NSCLC_not_specified	0.963857	1.198746	0.045765	1.731132	2.94819	3.905767	5.001675	0.926293	0.0014	1101434	2.106968	2.94819	4.879125
CAL-85-1	910852	breast	carcinoma	breast	breast	0.954313	1.158029	0.084661	1.411943	2.47184	3.378676	4.41766	0.896186	0.0016	991176	2.107601	2.47184	3.673285
CHL-1	910853	skin	malignant_melanoma	skin	melanoma	0.831537	0.551815	0.10747	0.307297	2.941941	5.025857	7.643634	0.832944	0.0125	1035749	1.632063	2.941941	6.223205
RL	910861	haematopoietic_and_lymphoid_tissue	lymphoid_neoplasm	blood	B_cell_lymphoma	0.895602	1.111638	0.095442	2.358747	4.184866	4.729362	5.995966	0.960801	0.0054	1323035	2.511729	4.184866	8.68429
NCI-H1651	910900	lung	carcinoma	lung	lung_NSCLC_adenocarcinoma	0.83912	0.85307	0.122507	2.169981	3.868199	5.199175	6.829632	0.946344	0.0041	1049294	2.600679	3.868199	6.539108
RPMI-7951	910903	skin	malignant_melanoma	skin	melanoma	0.936967	0.872503	0.067257	0.689338	2.017146	3.347609	4.729128	0.839272	0.0052	991145	1.78549	2.017146	2.323435
SCC-4	910904	upper_aerodigestive_tract	carcinoma	aero_digestive_tract	head_and_neck	0.848292	0.927605	0.153356	3.481767	5.354256	6.490541	8.058688	0.976964	0.0392	999935	3.029296	5.354256	9.870135
SNU-C1	910905	large_intestine	carcinoma	digestive_system	large_intestine	0.849654	1.031781	0.118466	5.321907	6.882943	7.911824	9.271743	0.996207	0.0158	1323567	4.097673	6.882943	11.357197
ST486	910906	haematopoietic_and_lymphoid_tissue	lymphoid_neoplasm	blood	Burkitt_lymphoma	0.925041	1.242405	0.091742	1.966862	3.709138	3.97788	5.055421	0.945642	0.0149	1263183	2.221937	3.709138	8.185203
SW1990	910907	pancreas	carcinoma	pancreas	pancreas	0.863017	0.969062	0.092863	2.028906	3.355732	4.563387	5.922549	0.932796	0.0034	991162	2.637631	3.355732	4.633756
SCC-15	910911	upper_aerodigestive_tract	carcinoma	aero_digestive_tract	head_and_neck	0.862335	0.989966	0.11095	3.977616	5.730266	6.700687	8.129732	0.986664	0.0086	1049131	3.385977	5.730266	10.261055
Capan-2	910915	pancreas	carcinoma	pancreas	pancreas	0.849846	1.008642	0.087424	2.409009	3.773462	4.876835	6.209183	0.952984	0.0067	1049164	2.804511	3.773462	5.681649
CAL-27	910916	upper_aerodigestive_tract	carcinoma	aero_digestive_tract	head_and_neck	0.920837	1.235685	0.080149	2.174085	3.572039	4.261511	5.313254	0.950202	0.0103	1049142	2.156879	3.572039	7.218809
BC-1	910919	haematopoietic_and_lymphoid_tissue	lymphoid_neoplasm	blood	B_cell_lymphoma	0.850311	1.01429	0.11169	4.017956	5.647236	6.716902	8.132711	0.987037	0.007	1324463	2.414059	5.647236	10.262248
A704	910920	kidney	carcinoma	kidney	kidney	0.80653	0.63642	0.060017	-0.286626	1.650361	3.715162	5.924881	0.797988	0.0027	1101381	0.983027	1.650361	2.22442
A101D	910921	skin	malignant_melanoma	skin	melanoma	0.810862	0.56503	0.117244	-0.092819	2.137739	4.394259	6.860098	0.831983	0.0047	1035687	1.414264	2.137739	3.226336
769-P	910922	kidney	carcinoma	kidney	kidney	0.934501	1.167451	0.117355	1.146591	2.1975	3.115122	4.162566	0.874534	0.0117	991214	1.789558	2.1975	3.30774
23132-87	910924	stomach	carcinoma	digestive_system	stomach	0.837805	1.110868	0.12333	0.803385	1.899527	3.024145	4.23069	0.846979	0.0148	1233256	1.487641	1.899527	2.373446

A431	910925	skin	carcinoma	skin	skin_other	0.859865	0.738438	0.083471	1.283151	2.962928	4.609665	6.402576	0.885372	0.0055	1035786	2.377478	2.962928	3.975541
BFTC-905	910926	urinary_tract	carcinoma	urogenital_system	Bladder	1.191077	1.149084	0.063391	-0.07054	0.868186	1.77765	2.65262	0.724294	0.001	1035821	0.724775	0.868186	1.006575
CAL-51	910927	breast	carcinoma	breast	breast	0.887308	1.098165	0.095159	1.267135	2.359101	3.435217	4.588836	0.885814	0.0077	1035677	2.031544	2.359101	2.862454
RERF-LC-MS	910931	lung	carcinoma	lung	lung_NSCLC_adenocarcinoma	0.878933	1.011514	0.124546	1.53441	2.833533	3.904454	5.193413	0.911491	0.0088	1049124	2.094932	2.833533	4.584052
GAK	910932	skin	malignant_melanoma	skin	melanoma	0.869996	0.892775	0.130838	1.276859	2.744094	3.998205	5.476385	0.891594	0.017	1035711	2.010207	2.744094	4.478929
AM-38	910933	central_nervous_system	glioma	nervous_system	glioma	0.703014	0.327711	0.151462	-3.588517	0.430576	4.879774	9.862242	0.78111	0.0118	1035692	-0.76641	0.430576	1.692866
A4-Fuk	910934	haematopoietic_and_lymphoid_tissue	lymphoid_neoplasm	blood	B_cell_lymphoma	0.865146	0.931793	0.101106	-0.199214	1.07356	2.395642	3.773045	0.772252	0.0005	1308457	0.743447	1.07356	1.400707
A3-KAW	910935	stomach	carcinoma	blood	B_cell_lymphoma	0.857916	1.11345	0.086729	4.572521	5.963801	6.981177	8.250134	0.993094	0.0072	1309829	2.59943	5.963801	10.984829
DOK	910936	upper_aerodigestive_tract	other	aero_digestive_tract	head_and_neck	1.087909	0.897859	0.091684	-0.757424	0.479738	1.703344	2.906083	0.685183	0.006	991217	0.218534	0.479738	0.740894
COR-L279	910937			lung	lung_small_cell_carcinoma	0.879563	0.963596	0.077243	3.193704	4.848905	5.894597	7.308497	0.976543	0.0014	1324155	3.158853	4.848905	8.336944
NEC8	910942	testis	germ_cell_tumour	urogenital_system	testis	0.806882	0.842773	0.132363	-0.680048	0.747975	2.274562	3.890838	0.716947	0.0102	1101480	0.319701	0.747975	1.170402
CAS-1	910943	central_nervous_system	glioma	nervous_system	glioma	0.705611	0.422735	0.147453	-0.819333	2.430183	5.749345	9.56732	0.837643	0.0252	1049159	0.924024	2.430183	4.490016
ALL-PO	910944	haematopoietic_and_lymphoid_tissue	lymphoid_neoplasm	blood	lymphoblastic_leukemia	1.278589	1.260064	0.131795	-0.84932	-0.009866	0.815149	1.592528	0.616822	0.0132	1254083	-0.371722	-0.009866	0.293405
S-117	910946	soft_tissue	sarcoma	thyroid	thyroid	0.854689	1.0477	0.110833	1.951996	3.255564	4.316561	5.594831	0.937676	0.012	1233262	2.417033	3.255564	5.212355
OCI-AML2	910947	haematopoietic_and_lymphoid_tissue	haematopoietic_neoplasm	blood	acute_myeloid_leukaemia	0.958123	1.455481	0.080623	1.700604	2.857233	3.197645	4.006577	0.931256	0.0104	1322867	2.153926	2.857233	6.183244
MFM-223	910948	breast	carcinoma	breast	breast	0.924376	0.980123	0.050902	1.330157	2.530729	3.706784	4.958944	0.895378	0.0068	991271	2.274913	2.530729	2.973602
CAL-54	910952	kidney	carcinoma	kidney	kidney	0.861533	0.937359	0.119015	3.484497	5.274541	6.39448	7.910443	0.978722	0.0284	999942	3.175082	5.274541	9.278545
EFO-21	911905	ovary	carcinoma	urogenital_system	ovary	0.849835	0.703198	0.08501	1.966951	3.864101	5.549809	7.491478	0.917906	0.0035	1035762	2.782294	3.864101	5.925737
LS-1034	917486	large_intestine	carcinoma	digestive_system	large_intestine	0.905838	1.062794	0.128873	1.504348	2.736566	3.742052	4.948054	0.905585	0.004	991192	2.084629	2.736566	4.302866
22RV1	924100	prostate	carcinoma	urogenital_system	prostate	0.819755	0.703622	0.104175	1.512851	3.357634	5.108854	7.073613	0.897422	0.0075	1049309	2.49399	3.357634	4.920951
8505C	924102	thyroid	carcinoma	thyroid	thyroid	0.8559	0.798092	0.099924	1.742417	3.617058	4.989299	6.652593	0.913346	0.0227	1101410	1.732311	3.617058	7.133628
BCPAP	924104	thyroid	carcinoma	thyroid	thyroid	0.86894	0.646101	0.109953	-0.717566	1.162849	3.052969	5.072631	0.743187	0.0097	1035816	0.707958	1.162849	1.80447
BPH-1	924105	prostate	hyperplasia	urogenital_system	prostate	0.912165	0.74191	0.091698	-0.532661	1.036137	2.62714	4.271815	0.743891	0.0035	991291	0.698779	1.036137	1.376415
CAL-39	924107	vulva	carcinoma	urogenital_system	cervix	0.926661	1.079714	0.116847	1.334852	2.505116	3.486944	4.63551	0.893259	0.0061	1049150	1.980035	2.505116	3.928071
CaR-1	924108	large_intestine	carcinoma	digestive_system	large_intestine	0.857281	0.862913	0.148286	2.094431	3.779118	5.04838	6.630226	0.938794	0.006	1049165	2.495713	3.779118	6.608828
EFE-184	924109	endometrium	carcinoma	urogenital_system	endometrium	0.884294	0.95344	0.057773	1.800123	3.09863	4.321125	5.668987	0.91865	0.0021	1233280	2.592627	3.09863	4.038467
HDLM-2	924110	haematopoietic_and_lymphoid_tissue	lymphoid_neoplasm	blood	Hodgkin_lymphoma	0.912198	1.2877	0.063104	2.034239	3.549123	3.861479	4.862202	0.952498	0.0029	1257555	2.455133	3.549123	7.591423
HO-1-N-1	924111	upper_aerodigestive_tract	carcinoma	aero_digestive_tract	head_and_neck	0.962954	1.096195	0.056278	1.062827	2.151953	3.037734	4.088349	0.865136	0.001	991237	1.845705	2.151953	3.042546
HTC-C3	924151	thyroid	carcinoma	thyroid	thyroid	0.917435	1.018007	0.064574	1.173272	2.326319	3.463252	4.671404	0.883961	0.0026	991242	2.074216	2.326319	2.781405
KGN	924186	ovary	other	urogenital_system	ovary	1.002385	0.953186	0.129833	-0.33908	0.835374	2.02456	3.213446	0.736776	0.0334	999948	0.344155	0.835374	1.264877
KLE	924187	endometrium	carcinoma	urogenital_system	endometrium	0.837276	0.83374	0.117418	1.752874	3.361043	4.762287	6.457332	0.916992	0.0262	999949	2.253262	3.361043	5.595192
KNS-81-FD	924188	central_nervous_system	glioma	nervous_system	glioma	0.870604	1.027933	0.118697	1.502031	2.735004	3.856405	5.125364	0.904131	0.0056	1049183	2.163537	2.735004	3.894888
K5	924238	thyroid	carcinoma	thyroid	thyroid	0.841845	0.902322	0.15233	0.164542	1.507886	2.869879	4.33509	0.792784	0.0308	1035720	1.046921	1.507886	2.079431
L-363	924239	haematopoietic_and_lymphoid_tissue	lymphoid_neoplasm	blood	Myeloma	0.946444	1.097404	0.074681	0.031636	1.073794	2.139038	3.228113	0.771548	0.0012	1262511	0.868005	1.073794	1.277747
MDA-MB-415	924240	breast	carcinoma	breast	breast	0.85572	0.943277	0.124628	2.745898	4.359554	5.492533	6.943819	0.963036	0.0043	1101461	2.865553	4.359554	7.447353
NCI-H1975	924244	lung	carcinoma	lung	lung_NSCLC_adenocarcinoma	0.794629	0.480733	0.077795	0.252756	2.86618	5.5118	8.430331	0.846775	0.0031	1233298	2.155242	2.86618	4.130754
SW954	924247	vulva	carcinoma	urogenital_system	urogenital_system_other	0.910218	1.15958	0.112186	2.214107	4.006774	4.458942	5.629056	0.955543	0.0177	1049210	2.132436	4.006774	8.721248
MKN7	924250	stomach	carcinoma	digestive_system	stomach	0.842182	0.888061	0.127126	3.417015	5.26639	6.486067	8.089524	0.975023	0.0152	1049290	3.174229	5.26639	9.404371
MDA-MB-157	925338	breast	carcinoma	breast	breast	0.889161	1.040914	0.105096	2.426194	3.980574	4.891262	6.19111	0.95799	0.0184	991266	2.657581	3.980574	7.609229
MMAC-SF	925339	skin	malignant_melanoma	skin	melanoma	0.735672	0.448956	0.165765	-1.714474	1.184377	4.228049	7.63956	0.780368	0.0087	1049187	0.152955	1.184377	2.391835
MKN45	925340	stomach	carcinoma	digestive_system	stomach	0.823192	0.927855	0.083563	2.722294	4.27033	5.497638	6.999435	0.958187	0.001	1049289	3.049099	4.27033	6.543605
NCI-H810	925341	lung	carcinoma	lung	lung_NSCLC_large_cell	0.848519	0.992666	0.157217	3.624974	5.35719	6.39412	7.838537	0.9831	0.0544	1049311	2.885748	5.35719	9.890507
NBsusSR	925342	autonomic_ganglia	neuroblastoma	nervous_system	neuroblastoma	0.7518	0.971011	0.078393	-1.171911	0.090386	1.454514	2.917115	0.660155	0.0014	1309717	-0.14996	0.090386	0.330075
MFH-ino	925343	soft_tissue	malignant_fibrous_histiocytoma-pleomorphic_sarcoma	soft_tissue	soft_tissue_other	0.918665	1.071484	0.145485	1.070229	2.223453	3.267345	4.43608	0.880052	0.0188	1311795	1.665798	2.223453	3.282135
NOS-1	925345	bone	osteosarcoma	bone	osteosarcoma	0.999498	0.915928	0.083143	-0.073378	1.160236	2.391272	3.641922	0.757881	0.0051	1035739	0.928211	1.160236	1.401471
PANC-03-27	925346	pancreas	carcinoma	pancreas	pancreas	0.85333	1.002839	0.093536	2.190736	3.525709	4.657828	5.982581	0.941494	0.0066	1049320	2.661017	3.525709	5.24721
PANC-08-13	925347	pancreas	carcinoma	pancreas	pancreas	0.868269	1.021163	0.094708	3.45841	5.109517	6.05194	7.397545	0.981239	0.006	1049321	3.098708	5.109517	9.278131
PANC-10-05	925348	pancreas	carcinoma	pancreas	pancreas	0.846849	0.886465	0.054154	1.401914	2.764188	4.147775	5.632233	0.896747	0.0013	991300	2.43244	2.764188	3.264987
RO82-W-1	930083	thyroid	carcinoma	thyroid	thyroid	0.924017	0.785142	0.091403	0.049176	1.547312	3.04098	4.606221	0.791186	0.0027	991144	1.223021	1.547312	1.98069
SiHa	930297	cervix	carcinoma	urogenital_system	cervix	0.826295	0.841937	0.128181	2.288397	4.009549	5.378443	7.045428	0.945706	0.0023	1101351	2.706912	4.009549	6.543418
SKG-IIIa	930298	cervix	carcinoma	urogenital_system	cervix	0.927181	1.009665	0.100837	1.070057	2.277893	3.354009	4.564767	0.873221	0.0015	1101353	1.874541	2.277893	3.444327
TT	930299	thyroid	carcinoma	thyroid	thyroid	0.799801	0.812569	0.10836	0.600288	2.125875	3.698569	5.414963	0.840844	0.0337	1233268	1.660263	2.125875	2.73533
TE-9	946353	oesophagus	carcinoma	aero_digestive_tract	oesophagus	0.88403	0.813269	0.093751	1.017664	2.539383	3.984188	5.573189	0.865516	0.004	1049216	2.053759	2.539383	3.498129
TE-11	946354	oesophagus	carcinoma	aero_digestive_tract	oesophagus	0.845879	0.851695	0.082331	1.344355	2.796365	4.215959	5.773215	0.89316	0.005	1049213	2.311896	2.796365	3.628004
TE-6	946355	oesophagus	carcinoma	aero_digestive_tract	oesophagus	0.880383	1.048565	0.116615	1.052901	2.223535	3.329206	4.556572	0.872342	0.0032	1049214	1.777375	2.223535	3.084425
TE-12	946356	oesophagus	carcinoma	aero_digestive_tract	oesophagus	0.952913	1.331898	0.073174	1.269573	2.136579	2.993882	3.888556	0.890735	0.0014	1263491	1.940911	2.136579	2.395884
TC-YIK	946357	cervix	carcinoma	urogenital_system	cervix	0.842332	0.925733	0.126312	1.902569	3.637905	4.741331	6.257599	0.928761	0.0021	1323987	2.070819	3.637905	7.313597
YT	946358	haematopoietic_and_lymphoid_tissue	lymphoid_neoplasm	blood	lymphoid_neoplasm_other	0.793554	0.572127	0.0748	0.257743	2.483269	4.71612	7.177572	0.857633	0.0013	1257835	1.641288	2.483269	3.802316
BT-474	946359	breast	carcinoma	breast	breast	0.817375	0.756308	0.108411	1.885972	3.671811	5.254497	7.117372	0.919508	0.0021	1049313	2.641719	3.671811	5.706896
OAW-28	946360	ovary	carcinoma	urogenital_system	ovary	0.860297	0.934115	0.074547	1.314772	2.611799	3.902819	5.293354	0.893986	0.001	1049318	2.246324	2.611799	3.189741
LCLC-97TM1	946361	lung	carcinoma	lung	lung_NSCLC_large_cell	0.833754	0.869048	0.135163	0.939711	2.369264	3.771044	5.317319	0.861232	0.0064	1049274	1.795937	2.369264	3.284787
D-247MG	946367	central_nervous_system	glioma	nervous_system	glioma	0.808162	0.493731	0.122154	-0.803997	1.756736	4.340943	7.167029	0.792517	0.012	1035699	1.049575	1.756736	2.810117
D-263MG	946368	central_nervous_system	glioma	nervous_system	glioma	0.895008	1.079829	0.161542	-0.068114	1.025365	2.155627	3.334644	0.752804	0.0222	1035700	0.53806	1.025365	1.598822
D-336MG	946369	central_nervous_system	glioma	nervous_system	glioma	0.717942	0.399038	0.065981	-0.381639	2.852995	6.290784	10.100668	0.85173	0.0024	1035701	2.103901	2.852995	3.966909
D-392MG	946370	central_nervous_system	glioma	nervous_system	glioma	0.785576	0.591155	0.110056	0.791943	2.974434	5.154832	7.577578	0.869701	0.0018	1049162	2.102817	2.974434	4.346741
D-423MG	946372	central_nervous_system	glioma	nervous_system	glioma	0.809467	0.673201	0.111385	0.595562	2.461002	4.344981	6.41343	0.84551	0.0007	1049163	1.877016	2.461002	3.367856
D-502MG	946373	central_nervous_system	glioma	nervous_system	glioma	0.814303	0.662784	0.111133	0.725165	2.643432	4.514406	6.622528	0.855756	0.0327	1035702	1.961345	2.643432	3.933563
D-566MG	946377	central_nervous_system	glioma	nervous_system	glioma	0.878428	0.799258	0.071528	0.32537	1.866522	3.480926	5.172892	0.814327	0.0031	1101382	1.339594	1.866522	2.558865
CAMA-1	946382	breast	carcinoma	breast	breast	0.826651	0.785215	0.082735	2.986239	4.925212	6.377643	8.190856	0.960568	0.0096	1035686	3.249814	4.925212	8.039876
CTB-1	949088	haematopoietic_and_lymphoid_tissue	lymphoid_neoplasm	blood	B_cell_lymphoma	0.951416	1.47149	0.067311	1.614071	2.420149	3.169972	3.976037	0.920386	0.0037	1262847	2.197697	2.420149	2.930744
Caov-4	949090	ovary	carcinoma	urogenital_system	ovary	0.825838	0.689016	0.119102	2.027267	4.034863	5.8294	7.867203	0.921729	0.0053	1233272	2.715399	4.034863	6.302186
M059J	949094	central_nervous_system	glioma	nervous_system	glioma	0.821025	0.710078	0.082175	1.750695	3.579878	5.287501	7.231668	0.91154	0.0068	991265	2.694958	3.579878	5.221587
HAL-01	949153	haematopoietic_and_lymphoid_tissue	lymphoid_neoplasm	blood	lymphoblastic_leukemia	1.312362	1.790883	0.058978	0.592027	1.17786	1.742134	2.271282	0.804563	0.0024	1258003	1.052932	1.17786	1.287775
OMC-1	949154	cervix	carcinoma	urogenital_system	cervix	0.86643	1.010191	0.096862	2.277108	3.683894	4.72741	6.053653	0.949594	0.0085	1101397	2.676521	3.683894	5.975013
ES8	949155	bone	Ewings_sarcoma-peripheral_primitive_neuroectodermal_tumour	bone	ewings_sarcoma	1.291283	1.113771	0.121557	-1.266897	-0.314411	0.608694	1.481508	0.560045	0.0076	1101388	-0.600592	-0.314411	-0.043604
ES4	949156	bone	Ewings_sarcoma-peripheral_primitive_neuroectodermal_tumour	bone	ewings_sarcoma	0.960871	0.867147	0.146948	-0.089781	1.28246	2.505606	3.840952	0.768936	0.0188	1049167	0.677878	1.28246	2.30324
ES6	949157	bone	Ewings_sarcoma-peripheral_primitive_neuroectodermal_tumour	bone	ewings_sarcoma	0.848906	0.830267	0.082595	1.001117	2.46755	3.943708	5.53027	0.864981	0.0018	1101386	2.064778	2.46755	3.086785
ES1	949158	bone	Ewings_sarcoma-peripheral_primitive_neuroectodermal_tumour	bone	ewings_sarcoma	1.194183	1.158704	0.145912	-1.008106	-0.078329	0.839943	1.717956	0.588692	0.0128	1101385	-0.410737	-0.078329	0.221995
EW-7	949160	bone	Ewings_sarcoma-peripheral_primitive_neuroectodermal_tumour	bone	ewings_sarcoma	0.832959	0.812709	0.094795	1.264196	2.794024	4.296104	5.952204	0.885929	0.0029	1264555	2.242806	2.794024	3.701045
EW-3	949161	bone	Ewings_sarcoma-peripheral_primitive_neuroectodermal_tumour	bone	ewings_sarcoma	0.95897	1.002615	0.133043	-0.042858	1.165828	2.339686	3.565951	0.760234	0.0081	1257079	0.675944	1.165828	1.815254
EW-1	949163	bone	Ewings_sarcoma-peripheral_primitive_neuroectodermal_tumour	bone	ewings_sarcoma	0.907074	0.765566	0.119792	-0.174049	1.380096	2.899234	4.52331	0.774652	0.0045	1101389	0.927751	1.380096	2.036417
EW-18	949164	bone	Ewings_sarcoma-peripheral_primitive_neuroectodermal_tumour	bone	ewings_sarcoma	1.014411	0.986516	0.105338	-0.816933	0.319806	1.475152	2.625165	0.681002	0.0011	1264443	-0.008451	0.319806	0.619135
EW-16	949165	bone	Ewings_sarcoma-peripheral_primitive_neuroectodermal_tumour	bone	ewings_sarcoma	0.745635	0.698874	0.073353	0.703195	2.528137	4.457903	6.558903	0.862736	0.0048	1049173	1.949152	2.528137	3.194005
EW-13	949166	bone	Ewings_sarcoma-peripheral_primitive_neuroectodermal_tumour	bone	ewings_sarcoma	0.856906	1.038224	0.081883	4.152462	5.783189	6.748184	8.124007	0.989455	0.0072	1321943	3.456575	5.783189	10.055924
EW-22	949167	bone	Ewings_sarcoma-peripheral_primitive_neuroectodermal_tumour	bone	ewings_sarcoma	0.863097	0.698501	0.115122	-0.514387	1.209222	2.975372	4.838994	0.750144	0.0078	1035710	0.78656	1.209222	1.682768
EW-24	949168	bone	Ewings_sarcoma-peripheral_primitive_neuroectodermal_tumour	bone	ewings_sarcoma	0.845561	0.93901	0.102325	3.986152	5.719342	6.860145	8.358758	0.986908	0.0006	1323427	3.482675	5.719342	9.785616
LAN-6	949170	autonomic_ganglia	neuroblastoma	nervous_system	neuroblastoma	0.954701	0.922427	0.09498	0.405761	1.709988	2.808587	4.072942	0.813621	0.0086	1035724	1.29052	1.709988	2.606471
NB10	949171	autonomic_ganglia	neuroblastoma	nervous_system	neuroblastoma	0.823154	0.467766	0.149742	-2.533927	0.207829	3.067434	6.180102	0.723172	0.01	1101396	-0.678008	0.207829	1.184556
NB12	949172	autonomic_ganglia	neuroblastoma	nervous_system	neuroblastoma	0.593719	0.934348	0.051796	-2.373084	-0.972046	0.631809	2.454474	0.547922	0.001	1035735	-1.141793	-0.972046	-0.782528
NB6	949173	autonomic_ganglia	neuroblastoma	nervous_system	neuroblastoma	0.69801	0.345474	0.144259	-0.84154	3.181479	7.335408	12.113342	0.847184	0.0222	1035738	1.252997	3.181479	6.024466
NB7	949174	autonomic_ganglia	neuroblastoma	nervous_system	neuroblastoma	0.858762	0.909512	0.106793	1.2185	2.581701	3.884458	5.33916	0.88182	0.0068	1049192	2.020987	2.581701	3.586201
NB17	949175	autonomic_ganglia	neuroblastoma	nervous_system	neuroblastoma	0.964444	0.782012	0.136586	-0.777716	0.781974	2.188425	3.70948	0.713925	0.0198	1035737	0.233824	0.781974	1.500248
NB5	949176	autonomic_ganglia	neuroblastoma	nervous_system	neuroblastoma	0.79093	0.466593	0.178607	-1.334244	1.533795	4.37069	7.560307	0.786331	0.0186	1049191	0.333659	1.533795	3.19074

NB13	949177	autonomic_ganglia	neuroblastoma	nervous_system	neuroblastoma	0.994992	1.351386	0.177516	0.968451	2.001488	2.541268	3.385362	0.857344	0.0576	1049190	1.348108	2.001488	4.454399
NB14	949178	autonomic_ganglia	neuroblastoma	nervous_system	neuroblastoma	1.082365	1.54679	0.083971	0.792158	1.67952	2.110963	2.82384	0.824974	0.0044	1035736	1.231995	1.67952	3.473759
NB1	949179	autonomic_ganglia	neuroblastoma	nervous_system	neuroblastoma	0.90041	1.027777	0.076179	1.249209	2.406666	3.553669	4.780512	0.890011	0.0019	1311711	2.107549	2.406666	2.917587
RH-1	971773	soft_tissue	rhabdomyosarcoma	soft_tissue	rhabdomyosarcoma	1.011586	1.096424	0.163109	-0.436677	0.60374	1.628699	2.656016	0.726213	0.0075	1049125	0.105967	0.60374	1.105045
RH-18	971774	soft_tissue	rhabdomyosarcoma	soft_tissue	rhabdomyosarcoma	0.828173	0.644815	0.13773	0.395025	2.376181	4.306184	6.463529	0.833932	0.0043	1049126	1.656908	2.376181	3.620945
MZ2-MEL	971777	skin	malignant_melanoma	skin	melanoma	0.973125	1.287668	0.100202	1.320902	2.401224	2.968375	3.871374	0.901763	0.0082	1049188	1.800484	2.401224	4.511525
