# Supplementary material for: LIM kinase inhibitors disrupt mitotic microtubule organization and impair tumor cell proliferation
Source: Oncotarget. 2015 Nov 3;6(36):38469–86. doi: 10.18632/oncotarget.6288 (PMC4770715; doi:10.18632/oncotarget.6288)
Supplement: Supplementary file 10 [file oncotarget-06-38469-s010.pdf]

drug name	tissue name	ttest p value	ttest effect size	group mean	muts	ttest q value
CRT0105446	B_cell_leukemia	0.0132944	1.29817	6.7572	7	0.181907
CRT0105446	B_cell_lymphoma	0.000212202	1.62401	7.07214	10	0.00987567
CRT0105446	Bladder	0.215701	-0.409016	5.07509	18	0.692213
CRT0105446	Burkitt_lymphoma	0.00162662	1.38358	6.83537	10	0.0452533
CRT0105446	Hodgkin_lymphoma	0.138732	0.839126	6.30434	6	0.596773
CRT0105446	Myeloma	0.0152532	1.27234	6.73165	7	0.197932
CRT0105446	T_cell_leukemia	0.724764	-0.487337	4.98629	1	0.936995
CRT0105446	acute_myeloid_leukaemia	0.000740992	1.14178	6.58507	17	0.0258638
CRT0105446	adrenal_gland	0.292	1.45793	6.92859	1	0.754709
CRT0105446	anaplastic_large_cell_lymphoma	0.946918	0.0921545	5.5649	1	0.988023
CRT0105446	biliary_tract	0.725513	-0.217992	5.25655	5	0.936945
CRT0105446	bone_other	0.865618	0.234234	5.70676	1	0.968037
CRT0105446	breast	0.954678	0.0129787	5.48509	39	0.989638
CRT0105446	cervix	0.939355	-0.0306583	5.44279	12	0.986137
CRT0105446	chondrosarcoma	0.882825	-0.204008	5.26919	1	0.97195
CRT0105446	chronic_myeloid_leukaemia	0.28879	0.55728	6.02422	7	0.753385
CRT0105446	digestive_system_other	0.650389	-0.627281	4.84656	1	0.915458
CRT0105446	endometrium	0.975107	0.014486	5.48717	9	0.994452
CRT0105446	ewings_sarcoma	0.461166	-0.243554	5.23601	18	0.846438
CRT0105446	fibrosarcoma	0.934066	-0.0810335	5.3921	2	0.985388
CRT0105446	glioma	0.0287777	-0.466271	5.0386	45	0.281772
CRT0105446	haematopoietic_neoplasm_other	0.103665	1.30115	6.76808	3	0.532404
CRT0105446	hairy_cell_leukaemia	0.911511	-0.088947	5.38434	3	0.98076
CRT0105446	head_and_neck	0.99654	-0.00124715	5.47168	24	0.999058
CRT0105446	kidney	0.00069663	-1.03566	4.47038	21	0.0247742
CRT0105446	large_intestine	0.0231857	0.544459	5.9883	35	0.250557
CRT0105446	leukemia	0.990395	-0.00963534	5.46329	3	0.997406
CRT0105446	liver	0.114227	-0.829342	4.65239	7	0.555132
CRT0105446	lung_NSCLC_adenocarcinoma	0.453254	0.1526	5.61385	50	0.842236
CRT0105446	lung_NSCLC_carcinoid	0.762105	-0.242276	5.23172	3	0.946169
CRT0105446	lung_NSCLC_large_cell	0.511952	0.254056	5.72191	13	0.865939
CRT0105446	lung_NSCLC_not_specified	0.363884	0.563605	6.03219	5	0.798595
CRT0105446	lung_NSCLC_squamous_cell_carcinoma	0.14753	0.712012	6.17621	8	0.609689
CRT0105446	lung_small_cell_carcinoma	0.173032	0.336389	5.79235	33	0.644219
CRT0105446	lymphoblastic_T_cell_leukaemia	0.0495453	0.910343	6.37074	9	0.373788
CRT0105446	lymphoblastic_leukemia	0.106188	0.711348	6.17339	10	0.539962
CRT0105446	lymphoid_neoplasm_other	0.0615463	0.981277	6.44369	7	0.415167
CRT0105446	medulloblastoma	0.681249	0.284913	5.75606	4	0.924269
CRT0105446	melanoma	1.81E-08	-1.24085	4.30959	41	4.98E-06
CRT0105446	mesothelioma	0.982929	-0.0121376	5.46086	6	0.995561
CRT0105446	neuroblastoma	0.000420535	-0.954163	4.55799	27	0.0167165
CRT0105446	oesophagus	0.838971	0.0596594	5.53045	23	0.963681
CRT0105446	osteosarcoma	0.709737	-0.172782	5.30247	9	0.93249
CRT0105446	ovary	0.980786	0.00775504	5.48042	19	0.99562
CRT0105446	pancreas	0.0813102	0.591776	6.04933	17	0.478303
CRT0105446	prostate	0.849002	0.118215	5.5902	5	0.965738
CRT0105446	rhabdomyosarcoma	0.0163966	-1.35838	4.12692	6	0.207184
CRT0105446	skin_other	0.160399	-1.12265	4.35537	3	0.628319
CRT0105446	soft_tissue_other	0.0832915	-0.851135	4.63213	8	0.484913
CRT0105446	stomach	0.796682	0.087567	5.55818	17	0.954212

CRT0105446	testis	0.404281	-0.816681	4.65869	2	0.819138
CRT0105446	thyroid	0.0182628	-0.949085	4.54116	12	0.220068
CRT0105446	urogenital_system_other	0.245061	0.805926	6.2739	4	0.719188
CRT0105446	uterus	0.258458	-1.56339	3.91187	1	0.730634
