# Supplementary material for: LIM kinase inhibitors disrupt mitotic microtubule organization and impair tumor cell proliferation
Source: Oncotarget. 2015 Nov 3;6(36):38469–86. doi: 10.18632/oncotarget.6288 (PMC4770715; doi:10.18632/oncotarget.6288)
Supplement: Supplementary file 11 [file oncotarget-06-38469-s011.pdf]

drug name	tissue name	ttest p value	ttest effect size	group mean	muts	ttest q value
CRT0105950	B_cell_leukemia	0.536063	0.377715	3.33027	7	0.875302
CRT0105950	B_cell_lymphoma	0.000775579	1.71352	4.64399	10	0.0267817
CRT0105950	Bladder	0.395385	-0.326259	2.63928	18	0.815207
CRT0105950	Burkitt_lymphoma	1.79E-05	2.18071	5.10406	10	0.00148958
CRT0105950	Hodgkin_lymphoma	0.00759251	1.75432	4.69486	6	0.13099
CRT0105950	Myeloma	0.0880398	1.04027	3.98576	7	0.498694
CRT0105950	T_cell_leukemia	0.971877	0.0566838	3.01319	1	0.994027
CRT0105950	acute_myeloid_leukaemia	0.215928	-0.488266	2.48098	17	0.69206
CRT0105950	adrenal_gland	0.411681	-1.31961	1.63899	1	0.823871
CRT0105950	anaplastic_large_cell_lymphoma	0.968958	0.0625688	3.01906	1	0.993783
CRT0105950	biliary_tract	0.348646	0.675748	3.62719	5	0.790227
CRT0105950	bone_other	0.955621	-0.089475	2.86725	1	0.989527
CRT0105950	breast	0.0102747	0.679092	3.59531	39	0.156494
CRT0105950	cervix	0.321668	0.463722	3.41183	12	0.776301
CRT0105950	chondrosarcoma	0.137438	-2.38628	0.573948	1	0.596543
CRT0105950	chronic_myeloid_leukaemia	0.152281	-0.873275	2.09263	7	0.616374
CRT0105950	digestive_system_other	0.412135	-1.31832	1.64027	1	0.824125
CRT0105950	endometrium	0.67438	0.226549	3.18003	9	0.922419
CRT0105950	ewings_sarcoma	0.00593265	-1.05349	1.93201	18	0.109897
CRT0105950	fibrosarcoma	0.89876	-0.144757	2.81227	2	0.976377
CRT0105950	glioma	0.654459	-0.111064	2.85314	45	0.916623
CRT0105950	haematopoietic_neoplasm_other	0.0579561	-1.76034	1.2043	3	0.405836
CRT0105950	hairy_cell_leukaemia	0.90221	0.114233	3.0703	3	0.977635
CRT0105950	head_and_neck	0.568171	-0.190674	2.77289	24	0.88688
CRT0105950	kidney	0.0381735	-0.737483	2.24271	21	0.325569
CRT0105950	large_intestine	0.188863	0.366503	3.30354	35	0.665684
CRT0105950	leukemia	0.00141928	-2.95491	0.0151873	3	0.0408413
CRT0105950	liver	0.559123	0.356581	3.30936	7	0.88324
CRT0105950	lung_NSCLC_adenocarcinoma	0.526063	0.14986	3.09503	50	0.872324
CRT0105950	lung_NSCLC_carcinoid	0.377515	0.820212	3.77305	3	0.805911
CRT0105950	lung_NSCLC_large_cell	0.305085	0.46141	3.40885	13	0.765099
CRT0105950	lung_NSCLC_not_specified	0.957694	0.0382613	2.99456	5	0.990222
CRT0105950	lung_NSCLC_squamous_cell_carcinoma	0.267404	0.633554	3.58242	8	0.736322
CRT0105950	lung_small_cell_carcinoma	0.0936846	0.480554	3.41297	33	0.509488
CRT0105950	lymphoblastic_T_cell_leukaemia	0.968836	-0.0210676	2.93581	9	0.993793
CRT0105950	lymphoblastic_leukemia	0.00983136	-1.31841	1.65828	10	0.153039
CRT0105950	lymphoid_neoplasm_other	0.784903	-0.166626	2.79174	7	0.952717
CRT0105950	medulloblastoma	0.563291	-0.465644	2.49378	4	0.885127
CRT0105950	melanoma	0.0803235	-0.452659	2.53222	41	0.474845
CRT0105950	mesothelioma	0.841457	0.131802	3.08718	6	0.96404
CRT0105950	neuroblastoma	1.39E-05	-1.36205	1.6506	27	0.00124162
CRT0105950	oesophagus	0.00971934	0.879551	3.8053	23	0.151923
CRT0105950	osteosarcoma	0.0308612	-1.1621	1.81044	9	0.292058
CRT0105950	ovary	0.753299	0.117554	3.07074	19	0.943784
CRT0105950	pancreas	0.131989	0.594192	3.53538	17	0.586852
CRT0105950	prostate	0.631772	-0.345621	2.6136	5	0.909111
CRT0105950	rhabdomyosarcoma	0.014874	-1.60112	1.37011	6	0.194688
CRT0105950	skin_other	0.80415	0.230545	3.18608	3	0.956622
CRT0105950	soft_tissue_other	0.0238252	-1.28908	1.68323	8	0.254308
CRT0105950	stomach	0.0193567	0.921375	3.85409	17	0.226609

CRT0105950	testis	0.034808	-2.39731	0.566584	2	0.310812
CRT0105950	thyroid	0.230294	-0.561216	2.40564	12	0.704937
CRT0105950	urogenital_system_other	0.942686	-0.0579298	2.89901	4	0.986795
CRT0105950	uterus	0.601787	0.83894	3.79425	1	0.899498
